# Effective pest management approaches can mitigate honey bee (*Apis mellifera*) colony winter loss across a range of weather conditions in small-scale, stationary apiaries

**DOI:** 10.1093/jisesa/ieae043

**Published:** 2024-05-28

**Authors:** Darcy Gray, Sarah Goslee, Melanie Kammerer, Christina M Grozinger

**Affiliations:** Department of Entomology, Intercollege Graduate Degree Program in Ecology, Center for Pollinator Research, Huck Institutes of the Life Sciences, Pennsylvania State University, University Park, PA 16802, USA; USDA Agricultural Research Service, University Park, PA 16802, USA; USDA Agricultural Research Service, University Park, PA 16802, USA; Department of Entomology, Intercollege Graduate Degree Program in Ecology, Center for Pollinator Research, Huck Institutes of the Life Sciences, Pennsylvania State University, University Park, PA 16802, USA

**Keywords:** honey bee, colony loss, management, random forest

## Abstract

Managed honey bee (*Apis mellifera* L.) colonies in North America and Europe have experienced high losses in recent years, which have been linked to weather conditions, lack of quality forage, and high parasite loads, particularly the obligate brood parasite, *Varroa destructor*. These factors may interact at various scales to have compounding effects on honey bee health, but few studies have been able to simultaneously investigate the effects of weather conditions, landscape factors, and management of parasites. We analyzed a dataset of 3,210 survey responses from beekeepers in Pennsylvania from 2017 to 2022 and combined these with remotely sensed weather variables and novel datasets about seasonal forage availability into a Random Forest model to investigate drivers of winter loss. We found that beekeepers who used treatment against *Varroa* had higher colony survival than those who did not treat. Moreover, beekeepers who used multiple types of *Varroa* treatment had higher colony survival rates than those who used 1 type of treatment. Our models found weather conditions are strongly associated with survival, but multiple-treatment type colonies had higher survival across a broader range of climate conditions. These findings suggest that the integrated pest management approach of combining treatment types can potentially buffer managed honey bee colonies from adverse weather conditions.

## Introduction

Honey bees (*Apis mellifera* Linnaeus, Hymenoptera: Apidae) are critical pollinators for agricultural systems (Calderone & Smagghe, 2012, Hung et al., 2018), but beekeepers have reported high losses among their colonies in recent years in North America and Europe (Bruckner et al. 2022, Gray et al. 2023). Beekeepers in the United States typically report losing 30% of their colonies on average each winter despite intensive management inputs (Kulhanek et al. 2017, Bruckner et al. 2022). A better understanding of the drivers of colony loss and their interactions could benefit beekeepers by informing in-hive management and apiary placement decisions. Additionally, wild bees and other pollinators are often exposed to similar environmental stressors as honey bees (Woodcock et al. 2017, Evans et al. 2018, Kammerer et al. 2021), though the extent to which honey bees can serve as an indicator of wild bee declines is an ongoing area of debate (Wood et al. 2020, Kammerer et al. 2021, Jackson et al. 2022).

The seasonal growth and associated behaviors of honey bee colonies in temperate regions are well understood (Winston 1987, Grozinger et al. 2014, Döke et al. 2015). Colony population size increases in spring, and colonies reproduce by colony fission (i.e., swarming) once the colony reaches a density threshold. Colony size continues to grow over the summer as foragers collect and store pollen and nectar. In the fall, colonies taper brood production and shift to producing long-lived winter bees, whose extended lifespan carries the colony through the winter until early spring when the queens start laying eggs again (Mattila et al. 2001).

In studies modeling colony loss at large geographic scales, weather variables consistently rank among top predictors (Switanek et al. 2017, Van Esch et al. 2020, Calovi et al. 2021, Insolia et al. 2022). Relationships between weather and honey bee health are often nonlinear or seasonally specific and likely reflect both direct physiological and behavioral effects on honey bees, as well as indirect effects through floral resource availability (Corbet 1990) and host–parasite dynamics with *Varroa destructor*, the preeminent pest of honey bees in temperate regions. For example, increased rates of winter colony loss have been associated with high fall temperatures (DeGrandi-Hoffman and Curry 2004, Smoliński et al. 2021, Overturf et al. 2022), fewer summer foraging days (Becsi et al. 2021), and extremes of summer temperature and precipitation (Calovi et al. 2021). Colony performance has also been negatively associated with above-normal spring precipitation (Quinlan et al. 2022, 2023). Uncoupling the direct and indirect effects of weather on colony performance and survival can be challenging in these large-scale studies.

Several other factors also catalyze winter loss, including large populations of *V. destructor* and nuanced interactions between *Varroa*, weather, and colony nutritional status. Winter is costly and high risk for honey bees as the colonies must cluster to maintain a steady temperature (Gates 1914) and are reliant on their stored resources and the longevity of winter bees. *Varroa* populations grow with colonies over the spring and summer months and can overwhelm an untreated colony by reducing the lifespan of winter bees (Dainat et al. 2012), and *Varroa* has been shown to contribute significantly to winter colony loss in various geographic regions (Dahle 2010, Genersch et al. 2010, Guzmán-Novoa et al. 2010, Molineri et al. 2018, Hernandez et al. 2022). Furthermore, weather and landscape conditions can exacerbate *Varroa* infestations. Longer growing seasons can lead to higher *Varroa* loads within honey bee colonies due to the extension of the brood-rearing period (DeGrandi-Hoffman and Curry 2004, Nürnberger et al. 2019). Both *Varroa* infestation and lack of forage in the spring and summer reduce population size and small colonies struggle to thermoregulate and have lower overwintering success (Owens 1971). Furthermore, poor nutrition disturbs honey bee colonies’ ability to withstand *Varroa* infestations; several studies describe a connection between protein supplemented diets, *Varroa* levels, and deformed wing virus (a virus vectored by *Varroa* mites) (Janmaat and Winston 2000, DeGrandi-Hoffman et al. 2010) as well as between landscape quality and *Varroa* parasitization (Dolezal et al. 2019, Hernandez et al. 2023).

Mite infestations may also alter the way bees are experiencing their environment. *Varroa* parasitization impacts foraging ability in honey bees by impeding learning (Kralj et al. 2007), flight duration and homing ability (Kralj and Fuchs 2006), landing ability (Muijres et al. 2020), and waggle dance communication (Pusceddu et al. 2021), thus reducing honey bees’ ability to take advantage of the available forage in their landscape. *Varroa* also increases bees’ vulnerability to temperature (Aldea-Sánchez et al. 2021) and water stress (Annoscia et al. 2012). Thus, there are several mechanisms whereby *Varroa* infestation and consequently *Varroa* management strategies may interact with landscape and weather conditions to influence colony survival, but few studies have demonstrated this at a landscape scale (but see Dolezal et al. 2019).

Examining how weather and landscape impact colony survival under different *Varroa* management regimes can provide insights into whether and how these different factors interact, potentially informing treatment decisions. Surveys of beekeepers provide a valuable tool to assess environmental drivers of colony loss alongside management (Bruckner et al. 2022, Gray et al. 2023). We leveraged a unique dataset, an annual survey of Pennsylvania beekeepers from 2017 to 2022, and combined the survey results with satellite-derived remote sensing data and seasonal forage availability datasets using a Random Forest model. We built upon previous analysis of this survey data (Calovi et al. 2021), which highlighted the importance of summer weather conditions to winter colony survival. Our analysis uses 3 additional years of data and investigates the effect of management by separating colonies into 2 models based on their level of *Varroa* treatment. This approach allowed us to holistically predict loss in Pennsylvania, first asking how management decisions impact winter colony loss, and then investigating whether these management practices interact with landscape and weather factors in multiplicative or additive ways.

## Materials and Methods

### Survey Data

An annual survey on winter honey bee colony loss and beekeeping management has been sent to Pennsylvania beekeepers each spring for over 15 years; we used 6 years of data from the 2017 to 2022 surveys as these were conducted online and included apiary locations for some sites. The survey consists of 36 questions about beekeeping management and colony loss including pre-winter and post-winter colony numbers (November and April, respectively), beekeeper years of experience, *Varroa* treatment (binary), type of *Varroa* treatment, supplemental feed (binary), type of supplemental feed, and an option to submit apiary coordinates. Anonymized survey data, after preprocessing (see below), is provided in Supplementary Table S1 in Supplemental Materials.

### Preprocessing of Survey Data

From the survey responses, we calculated apiary survival rate (number of colonies post-winter/number of colonies pre-winter), *Varroa* treatment category, *Varroa* treatment types, and number of treatment types used. Mite treatment type was based on beekeeper responses to the question “what mite treatment(s) did you use?” for which participants could select multiple choices from a list. We categorized treatment into 3 categories of increasing intervention and toxicity: mechanical, soft chemical, and hard chemical (Underwood and López-Uribe 2019). Mechanical treatments described nonchemical interventions (i.e., drone brood removal, breaking of the brood cycle, screened bottom boards, powdered sugar). Soft chemical treatments were designated as naturally derived, organic compounds (i.e., thymol, formic acid, oxalic acid, or hop beta acids), which have been shown to have fewer lasting effects on bee health and less accumulation in honey and wax (Lindberg et al. 2000, Imdorf and Charrière 2003, Rosenkranz et al. 2010). Hard chemicals were synthetic chemicals (i.e., tau-fluvalinate, amitraz), which have historically been widely used and effective but are becoming less utilized due to the increasing prevalence of resistant *Varroa* populations (Hillesheim et al. 1996, Milani 1999) and concerns about negative impacts on bees due to persistence in wax (Bogdanov et al. 1998, Martel et al. 2007). Number of *Varroa* treatment types captures how many distinct treatment types a beekeeper used; this does not describe how many times a beekeeper treated for *Varroa*, which was not a question on the survey. For example, if a beekeeper responded that they treated with oxalic acid and formic acid, the number of treatment types is 2, even though a beekeeper may have used each treatment multiple times. Multiple-treatment type types within the same category, such as multiple soft chemicals, were considered as separate treatments, but multiple types of application for the same chemical, such as Oxalic (drip) and Oxalic (vapor), were not.

There were initially 3,230 beekeeper surveys with 23,922 total colonies recorded over the survey years of 2017–2022. Responses from beekeepers who reported using their colonies for migratory pollination services and beekeepers who reported having multiple apiaries were excluded. In the survey that was used, each beekeeper could only report 1 GPS location, and thus georeferenced locations of additional apiaries or migratory locations were not available. The single reported GPS location would not reflect the landscape and weather conditions, as they were exposed to throughout the year. Thus, the exclusion of this cohort ensures that the geospatial data corresponding to the points is accurate and that the statistical analysis of treatment and survival is reflective of apiaries within Pennsylvania, which was the focus of the study. Additionally, any records where the number of colonies in the spring exceeded the number of colonies in the preceding fall were excluded, as this could represent either incorrect record keeping or the addition of colonies from other apiaries. After these quality control measures, 3,047 responses with 11,799 colonies remained for analysis. The remaining apiaries ranged in size from 1 colony to 102 colonies, with a median of 3 colonies and a mean of 4.53. The survey data are anonymized, so while the same beekeepers are likely to repeat the survey in multiple years, we had to treat each entry as a unique apiary within each year.

We divided survey responses into subsets appropriate for 2 analyses: a nonspatial model examining the effects of beekeeper management and a spatial model including management and spatial variables (landscape and weather). Our nonspatial model included all 3,047 response records remaining after the filtering described above, while the spatial model included the 708 records for which the beekeeper treated for *Varroa* and provided apiary location information. For these georeferenced points, we standardized the coordinates and eliminated any that were outside Pennsylvania. To increase the strength of the model, each colony was used as an individual record in this analysis (*N* = 3,072), and survival was binary, where 0 was mortality and 1 was survival, as in Calovi et al. (2021).

### Climate Variables

For each apiary location in the georeferenced dataset, we derived climate variables from the PRISM climate dataset, which provides gridded daily coverage of temperature and precipitation for the conterminous United States at approximately 4-km resolution (PRISM Climate Group 2014). To understand the specific seasonal effects of weather on colony survival, we calculated total precipitation and mean temperature for the summer (June, July, August) and fall (September, October, November) preceding the survey year, and the winter (December, January, February) and spring (March, April, May) of the survey year. We additionally included the BIOCLIM weather variables (Fick and Hijmans 2017) that were both relevant for plant and bee phenology and that had sufficient variability across Pennsylvania. These were growing degree days, annual precipitation, and temperature seasonality (SD × 100).

### Landscape Variables

We used a 5 km buffer around each apiary to generate landscape metrics, since this contains 95% of a colony’s typical foraging (Visscher and Seeley 1982, Couvillon et al. 2015). We combined data on land cover with estimates of forage quality per land cover class to represent landscape-total forage available to honey bees (Lonsdorf et al. 2009, Koh et al. 2016). We used relative values of forage quality per land cover class per season from Koh et al. (2016) to convert the Cropland Data Layer (USDA NASS 2021) land cover to available forage. Prior to summing, we weighted values with an exponential decline function (Lonsdorf et al. 2009). We used CDL from the preceding year (since this was the time period when the bees foraged) and applied distance-weighting calculation before summing forage quality within 5 km of apiary locations. We calculated forage quality in spring, summer, and fall, with seasonal date ranges defined by Koh et al. (2016).

To capture developed land area, which presumably has low forage availability, we calculated the mean impervious surface value within 2.5 km of each apiary from the National Land Cover Dataset (NLCD) impervious surface map for 2019 (Dewitz and US Geological Survey 2021). The NLCD is only available every 5 years with the most recent data being from 2019, but we would not expect considerable changes over the study period. All model variables are presented in Table 1

**Table 1.** Weather, landscape, and management variables included in RF models of winter colony survival. Seasonal weather variables as well as several annual BIOCLIM variables are included. Summer (June, July, August) and fall (September, October, November) variables are for the year preceding the survey; Winter (December, January, February) and Spring (March, April, May) are for the survey year. All forage variables are for the preceding year

Category	Variable	Source
Annual weather	Annual precipitation	PRISM
	Growing degree days	PRISM
	Seasonality	PRISM
Seasonal weather	Spring total precipitation	PRISM
	Summer total precipitation	PRISM
	Fall total precipitation	PRISM
	Winter total precipitation	PRISM
	Spring mean temperature	PRISM
	Summer mean temperature	PRISM
	Fall mean temperature	PRISM
	Winter mean temperature	PRISM
Landscape	Spring forage score	NLCD & estimates of per-land cover class flower cover (Lonsdorf et al. 2009, Koh et al. 2016)
	Summer forage score	NLCD & and estimates of per-land cover class flower cover (Lonsdorf et al. 2009, Koh et al. 2016)
	Fall forage	NLCD & estimates of per-land cover class flower cover (Lonsdorf et al. 2009, Koh et al. 2016)
	Impervious surface	NLCD
Beekeeping management	Treatment type	Beekeeper survey
	Supplemental feed type	Beekeeper survey

### Statistical Analysis

To test the effect of *Varroa* treatment on survival, we used Mann–Whitney tests and Kruskal–Wallis 1-way analyses of variance in R Statistical Software v4.1.2 (R Core Team 2022). We estimated mean percent survival based on *Varroa* treatment (binary), *Varroa* treatment type, *Varroa* treatment category, number of *Varroa* treatment types, feed type, beekeeper experience (binned in 4 categories), supplemental feed (binary), supplemental feed type, and apiary size. We compared treatment type and category only among beekeepers who reported using only 1 type of treatment.

### Random Forest Model

We adapted the Random Forest (RF) classification model from Calovi et al. (2021) using the ranger package in R (Wright and Ziegler 2017). Random Forest models are strong in characterizing nonlinear relationships with many correlated predictor variables (Berk 2008, Shoemaker et al. 2018). Due to the high degree of variability between years, a single year does not act as a good testing dataset for the model, so instead we used 10-fold cross-validation to estimate error, ensuring approximately equal stratification of training and testing datasets across years. We first used 10-fold validation to tune our model to the best number of trees and variables to sample in each tree (mtry) and then used 10-fold cross-validation to generate model accuracy estimates (Wong and Yeh 2020). We stratified the groups by year to ensure a similar proportion of each year represented in each group because the weather was substantially different between years.

To determine variable importance for each variable in the model, the RF model reports premutation variable importance, which is the mean decrease in prediction accuracy across all trees when a predictor variable is randomly ordered. In our full model of all treated colonies, growing degree days and diversity of treatment types had the highest importance. We then separated our data into single-treatment type and multiple-treatment type colonies. Again, number of treatment types refers to the number of different types of *Varroa* treatment reportedly used, not to the number of applications of treatment. For example, colonies within the single-treatment type model may have been treated once or multiple times with the same type of treatment, as data on treatment quantity and frequency was not included in the survey. Thus, in comparing variable importance between these 2 models, we did not assess the effect of treatment quantity but rather treatment diversity. We included 16 variables in the single-treatment type model and 17 in the multiple-treatment type model as number of treatment types was only relevant for the latter. We repeated calculations of variable importance within a 50-fold cross-validation on each of 10 different random seeds to ensure the stability of the results (Strobl et al. 2007) and took the mean permutation importance value to rank the variables. The variance between the importance values was very low among model runs (see Supplementary Fig. S1). We generated the plots using the ggplot2, cowplot, and pdp packages (Wickham 2016, Greenwell 2017), with additional refinements in Adobe Illustrator 26.2.1.

### Partial Dependence

To examine the predicted relationship between individual variables and colony survival, we generated partial dependence plots (Greenwell 2017) for top variables for both the single-treatment type and multiple-treatment type models. We generated both single variable and bivariate partial dependence plots, to visualize how the predicted colony survival rate changes when either 1 or 2 variables, respectively, are varied while holding all other predictors constant.

## Results

### Beekeeping Management Variables

The average loss among nonmigratory, small-scale Pennsylvania beekeepers included in the survey was 50.3% with a standard deviation of 40.7; mean annual losses ranged from 43.4% in the 2017 survey to 55.3% in the 2019 survey. Apiaries that were ten colonies or fewer accounted for 91.9% of survey responses (2,801) and 79% (2,406) had 5 or fewer. Among the nonmigratory and nonmoved apiaries, the mean number of respondents to the survey was 508 per year. Most beekeepers in the survey (64.9%) had 1–5 years of experience once those with multiple apiaries and those with migratory operations were excluded.

**Fig. 1.**
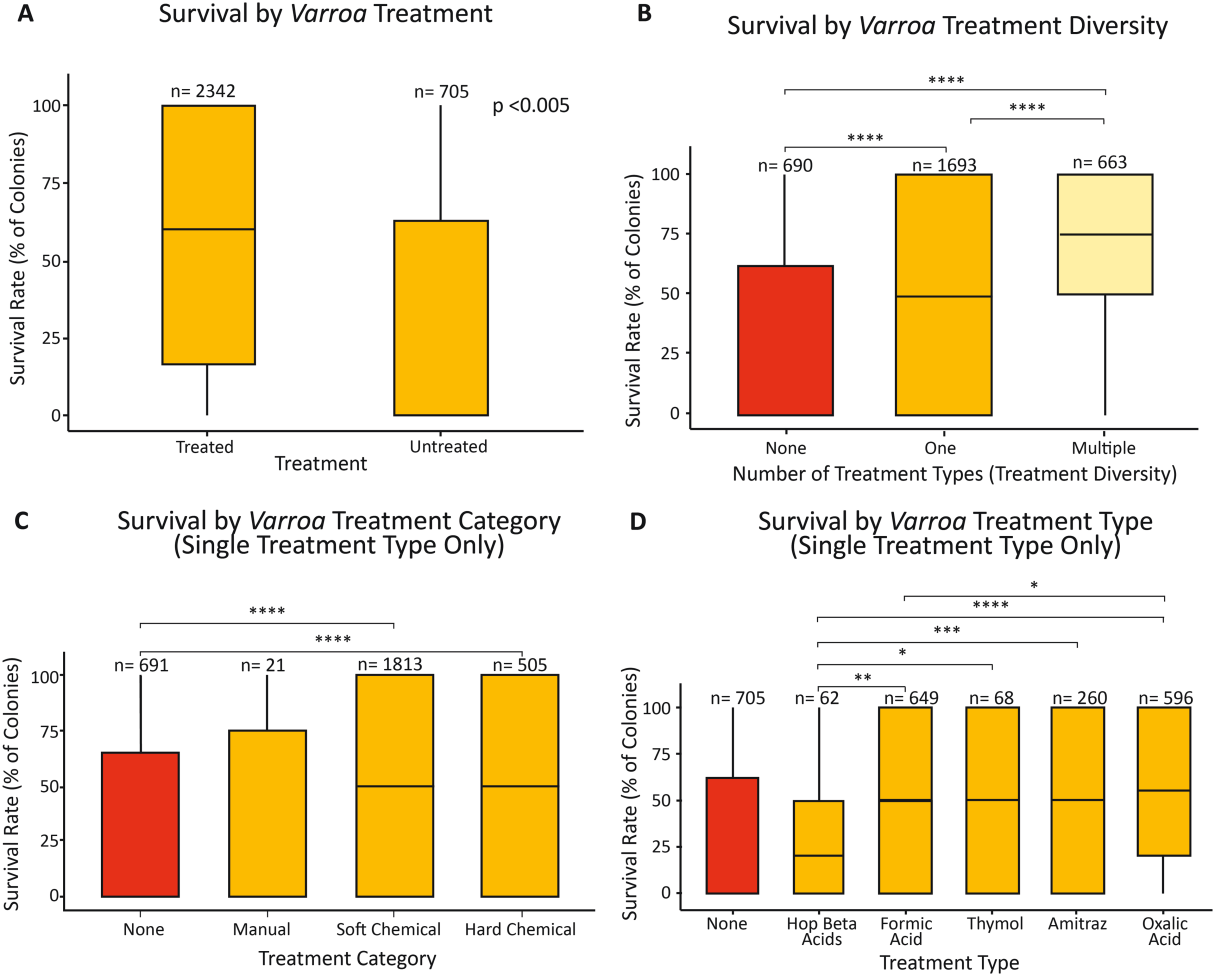
Box and whisker plots showing the statistical summaries of the winter colony survival rates relating to Varroa management practices. Boxes represent the interquartile range (IQR); the horizontal line represents the median and the whiskers represent minimum and maximum values 1.5 times the IQR. There was a significant increase in colony survival among beekeepers who treated for Varroa A) and beekeepers who used multiple types of treatment had higher colony survival than those who used no treatment or one type of treatment B). There was no significant difference between beekeepers who used hard and soft chemicals but both had significantly higher survival than untreated C). All treatment types had higher survival than none except hop beta acids D). *Degree of significance between variables connected with lines. (**** indicates p<0.0001, *** indicates p<0.001, ** indicates p <0.01 , * indicates p<0.05).

Apiaries treated to control *Varroa* mites had higher survival (mean = 56.0%, SD = 39.2, *n* = 2,342) than untreated apiaries (31.1%, SD = 39.8, *n* = 705, *P* < 2.2e-16, Fig. 1A). We next separated apiaries into those that received a single type of *Varroa* treatment and those that received multiple types of treatment. The survey did not request information on how frequently apiaries were treated, and thus “single-type” treatment apiaries may have received that treatment multiple times in the year. The survival rate for apiaries treated with multiple types of *Varroa* treatment (mean = 67.1%, SD = 34.7) was significantly higher than apiaries treated with 1 type of *Varroa* treatment (mean = 51.5%, SD = 40.0, *P* = 0.0008, Fig. 1B). There was no difference between colonies treated with 2, 3, and 4 types of treatment (*n* = 581, 77, 5, χ^2^ = 0.93, *P* = 0.62, data not shown).

Among those apiaries where only 1 type of chemical treatment was used, there was a significant difference in survival rate between categories of treatment from a Kruskal–Wallis test (χ^2^ = 10.28, *P* = 0.01); however, the pairwise comparisons between categories showed no significant differences in survival between groups. This discrepancy is likely due to the small sample size of mechanically treated colonies (*n* = 21). There was no significant difference in survival between apiaries treated with soft chemicals and those treated with hard chemicals (*P* = 0.37; Fig. 1C). Apiaries treated with chemical treatments (hard and soft) had a mean survival of 52% and those treated with mechanical treatments had a mean survival of 36.6% though this difference was marginally nonsignificant (*P* = 0.117). There was no difference (*P* = 0.41) between colonies treated with mechanical treatment and untreated apiaries, though few apiaries were mechanically treated (*n* = 21).

There was a significant difference in survival rate among types (χ^2^ = 19.882, *P* = 0.0005) of treatment for singly treated colonies (Fig. 1D). Apiaries treated only with hop beta acids (*n* = 62) did not have significantly different survival than untreated apiaries (*P* = 0.26) and had significantly lower survival than those treated with formic acid (*n* = 649), thymol (*n* = 68), amitraz (*n* = 260), or oxalic acid (*n* = 596, *P* < 0.05). Apiaries treated with only formic acid had lower survival than those treated with only oxalic acid (*P* = 0.02), and there was no significant difference between the remaining treatment types. Significance values for all pairwise comparisons of both *Varroa* treatment and supplemental feed are presented in the Supplementary Materials.

There was a significant positive relationship between apiary size and survival (*P* = 0.04, data not shown) and no relationship between beekeeper years of experience and survival (*P* = 0.95, data not shown). There was a significant difference in survival among type of supplemental feed (χ^2^ = 69.053, *P* < 0.001, Supplementary Fig. S2), and in particular, survival of apiaries fed Fondant or Sugar Candy (mean = 55.8%, SD = 40.3) was higher than unfed apiaries (mean = 47.6%, SD = 41.4, Supplementary Fig. S2). There was no difference between unfed colonies and all fed colonies however (mean = 50.5%, sd = 40.7, *P* = 0.35). The analysis of supplemental feed (binary) was highly unbalanced, as only 185 records were associated with apiaries that were not fed supplemental feed, while 2,862 records were. There was no significant relationship between survival and number of supplemental feed types (*P* = 0.65). Significance values for all analyses are presented in Supplementary Material.

### Effects of Landscape Conditions, Weather, and Management on Predicted Survival

Using a RF model, we examined the effect of 17 landscape, weather, and management variables on winter survival of apiaries managed using a single *Varroa* treatment type and apiaries managed using multiple types of *Varroa* treatment. For both models, we used the apiaries for which beekeepers reported that it was their only apiary, they were not part of migratory practice, and they volunteered their apiary location. The final single-treatment type model trained on 1,856 colonies used 2,500 trees with an mtry of 3 and the multiple-treatment type model trained on 1,054 colonies used 4,000 trees with an mtry of 3. The model for the single-treatment type apiaries predicted colony survival with a cross-validated out-of-bag (OOB) error of 19.5% and a prediction accuracy of 70.5%. The model for the multiple-treatment type apiaries predicted colony survival with a cross-validated OOB accuracy of 17% and a 76.3% prediction accuracy.

**Fig. 2.**
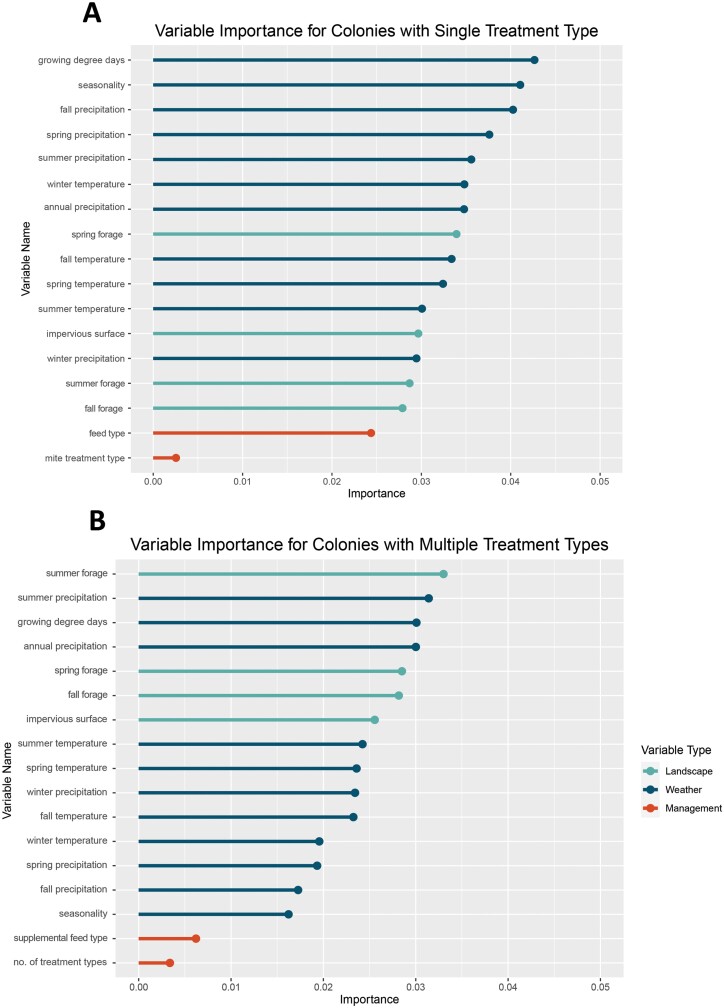
Variable importance of input variables using the permutation importance metric for A) single-treatment type and B) multiple-treatment type RF models of winter colony survival.

The most important variables for the single-treatment type model were growing degree days, seasonality, fall precipitation, spring precipitation, summer precipitation, and winter temperature (Fig. 2A). The most important variables for the multiple-treatment type model were summer forage, summer precipitation, growing degree days, annual precipitation, spring forage, and fall forage (Fig. 2B). The landscape variables had higher ranking importance in the multiple-treatment type model (3 of the top 6 variables) than in the single-treatment type model, where the top 7 variables were all weather-related.

**Fig. 3.**
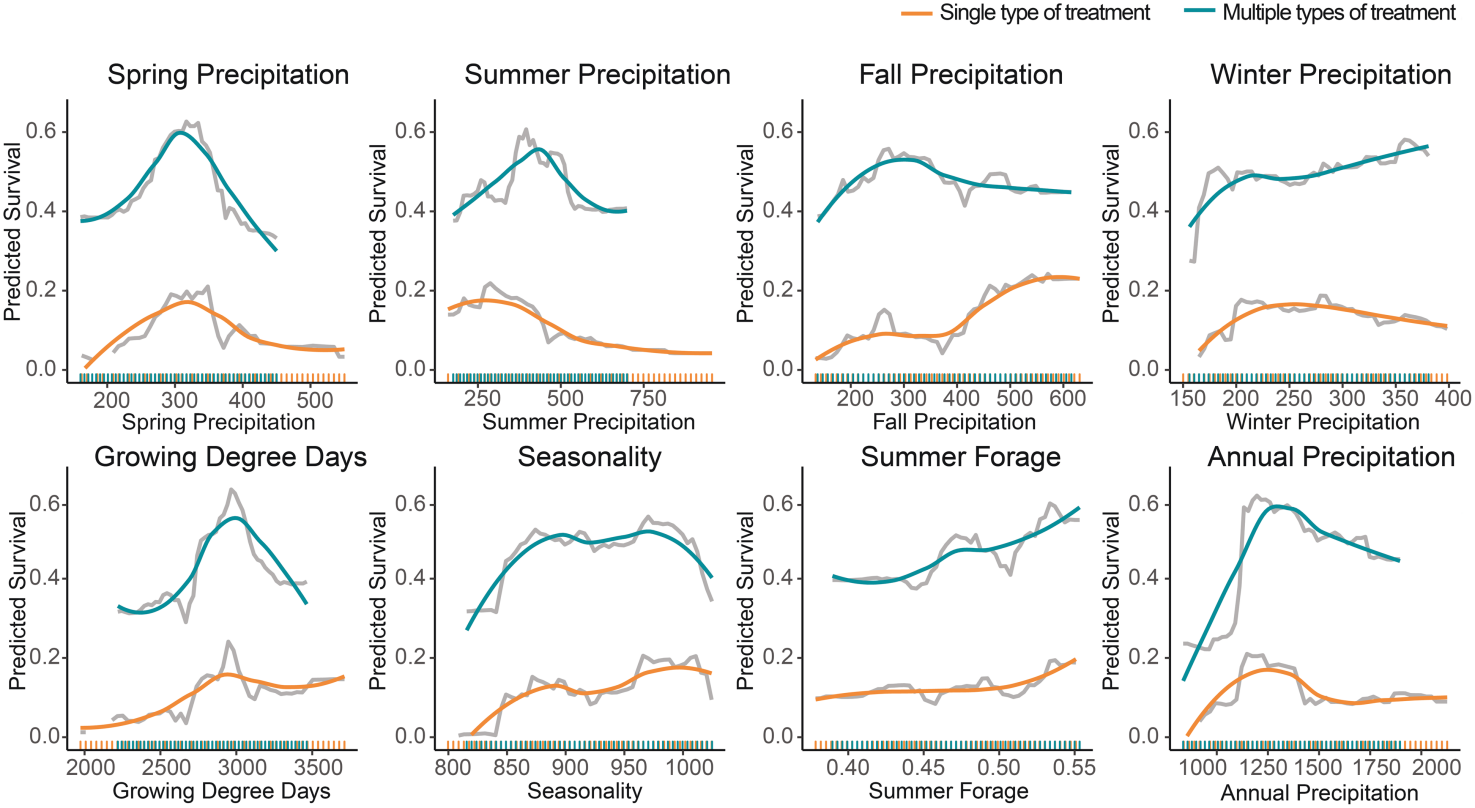
Partial dependence of model prediction on seasonal weather variables, select annual variables, and summer forage. RF predicted survival for single-treatment type colonies (smoothed in orange) and multiple-treatment type colonies (smoothed in blue) have similar trends, but survival for colonies treated with multiple types of treatment is higher. Rug diagrams on the x axis show the distribution of data for each variable for the single-treatment type (orange) and multiple-treatment type (blue) training datasets.

The single-treatment type and multiple-treatment type models show similar trends for many of the weather variables including a positive relationship between spring, fall, and winter precipitation and survival and a negative relationship between summer precipitation and survival (Fig. 3). Growing degree days and annual precipitation have nonlinear trends with survival, showing positive associations at intermediate values and then negative relationship at the low or high extremes. Predicted survival also increases with seasonality, suggesting that areas with larger differences in mean temperatures between each season have higher survival. All forage variables were positively associated with winter survival.

**Fig. 4.**
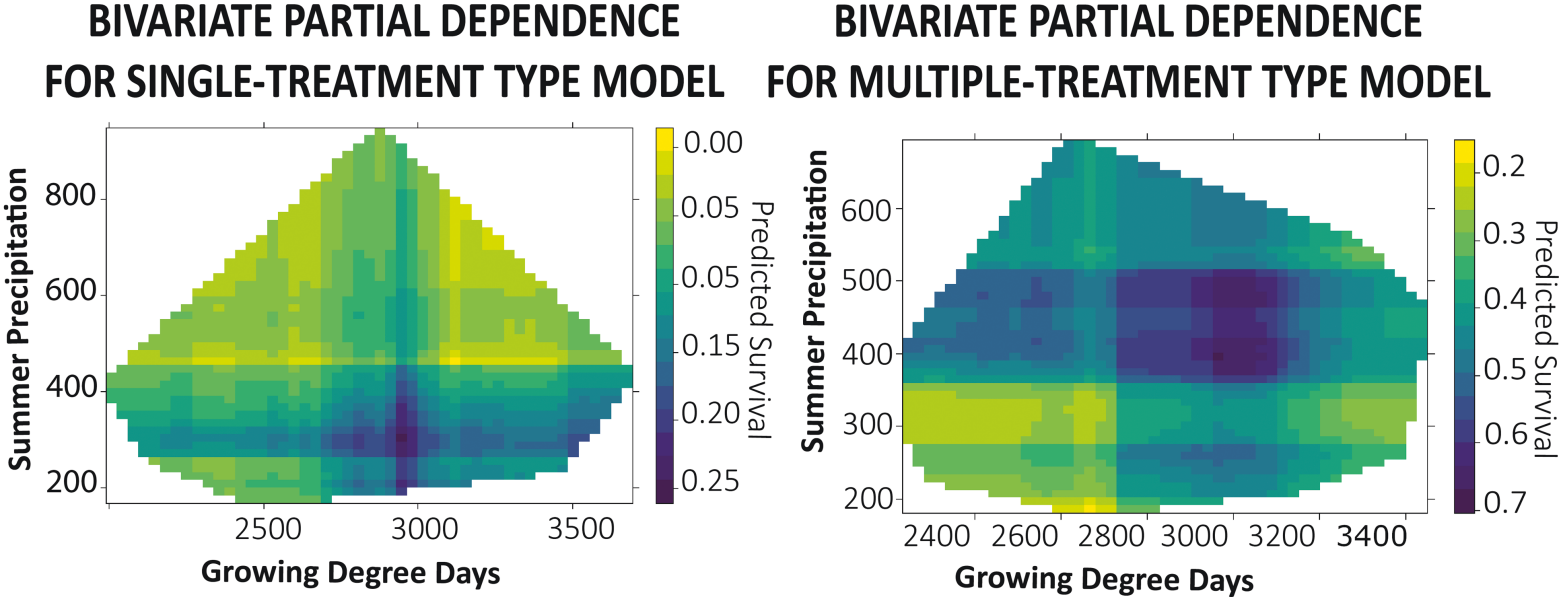
Bivariate partial dependence plots showing the combined influence of summer precipitation and growing degree days on A) single-treatment type (A) and B) multiple-treatment type predicted survival. High survival is in blue, and low survival is in yellow, and data outside the range of the training data are cropped from the predicted results. Middle range precipitation and growing degree-day values predict higher survival for both models, and the multiple-treatment model has higher predicted survival rates across the range.

**Fig. 5.**
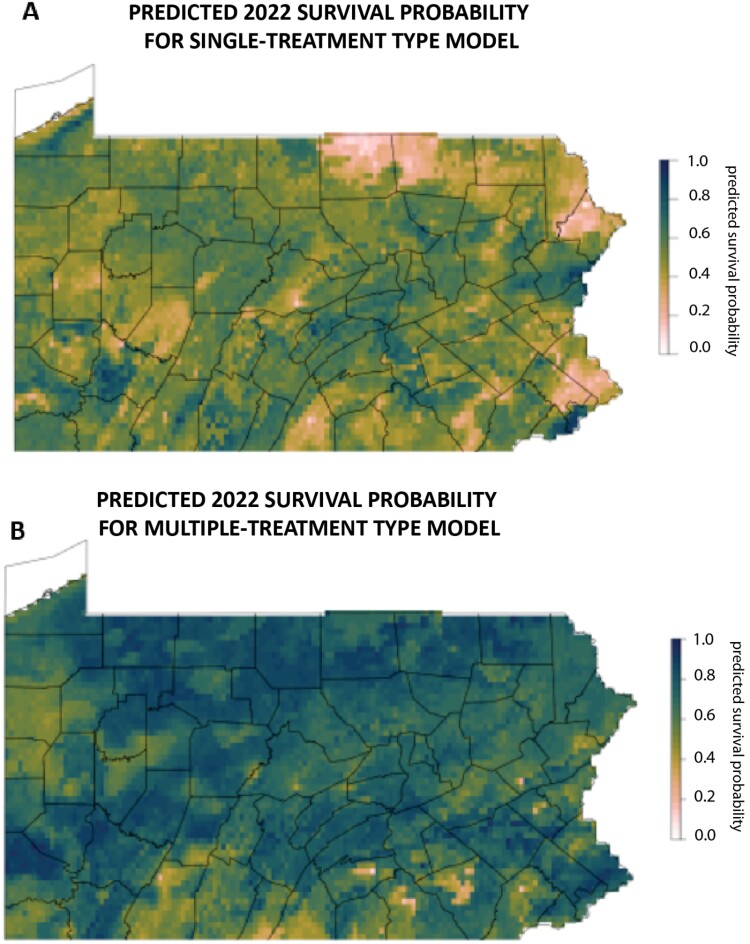
Predicted survival for 2022 across Pennsylvania for A) single-treatment type and B) multiple-treatment type treated apiaries from RF models based on exclusively climate variables. High predicted survival is in dark green, and low predicted survival is in white. The multiple-treatment type model has higher predicted survival across the state.

Bivariate partial dependence plots show predicted survival based on both growing degree days and summer precipitation. The plots demonstrated that multiple-treatment type apiaries have higher survival rates across a greater range of both growing degree days and precipitation (Fig. 4). Survival is lowest for the single-treatment type model when precipitation is over 450 mm and growing degree days are outside of a middle range of approximately 2,700–3,200 degree days. Survival is lowest for the multiple-treatment type model when summer precipitation and growing degree days are both low. Multiple-treatment type apiaries had a higher survival across a geographic range in Pennsylvania than single-treatment type apiaries (Fig. 5).

## Discussion

In an analysis of 3,047 survey results from beekeepers in Pennsylvania, we found that beekeepers who used *Varroa* treatment in their apiaries had significantly higher survival than those that did not treat. This underscores the importance of this parasite and is consistent with previous studies (Imdorf and Charrière 2003, Rosenkranz et al. 2010, Jacques et al. 2017, Beyer et al. 2018). We also found no significant difference between “hard” and “soft” chemical treatments, which again is consistent with previous studies supporting the efficacy of both treatment types (Imdorf and Charrière 2003, Vandervalk et al. 2014, Sabahi et al. 2020, Qadir et al. 2021, Underwood et al. 2023). Soft chemicals were as effective at increasing winter survival as hard chemicals, which have been shown to leave long-term residue in the colony (Martel et al. 2007) and continuous exposure to both pyrethroids and organophosphates has generated resistance of *Varroa* to these chemicals (Rinkevich 2020). We also found that beekeepers who used multiple *Varroa* treatment types had significantly higher colony survival than those who used a single *Varroa* treatment type. This finding underscores the potential power of using treatments in combination, which has been shown to effectively diminish *Varroa* populations and is often recommended to beekeepers as an Integrated Pest Management strategy (Delaplane et al. 2005, Roth et al. 2020, Jack and Ellis 2021).

In order to understand how treatment type diversity may influence survival, we divided our data into 2 models, based on whether the beekeeper used 1 or multiple types of treatment. We found no evidence of interactive or multiplicative effects of treatment diversity and weather variables on survival. The partial dependence trends were similar across both models, demonstrating that all treated colonies have similar relationships between these weather variables and survival. However, the multiple-treatment type colonies had higher survival on average and higher predicted survival across a greater range of temperature and precipitation conditions. These results suggest that the relationship between *Varroa* treatment diversity and weather is additive rather than synergistic, as the variation in survival across conditions is similar among partial dependence plots for both models. Colonies treated for *Varroa* have been shown to have higher survival than untreated ones across geographies with a range of climatic conditions (Genersch et al. 2010, Guzmán-Novoa et al. 2010, Dainat et al. 2012, Nazzi et al. 2012, van Dooremalen et al. 2012, Molineri et al. 2018, Hernandez et al. 2022). *Varroa* infestations suppress immunity (Gregory et al. 2005, Zaobidna et al. 2017, Annoscia et al. 2019), reduce nutrition and foraging abilities (Kralj and Fuchs 2006, Dolezal and Toth 2018, Yang et al. 2021), and diminish the lifespan of honey bees (Kovac and Crailsheim 1988, Amdam et al. 2004, van Dooremalen et al. 2012), all of which can also be exacerbated by adverse weather conditions (Le Conte and Navajas 2008, Clarke and Robert 2018, Szentgyörgyi et al. 2018). With this in mind, our results suggest that colonies adequately treated to prevent *Varroa* may be buffered against some of these deleterious effects of weather. While we did not find a significant interactive relationship between *Varroa* treatment and seasonal weather, previous work has demonstrated that timing of *Varroa* treatments influences their efficacy (Strange and Sheppard 2001, Currie and Gatien 2006). This is likely largely due to seasonal variations in the *Varroa* reproductive cycle and development of honey bee brood (Rosenkranz et al. 2010), but Beyer et al. (2018) also found an interactive relationship wherein weather conditions influenced treatment efficacy and Desai and Currie (2016) found an interaction between season and wintering method on concentration of deformed wing virus.

Forage availability had higher variable importance in the multiple-treatment type model than in the single-treatment type model. This suggests that in colonies where *Varroa* was managed in a more integrated manner, using multiple-treatment types, the relative importance of weather versus landscape variables may have shifted, allowing the relationships between landscape and colony survival to be illuminated, although it should be noted that the treatment quantity and frequency is not known, and that treatment diversity may not be related to treatment intensity. Predicted survival increased with forage score for spring, summer, and fall in our study, which is consistent with previous work establishing links between forage availability in the landscape improving honey bee colony health (Donkersley et al., 2014, St Clair et al. 2020, Ochungo et al. 2022). Increased nutrition can mitigate the impacts of *Varroa* infestation as well (Aronstein et al. 2012, DeGrandi-Hoffman and Chen 2015, Dolezal and Toth 2018). Indeed, DeGrandi-Hoffman et al. (2020) found that survival of fed colonies was higher than unfed colonies, despite fed colonies having higher *Varroa* populations and deformed wing virus levels. Furthermore, Hernandez et al. (2023) demonstrated that increased forage quality led to higher populations of honey bees and consequently higher resistance in the population against *Varroa*. Future work should investigate these links, particularly the influence of landscape quality and supplemental feed on the ability of a colony to withstand *Varroa*. However, in our study, forage availability was less important for the survival of single-treated colonies than the weather was, suggesting that management strategies that keep *Varroa* below a certain threshold are necessary to obtain the benefits of landscape-scale floral resources.

Our results also support evidence found in other studies that the effect of weather on colony survival varies seasonally (Switanek et al. 2017, Beyer et al. 2018, Becsi et al. 2021) and highlights the value of including seasonal variables in models of colony survival. While spring, fall, and winter precipitation increased survival, summer precipitation decreased colony survival, consistent with a previous analysis of the first several years of this data (Calovi et al. 2021). Our finding of a detrimental effect of summer precipitation is consistent with some previous studies (Beyer et al. 2018, Becsi et al. 2021), but not others (Quinlan et al. 2023 found no effect of summer weather on winter cluster size). Many consecutive days of summer rain may decrease survival by reducing bee foraging, leading to reduced brood production and less food stored for winter. Our finding that spring, autumn, and winter precipitation supports higher survival is consistent with Switanek et al. (2017), which found that across all seasons, precipitation was linked to higher winter survival, but contrasts with other recent work showing that above-normal spring precipitation is linked to higher mortality (Quinlan et al. 2023, Quinlan and Grozinger 2023) possibly due to spring rain encouraging grasses, but not flowering plants. Furthermore, we found that winter temperature and survival had a nonlinear relationship, with high and low winter temperatures increasing colony loss. This may reflect that honey bees can succumb to overly cold winters (Southwick and Heldmaier 1987), but that there is a necessity for the temperature to stay consistently low enough for the bees to remain in their winter cluster to minimize energy consumption (Currie et al. 1992). Differences in findings about the effects of weather across these studies may be partially due to geographic differences between studies as different ecoregions can vary in overall baseline climatic conditions as well as plant communities and land use types, which in turn mediate the impacts of weather (Quinlan et al. 2022).

In diverse agricultural systems, deliberate and proactive management can reduce the impacts of climate change (Lipper et al. 2014, Malhi et al. 2021). In many examples of climate-smart agricultural adaptations, the extent of the management intervention contributes to the resilience of the system (Zilberman et al. 2018). For managed honey bee systems, controlling the introduced pest *V. destructor* augments overwintering survival (Rosenkranz et al. 2010, Jacques et al. 2017, Sabahi et al. 2020) and most beekeepers in the United States use some kind of treatment regime for the pest (Underwood et al. 2019). To our knowledge, our study is the first to show that not only treatment, but using multiple-treatment type types, bolsters winter survival of honey bees. The adaptive management capacity of a beekeeper has been demonstrated as integral to their success in the face of uncertain or variable conditions (Kouchner et al. 2019), and while weather conditions (which are outside of a beekeepers’ control) impacted colony survival considerably in our models, *Varroa* treatment is an effective way to increase survival across a range of otherwise-deleterious weather conditions.

Our study is limited in that we lacked data on some key aspects of beekeeping management and colony health, including mite counts, queen genotypes, honey harvest quantities, and number and timing of *Varroa* treatments applied. While the weather variables had higher predictive power on survival in the single-treatment type model than in the multiple-treatment type model, comparison of variable importance between models should be cautiously interpreted as the 2 models are trained on separate sets of data. Without data on treatment quantity, we cannot determine whether the results we report are effects of combining treatments or are caused by increased treatment quantity or time spent treated in colonies with multiple types of treatment. Beekeepers in the single-treatment type group may have treated repeatedly or heavily, while those with multiple-treatment types may have applied treatment less often, or vice versa. Furthermore, variable importance scores can be inconsistent in RF models, but we addressed this by calculating variable importance using 50-fold cross-validation on 10 random seeds and by using the unbiased permutation importance metric (Strobl et al. 2007). Our data are self-reported by beekeepers across Pennsylvania, which creates room for error and some responses, such as number of treatment types, may be confounded by general beekeeping effort and investment. Additionally, due to regulatory changes in the United States which have limited the amount of data that is publicly available on pesticide use in recent years (Hitaj et al. 2020), we were not able to estimate insecticide exposure. Finally, seasonal floral resources values are derived using expert opinion and based on the Cropland Data Layer and thus may not always capture habitat at a suburban and urban scale; future studies should incorporate finer resolution landscape data.

Overall, our study demonstrates the role of beekeeping management in buffering honey bee colonies against adverse weather conditions in temperate regions. In the context of the growing evidence that increasing climate variation may impact bee health (reviewed in Le Conte and Navajas 2008, Cunningham et al. 2022, de Jongh et al. 2022), these results underscore the ability of Integrated Pest Management strategies to increase the survival of honey bees even in adverse weather conditions.

## Supplementary Material

ieae043_suppl_Supplementary_Material

## References

[CIT0001] Aldea-Sánchez P , Ramírez-Cáceres GE, Rezende EL, Bozinovic F. Heat tolerance, energetics, and thermal treatments of honeybees parasitized with *Varroa*. Front Ecol Evol. 2021:9:656504.

[CIT0002] Amdam GV , Hartfelder K, Norberg K, Hagen A, Omholt SW. Altered physiology in worker honey bees (Hymenoptera: Apidae) infested with the mite *Varroa destructor* (Acari: Varroidae): a factor in colony loss during overwintering? Apiculture and Social Insects. J Econ Entomol. 2004:97(3):741–747. 10.1093/jee/97.3.741 15279246

[CIT0003] Annoscia D , Brown SP, Di Prisco G, De Paoli E, Del Fabbro S, Frizzera D, Zanni V, Galbraith DA, Caprio E, Grozinger CM, et al. Haemolymph removal by *Varroa* mite destabilizes the dynamical interaction between immune effectors and virus in bees, as predicted by Volterra’s model. Proc R Soc B Biol Sci. 2019:286(1901):20190331. 10.1098/rspb.2019.0331 PMC6501932 30991929

[CIT0004] Annoscia D , Del Piccolo F, Nazzi F. How does the mite *Varroa destructor* kill the honeybee *Apis mellifera*? Alteration of cuticular hydrcarbons and water loss in infested honeybees. J Insect Physiol. 2012:58(12):1548–1555. 10.1016/j.jinsphys.2012.09.008 23041382

[CIT0005] Aronstein KA , Saldivar E, Vega R, Westmiller S, Douglas AE. How *Varroa* parasitism affects the immunological and nutritional status of the honey bee, *Apis mellifera*. Insects. 2012:3(3):601–615. 10.3390/insects3030601 26466617 PMC4553578

[CIT0006] Becsi B , Formayer H, Brodschneider R. A biophysical approach to assess weather impacts on honey bee colony winter mortality. R Soc Open Sci. 2021:8(9):210618. 10.1098/rsos.210618 34631120 PMC8483266

[CIT0007] Berk R. Statistical learning from a regression perspective, springer series in statistics. New York (NY): Springer New York; 2008.

[CIT0008] Beyer M , Junk J, Eickermann M, Clermont A, Kraus F, Georges C, Reichart A, Hoffmann L. Winter honey bee colony losses, *Varroa destructor* control strategies, and the role of weather conditions: results from a survey among beekeepers. Res Vet Sci. 2018:118:52–60. 10.1016/j.rvsc.2018.01.012 29421484

[CIT0009] Bogdanov S , Kilchenmann V, Imdorf A. Acaricide residues in some bee products. J Apic Res. 1998:37(2):57–67. 10.1080/00218839.1998.11100956

[CIT0010] Bruckner S , Wilson M, Aurell D, Rennich K, vanEngelsdorp D, Steinhauer N, Williams GR. A national survey of managed honey bee colony losses in the USA: results from the Bee Informed Partnership for 2017–18, 2018–19, and 2019–20. J Apic Res. 2022:62(3):429–443. 10.1080/00218839.2022.2158586

[CIT0011] Calovi M , Grozinger CM, Miller DA, Goslee SC. Summer weather conditions influence winter survival of honey bees (*Apis mellifera*) in the northeastern United States. Sci Rep. 2021:11(1):1553. 10.1038/s41598-021-81051-8 33452352 PMC7811010

[CIT0106] Calderone, N. W, Smagghe G. 2012. Insect Pollinated Crops, Insect Pollinators and US Agriculture: Trend Analysis of Aggregate Data for the Period 1992–2009. PLoS ONE 7:e37235–e37235. 22629374 10.1371/journal.pone.0037235 PMC3358326

[CIT0012] Clarke D , Robert D. Predictive modelling of honey bee foraging activity using local weather conditions. Apidologie. 2018:49(3):386–396. 10.1007/s13592-018-0565-3

[CIT0013] Corbet SA. Pollination and the weather. Isr J Bot. 1990:39(1–2):13–30.

[CIT0014] Couvillon MJ , Riddell Pearce FC, Accleton C, Fensome KA, Quah SKL, Taylor EL, Ratnieks FLW. Honey bee foraging distance depends on month and forage type. Apidologie. 2015:46(1):61–70. 10.1007/s13592-014-0302-5

[CIT0015] Cunningham MM , Tran L, McKee CG, Ortega Polo R, Newman T, Lansing L, Griffiths JS, Bilodeau GJ, Rott M, Marta Guarna M. Honey bees as biomonitors of environmental contaminants, pathogens, and climate change. Ecol Indic. 2022:134:108457. 10.1016/j.ecolind.2021.108457

[CIT0016] Currie RW , Gatien P. Timing acaricide treatments to prevent *Varroa destructor* (Acari: Varroidae) from causing economic damage to honey bee colonies. *The Canadian Entomologiest.* 2006:138(2):238–252. 10.4039/n05-024

[CIT0017] Currie RW , Spivak M, Reuter GS. Winter management of honey bee colonies in the hive and the honey bee: a new book on beekeeping which continues the tradition of Langstroth on the hive and the honey bee. Hamilton, Illinois: Dadant & Sons; 1992. p. 629–670.

[CIT0018] Dahle B. The role of *Varroa destructor* for honey bee colony losses in Norway. J Apic Res. 2010:49(1):124–125. 10.3896/ibra.1.49.1.26

[CIT0019] Dainat B , Evans JD, Chen YP, Gauthier L, Neumanna P. Dead or alive: deformed wing virus and *Varroa destructor* reduce the life span of winter honeybees. Appl Environ Microbiol. 2012:78(4):981–987. 22179240 10.1128/AEM.06537-11 PMC3273028

[CIT0020] de Jongh EJ , Harper SL, Yamamoto SS, Wright CJ, Wilkinson CW, Ghosh S, Otto SJG. One Health, one hive: a scoping review of honey bees, climate change, pollutants, and antimicrobial resistance. PLoS One. 2022:17(2):e0242393. 10.1371/journal.pone.0242393 35171904 PMC8849492

[CIT0021] DeGrandi-Hoffman G , Chen Y. Nutrition, immunity and viral infections in honey bees. Curr Opin Insect Sci. 2015:10:170–176. 10.1016/j.cois.2015.05.007 29588005

[CIT0022] DeGrandi-Hoffman G , Chen Y, Huang E, Huang MH. The effect of diet on protein concentration, hypopharyngeal gland development and virus load in worker honey bees (*Apis mellifera* L.). J Insect Physiol. 2010:56(9):1184–1191. 10.1016/j.jinsphys.2010.03.017 20346950

[CIT0023] DeGrandi-Hoffman G , Corby-Harris V, Chen Y, Graham H, Chambers M, Watkins deJong E, Ziolkowski N, Kang Y, Gage S, Deeter M, et al. Can supplementary pollen feeding reduce *Varroa* mite and virus levels and improve honey bee colony survival? Exp Appl Acarol. 2020:82:455–473. 33125599 10.1007/s10493-020-00562-7 PMC7686192

[CIT0024] DeGrandi-Hoffman G , Curry R. A mathematical model of *Varroa* mite (*Varroa destructor* Anderson and Trueman) and honeybee (*Apis mellifera* L.) population dynamics. Int J Acarol. 2004:30(3):259–274. 10.1080/01647950408684393

[CIT0025] Delaplane KS , Berry JA, Skinner JA, Parkman JP, Hood WM. Integrated pest management against *Varroa destructor* reduces colony mite levels and delays treatment threshold. J Apic Res. 2005:44(4):157–162.

[CIT0026] Desai SD , Currie RW. Effects of wintering environment and parasite-pathogen interactions on honey bee colony loss in north temperate regions. PLoS One. 2016:11(7):e0159615. 10.1371/journal.pone.0159615 27448049 PMC4957765

[CIT0027] Dewitz J ; U.S. Geological Survey. National Land Cover Database (NLCD) 2019 products (ver. 2.0, June 2021). U.S. Geological Survey data release; 2021.

[CIT0028] Döke MA , Frazier M, Grozinger CM. Overwintering honey bees: biology and management. Curr Opin Insect Sci. 2015:10:185–193. 10.1016/j.cois.2015.05.014 29588007

[CIT0029] Dolezal AG , St Clair AL, Zhang G, Toth AL, Matthew EO. Native habitat mitigates feast-famine conditions faced by honey bees in an agricultural landscape. Proc Natl Acad Sci USA. 2019:116(50):25147–25155. 31767769 10.1073/pnas.1912801116 PMC6911205

[CIT0030] Dolezal AG , Toth AL. Feedbacks between nutrition and disease in honey bee health. Curr Opin Insect Sci. 2018:26:114–119. 10.1016/j.cois.2018.02.006 29764650

[CIT0031] Evans E , Smart M, Cariveau D, Spivak M. Wild, native bees and managed honey bees benefit from similar agricultural land uses. Agric Ecosyst Environ. 2018:268:162–170. 10.1016/j.agee.2018.09.014

[CIT0109] Donkersley P , Rhodes G, Pickup RW, Jones KC, Wilson K. Honeybee nutrition is linked to landscape composition. Ecology and Evolution 2014:4:4195–4206 25505544 10.1002/ece3.1293 PMC4242570

[CIT0032] Fick SE , Hijmans RJ. WorldClim 2: new 1-km spatial resolution climate surfaces for global land areas. Int J Climatol. 2017:37(12):4302–4315. 10.1002/joc.5086

[CIT0033] Gates BN. The temperature of the bee colony. Bulletin 96. Washington, D.C.: United States Department of Agriculture; 1914.

[CIT0034] Genersch E , Von Der Ohe W, Kaatz H, Schroeder A, Otten C, Büchler R, Berg S, Ritter W, Mühlen W, Gisder S, et al. The German bee monitoring project: a long term study to understand periodically high winter losses of honey bee colonies. Apidologie. 2010:41(3):332–352. 10.1051/apido/2010014

[CIT0035] Gray A , Adjlane N, Arab A, Ballis A, Brusbardis V, Bugeja Douglas A, Cadahía L, Charrière JD, Chlebo R, Coffey MF, et al. Honey bee colony loss rates in 37 countries using the COLOSS survey for winter 2019–2020: the combined effects of operation size, migration and queen replacement. J Apic Res. 2023:62(2):204–210.

[CIT0036] Greenwell BM. pdp: an R package for constructing partial dependence plots. R J. 2017:9(1):421–436. 10.32614/rj-2017-016

[CIT0037] Gregory PG , Evans JD, Rinderer T, De Guzman L. Conditional immune-gene suppression of honeybees parasitized by *Varroa* mites. J Insect Sci. 2005:5(1):5. 16299597 10.1093/jis/5.1.7 PMC1283888

[CIT0038] Grozinger CM , Richards J, Mattila HR. From molecules to societies: mechanisms regulating swarming behavior in honey bees (*Apis* spp.). Apidologie. 2014:45(3):327–346. 10.1007/s13592-013-0253-2

[CIT0039] Guzmán-Novoa E , Eccles L, Calvete Y, McGowan J, Kelly PG, Correa-Benítez A. *Varroa destructor* is the main culprit for the death and reduced populations of overwintered honey bee (*Apis mellifera*) colonies in Ontario, Canada. Apidologie. 2010:41(4):443–450. 10.1051/apido/2009076

[CIT0040] Hernandez J , Hattendorf J, Aebi A, Dietemann V. Compliance with recommended *Varroa destructor* treatment regimens improves the survival of honey bee colonies over winter. Res Vet Sci. 2022:144:1–10. 10.1016/j.rvsc.2021.12.025 35032751

[CIT0041] Hernandez J , Varennes YD, Aebi A, Dietemann V, Kretzschmar A. Agroecological measures in meadows promote honey bee colony development and winter survival. Ecosphere. 2023:14:e4396.

[CIT0042] Hillesheim E , Ritter W, Bassand D. First data on resistance mechanisms of *Varroa jacobsoni* (OUD.) against tau-fluvalinate. Exp Appl Acarol. 1996:20(5):283–296. 10.1007/bf00052878

[CIT0043] Hitaj C , Smith DJ, Code A, Wechsler S, Esker PD, Douglas MR. Sowing uncertainty: what we do and don’t know about the planting of pesticide-treated seed. Bioscience. 2020:70(5):390–403. 10.1093/biosci/biaa019

[CIT0044] Imdorf A , Charrière J-D. Alternative *Varroa* control. *American Bee Journal*. Hamilton, Illinois; 2003.

[CIT0107] Hung KJ , Kingston JM, Albrecht M, Holway DA, Kohn JR. The worldwide importance of honey bees as pollinators in natural habitats. Proceed Royal Soc B: Biol Sci. 2018:285:20172140–20172140 10.1098/rspb.2017.2140 PMC5784195 29321298

[CIT0045] Insolia L , Molinari R, Rogers SR, Williams GR, Chiaromonte F, Calovi M. Honey bee colony loss linked to parasites, pesticides and extreme weather across the United States. Sci Rep. 2022:12(1):20787. 10.1038/s41598-022-24946-4 36456591 PMC9714769

[CIT0046] Jack CJ , Ellis JD. Integrated pest management control of *Varroa destructor* (Acari: Varroidae), the most damaging pest of (*Apis mellifera* L. (Hymenoptera: Apidae)) colonies. J Insect Sci. 2021:21(5):6. 10.1093/jisesa/ieab058 PMC8449538 34536080

[CIT0047] Jackson HM , Johnson SA, Morandin LA, Richardson LL, Guzman LM, M’Gonigle LK. Climate change winners and losers among North American bumblebees. Biol Lett. 2022:18(6):20210551. 10.1098/rsbl.2021.0551 35728617 PMC9213113

[CIT0048] Jacques A , Laurent M, Ribière-Chabert M, Saussac M, Bougeard S, Budge GE, Hendrikx P, Chauzat MP, De Graaf D, Estelle M, et al. A pan-European epidemiological study reveals honey bee colony survival depends on beekeeper education and disease control. PLoS One. 2017:12(3):e0172591. 28278255 10.1371/journal.pone.0172591 PMC5344352

[CIT0049] Janmaat AF , Winston ML. The influence of pollen storage area and *Varroa jacobsoni* Oedemans parasetism on temporal caste structure in honey bees (*Apis mellifera* L.). Insectes Soc. 2000:47(2):177–182. 10.1007/pl00001698

[CIT0050] Kammerer M , Goslee SC, Douglas MR, Tooker JF, Grozinger CM. Wild bees as winners and losers: relative impacts of landscape composition, quality, and climate. Glob Change Biol. 2021:27(6):1250–1265. 10.1111/gcb.15485 PMC7986353 33433964

[CIT0051] Koh I , Lonsdorf EV, Williams NM, Brittain C, Isaacs R, Gibbs J, Ricketts TH. Modeling the status, trends, and impacts of wild bee abundance in the United States. Proc Natl Acad Sci USA. 2016:113(1):140–145. 10.1073/pnas.1517685113 26699460 PMC4711882

[CIT0052] Kouchner C , Ferrus C, Blanchard S, Decourtye A, Basso B, Le Conte Y, Tchamitchian M. Bee farming system sustainability: an assessment framework in metropolitan France. Agric Syst. 2019:176:102653. 10.1016/j.agsy.2019.102653

[CIT0053] Kovac H , Crailsheim K. Lifespan of *Apis mellifera* Carnica Pollm. Infested by *Varroa jacobsoni* Oud. in relation to season and extent of infestation. J Apic Res. 1988:27(4):230–238.

[CIT0054] Kralj J , Brockmann A, Fuchs S, Tautz J. The parasitic mite *Varroa destructor* affects non-associative learning in honey bee foragers, *Apis mellifera* L. J Comp Physiol A Neuroethol Sens Neural Behav Physiol. 2007:193(3):363–370. 10.1007/s00359-006-0192-8 17123087

[CIT0055] Kralj J , Fuchs S. Parasitic *Varroa destructor* mites influence flight duration and homing ability of infested *Apis mellifera* foragers. Apidologie. 2006:37(5):577–587. 10.1051/apido:2006040

[CIT0056] Kulhanek K , Steinhauer N, Rennich K, Caron DM, Sagili RR, Pettis JS, Ellis JD, Wilson ME, Wilkes JT, Tarpy DR, et al. Encuesta nacional 2015–2016 sobre pérdidas anuales de colonias de la abeja de la miel manejada en los EE.UU. J Apic Res. 2017:56:328–340.

[CIT0057] Le Conte Y , Navajas M. Climate change: impact on honey bee populations and diseases. Rev Sci Tech Off Int Epiz. 2008:27(2):485–97, 499. 18819674

[CIT0058] Lindberg CM , Melathopoulos AP, Winston ML. Laboratory evaluation of Miticides to control *Varroa jacobsoni* (Acari: Varroidae), a honey bee (Hymenoptera: Apidae) parasite, apiculture and social insects. J Econ Entomol. 2000:93(2):189–198. 10.1603/0022-0493-93.2.189 10826162

[CIT0059] Lipper L , Thornton P, Campbell BM, Baedeker T, Braimoh A, Bwalya M, Caron P, Cattaneo A, Garrity D, Henry K, et al. Climate-smart agriculture for food security. Nat Clim Change. 2014:4(12):1068–1072. 10.1038/nclimate2437

[CIT0060] Lonsdorf E , Kremen C, Ricketts T, Winfree R, Williams N, Greenleaf S. Modelling pollination services across agricultural landscapes. Ann Bot. 2009:103(9):1589–1600. 10.1093/aob/mcp069 19324897 PMC2701767

[CIT0061] Malhi GS , Kaur M, Kaushik P. Impact of climate change on agriculture and its mitigation strategies: a review. Sustainability (Switzerland). 2021:13(3):1318. 10.3390/su13031318

[CIT0062] Martel AC , Zeggane S, Aurières C, Drajnudel P, Faucon JP, Aubert M. Acaricide residues in honey and wax after treatment of honey bee colonies with Apivar® or Asuntol® 50. Apidologie. 2007:38(6):534–544. 10.1051/apido:2007038

[CIT0063] Mattila HR , Harris JL, Otis GW. Timing of production of winter bees in honey bee (*Apis mellifera*) colonies. Insectes Soc. 2001:48(2):88–93. 10.1007/pl00001764

[CIT0064] Milani N. The resistance of *Varroa jacobsoni* Oud. to acaricides. Apidologie. 1999:30(2–3):229–234. 10.1051/apido:19990211

[CIT0065] Molineri A , Giacobino A, Pacini A, Bulacio Cagnolo N, Merke J, Orellano E, Bertozzi E, Zago L, Aignasse A, Pietronave H, et al. Environment and *Varroa destructor* management as determinant of colony losses in apiaries under temperate and subtropical climate. J Apic Res. 2018:57(4):551–564. 10.1080/00218839.2018.1475697

[CIT0066] Muijres FT , Van Dooremalen C, Lankheet M, Lugt H, De Vries LJ, Van Langevelde F. *Varroa destructor* infestation impairs the improvement of landing performance in foraging honeybees: landing in *Varroa*-infested honeybees. R Soc Open Sci. 2020:7(9):201222. 10.1098/rsos.201222 33047066 PMC7540786

[CIT0067] Nazzi F , Brown SP, Annoscia D, Del Piccolo F, Di Prisco G, Varricchio P, Della Vedova G, Cattonaro F, Caprio E, Pennacchio F. Synergistic parasite-pathogen interactions mediated by host immunity can drive the collapse of honeybee colonies. PLoS Pathog. 2012:8(6):e1002735. 10.1371/journal.ppat.1002735 22719246 PMC3375299

[CIT0068] Nürnberger F , Härtel S, Steffan-Dewenter I. Seasonal timing in honey bee colonies: phenology shifts affect honey stores and *Varroa* infestation levels. Oecologia. 2019:189(4):1121–1131. 10.1007/s00442-019-04377-1 30879141

[CIT0069] Ochungo P , Veldtman R, Abdel‐Rahman EM, Muli E, Ng’ang’a J, Tonnang HEZ, Landmann T. Fragmented landscapes affect honey bee colony strength at diverse spatial scales in agroecological landscapes in Kenya. Ecol Appl. 2022:32(1):e02483. 34674336 10.1002/eap.2483

[CIT0070] Overturf KA , Steinhauer N, Molinari R, Wilson ME, Watt AC, Cross RM, vanEngelsdorp D, Williams GR, Rogers SR. Winter weather predicts honey bee colony loss at the national scale. Ecol Indic. 2022:145:109709. 10.1016/j.ecolind.2022.109709

[CIT0071] Owens CD. The thermology of wintering honey bee colonies. Washington, D.C.: US Agricultural Research Service; 1971.

[CIT0072] PRISM Climate Group, O.S.U. https://prism.oregonstate.edu. 2014 (2 March 2023, date last accessed).

[CIT0073] Pusceddu M , Cini A, Alberti S, Salaris E, Theodorou P, Floris I, Satta A. Honey bees increase social distancing when facing the ectoparasite *Varroa destructor*. Sci Adv. 2021:7(44):abj1398. 10.1126/sciadv.abj1398 PMC8555907 34714677

[CIT0074] Qadir ZA , Idrees A, Mahmood R, Sarwar G, Bakar MA, Ahmad S, Raza MM, Li J. Effectiveness of different soft acaricides against honey bee ectoparasitic mite *Varroa destructor* (Acari: Varroidae). Insects. 2021:12(11):1032. 10.3390/insects12111032 34821832 PMC8624935

[CIT0075] Quinlan GM , Isaacs R, Otto CRV, Smart AH, Milbrath MO. Association of excessive precipitation and agricultural land use with honey bee colony performance. Landsc Ecol. 2023:38(6):1555–1569. 10.1007/s10980-023-01638-6

[CIT0076] Quinlan GM , Sponsler D, Gaines-Day HR, McMinn-Sauder HBG, Otto CRV, Smart AH, Colin T, Gratton C, Isaacs R, Johnson R, et al. Grassy–herbaceous land moderates regional climate effects on honey bee colonies in the Northcentral US. Environ Res Lett. 2022:17(6):064036.

[CIT0110] Quinlan, G. M, Grozinger C. M. Elsevier BV. 2023:64:289–345

[CIT0077] R Core Team. R: a language and environment for statistical computing. Vienna (Austria): R Foundation for Statistical Computing; 2022. https://www.R-project.org/.

[CIT0078] Rinkevich FD. Detection of amitraz resistance and reduced treatment efficacy in the *Varroa* mite, *Varroa destructor*, within commercial beekeeping operations. PLoS One. 2020:15(1):e0227264. 10.1371/journal.pone.0227264 31951619 PMC6968863

[CIT0079] Rosenkranz P , Aumeier P, Ziegelmann B. Biology and control of *Varroa destructor*. J Invertebr Pathol. 2010:103(Suppl 1):S96–119. 10.1016/j.jip.2009.07.016 19909970

[CIT0080] Roth MA , Wilson JM, Tignor KR, Gross AD. Biology and management of *Varroa destructor* (Mesostigmata: Varroidae) in *Apis mellifera* (Hymenoptera: Apidae) colonies. J Integr Pest Manag. 2020:11(1):1.

[CIT0081] Sabahi Q , Morfin N, Emsen B, Gashout HA, Kelly PG, Otto S, Merrill AR, Guzman-Novoa E. Evaluation of dry and wet formulations of oxalic acid, thymol, and oregano oil for *Varroa* mite (Acari: Varroidae) control in honey bee (Hymenoptera: Apidae) colonies. J Econ Entomol. 2020:113(6):2588–2594. 10.1093/jee/toaa218 33001171

[CIT0082] Shoemaker KT , Heffelfinger LJ, Jackson NJ, Blum ME, Wasley T, Stewart KM. A machine-learning approach for extending classical wildlife resource selection analyses. Ecol Evol. 2018:8(6):3556–3569. 10.1002/ece3.3936 29607046 PMC5869366

[CIT0083] Smoliński S , Langowska A, Glazaczow A. Raised seasonal temperatures reinforce autumn *Varroa destructor* infestation in honey bee colonies. Sci Rep. 2021:11(1):22256. 10.1038/s41598-021-01369-1 34782664 PMC8593171

[CIT0084] Southwick EE , Heldmaier G. Temperature control in honey bee colonies. Am Inst Biol Sci. 1987:37(6):395–399.

[CIT0085] St Clair AL , St Clair AL, Zhang G, Dolezal AG, O’Neal ME, Toth AL, Toth AL. Diversified farming in a monoculture landscape: effects on honey bee health and wild bee communities. Environ Entomol. 2020:49(3):753–764. 32249293 10.1093/ee/nvaa031 PMC7371362

[CIT0086] Strange JP , Sheppard WS. Optimum timing of miticide applications for control of *Varroa destructor* (Acari: Varroidae) in *Apis mellifera* (Hymenoptera: Apidae) in Washington State, USA. J Econ Entomol. 2001:94(6):1324–1331. 10.1603/0022-0493-94.6.1324 11777032

[CIT0087] Strobl C , Boulesteix AL, Zeileis A, Hothorn T. Bias in random forest variable importance measures: illustrations, sources and a solution. BMC Bioinf. 2007:8:25. 10.1186/1471-2105-8-25 PMC1796903 17254353

[CIT0088] Switanek M , Crailsheim K, Truhetz H, Brodschneider R. Modelling seasonal effects of temperature and precipitation on honey bee winter mortality in a temperate climate. Sci Total Environ. 2017:579:1581–1587. 10.1016/j.scitotenv.2016.11.178 27916302

[CIT0089] Szentgyörgyi H , Czekońska K, Tofilski A. Honey bees are larger and live longer after developing at low temperature. J Therm Biol. 2018:78:219–226. 10.1016/j.jtherbio.2018.09.007 30509639

[CIT0090] Underwood RM , Lawrence BL, Turley NE, Cambron-Kopco LD, Kietzman PM, Traver BE, López-Uribe MM. A longitudinal experiment demonstrates that honey bee colonies managed organically are as healthy and productive as those managed conventionally. Sci Rep. 2023:13(1):6072. 10.1038/s41598-023-32824-w 37055462 PMC10100614

[CIT0091] Underwood RM , Traver BE, López-Uribe MM. Beekeeping management practices are associated with operation size and beekeepers’ philosophy towards in-hive chemicals. Insects. 2019:10(1):10. 10.3390/insects10010010 30626023 PMC6359672

[CIT0108] Underwood RM , López-Uribe MM. Methods to control Varroa mites: An integrated pest management approach. Pennsylvania State Extension. 2019. https://extension.psu.edu/methods-to-control-varroa-mites-an-integrated-pest-management-approach

[CIT0092] USDA NASS. USDA National Agricultural Statistics Service Cropland Data Layer. 2021. https://www.nass.usda.gov/Research_and_Science/Cropland/Release/index.php. (2 March 2023, date last accessed).

[CIT0093] van Dooremalen C , Gerritsen L, Cornelissen B, van der Steen JJM, Langevelde F. van, Blacquière T. Winter survival of individual honey bees and honey bee colonies depends on level of *Varroa destructor* infestation. PLoS One. 2012:7(4):e36285. 22558421 10.1371/journal.pone.0036285 PMC3338694

[CIT0094] Van Esch L , De Kok JL, Janssen L, Buelens B, De Smet L, de Graaf DC, Engelen G. Multivariate landscape analysis of honey bee winter mortality in Wallonia, Belgium. Environ Model Assess. 2020:25(3):441–452. 10.1007/s10666-019-09682-w

[CIT0095] Vandervalk LP , Nasr ME, Dosdall LM. New miticides for integrated pest management of *Varroa destructor* (Acari: Varroidae) in honey bee colonies on the Canadian prairies. J Econ Entomol. 2014:107(6):2030–2036. 10.1603/EC14048 26470066

[CIT0096] Visscher PK , Seeley TD. Foraging strategy of honeybee colonies in a temperate deciduous forest. Ecology. 1982:63(6):1790. 10.2307/1940121

[CIT0097] Wickham H. ggplot2: elegant graphics for data analysis. New York (NY): Springer-Verlag; 2016.

[CIT0098] Winston ML. The biology of the honey bee. Cambridge, Massachusetts: Harvard University Press; 1987.

[CIT0099] Wong TT , Yeh PY. Reliable accuracy estimates from k-fold cross validation. IEEE Trans Knowl Data Eng. 2020:32(8):1586–1594. 10.1109/tkde.2019.2912815

[CIT0100] Wood TJ , Michez D, Paxton RJ, Drossart M, Neumann P, Gérard M, Vanderplanck M, Barraud A, Martinet B, Leclercq N, et al. Managed honey bees as a radar for wild bee decline? Apidologie. 2020:51(6):1100–1116. 10.1007/s13592-020-00788-9

[CIT0101] Woodcock BA , Bullock JM, Shore RF, Heard MS, Pereira MG, Redhead J, Ridding L, Dean H, Sleep D, Henrys P, et al. Country-specific effects of neonicotinoid pesticides on honey bees and wild bees. Science. 2017:356(6345):1393–1395. 10.1126/science.aaa1190 28663502

[CIT0102] Wright MN , Ziegler A. ranger: a fast implementation of random forests for high dimensional data in *C++* and *R*. J Stat Softw. 2017:77(1):1–17.

[CIT0103] Yang H , Shi J, Liao C, Yan W, Wu X. *Varroa destructor* mite infestations in capped brood cells of honeybee workers affect emergence development and adult foraging ability. Curr Zool. 2021:67(5):569–571. 10.1093/cz/zoab002 34616956 PMC8489162

[CIT0104] Zaobidna EA , Zółtowska K, Łopieńska-Biernat E. *Varroa destructor* induces changes in the expression of immunity-related genes during the development of *Apis mellifera* worker and drone broods. Acta Parasitol. 2017:62(4):779–789. 10.1515/ap-2017-0094 29035869

[CIT0105] Zilberman D , Goetz R, Garrido A. Climate smart agriculture: building resilience to climate change. New York: Springer; 2018.

