# Supplementary material for: Effective pest management approaches can mitigate honey bee (Apis mellifera) colony winter loss across a range of weather conditions in small-scale, stationary apiaries
Source: J Insect Sci. 2024 May 28;24(3):15. doi: 10.1093/jisesa/ieae043 (PMC11132132; doi:10.1093/jisesa/ieae043)
Supplement: ieae043_suppl_Supplementary_Material [file ieae043_suppl_supplementary_material.docx]

**Supplementary Materials**

Effective pest management approaches can mitigate honey bee colony winter loss (*Apis mellifera*) across a range of weather conditions in small-scale, stationary apiaries

**Variable Importance Results**


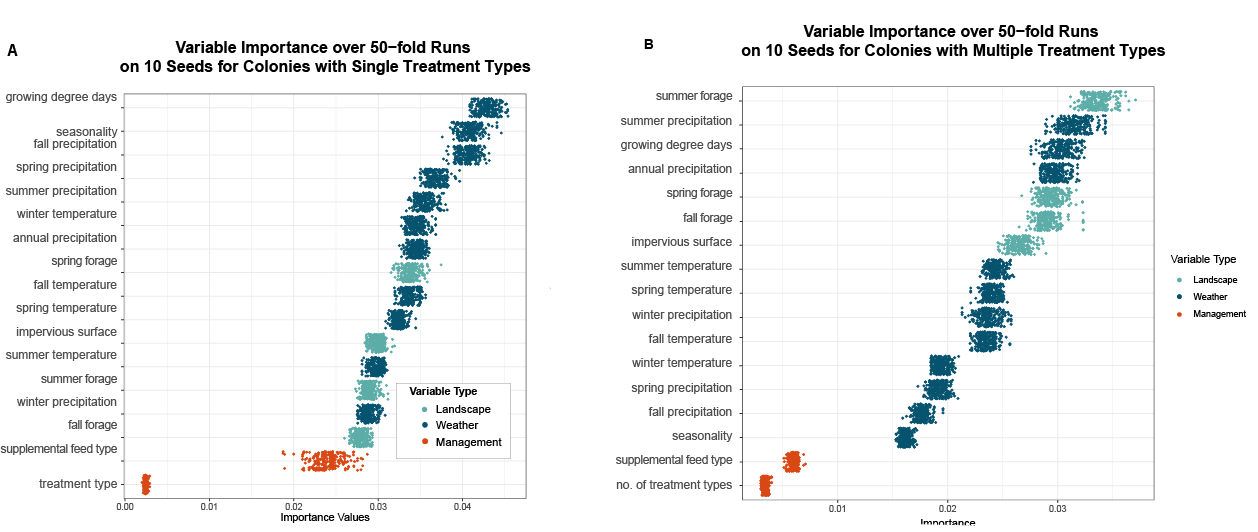


**Figure S1**. Variable importance from a 50-fold cross validation on each of 10 random seeds to determine the mean variable importance for the single-treatment type model (**A**) of colony survival and the multiple-treatment type model (**B**) of survival. Variable type is represented by color: light blue is landscape, dark blue is weather, and orange is management variables. Variance in importance between model runs was low.

**Beekeeping Management and Colony Survival Results**


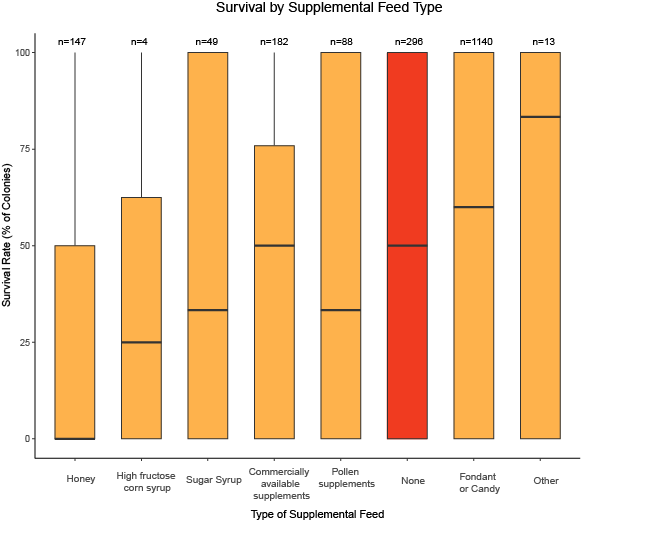


**Figure S2.** Box and whisker plots showing the distribution of winter colony survival for supplemental feed types for colonies fed only one type of supplemental feed. Boxes represent the interquartile range (IQR), the horizontal line represents the median and the whiskers represent minimum and maximum values 1.5 times the IQR. There was an overall significant difference in survival between types of supplemental feed (χ2= 69.05, p= 2.295e-12). Fondant or sugar candy significantly higher mean survival than unfed colonies. Colonies fed honey had significantly lower survival than all other groups. The remaining pairwise relationships were not significant.

**Results of Supplemental Feed Analysis**

Colonies fed honey had significantly lower survival (mean= 28.6%, sd=37.2 ) than those that were unfed (mean= 48.7%, sd=41.5, p= 3.638e-05), those fed sugar syrup (mean= 41.9%, sd=42.3, p= 0.0062), those fed commercially available supplements (mean = 43.5%, sd= 38.4, p= 0.003), pollen supplements (mean= 46.4%, sd=44.7 , p = 0.024 ), fondant or candy (mean= 55.7%, sd=40.3 , p = 2.532e-13), and other (mean = 58.44%, sd=43.9, , p = 0.0297). There was no difference in survival between those fed honey and those fed high fructose corn syrup (mean= 37.5%, 47.9, p =0.7551). High fructose corn syrup had small sample size (n=4) and did not have significantly different survival from any other supplemental feed. Colonies fed fondant or candy had significantly higher survival than those that were not fed (p= 0.04) and higher survival than those fed sugar syrup (p= 7.931e-05).

**Results** **of Treatment Type Analysis- Single Treatment Type**

Apiaries treated with formic acid had higher mean survival (mean= 50.2%, sd=40.5) than untreated colonies (mean= 31.1%, sd= 39.8 , p= 0.0001) as did those treated with thymol (mean= 50.5% , sd = 41, p< 2.2e-16), amitraz (mean = 54.9%, sd =40.7 , p= 1.123e-15), and oxalic acid (mean= 55.4%, sd = 38.3, p < 2.2e-16). Apiaries treated with hop beta acids (mean=33.1%, sd=36.7) did not have significantly higher survival than untreated (p= 0.2604). Those treated with hop beta acids had lower survival than those treated with formic acid (p= 0.0024), thymol (p= 0.022), amitraz (p=0.0003), and oxalic acid (p= 2.396e-05). Apiaries treated with oxalic acid had significantly higher survival than those treated with formic (p = 0.0242). There was no significant differences between colonies treated with formic and thymol (p = 0.9389), those treated with formic and those treated with amitraz (p= 0.1207), thymol and amitraz (p=0.4306) and thymol and oxalic acid (p = 0.3541).

**Data**

**Table S1. Anonymized Survey Responses with Weather Variables**

| **site** | **year** | **mean_imperv** | **mite.monitor.boolean** | **mite.monitor.type** | **mite.treatment.boolean** | **mite.treatment.type** | **supp.winter.feed.boolean** | **supp.winter.feed.type** | **winter.feed.type** |
| --- | --- | --- | --- | --- | --- | --- | --- | --- | --- |
| 1 | 2017 | 1.567923 | Yes | Drone brood inspection | Yes | Oxalic Acid | Yes | Sugar syrup, Pollen substitute | none |
| 2 | 2017 | 1.567923 | Yes | Drone brood inspection | Yes | Oxalic Acid | Yes | Sugar syrup, Pollen substitute | none |
| 3 | 2017 | 1.567923 | Yes | Drone brood inspection | Yes | Oxalic Acid | Yes | Sugar syrup, Pollen substitute | none |
| 4 | 2017 | 1.567923 | Yes | Drone brood inspection | Yes | Oxalic Acid | Yes | Sugar syrup, Pollen substitute | none |
| 5 | 2017 | 1.567923 | Yes | Drone brood inspection | Yes | Oxalic Acid | Yes | Sugar syrup, Pollen substitute | none |
| 6 | 2017 | 2.492022 | Yes | treatment | Yes | Oxalic Acid | Yes | Commercially available supplements | none |
| 7 | 2017 | 2.492022 | Yes | treatment | Yes | Oxalic Acid | Yes | Commercially available supplements | none |
| 8 | 2017 | 8.095434 | Yes | Sugar roll | Yes | Apivar (Amitraz) | Yes | Dry sugar | sugar only |
| 9 | 2017 | 8.095434 | Yes | Sugar roll | Yes | Apivar (Amitraz) | Yes | Dry sugar | sugar only |
| 10 | 2017 | 8.095434 | Yes | Sugar roll | Yes | Apivar (Amitraz) | Yes | Dry sugar | sugar only |
| 11 | 2017 | 0.630588 | Yes | Sugar roll | Yes | Oxalic Acid | Yes | Fondant or sugar candy | sugar only |
| 12 | 2017 | 0.630588 | Yes | Sugar roll | Yes | Oxalic Acid | Yes | Fondant or sugar candy | sugar only |
| 13 | 2017 | 0.630588 | Yes | Sugar roll | Yes | Oxalic Acid | Yes | Fondant or sugar candy | sugar only |
| 14 | 2017 | 0.630588 | Yes | Sugar roll | Yes | Oxalic Acid | Yes | Fondant or sugar candy | sugar only |
| 15 | 2017 | 0.630588 | Yes | Sugar roll | Yes | Oxalic Acid | Yes | Fondant or sugar candy | sugar only |
| 16 | 2017 | 0.630588 | Yes | Sugar roll | Yes | Oxalic Acid | Yes | Fondant or sugar candy | sugar only |
| 17 | 2017 | 0.630588 | Yes | Sugar roll | Yes | Oxalic Acid | Yes | Fondant or sugar candy | sugar only |
| 18 | 2017 | 0.630588 | Yes | Sugar roll | Yes | Oxalic Acid | Yes | Fondant or sugar candy | sugar only |
| 19 | 2017 | 0.630588 | Yes | Sugar roll | Yes | Oxalic Acid | Yes | Fondant or sugar candy | sugar only |
| 20 | 2017 | 0.630588 | Yes | Sugar roll | Yes | Oxalic Acid | Yes | Fondant or sugar candy | sugar only |
| 21 | 2017 | 0.630588 | Yes | Sugar roll | Yes | Oxalic Acid | Yes | Fondant or sugar candy | sugar only |
| 22 | 2017 | 0.630588 | Yes | Sugar roll | Yes | Oxalic Acid | Yes | Fondant or sugar candy | sugar only |
| 23 | 2017 | 0.630588 | Yes | Sugar roll | Yes | Oxalic Acid | Yes | Fondant or sugar candy | sugar only |
| 24 | 2017 | 0.630588 | Yes | Sugar roll | Yes | Oxalic Acid | Yes | Fondant or sugar candy | sugar only |
| 25 | 2017 | 0.630588 | Yes | Sugar roll | Yes | Oxalic Acid | Yes | Fondant or sugar candy | sugar only |
| 26 | 2017 | 0.630588 | Yes | Sugar roll | Yes | Oxalic Acid | Yes | Fondant or sugar candy | sugar only |
| 27 | 2017 | 25.53753 | No | NA | Yes | Formic Acid (Mite Away Quick Strips) | Yes | Fondant or sugar candy | sugar only |
| 28 | 2017 | 25.53753 | No | NA | Yes | Formic Acid (Mite Away Quick Strips) | Yes | Fondant or sugar candy | sugar only |
| 29 | 2017 | 9.911617 | No | NA | Yes | Oxalic Acid | Yes | Fondant or sugar candy, Commercially available supplements | none |
| 30 | 2017 | 9.911617 | No | NA | Yes | Oxalic Acid | Yes | Fondant or sugar candy, Commercially available supplements | none |
| 31 | 2017 | 9.911617 | No | NA | Yes | Oxalic Acid | Yes | Fondant or sugar candy, Commercially available supplements | none |
| 32 | 2017 | 9.911617 | No | NA | Yes | Oxalic Acid | Yes | Fondant or sugar candy, Commercially available supplements | none |
| 33 | 2017 | 9.911617 | No | NA | Yes | Oxalic Acid | Yes | Fondant or sugar candy, Commercially available supplements | none |
| 34 | 2017 | 9.911617 | No | NA | Yes | Oxalic Acid | Yes | Fondant or sugar candy, Commercially available supplements | none |
| 35 | 2017 | 9.911617 | No | NA | Yes | Oxalic Acid | Yes | Fondant or sugar candy, Commercially available supplements | none |
| 36 | 2017 | 3.302294 | Yes | Sugar roll | Yes | Oxalic Acid | Yes | Fondant or sugar candy, Sugar syrup, Commercially available supplements | none |
| 37 | 2017 | 3.302294 | Yes | Sugar roll | Yes | Oxalic Acid | Yes | Fondant or sugar candy, Sugar syrup, Commercially available supplements | none |
| 38 | 2017 | 3.302294 | Yes | Sugar roll | Yes | Oxalic Acid | Yes | Fondant or sugar candy, Sugar syrup, Commercially available supplements | none |
| 39 | 2017 | 3.302294 | Yes | Sugar roll | Yes | Oxalic Acid | Yes | Fondant or sugar candy, Sugar syrup, Commercially available supplements | none |
| 40 | 2017 | 3.302294 | Yes | Sugar roll | Yes | Oxalic Acid | Yes | Fondant or sugar candy, Sugar syrup, Commercially available supplements | none |
| 41 | 2017 | 3.302294 | Yes | Sugar roll | Yes | Oxalic Acid | Yes | Fondant or sugar candy, Sugar syrup, Commercially available supplements | none |
| 42 | 2017 | 1.530424 | Yes | Drone brood inspection, droone comb | Yes | Apivar (Amitraz) | Yes | Honey from your own stock, Commercially available supplements | none |
| 43 | 2017 | 1.530424 | Yes | Drone brood inspection, droone comb | Yes | Apivar (Amitraz) | Yes | Honey from your own stock, Commercially available supplements | none |
| 44 | 2017 | 1.530424 | Yes | Drone brood inspection, droone comb | Yes | Apivar (Amitraz) | Yes | Honey from your own stock, Commercially available supplements | none |
| 45 | 2017 | 1.530424 | Yes | Drone brood inspection, droone comb | Yes | Apivar (Amitraz) | Yes | Honey from your own stock, Commercially available supplements | none |
| 46 | 2017 | 1.530424 | Yes | Drone brood inspection, droone comb | Yes | Apivar (Amitraz) | Yes | Honey from your own stock, Commercially available supplements | none |
| 47 | 2017 | 1.530424 | Yes | Drone brood inspection, droone comb | Yes | Apivar (Amitraz) | Yes | Honey from your own stock, Commercially available supplements | none |
| 48 | 2017 | 1.530424 | Yes | Drone brood inspection, droone comb | Yes | Apivar (Amitraz) | Yes | Honey from your own stock, Commercially available supplements | none |
| 49 | 2017 | 1.530424 | Yes | Drone brood inspection, droone comb | Yes | Apivar (Amitraz) | Yes | Honey from your own stock, Commercially available supplements | none |
|  |  |  |  |  |  |  |  |  |  |
| 50 | 2017 | 1.530424 | Yes | Drone brood inspection, droone comb | Yes | Apivar (Amitraz) | Yes | Honey from your own stock, Commercially available supplements | none |
| 51 | 2017 | 1.530424 | Yes | Drone brood inspection, droone comb | Yes | Apivar (Amitraz) | Yes | Honey from your own stock, Commercially available supplements | none |
| 52 | 2017 | 1.530424 | Yes | Drone brood inspection, droone comb | Yes | Apivar (Amitraz) | Yes | Honey from your own stock, Commercially available supplements | none |
| 53 | 2017 | 1.530424 | Yes | Drone brood inspection, droone comb | Yes | Apivar (Amitraz) | Yes | Honey from your own stock, Commercially available supplements | none |
| 54 | 2017 | 4.544047 | Yes | Sugar roll | Yes | Formic Acid (Mite Away Quick Strips) | Yes | Fondant or sugar candy | sugar only |
| 55 | 2017 | 4.544047 | Yes | Sugar roll | Yes | Formic Acid (Mite Away Quick Strips) | Yes | Fondant or sugar candy | sugar only |
| 56 | 2017 | 4.544047 | Yes | Sugar roll | Yes | Formic Acid (Mite Away Quick Strips) | Yes | Fondant or sugar candy | sugar only |
| 57 | 2017 | 4.544047 | Yes | Sugar roll | Yes | Formic Acid (Mite Away Quick Strips) | Yes | Fondant or sugar candy | sugar only |
| 58 | 2017 | 8.639937 | Yes | Sugar roll | Yes | Apivar (Amitraz) | Yes | Fondant or sugar candy | sugar only |
| 59 | 2017 | 8.639937 | Yes | Sugar roll | Yes | Apivar (Amitraz) | Yes | Fondant or sugar candy | sugar only |
| 60 | 2017 | 0.823756 | Yes | 48 hr drop, Sugar roll | Yes | Formic Acid (Mite Away Quick Strips) | Yes | Fondant or sugar candy, Dry sugar | none |
| 61 | 2017 | 0.823756 | Yes | 48 hr drop, Sugar roll | Yes | Formic Acid (Mite Away Quick Strips) | Yes | Fondant or sugar candy, Dry sugar | none |
| 62 | 2017 | 0.823756 | Yes | 48 hr drop, Sugar roll | Yes | Formic Acid (Mite Away Quick Strips) | Yes | Fondant or sugar candy, Dry sugar | none |
| 63 | 2017 | 1.208234 | Yes | Sugar roll | Yes | Oxalic Acid | Yes | Fondant or sugar candy, Honey from your own stock | none |
| 64 | 2017 | 1.208234 | Yes | Sugar roll | Yes | Oxalic Acid | Yes | Fondant or sugar candy, Honey from your own stock | none |
| 65 | 2017 | 1.208234 | Yes | Sugar roll | Yes | Oxalic Acid | Yes | Fondant or sugar candy, Honey from your own stock | none |
| 66 | 2017 | 1.208234 | Yes | Sugar roll | Yes | Oxalic Acid | Yes | Fondant or sugar candy, Honey from your own stock | none |
| 67 | 2017 | 1.208234 | Yes | Sugar roll | Yes | Oxalic Acid | Yes | Fondant or sugar candy, Honey from your own stock | none |
| 68 | 2017 | 1.208234 | Yes | Sugar roll | Yes | Oxalic Acid | Yes | Fondant or sugar candy, Honey from your own stock | none |
| 69 | 2017 | 1.208234 | Yes | Sugar roll | Yes | Oxalic Acid | Yes | Fondant or sugar candy, Honey from your own stock | none |
| 70 | 2017 | 1.208234 | Yes | Sugar roll | Yes | Oxalic Acid | Yes | Fondant or sugar candy, Honey from your own stock | none |
| 71 | 2017 | 1.208234 | Yes | Sugar roll | Yes | Oxalic Acid | Yes | Fondant or sugar candy, Honey from your own stock | none |
| 72 | 2017 | 1.208234 | Yes | Sugar roll | Yes | Oxalic Acid | Yes | Fondant or sugar candy, Honey from your own stock | none |
| 73 | 2017 | 1.208234 | Yes | Sugar roll | Yes | Oxalic Acid | Yes | Fondant or sugar candy, Honey from your own stock | none |
| 74 | 2017 | 1.208234 | Yes | Sugar roll | Yes | Oxalic Acid | Yes | Fondant or sugar candy, Honey from your own stock | none |
| 75 | 2017 | 1.208234 | Yes | Sugar roll | Yes | Oxalic Acid | Yes | Fondant or sugar candy, Honey from your own stock | none |
| 76 | 2017 | 1.208234 | Yes | Sugar roll | Yes | Oxalic Acid | Yes | Fondant or sugar candy, Honey from your own stock | none |
| 77 | 2017 | 1.208234 | Yes | Sugar roll | Yes | Oxalic Acid | Yes | Fondant or sugar candy, Honey from your own stock | none |
| 78 | 2017 | 1.208234 | Yes | Sugar roll | Yes | Oxalic Acid | Yes | Fondant or sugar candy, Honey from your own stock | none |
| 79 | 2017 | 1.208234 | Yes | Sugar roll | Yes | Oxalic Acid | Yes | Fondant or sugar candy, Honey from your own stock | none |
| 80 | 2017 | 1.208234 | Yes | Sugar roll | Yes | Oxalic Acid | Yes | Fondant or sugar candy, Honey from your own stock | none |
| 81 | 2017 | 1.208234 | Yes | Sugar roll | Yes | Oxalic Acid | Yes | Fondant or sugar candy, Honey from your own stock | none |
| 82 | 2017 | 1.208234 | Yes | Sugar roll | Yes | Oxalic Acid | Yes | Fondant or sugar candy, Honey from your own stock | none |
| 83 | 2017 | 4.456251 | Yes | 48 hr drop | Yes | Formic Acid (Mite Away Quick Strips) | Yes | Fondant or sugar candy, Honey from your own stock | none |
| 84 | 2017 | 4.456251 | Yes | 48 hr drop | Yes | Formic Acid (Mite Away Quick Strips) | Yes | Fondant or sugar candy, Honey from your own stock | none |
| 85 | 2017 | 4.456251 | Yes | 48 hr drop | Yes | Formic Acid (Mite Away Quick Strips) | Yes | Fondant or sugar candy, Honey from your own stock | none |
| 86 | 2017 | 4.456251 | Yes | 48 hr drop | Yes | Formic Acid (Mite Away Quick Strips) | Yes | Fondant or sugar candy, Honey from your own stock | none |
| 87 | 2017 | 4.456251 | Yes | 48 hr drop | Yes | Formic Acid (Mite Away Quick Strips) | Yes | Fondant or sugar candy, Honey from your own stock | none |
| 88 | 2017 | 5.975504 | No | NA | Yes | Apivar (Amitraz) | Yes | Dry sugar | sugar only |
| 89 | 2017 | 5.975504 | No | NA | Yes | Apivar (Amitraz) | Yes | Dry sugar | sugar only |
| 90 | 2017 | 5.975504 | No | NA | Yes | Apivar (Amitraz) | Yes | Dry sugar | sugar only |
| 91 | 2017 | 5.975504 | No | NA | Yes | Apivar (Amitraz) | Yes | Dry sugar | sugar only |
| 92 | 2017 | 5.975504 | No | NA | Yes | Apivar (Amitraz) | Yes | Dry sugar | sugar only |
| 93 | 2017 | 5.975504 | No | NA | Yes | Apivar (Amitraz) | Yes | Dry sugar | sugar only |
| 94 | 2017 | 5.975504 | No | NA | Yes | Apivar (Amitraz) | Yes | Dry sugar | sugar only |
| 95 | 2017 | 4.288306 | Yes | Drone brood inspection | Yes | Formic Acid (Mite Away Quick Strips) | Yes | Dry sugar, Pollen substitute | none |
| 96 | 2017 | 4.288306 | Yes | Drone brood inspection | Yes | Formic Acid (Mite Away Quick Strips) | Yes | Dry sugar, Pollen substitute | none |
| 97 | 2017 | 4.288306 | Yes | Drone brood inspection | Yes | Formic Acid (Mite Away Quick Strips) | Yes | Dry sugar, Pollen substitute | none |
| 98 | 2017 | 4.288306 | Yes | Drone brood inspection | Yes | Formic Acid (Mite Away Quick Strips) | Yes | Dry sugar, Pollen substitute | none |
| 99 | 2017 | 4.288306 | Yes | Drone brood inspection | Yes | Formic Acid (Mite Away Quick Strips) | Yes | Dry sugar, Pollen substitute | none |
| 100 | 2017 | 4.288306 | Yes | Drone brood inspection | Yes | Formic Acid (Mite Away Quick Strips) | Yes | Dry sugar, Pollen substitute | none |
| 101 | 2017 | 4.288306 | Yes | Drone brood inspection | Yes | Formic Acid (Mite Away Quick Strips) | Yes | Dry sugar, Pollen substitute | none |
| 102 | 2017 | 4.288306 | Yes | Drone brood inspection | Yes | Formic Acid (Mite Away Quick Strips) | Yes | Dry sugar, Pollen substitute | none |
| 103 | 2017 | 4.288306 | Yes | Drone brood inspection | Yes | Formic Acid (Mite Away Quick Strips) | Yes | Dry sugar, Pollen substitute | none |
| 104 | 2017 | 4.288306 | Yes | Drone brood inspection | Yes | Formic Acid (Mite Away Quick Strips) | Yes | Dry sugar, Pollen substitute | none |
| 105 | 2017 | 4.288306 | Yes | Drone brood inspection | Yes | Formic Acid (Mite Away Quick Strips) | Yes | Dry sugar, Pollen substitute | none |
| 106 | 2017 | 36.75384 | Yes | 48 hr drop | Yes | hopguard | Yes | Fondant or sugar candy, Pollen substitute | none |
| 107 | 2017 | 36.75384 | Yes | 48 hr drop | Yes | hopguard | Yes | Fondant or sugar candy, Pollen substitute | none |
| 108 | 2017 | 36.75384 | Yes | 48 hr drop | Yes | hopguard | Yes | Fondant or sugar candy, Pollen substitute | none |
| 109 | 2017 | 5.812994 | Yes | Drone brood inspection | Yes | Oxalic Acid | Yes | Sugar syrup | none |
| 110 | 2017 | 20.07676 | Yes | 48 hr drop | Yes | Formic Acid (Mite Away Quick Strips) | Yes | Fondant or sugar candy | sugar only |
| 111 | 2017 | 25.87177 | No | Visual Inspection | Yes | Formic Acid (Mite Away Quick Strips) | Yes | Fondant or sugar candy, Sugar syrup | none |
| 112 | 2017 | 25.87177 | No | Visual Inspection | Yes | Formic Acid (Mite Away Quick Strips) | Yes | Fondant or sugar candy, Sugar syrup | none |
| 113 | 2017 | 6.043198 | Yes | Drone brood inspection | Yes | Formic Acid (Mite Away Quick Strips) | Yes | Fondant or sugar candy | sugar only |
| 114 | 2017 | 6.043198 | Yes | Drone brood inspection | Yes | Formic Acid (Mite Away Quick Strips) | Yes | Fondant or sugar candy | sugar only |
| 115 | 2017 | 3.754754 | No | NA | Yes | Oxalic Acid | Yes | Fondant or sugar candy, pollen patties from Brushy Mountain | none |
| 116 | 2017 | 3.754754 | No | NA | Yes | Oxalic Acid | Yes | Fondant or sugar candy, pollen patties from Brushy Mountain | none |
| 117 | 2017 | 3.754754 | No | NA | Yes | Oxalic Acid | Yes | Fondant or sugar candy, pollen patties from Brushy Mountain | none |
| 118 | 2017 | 0.524944 | Yes | 48 hr drop, Drone brood inspection | Yes | Formic Acid (Mite Away Quick Strips) | Yes | Fondant or sugar candy, Pollen substitute, Honey from your own stock | none |
| 119 | 2017 | 0.524944 | Yes | 48 hr drop, Drone brood inspection | Yes | Formic Acid (Mite Away Quick Strips) | Yes | Fondant or sugar candy, Pollen substitute, Honey from your own stock | none |
| 120 | 2017 | 0.524944 | Yes | 48 hr drop, Drone brood inspection | Yes | Formic Acid (Mite Away Quick Strips) | Yes | Fondant or sugar candy, Pollen substitute, Honey from your own stock | none |
| 121 | 2017 | 0.524944 | Yes | 48 hr drop, Drone brood inspection | Yes | Formic Acid (Mite Away Quick Strips) | Yes | Fondant or sugar candy, Pollen substitute, Honey from your own stock | none |
| 122 | 2017 | 0.524944 | Yes | 48 hr drop, Drone brood inspection | Yes | Formic Acid (Mite Away Quick Strips) | Yes | Fondant or sugar candy, Pollen substitute, Honey from your own stock | none |
| 123 | 2017 | 0.524944 | Yes | 48 hr drop, Drone brood inspection | Yes | Formic Acid (Mite Away Quick Strips) | Yes | Fondant or sugar candy, Pollen substitute, Honey from your own stock | none |
| 124 | 2017 | 0.524944 | Yes | 48 hr drop, Drone brood inspection | Yes | Formic Acid (Mite Away Quick Strips) | Yes | Fondant or sugar candy, Pollen substitute, Honey from your own stock | none |
| 125 | 2017 | 0.524944 | Yes | 48 hr drop, Drone brood inspection | Yes | Formic Acid (Mite Away Quick Strips) | Yes | Fondant or sugar candy, Pollen substitute, Honey from your own stock | none |
| 126 | 2017 | 0.524944 | Yes | 48 hr drop, Drone brood inspection | Yes | Formic Acid (Mite Away Quick Strips) | Yes | Fondant or sugar candy, Pollen substitute, Honey from your own stock | none |
| 127 | 2017 | 0.524944 | Yes | 48 hr drop, Drone brood inspection | Yes | Formic Acid (Mite Away Quick Strips) | Yes | Fondant or sugar candy, Pollen substitute, Honey from your own stock | none |
| 128 | 2017 | 4.320623 | Yes | Sugar roll | Yes | Formic Acid (Mite Away Quick Strips) | Yes | Dry sugar, Pollen substitute | none |
| 129 | 2017 | 6.26582 | No | NA | Yes | Apivar (Amitraz) | Yes | Fondant or sugar candy | sugar only |
| 130 | 2017 | 1.200528 | Yes | Sugar roll | Yes | Oxalic Acid | Yes | Pollen substitute, Honey from your own stock | none |
| 131 | 2017 | 1.200528 | Yes | Sugar roll | Yes | Oxalic Acid | Yes | Pollen substitute, Honey from your own stock | none |
| 132 | 2017 | 1.200528 | Yes | Sugar roll | Yes | Oxalic Acid | Yes | Pollen substitute, Honey from your own stock | none |
| 133 | 2017 | 1.200528 | Yes | Sugar roll | Yes | Oxalic Acid | Yes | Pollen substitute, Honey from your own stock | none |
| 134 | 2017 | 1.200528 | Yes | Sugar roll | Yes | Oxalic Acid | Yes | Pollen substitute, Honey from your own stock | none |
| 135 | 2017 | 1.200528 | Yes | Sugar roll | Yes | Oxalic Acid | Yes | Pollen substitute, Honey from your own stock | none |
| 136 | 2017 | 1.200528 | Yes | Sugar roll | Yes | Oxalic Acid | Yes | Pollen substitute, Honey from your own stock | none |
| 137 | 2017 | 1.200528 | Yes | Sugar roll | Yes | Oxalic Acid | Yes | Pollen substitute, Honey from your own stock | none |
| 138 | 2017 | 1.200528 | Yes | Sugar roll | Yes | Oxalic Acid | Yes | Pollen substitute, Honey from your own stock | none |
| 139 | 2017 | 1.200528 | Yes | Sugar roll | Yes | Oxalic Acid | Yes | Pollen substitute, Honey from your own stock | none |
| 140 | 2017 | 1.200528 | Yes | Sugar roll | Yes | Oxalic Acid | Yes | Pollen substitute, Honey from your own stock | none |
| 141 | 2017 | 1.200528 | Yes | Sugar roll | Yes | Oxalic Acid | Yes | Pollen substitute, Honey from your own stock | none |
| 142 | 2017 | 1.200528 | Yes | Sugar roll | Yes | Oxalic Acid | Yes | Pollen substitute, Honey from your own stock | none |
| 143 | 2017 | 1.200528 | Yes | Sugar roll | Yes | Oxalic Acid | Yes | Pollen substitute, Honey from your own stock | none |
| 144 | 2017 | 1.200528 | Yes | Sugar roll | Yes | Oxalic Acid | Yes | Pollen substitute, Honey from your own stock | none |
| 145 | 2017 | 3.91081 | Yes | Drone brood inspection | Yes | Oxalic Acid | Yes | Dry sugar | sugar only |
| 146 | 2017 | 3.91081 | Yes | Drone brood inspection | Yes | Oxalic Acid | Yes | Dry sugar | sugar only |
| 147 | 2017 | 3.91081 | Yes | Drone brood inspection | Yes | Oxalic Acid | Yes | Dry sugar | sugar only |
| 148 | 2017 | 0.818784 | Yes | Sugar roll | Yes | Formic Acid (Mite Away Quick Strips) | Yes | Honey from your own stock | none |
| 149 | 2017 | 0.818784 | Yes | Sugar roll | Yes | Formic Acid (Mite Away Quick Strips) | Yes | Honey from your own stock | none |
| 150 | 2017 | 0.818784 | Yes | Sugar roll | Yes | Formic Acid (Mite Away Quick Strips) | Yes | Honey from your own stock | none |
| 151 | 2017 | 0.818784 | Yes | Sugar roll | Yes | Formic Acid (Mite Away Quick Strips) | Yes | Honey from your own stock | none |
| 152 | 2017 | 0.818784 | Yes | Sugar roll | Yes | Formic Acid (Mite Away Quick Strips) | Yes | Honey from your own stock | none |
| 153 | 2017 | 0.818784 | Yes | Sugar roll | Yes | Formic Acid (Mite Away Quick Strips) | Yes | Honey from your own stock | none |
| 154 | 2017 | 0.818784 | Yes | Sugar roll | Yes | Formic Acid (Mite Away Quick Strips) | Yes | Honey from your own stock | none |
| 155 | 2017 | 0.818784 | Yes | Sugar roll | Yes | Formic Acid (Mite Away Quick Strips) | Yes | Honey from your own stock | none |
| 156 | 2017 | 1.232476 | No | NA | Yes | Formic Acid (Mite Away Quick Strips) | Yes | Fondant or sugar candy | sugar only |
| 157 | 2017 | 1.232476 | No | NA | Yes | Formic Acid (Mite Away Quick Strips) | Yes | Fondant or sugar candy | sugar only |
| 158 | 2017 | 1.232476 | No | NA | Yes | Formic Acid (Mite Away Quick Strips) | Yes | Fondant or sugar candy | sugar only |
| 159 | 2017 | 1.190893 | Yes | Sugar roll | Yes | Oxalic Acid | Yes | Fondant or sugar candy | sugar only |
| 160 | 2017 | 1.190893 | Yes | Sugar roll | Yes | Oxalic Acid | Yes | Fondant or sugar candy | sugar only |
| 161 | 2017 | 1.190893 | Yes | Sugar roll | Yes | Oxalic Acid | Yes | Fondant or sugar candy | sugar only |
| 162 | 2017 | 1.190893 | Yes | Sugar roll | Yes | Oxalic Acid | Yes | Fondant or sugar candy | sugar only |
| 163 | 2017 | 1.190893 | Yes | Sugar roll | Yes | Oxalic Acid | Yes | Fondant or sugar candy | sugar only |
| 164 | 2017 | 1.190893 | Yes | Sugar roll | Yes | Oxalic Acid | Yes | Fondant or sugar candy | sugar only |
| 165 | 2017 | 1.190893 | Yes | Sugar roll | Yes | Oxalic Acid | Yes | Fondant or sugar candy | sugar only |
| 166 | 2017 | 1.190893 | Yes | Sugar roll | Yes | Oxalic Acid | Yes | Fondant or sugar candy | sugar only |
| 167 | 2017 | 1.190893 | Yes | Sugar roll | Yes | Oxalic Acid | Yes | Fondant or sugar candy | sugar only |
| 168 | 2017 | 1.190893 | Yes | Sugar roll | Yes | Oxalic Acid | Yes | Fondant or sugar candy | sugar only |
| 169 | 2017 | 1.190893 | Yes | Sugar roll | Yes | Oxalic Acid | Yes | Fondant or sugar candy | sugar only |
| 170 | 2017 | 1.190893 | Yes | Sugar roll | Yes | Oxalic Acid | Yes | Fondant or sugar candy | sugar only |
| 171 | 2017 | 1.190893 | Yes | Sugar roll | Yes | Oxalic Acid | Yes | Fondant or sugar candy | sugar only |
| 172 | 2017 | 1.190893 | Yes | Sugar roll | Yes | Oxalic Acid | Yes | Fondant or sugar candy | sugar only |
| 173 | 2017 | 1.190893 | Yes | Sugar roll | Yes | Oxalic Acid | Yes | Fondant or sugar candy | sugar only |
| 174 | 2017 | 1.190893 | Yes | Sugar roll | Yes | Oxalic Acid | Yes | Fondant or sugar candy | sugar only |
| 175 | 2017 | 1.190893 | Yes | Sugar roll | Yes | Oxalic Acid | Yes | Fondant or sugar candy | sugar only |
| 176 | 2017 | 1.190893 | Yes | Sugar roll | Yes | Oxalic Acid | Yes | Fondant or sugar candy | sugar only |
| 177 | 2017 | 1.190893 | Yes | Sugar roll | Yes | Oxalic Acid | Yes | Fondant or sugar candy | sugar only |
| 178 | 2017 | 1.190893 | Yes | Sugar roll | Yes | Oxalic Acid | Yes | Fondant or sugar candy | sugar only |
| 179 | 2017 | 1.190893 | Yes | Sugar roll | Yes | Oxalic Acid | Yes | Fondant or sugar candy | sugar only |
| 180 | 2017 | 1.190893 | Yes | Sugar roll | Yes | Oxalic Acid | Yes | Fondant or sugar candy | sugar only |
| 181 | 2017 | 1.190893 | Yes | Sugar roll | Yes | Oxalic Acid | Yes | Fondant or sugar candy | sugar only |
| 182 | 2017 | 1.190893 | Yes | Sugar roll | Yes | Oxalic Acid | Yes | Fondant or sugar candy | sugar only |
| 183 | 2017 | 1.190893 | Yes | Sugar roll | Yes | Oxalic Acid | Yes | Fondant or sugar candy | sugar only |
| 184 | 2017 | 3.278686 | No | NA | Yes | Formic Acid (Mite Away Quick Strips) | Yes | Fondant or sugar candy, Sugar syrup, Dry sugar | none |
| 185 | 2017 | 3.278686 | No | NA | Yes | Formic Acid (Mite Away Quick Strips) | Yes | Fondant or sugar candy, Sugar syrup, Dry sugar | none |
| 186 | 2017 | 3.278686 | No | NA | Yes | Formic Acid (Mite Away Quick Strips) | Yes | Fondant or sugar candy, Sugar syrup, Dry sugar | none |
| 187 | 2017 | 3.278686 | No | NA | Yes | Formic Acid (Mite Away Quick Strips) | Yes | Fondant or sugar candy, Sugar syrup, Dry sugar | none |
| 188 | 2017 | 3.278686 | No | NA | Yes | Formic Acid (Mite Away Quick Strips) | Yes | Fondant or sugar candy, Sugar syrup, Dry sugar | none |
| 189 | 2017 | 3.278686 | No | NA | Yes | Formic Acid (Mite Away Quick Strips) | Yes | Fondant or sugar candy, Sugar syrup, Dry sugar | none |
| 190 | 2017 | 3.278686 | No | NA | Yes | Formic Acid (Mite Away Quick Strips) | Yes | Fondant or sugar candy, Sugar syrup, Dry sugar | none |
| 191 | 2017 | 3.278686 | No | NA | Yes | Formic Acid (Mite Away Quick Strips) | Yes | Fondant or sugar candy, Sugar syrup, Dry sugar | none |
| 192 | 2017 | 0.585184 | Yes | Sugar roll | Yes | Hop guard | Yes | Fondant or sugar candy, Sugar syrup, Honey from your own stock | none |
| 193 | 2017 | 0.585184 | Yes | Sugar roll | Yes | Hop guard | Yes | Fondant or sugar candy, Sugar syrup, Honey from your own stock | none |
| 194 | 2017 | 0.585184 | Yes | Sugar roll | Yes | Hop guard | Yes | Fondant or sugar candy, Sugar syrup, Honey from your own stock | none |
| 195 | 2017 | 0.585184 | Yes | Sugar roll | Yes | Hop guard | Yes | Fondant or sugar candy, Sugar syrup, Honey from your own stock | none |
| 196 | 2017 | 0.585184 | Yes | Sugar roll | Yes | Hop guard | Yes | Fondant or sugar candy, Sugar syrup, Honey from your own stock | none |
| 197 | 2017 | 0.585184 | Yes | Sugar roll | Yes | Hop guard | Yes | Fondant or sugar candy, Sugar syrup, Honey from your own stock | none |
| 198 | 2017 | 0.585184 | Yes | Sugar roll | Yes | Hop guard | Yes | Fondant or sugar candy, Sugar syrup, Honey from your own stock | none |
| 199 | 2017 | 0.585184 | Yes | Sugar roll | Yes | Hop guard | Yes | Fondant or sugar candy, Sugar syrup, Honey from your own stock | none |
| 200 | 2017 | 0.585184 | Yes | Sugar roll | Yes | Hop guard | Yes | Fondant or sugar candy, Sugar syrup, Honey from your own stock | none |
| 201 | 2017 | 0.585184 | Yes | Sugar roll | Yes | Hop guard | Yes | Fondant or sugar candy, Sugar syrup, Honey from your own stock | none |
| 202 | 2017 | 0.585184 | Yes | Sugar roll | Yes | Hop guard | Yes | Fondant or sugar candy, Sugar syrup, Honey from your own stock | none |
| 203 | 2017 | 26.69584 | Yes | Sugar roll | Yes | Oxalic Acid | Yes | bee patties | none |
| 204 | 2017 | 26.69584 | Yes | Sugar roll | Yes | Oxalic Acid | Yes | bee patties | none |
| 205 | 2017 | 26.69584 | Yes | Sugar roll | Yes | Oxalic Acid | Yes | bee patties | none |
| 206 | 2017 | 26.69584 | Yes | Sugar roll | Yes | Oxalic Acid | Yes | bee patties | none |
| 207 | 2017 | 26.69584 | Yes | Sugar roll | Yes | Oxalic Acid | Yes | bee patties | none |
| 208 | 2018 | 7.522389 | No | NA | Yes | Hopguard | Yes | Fondant or sugar candy, Dry sugar | none |
| 209 | 2018 | 7.522389 | No | NA | Yes | Hopguard | Yes | Fondant or sugar candy, Dry sugar | none |
| 210 | 2018 | 7.522389 | No | NA | Yes | Hopguard | Yes | Fondant or sugar candy, Dry sugar | none |
| 211 | 2018 | 1.394164 | Yes | Alcohol wash | Yes | Oxalic Acid | Yes | Dry sugar, winter patties from Mann Lake | none |
| 212 | 2018 | 1.394164 | Yes | Alcohol wash | Yes | Oxalic Acid | Yes | Dry sugar, winter patties from Mann Lake | none |
| 213 | 2018 | 1.394164 | Yes | Alcohol wash | Yes | Oxalic Acid | Yes | Dry sugar, winter patties from Mann Lake | none |
| 214 | 2018 | 1.394164 | Yes | Alcohol wash | Yes | Oxalic Acid | Yes | Dry sugar, winter patties from Mann Lake | none |
| 215 | 2018 | 1.394164 | Yes | Alcohol wash | Yes | Oxalic Acid | Yes | Dry sugar, winter patties from Mann Lake | none |
| 216 | 2018 | 1.394164 | Yes | Alcohol wash | Yes | Oxalic Acid | Yes | Dry sugar, winter patties from Mann Lake | none |
| 217 | 2018 | 1.394164 | Yes | Alcohol wash | Yes | Oxalic Acid | Yes | Dry sugar, winter patties from Mann Lake | none |
| 218 | 2018 | 4.904595 | Yes | Drone brood inspection | Yes | Oxalic Acid | Yes | Fondant or sugar candy, Honey from your own stock | none |
| 219 | 2018 | 4.904595 | Yes | Drone brood inspection | Yes | Oxalic Acid | Yes | Fondant or sugar candy, Honey from your own stock | none |
| 220 | 2018 | 4.904595 | Yes | Drone brood inspection | Yes | Oxalic Acid | Yes | Fondant or sugar candy, Honey from your own stock | none |
| 221 | 2018 | 4.904595 | Yes | Drone brood inspection | Yes | Oxalic Acid | Yes | Fondant or sugar candy, Honey from your own stock | none |
| 222 | 2018 | 4.904595 | Yes | Drone brood inspection | Yes | Oxalic Acid | Yes | Fondant or sugar candy, Honey from your own stock | none |
| 223 | 2018 | 4.904595 | Yes | Drone brood inspection | Yes | Oxalic Acid | Yes | Fondant or sugar candy, Honey from your own stock | none |
| 224 | 2018 | 4.904595 | Yes | Drone brood inspection | Yes | Oxalic Acid | Yes | Fondant or sugar candy, Honey from your own stock | none |
| 225 | 2018 | 1.678768 | No | NA | Yes | Apivar (Amitraz) | Yes | Fondant or sugar candy, Honey from your own stock | none |
| 226 | 2018 | 1.678768 | No | NA | Yes | Apivar (Amitraz) | Yes | Fondant or sugar candy, Honey from your own stock | none |
| 227 | 2018 | 8.421124 | Yes | Sugar roll | Yes | Oxalic Acid | Yes | Fondant or sugar candy, Sugar syrup, Pollen substitute, Honey from your own stock | none |
| 228 | 2018 | 8.421124 | Yes | Sugar roll | Yes | Oxalic Acid | Yes | Fondant or sugar candy, Sugar syrup, Pollen substitute, Honey from your own stock | none |
| 229 | 2018 | 42.24231 | No | NA | Yes | Formic Acid (Mite Away Quick Strips) | Yes | Fondant or sugar candy | sugar only |
| 230 | 2018 | 42.24231 | No | NA | Yes | Formic Acid (Mite Away Quick Strips) | Yes | Fondant or sugar candy | sugar only |
| 231 | 2018 | 42.24231 | No | NA | Yes | Formic Acid (Mite Away Quick Strips) | Yes | Fondant or sugar candy | sugar only |
| 232 | 2018 | 42.24231 | No | NA | Yes | Formic Acid (Mite Away Quick Strips) | Yes | Fondant or sugar candy | sugar only |
| 233 | 2018 | 42.24231 | No | NA | Yes | Formic Acid (Mite Away Quick Strips) | Yes | Fondant or sugar candy | sugar only |
| 234 | 2018 | 8.976162 | Yes | 48 hr drop | Yes | Oxalic Acid | Yes | Fondant or sugar candy | sugar only |
| 235 | 2018 | 8.976162 | Yes | 48 hr drop | Yes | Oxalic Acid | Yes | Fondant or sugar candy | sugar only |
| 236 | 2018 | 8.976162 | Yes | 48 hr drop | Yes | Oxalic Acid | Yes | Fondant or sugar candy | sugar only |
| 237 | 2018 | 9.461093 | Yes | Sugar roll | Yes | Formic Acid (Mite Away Quick Strips) | Yes | Commercially available supplements | none |
| 238 | 2018 | 9.461093 | Yes | Sugar roll | Yes | Formic Acid (Mite Away Quick Strips) | Yes | Commercially available supplements | none |
| 239 | 2018 | 11.37215 | Yes | Sugar roll | Yes | Oxalic Acid | Yes | Fondant or sugar candy | sugar only |
| 240 | 2018 | 11.37215 | Yes | Sugar roll | Yes | Oxalic Acid | Yes | Fondant or sugar candy | sugar only |
| 241 | 2018 | 21.18615 | Yes | Sugar roll | Yes | Oxalic Acid | Yes | Dry sugar, Pollen substitute, Honey bee healthy | none |
| 242 | 2018 | 21.18615 | Yes | Sugar roll | Yes | Oxalic Acid | Yes | Dry sugar, Pollen substitute, Honey bee healthy | none |
| 243 | 2018 | 5.177119 | Yes | 48 hr drop | Yes | Formic Acid (Mite Away Quick Strips) | Yes | Fondant or sugar candy | sugar only |
| 244 | 2018 | 5.177119 | Yes | 48 hr drop | Yes | Formic Acid (Mite Away Quick Strips) | Yes | Fondant or sugar candy | sugar only |
| 245 | 2018 | 1.592621 | Yes | Sugar roll | Yes | Oxalic Acid | Yes | Sugar syrup, Dry sugar | none |
| 246 | 2018 | 1.592621 | Yes | Sugar roll | Yes | Oxalic Acid | Yes | Sugar syrup, Dry sugar | none |
| 247 | 2018 | 1.592621 | Yes | Sugar roll | Yes | Oxalic Acid | Yes | Sugar syrup, Dry sugar | none |
| 248 | 2018 | 1.592621 | Yes | Sugar roll | Yes | Oxalic Acid | Yes | Sugar syrup, Dry sugar | none |
| 249 | 2018 | 1.592621 | Yes | Sugar roll | Yes | Oxalic Acid | Yes | Sugar syrup, Dry sugar | none |
| 250 | 2018 | 1.592621 | Yes | Sugar roll | Yes | Oxalic Acid | Yes | Sugar syrup, Dry sugar | none |
| 251 | 2018 | 1.592621 | Yes | Sugar roll | Yes | Oxalic Acid | Yes | Sugar syrup, Dry sugar | none |
| 252 | 2018 | 1.592621 | Yes | Sugar roll | Yes | Oxalic Acid | Yes | Sugar syrup, Dry sugar | none |
| 253 | 2018 | 1.592621 | Yes | Sugar roll | Yes | Oxalic Acid | Yes | Sugar syrup, Dry sugar | none |
| 254 | 2018 | 1.592621 | Yes | Sugar roll | Yes | Oxalic Acid | Yes | Sugar syrup, Dry sugar | none |
| 255 | 2018 | 1.592621 | Yes | Sugar roll | Yes | Oxalic Acid | Yes | Sugar syrup, Dry sugar | none |
| 256 | 2018 | 1.592621 | Yes | Sugar roll | Yes | Oxalic Acid | Yes | Sugar syrup, Dry sugar | none |
| 257 | 2018 | 1.592621 | Yes | Sugar roll | Yes | Oxalic Acid | Yes | Sugar syrup, Dry sugar | none |
| 258 | 2018 | 1.592621 | Yes | Sugar roll | Yes | Oxalic Acid | Yes | Sugar syrup, Dry sugar | none |
| 259 | 2018 | 1.592621 | Yes | Sugar roll | Yes | Oxalic Acid | Yes | Sugar syrup, Dry sugar | none |
| 260 | 2018 | 7.816038 | Yes | Sugar roll | Yes | Formic Acid (Mite Away Quick Strips) | Yes | Fondant or sugar candy | sugar only |
| 261 | 2018 | 7.816038 | Yes | Sugar roll | Yes | Formic Acid (Mite Away Quick Strips) | Yes | Fondant or sugar candy | sugar only |
| 262 | 2018 | 46.00931 | Yes | Alcohol wash | Yes | Oxalic Acid | Yes | Fondant or sugar candy, Sugar syrup | none |
| 263 | 2018 | 46.00931 | Yes | Alcohol wash | Yes | Oxalic Acid | Yes | Fondant or sugar candy, Sugar syrup | none |
| 264 | 2018 | 4.071777 | No | NA | Yes | Oxalic Acid | Yes | Fondant or sugar candy, Pollen substitute | none |
| 265 | 2018 | 4.071777 | No | NA | Yes | Oxalic Acid | Yes | Fondant or sugar candy, Pollen substitute | none |
| 266 | 2018 | 4.071777 | No | NA | Yes | Oxalic Acid | Yes | Fondant or sugar candy, Pollen substitute | none |
| 267 | 2018 | 4.071777 | No | NA | Yes | Oxalic Acid | Yes | Fondant or sugar candy, Pollen substitute | none |
| 268 | 2018 | 4.071777 | No | NA | Yes | Oxalic Acid | Yes | Fondant or sugar candy, Pollen substitute | none |
| 269 | 2018 | 4.071777 | No | NA | Yes | Oxalic Acid | Yes | Fondant or sugar candy, Pollen substitute | none |
| 270 | 2018 | 4.071777 | No | NA | Yes | Oxalic Acid | Yes | Fondant or sugar candy, Pollen substitute | none |
| 271 | 2018 | 4.071777 | No | NA | Yes | Oxalic Acid | Yes | Fondant or sugar candy, Pollen substitute | none |
| 272 | 2018 | 4.071777 | No | NA | Yes | Oxalic Acid | Yes | Fondant or sugar candy, Pollen substitute | none |
| 273 | 2018 | 4.071777 | No | NA | Yes | Oxalic Acid | Yes | Fondant or sugar candy, Pollen substitute | none |
| 274 | 2018 | 4.071777 | No | NA | Yes | Oxalic Acid | Yes | Fondant or sugar candy, Pollen substitute | none |
| 275 | 2018 | 4.071777 | No | NA | Yes | Oxalic Acid | Yes | Fondant or sugar candy, Pollen substitute | none |
| 276 | 2018 | 4.071777 | No | NA | Yes | Oxalic Acid | Yes | Fondant or sugar candy, Pollen substitute | none |
| 277 | 2018 | 4.071777 | No | NA | Yes | Oxalic Acid | Yes | Fondant or sugar candy, Pollen substitute | none |
| 278 | 2018 | 4.071777 | No | NA | Yes | Oxalic Acid | Yes | Fondant or sugar candy, Pollen substitute | none |
| 279 | 2018 | 4.071777 | No | NA | Yes | Oxalic Acid | Yes | Fondant or sugar candy, Pollen substitute | none |
| 280 | 2018 | 4.071777 | No | NA | Yes | Oxalic Acid | Yes | Fondant or sugar candy, Pollen substitute | none |
| 281 | 2018 | 4.071777 | No | NA | Yes | Oxalic Acid | Yes | Fondant or sugar candy, Pollen substitute | none |
| 282 | 2018 | 4.071777 | No | NA | Yes | Oxalic Acid | Yes | Fondant or sugar candy, Pollen substitute | none |
| 283 | 2018 | 1.32028 | No | NA | Yes | Apiguard | Yes | Fondant or sugar candy | sugar only |
| 284 | 2018 | 11.19338 | Yes | 48 hr drop, Drone brood inspection | Yes | Oxalic Acid | Yes | Dry sugar | sugar only |
| 285 | 2018 | 11.19338 | Yes | 48 hr drop, Drone brood inspection | Yes | Oxalic Acid | Yes | Dry sugar | sugar only |
| 286 | 2018 | 11.19338 | Yes | 48 hr drop, Drone brood inspection | Yes | Oxalic Acid | Yes | Dry sugar | sugar only |
| 287 | 2018 | 11.19338 | Yes | 48 hr drop, Drone brood inspection | Yes | Oxalic Acid | Yes | Dry sugar | sugar only |
| 288 | 2018 | 11.19338 | Yes | 48 hr drop, Drone brood inspection | Yes | Oxalic Acid | Yes | Dry sugar | sugar only |
| 289 | 2018 | 11.19338 | Yes | 48 hr drop, Drone brood inspection | Yes | Oxalic Acid | Yes | Dry sugar | sugar only |
| 290 | 2018 | 11.19338 | Yes | 48 hr drop, Drone brood inspection | Yes | Oxalic Acid | Yes | Dry sugar | sugar only |
| 291 | 2018 | 11.19338 | Yes | 48 hr drop, Drone brood inspection | Yes | Oxalic Acid | Yes | Dry sugar | sugar only |
| 292 | 2018 | 11.19338 | Yes | 48 hr drop, Drone brood inspection | Yes | Oxalic Acid | Yes | Dry sugar | sugar only |
| 293 | 2018 | 11.19338 | Yes | 48 hr drop, Drone brood inspection | Yes | Oxalic Acid | Yes | Dry sugar | sugar only |
| 294 | 2018 | 11.19338 | Yes | 48 hr drop, Drone brood inspection | Yes | Oxalic Acid | Yes | Dry sugar | sugar only |
| 295 | 2018 | 2.159079 | Yes | Sugar roll | Yes | Oxalic Acid | Yes | Fondant or sugar candy | sugar only |
| 296 | 2018 | 2.159079 | Yes | Sugar roll | Yes | Oxalic Acid | Yes | Fondant or sugar candy | sugar only |
| 297 | 2018 | 2.159079 | Yes | Sugar roll | Yes | Oxalic Acid | Yes | Fondant or sugar candy | sugar only |
| 298 | 2018 | 2.159079 | Yes | Sugar roll | Yes | Oxalic Acid | Yes | Fondant or sugar candy | sugar only |
| 299 | 2018 | 2.159079 | Yes | Sugar roll | Yes | Oxalic Acid | Yes | Fondant or sugar candy | sugar only |
| 300 | 2018 | 2.159079 | Yes | Sugar roll | Yes | Oxalic Acid | Yes | Fondant or sugar candy | sugar only |
| 301 | 2018 | 19.97254 | Yes | Sugar roll | Yes | Formic Acid (Mite Away Quick Strips) | Yes | Fondant or sugar candy, Honey from your own stock, Pollen from your own stock | none |
| 302 | 2018 | 19.97254 | Yes | Sugar roll | Yes | Formic Acid (Mite Away Quick Strips) | Yes | Fondant or sugar candy, Honey from your own stock, Pollen from your own stock | none |
| 303 | 2018 | 3.94617 | Yes | 48 hr drop, Sugar roll | Yes | Apivar (Amitraz) | No | Dry sugar, Pollen substitute | none |
| 304 | 2018 | 3.94617 | Yes | 48 hr drop, Sugar roll | Yes | Apivar (Amitraz) | No | Dry sugar, Pollen substitute | none |
| 305 | 2018 | 3.94617 | Yes | 48 hr drop, Sugar roll | Yes | Apivar (Amitraz) | No | Dry sugar, Pollen substitute | none |
| 306 | 2018 | 3.419697 | Yes | Alcohol wash | Yes | Formic Acid (Mite Away Quick Strips) | Yes | Sugar syrup | none |
| 307 | 2018 | 20.95261 | Yes | 48 hr drop | Yes | Formic Acid (Mite Away Quick Strips) | Yes | Fondant or sugar candy | sugar only |
| 308 | 2018 | 20.95261 | Yes | 48 hr drop | Yes | Formic Acid (Mite Away Quick Strips) | Yes | Fondant or sugar candy | sugar only |
| 309 | 2018 | 2.312939 | Yes | Sugar roll | Yes | Oxalic Acid | Yes | Fondant or sugar candy | sugar only |
| 310 | 2018 | 2.312939 | Yes | Sugar roll | Yes | Oxalic Acid | Yes | Fondant or sugar candy | sugar only |
| 311 | 2018 | 15.14522 | Yes | Sugar roll | Yes | Formic Acid (Mite Away Quick Strips) | Yes | Dry sugar | sugar only |
| 312 | 2018 | 1.207057 | Yes | Sugar roll | Yes | Formic Acid (Mite Away Quick Strips) | Yes | Fondant or sugar candy | sugar only |
| 313 | 2018 | 1.207057 | Yes | Sugar roll | Yes | Formic Acid (Mite Away Quick Strips) | Yes | Fondant or sugar candy | sugar only |
| 314 | 2018 | 1.207057 | Yes | Sugar roll | Yes | Formic Acid (Mite Away Quick Strips) | Yes | Fondant or sugar candy | sugar only |
| 315 | 2018 | 1.207057 | Yes | Sugar roll | Yes | Formic Acid (Mite Away Quick Strips) | Yes | Fondant or sugar candy | sugar only |
| 316 | 2018 | 1.207057 | Yes | Sugar roll | Yes | Formic Acid (Mite Away Quick Strips) | Yes | Fondant or sugar candy | sugar only |
| 317 | 2018 | 1.207057 | Yes | Sugar roll | Yes | Formic Acid (Mite Away Quick Strips) | Yes | Fondant or sugar candy | sugar only |
| 318 | 2018 | 1.207057 | Yes | Sugar roll | Yes | Formic Acid (Mite Away Quick Strips) | Yes | Fondant or sugar candy | sugar only |
| 319 | 2018 | 1.207057 | Yes | Sugar roll | Yes | Formic Acid (Mite Away Quick Strips) | Yes | Fondant or sugar candy | sugar only |
| 320 | 2018 | 1.207057 | Yes | Sugar roll | Yes | Formic Acid (Mite Away Quick Strips) | Yes | Fondant or sugar candy | sugar only |
| 321 | 2018 | 1.207057 | Yes | Sugar roll | Yes | Formic Acid (Mite Away Quick Strips) | Yes | Fondant or sugar candy | sugar only |
| 322 | 2018 | 1.207057 | Yes | Sugar roll | Yes | Formic Acid (Mite Away Quick Strips) | Yes | Fondant or sugar candy | sugar only |
| 323 | 2018 | 1.207057 | Yes | Sugar roll | Yes | Formic Acid (Mite Away Quick Strips) | Yes | Fondant or sugar candy | sugar only |
| 324 | 2018 | 1.207057 | Yes | Sugar roll | Yes | Formic Acid (Mite Away Quick Strips) | Yes | Fondant or sugar candy | sugar only |
| 325 | 2018 | 1.207057 | Yes | Sugar roll | Yes | Formic Acid (Mite Away Quick Strips) | Yes | Fondant or sugar candy | sugar only |
| 326 | 2018 | 1.207057 | Yes | Sugar roll | Yes | Formic Acid (Mite Away Quick Strips) | Yes | Fondant or sugar candy | sugar only |
| 327 | 2018 | 1.207057 | Yes | Sugar roll | Yes | Formic Acid (Mite Away Quick Strips) | Yes | Fondant or sugar candy | sugar only |
| 328 | 2018 | 1.207057 | Yes | Sugar roll | Yes | Formic Acid (Mite Away Quick Strips) | Yes | Fondant or sugar candy | sugar only |
| 329 | 2018 | 1.207057 | Yes | Sugar roll | Yes | Formic Acid (Mite Away Quick Strips) | Yes | Fondant or sugar candy | sugar only |
| 330 | 2018 | 1.207057 | Yes | Sugar roll | Yes | Formic Acid (Mite Away Quick Strips) | Yes | Fondant or sugar candy | sugar only |
| 331 | 2018 | 1.207057 | Yes | Sugar roll | Yes | Formic Acid (Mite Away Quick Strips) | Yes | Fondant or sugar candy | sugar only |
| 332 | 2018 | 1.207057 | Yes | Sugar roll | Yes | Formic Acid (Mite Away Quick Strips) | Yes | Fondant or sugar candy | sugar only |
| 333 | 2018 | 1.207057 | Yes | Sugar roll | Yes | Formic Acid (Mite Away Quick Strips) | Yes | Fondant or sugar candy | sugar only |
| 334 | 2018 | 1.207057 | Yes | Sugar roll | Yes | Formic Acid (Mite Away Quick Strips) | Yes | Fondant or sugar candy | sugar only |
| 335 | 2018 | 1.207057 | Yes | Sugar roll | Yes | Formic Acid (Mite Away Quick Strips) | Yes | Fondant or sugar candy | sugar only |
| 336 | 2018 | 1.207057 | Yes | Sugar roll | Yes | Formic Acid (Mite Away Quick Strips) | Yes | Fondant or sugar candy | sugar only |
| 337 | 2018 | 1.207057 | Yes | Sugar roll | Yes | Formic Acid (Mite Away Quick Strips) | Yes | Fondant or sugar candy | sugar only |
| 338 | 2018 | 2.422462 | Yes | Sugar roll | Yes | Oxalic Acid | Yes | Fondant or sugar candy | sugar only |
| 339 | 2018 | 2.422462 | Yes | Sugar roll | Yes | Oxalic Acid | Yes | Fondant or sugar candy | sugar only |
| 340 | 2018 | 5.749873 | Yes | Sugar roll, Alcohol wash | Yes | Formic Acid (Mite Away Quick Strips) | Yes | Fondant or sugar candy, Honey from your own stock, winter patties | none |
| 341 | 2018 | 5.749873 | Yes | Sugar roll, Alcohol wash | Yes | Formic Acid (Mite Away Quick Strips) | Yes | Fondant or sugar candy, Honey from your own stock, winter patties | none |
| 342 | 2018 | 5.749873 | Yes | Sugar roll, Alcohol wash | Yes | Formic Acid (Mite Away Quick Strips) | Yes | Fondant or sugar candy, Honey from your own stock, winter patties | none |
| 343 | 2018 | 5.749873 | Yes | Sugar roll, Alcohol wash | Yes | Formic Acid (Mite Away Quick Strips) | Yes | Fondant or sugar candy, Honey from your own stock, winter patties | none |
| 344 | 2018 | 5.749873 | Yes | Sugar roll, Alcohol wash | Yes | Formic Acid (Mite Away Quick Strips) | Yes | Fondant or sugar candy, Honey from your own stock, winter patties | none |
| 345 | 2018 | 5.749873 | Yes | Sugar roll, Alcohol wash | Yes | Formic Acid (Mite Away Quick Strips) | Yes | Fondant or sugar candy, Honey from your own stock, winter patties | none |
| 346 | 2018 | 5.749873 | Yes | Sugar roll, Alcohol wash | Yes | Formic Acid (Mite Away Quick Strips) | Yes | Fondant or sugar candy, Honey from your own stock, winter patties | none |
| 347 | 2018 | 5.749873 | Yes | Sugar roll, Alcohol wash | Yes | Formic Acid (Mite Away Quick Strips) | Yes | Fondant or sugar candy, Honey from your own stock, winter patties | none |
| 348 | 2018 | 5.749873 | Yes | Sugar roll, Alcohol wash | Yes | Formic Acid (Mite Away Quick Strips) | Yes | Fondant or sugar candy, Honey from your own stock, winter patties | none |
| 349 | 2018 | 5.749873 | Yes | Sugar roll, Alcohol wash | Yes | Formic Acid (Mite Away Quick Strips) | Yes | Fondant or sugar candy, Honey from your own stock, winter patties | none |
| 350 | 2018 | 5.749873 | Yes | Sugar roll, Alcohol wash | Yes | Formic Acid (Mite Away Quick Strips) | Yes | Fondant or sugar candy, Honey from your own stock, winter patties | none |
| 351 | 2018 | 5.749873 | Yes | Sugar roll, Alcohol wash | Yes | Formic Acid (Mite Away Quick Strips) | Yes | Fondant or sugar candy, Honey from your own stock, winter patties | none |
| 352 | 2018 | 5.749873 | Yes | Sugar roll, Alcohol wash | Yes | Formic Acid (Mite Away Quick Strips) | Yes | Fondant or sugar candy, Honey from your own stock, winter patties | none |
| 353 | 2018 | 5.749873 | Yes | Sugar roll, Alcohol wash | Yes | Formic Acid (Mite Away Quick Strips) | Yes | Fondant or sugar candy, Honey from your own stock, winter patties | none |
| 354 | 2018 | 5.749873 | Yes | Sugar roll, Alcohol wash | Yes | Formic Acid (Mite Away Quick Strips) | Yes | Fondant or sugar candy, Honey from your own stock, winter patties | none |
| 355 | 2018 | 5.749873 | Yes | Sugar roll, Alcohol wash | Yes | Formic Acid (Mite Away Quick Strips) | Yes | Fondant or sugar candy, Honey from your own stock, winter patties | none |
| 356 | 2018 | 5.749873 | Yes | Sugar roll, Alcohol wash | Yes | Formic Acid (Mite Away Quick Strips) | Yes | Fondant or sugar candy, Honey from your own stock, winter patties | none |
| 357 | 2018 | 41.46113 | Yes | Alcohol wash | Yes | Formic Acid (Mite Away Quick Strips) | Yes | Dry sugar | sugar only |
| 358 | 2018 | 41.46113 | Yes | Alcohol wash | Yes | Formic Acid (Mite Away Quick Strips) | Yes | Dry sugar | sugar only |
| 359 | 2018 | 41.46113 | Yes | Alcohol wash | Yes | Formic Acid (Mite Away Quick Strips) | Yes | Dry sugar | sugar only |
| 360 | 2018 | 41.46113 | Yes | Alcohol wash | Yes | Formic Acid (Mite Away Quick Strips) | Yes | Dry sugar | sugar only |
| 361 | 2018 | 41.46113 | Yes | Alcohol wash | Yes | Formic Acid (Mite Away Quick Strips) | Yes | Dry sugar | sugar only |
| 362 | 2018 | 41.46113 | Yes | Alcohol wash | Yes | Formic Acid (Mite Away Quick Strips) | Yes | Dry sugar | sugar only |
| 363 | 2018 | 41.46113 | Yes | Alcohol wash | Yes | Formic Acid (Mite Away Quick Strips) | Yes | Dry sugar | sugar only |
| 364 | 2018 | 41.46113 | Yes | Alcohol wash | Yes | Formic Acid (Mite Away Quick Strips) | Yes | Dry sugar | sugar only |
| 365 | 2018 | 41.46113 | Yes | Alcohol wash | Yes | Formic Acid (Mite Away Quick Strips) | Yes | Dry sugar | sugar only |
| 366 | 2018 | 0.552949 | Yes | Sugar roll | Yes | Formic Acid (Mite Away Quick Strips) | Yes | Fondant or sugar candy, Pollen substitute | none |
| 367 | 2018 | 0.552949 | Yes | Sugar roll | Yes | Formic Acid (Mite Away Quick Strips) | Yes | Fondant or sugar candy, Pollen substitute | none |
| 368 | 2018 | 1.366073 | Yes | Sugar roll | Yes | Oxalic Acid | Yes | Fondant or sugar candy, Sugar syrup, Dry sugar | none |
| 369 | 2018 | 1.366073 | Yes | Sugar roll | Yes | Oxalic Acid | Yes | Fondant or sugar candy, Sugar syrup, Dry sugar | none |
| 370 | 2018 | 1.366073 | Yes | Sugar roll | Yes | Oxalic Acid | Yes | Fondant or sugar candy, Sugar syrup, Dry sugar | none |
| 371 | 2018 | 1.366073 | Yes | Sugar roll | Yes | Oxalic Acid | Yes | Fondant or sugar candy, Sugar syrup, Dry sugar | none |
| 372 | 2018 | 1.366073 | Yes | Sugar roll | Yes | Oxalic Acid | Yes | Fondant or sugar candy, Sugar syrup, Dry sugar | none |
| 373 | 2018 | 1.366073 | Yes | Sugar roll | Yes | Oxalic Acid | Yes | Fondant or sugar candy, Sugar syrup, Dry sugar | none |
| 374 | 2018 | 1.366073 | Yes | Sugar roll | Yes | Oxalic Acid | Yes | Fondant or sugar candy, Sugar syrup, Dry sugar | none |
| 375 | 2018 | 1.366073 | Yes | Sugar roll | Yes | Oxalic Acid | Yes | Fondant or sugar candy, Sugar syrup, Dry sugar | none |
| 376 | 2018 | 1.366073 | Yes | Sugar roll | Yes | Oxalic Acid | Yes | Fondant or sugar candy, Sugar syrup, Dry sugar | none |
| 377 | 2018 | 1.366073 | Yes | Sugar roll | Yes | Oxalic Acid | Yes | Fondant or sugar candy, Sugar syrup, Dry sugar | none |
| 378 | 2018 | 1.366073 | Yes | Sugar roll | Yes | Oxalic Acid | Yes | Fondant or sugar candy, Sugar syrup, Dry sugar | none |
| 379 | 2018 | 1.366073 | Yes | Sugar roll | Yes | Oxalic Acid | Yes | Fondant or sugar candy, Sugar syrup, Dry sugar | none |
| 380 | 2018 | 1.366073 | Yes | Sugar roll | Yes | Oxalic Acid | Yes | Fondant or sugar candy, Sugar syrup, Dry sugar | none |
| 381 | 2018 | 1.366073 | Yes | Sugar roll | Yes | Oxalic Acid | Yes | Fondant or sugar candy, Sugar syrup, Dry sugar | none |
| 382 | 2018 | 0.517928 | Yes | Sugar roll, Alcohol wash | Yes | Api life Var | Yes | Fondant or sugar candy | sugar only |
| 383 | 2018 | 12.65538 | No | NA | Yes | Formic Acid (Mite Away Quick Strips) | Yes | Fondant or sugar candy | sugar only |
| 384 | 2018 | 12.65538 | No | NA | Yes | Formic Acid (Mite Away Quick Strips) | Yes | Fondant or sugar candy | sugar only |
| 385 | 2018 | 12.65538 | No | NA | Yes | Formic Acid (Mite Away Quick Strips) | Yes | Fondant or sugar candy | sugar only |
| 386 | 2018 | 12.65538 | No | NA | Yes | Formic Acid (Mite Away Quick Strips) | Yes | Fondant or sugar candy | sugar only |
| 387 | 2018 | 12.65538 | No | NA | Yes | Formic Acid (Mite Away Quick Strips) | Yes | Fondant or sugar candy | sugar only |
| 388 | 2018 | 12.65538 | No | NA | Yes | Formic Acid (Mite Away Quick Strips) | Yes | Fondant or sugar candy | sugar only |
| 389 | 2018 | 12.65538 | No | NA | Yes | Formic Acid (Mite Away Quick Strips) | Yes | Fondant or sugar candy | sugar only |
| 390 | 2018 | 12.65538 | No | NA | Yes | Formic Acid (Mite Away Quick Strips) | Yes | Fondant or sugar candy | sugar only |
| 391 | 2018 | 12.65538 | No | NA | Yes | Formic Acid (Mite Away Quick Strips) | Yes | Fondant or sugar candy | sugar only |
| 392 | 2018 | 15.36603 | Yes | Drone brood inspection | Yes | Formic Acid (Mite Away Quick Strips) | Yes | Fondant or sugar candy, Dry sugar | none |
| 393 | 2018 | 15.36603 | Yes | Drone brood inspection | Yes | Formic Acid (Mite Away Quick Strips) | Yes | Fondant or sugar candy, Dry sugar | none |
| 394 | 2018 | 3.063161 | Yes | Sugar roll | Yes | Formic Acid (Mite Away Quick Strips) | Yes | Fondant or sugar candy | sugar only |
| 395 | 2018 | 50.22795 | Yes | 48 hr drop, Alcohol wash | Yes | Formic Acid (Mite Away Quick Strips) | Yes | Fondant or sugar candy | sugar only |
| 396 | 2018 | 50.22795 | Yes | 48 hr drop, Alcohol wash | Yes | Formic Acid (Mite Away Quick Strips) | Yes | Fondant or sugar candy | sugar only |
| 397 | 2018 | 50.22795 | Yes | 48 hr drop, Alcohol wash | Yes | Formic Acid (Mite Away Quick Strips) | Yes | Fondant or sugar candy | sugar only |
| 398 | 2018 | 50.22795 | Yes | 48 hr drop, Alcohol wash | Yes | Formic Acid (Mite Away Quick Strips) | Yes | Fondant or sugar candy | sugar only |
| 399 | 2018 | 1.147545 | Yes | Take pictures of the frames , then zoom in on computer. | Yes | Formic Acid (Mite Away Quick Strips) | Yes | Fondant or sugar candy | sugar only |
| 400 | 2018 | 1.147545 | Yes | Take pictures of the frames , then zoom in on computer. | Yes | Formic Acid (Mite Away Quick Strips) | Yes | Fondant or sugar candy | sugar only |
| 401 | 2018 | 1.147545 | Yes | Take pictures of the frames , then zoom in on computer. | Yes | Formic Acid (Mite Away Quick Strips) | Yes | Fondant or sugar candy | sugar only |
| 402 | 2018 | 17.12619 | Yes | Sugar roll | Yes | Apivar (Amitraz) | Yes | Fondant or sugar candy, Sugar syrup | none |
| 403 | 2018 | 17.12619 | Yes | Sugar roll | Yes | Apivar (Amitraz) | Yes | Fondant or sugar candy, Sugar syrup | none |
| 404 | 2018 | 17.12619 | Yes | Sugar roll | Yes | Apivar (Amitraz) | Yes | Fondant or sugar candy, Sugar syrup | none |
| 405 | 2018 | 4.722392 | Yes | Sugar roll | Yes | Oxalic Acid | Yes | Fondant or sugar candy, Pollen substitute | none |
| 406 | 2018 | 4.722392 | Yes | Sugar roll | Yes | Oxalic Acid | Yes | Fondant or sugar candy, Pollen substitute | none |
| 407 | 2018 | 4.722392 | Yes | Sugar roll | Yes | Oxalic Acid | Yes | Fondant or sugar candy, Pollen substitute | none |
| 408 | 2018 | 4.722392 | Yes | Sugar roll | Yes | Oxalic Acid | Yes | Fondant or sugar candy, Pollen substitute | none |
| 409 | 2018 | 4.722392 | Yes | Sugar roll | Yes | Oxalic Acid | Yes | Fondant or sugar candy, Pollen substitute | none |
| 410 | 2018 | 4.722392 | Yes | Sugar roll | Yes | Oxalic Acid | Yes | Fondant or sugar candy, Pollen substitute | none |
| 411 | 2018 | 4.722392 | Yes | Sugar roll | Yes | Oxalic Acid | Yes | Fondant or sugar candy, Pollen substitute | none |
| 412 | 2018 | 4.722392 | Yes | Sugar roll | Yes | Oxalic Acid | Yes | Fondant or sugar candy, Pollen substitute | none |
| 413 | 2018 | 3.393356 | Yes | 48 hr drop | Yes | Oxalic Acid | Yes | Sugar syrup | none |
| 414 | 2019 | 8.55092 | Yes | Alcohol wash, Drone brood inspection | Yes | Oxalic Acid | Yes | Fondant or sugar candy, Dry sugar | none |
| 415 | 2019 | 8.55092 | Yes | Alcohol wash, Drone brood inspection | Yes | Oxalic Acid | Yes | Fondant or sugar candy, Dry sugar | none |
| 416 | 2019 | 8.55092 | Yes | Alcohol wash, Drone brood inspection | Yes | Oxalic Acid | Yes | Fondant or sugar candy, Dry sugar | none |
| 417 | 2019 | 8.55092 | Yes | Alcohol wash, Drone brood inspection | Yes | Oxalic Acid | Yes | Fondant or sugar candy, Dry sugar | none |
| 418 | 2019 | 8.55092 | Yes | Alcohol wash, Drone brood inspection | Yes | Oxalic Acid | Yes | Fondant or sugar candy, Dry sugar | none |
| 419 | 2019 | 8.55092 | Yes | Alcohol wash, Drone brood inspection | Yes | Oxalic Acid | Yes | Fondant or sugar candy, Dry sugar | none |
| 420 | 2019 | 8.55092 | Yes | Alcohol wash, Drone brood inspection | Yes | Oxalic Acid | Yes | Fondant or sugar candy, Dry sugar | none |
| 421 | 2019 | 8.55092 | Yes | Alcohol wash, Drone brood inspection | Yes | Oxalic Acid | Yes | Fondant or sugar candy, Dry sugar | none |
| 422 | 2019 | 8.55092 | Yes | Alcohol wash, Drone brood inspection | Yes | Oxalic Acid | Yes | Fondant or sugar candy, Dry sugar | none |
| 423 | 2019 | 8.55092 | Yes | Alcohol wash, Drone brood inspection | Yes | Oxalic Acid | Yes | Fondant or sugar candy, Dry sugar | none |
| 424 | 2019 | 8.55092 | Yes | Alcohol wash, Drone brood inspection | Yes | Oxalic Acid | Yes | Fondant or sugar candy, Dry sugar | none |
| 425 | 2019 | 8.55092 | Yes | Alcohol wash, Drone brood inspection | Yes | Oxalic Acid | Yes | Fondant or sugar candy, Dry sugar | none |
| 426 | 2019 | 4.230763 | Yes | 48 hr drop (sticky board), Alcohol wash | Yes | Formic Acid (Mite Away Quick Strips) | Yes | Commercially available supplements | none |
| 427 | 2019 | 4.230763 | Yes | 48 hr drop (sticky board), Alcohol wash | Yes | Formic Acid (Mite Away Quick Strips) | Yes | Commercially available supplements | none |
| 428 | 2019 | 21.74279 | Yes | Alcohol wash | Yes | Oxalic Acid | Yes | Fondant or sugar candy, Sugar syrup | none |
| 429 | 2019 | 21.74279 | Yes | Alcohol wash | Yes | Oxalic Acid | Yes | Fondant or sugar candy, Sugar syrup | none |
| 430 | 2019 | 15.47573 | Yes | Drone brood inspection | Yes | Formic Acid (Mite Away Quick Strips) | Yes | Fondant or sugar candy, Pollen substitute | none |
| 431 | 2019 | 15.47573 | Yes | Drone brood inspection | Yes | Formic Acid (Mite Away Quick Strips) | Yes | Fondant or sugar candy, Pollen substitute | none |
| 432 | 2019 | 20.43529 | No | NA | Yes | Formic Acid (Mite Away Quick Strips) | Yes | Fondant or sugar candy, Pollen substitute | none |
| 433 | 2019 | 20.43529 | No | NA | Yes | Formic Acid (Mite Away Quick Strips) | Yes | Fondant or sugar candy, Pollen substitute | none |
| 434 | 2019 | 20.43529 | No | NA | Yes | Formic Acid (Mite Away Quick Strips) | Yes | Fondant or sugar candy, Pollen substitute | none |
| 435 | 2019 | 20.43529 | No | NA | Yes | Formic Acid (Mite Away Quick Strips) | Yes | Fondant or sugar candy, Pollen substitute | none |
| 436 | 2019 | 20.43529 | No | NA | Yes | Formic Acid (Mite Away Quick Strips) | Yes | Fondant or sugar candy, Pollen substitute | none |
| 437 | 2019 | 20.43529 | No | NA | Yes | Formic Acid (Mite Away Quick Strips) | Yes | Fondant or sugar candy, Pollen substitute | none |
| 438 | 2019 | 20.43529 | No | NA | Yes | Formic Acid (Mite Away Quick Strips) | Yes | Fondant or sugar candy, Pollen substitute | none |
| 439 | 2019 | 20.43529 | No | NA | Yes | Formic Acid (Mite Away Quick Strips) | Yes | Fondant or sugar candy, Pollen substitute | none |
| 440 | 2019 | 20.43529 | No | NA | Yes | Formic Acid (Mite Away Quick Strips) | Yes | Fondant or sugar candy, Pollen substitute | none |
| 441 | 2019 | 3.780241 | No | NA | Yes | Oxalic Acid | Yes | Fondant or sugar candy | sugar only |
| 442 | 2019 | 3.780241 | No | NA | Yes | Oxalic Acid | Yes | Fondant or sugar candy | sugar only |
| 443 | 2019 | 3.780241 | No | NA | Yes | Oxalic Acid | Yes | Fondant or sugar candy | sugar only |
| 444 | 2019 | 3.780241 | No | NA | Yes | Oxalic Acid | Yes | Fondant or sugar candy | sugar only |
| 445 | 2019 | 3.780241 | No | NA | Yes | Oxalic Acid | Yes | Fondant or sugar candy | sugar only |
| 446 | 2019 | 3.780241 | No | NA | Yes | Oxalic Acid | Yes | Fondant or sugar candy | sugar only |
| 447 | 2019 | 3.780241 | No | NA | Yes | Oxalic Acid | Yes | Fondant or sugar candy | sugar only |
| 448 | 2019 | 3.780241 | No | NA | Yes | Oxalic Acid | Yes | Fondant or sugar candy | sugar only |
| 449 | 2019 | 3.780241 | No | NA | Yes | Oxalic Acid | Yes | Fondant or sugar candy | sugar only |
| 450 | 2019 | 3.780241 | No | NA | Yes | Oxalic Acid | Yes | Fondant or sugar candy | sugar only |
| 451 | 2019 | 20.87631 | Yes | 48 hr drop (sticky board) | Yes | Oxalic Acid | Yes | Fondant or sugar candy | sugar only |
| 452 | 2019 | 20.87631 | Yes | 48 hr drop (sticky board) | Yes | Oxalic Acid | Yes | Fondant or sugar candy | sugar only |
| 453 | 2019 | 7.7995 | Yes | Alcohol wash | Yes | Formic Acid (Mite Away Quick Strips) | Yes | Fondant or sugar candy, Pollen substitute, Honey from your own stock | none |
| 454 | 2019 | 7.7995 | Yes | Alcohol wash | Yes | Formic Acid (Mite Away Quick Strips) | Yes | Fondant or sugar candy, Pollen substitute, Honey from your own stock | none |
| 455 | 2019 | 7.7995 | Yes | Alcohol wash | Yes | Formic Acid (Mite Away Quick Strips) | Yes | Fondant or sugar candy, Pollen substitute, Honey from your own stock | none |
| 456 | 2019 | 32.81493 | Yes | Sugar roll | Yes | Formic Acid (Mite Away Quick Strips) | Yes | Fondant or sugar candy, Pollen substitute | none |
| 457 | 2019 | 10.44072 | Yes | Alcohol wash | Yes | Oxalic Acid | Yes | Fondant or sugar candy, Pollen substitute, Prairie Pride Vitamins & Electrolytes | none |
| 458 | 2019 | 10.44072 | Yes | Alcohol wash | Yes | Oxalic Acid | Yes | Fondant or sugar candy, Pollen substitute, Prairie Pride Vitamins & Electrolytes | none |
| 459 | 2019 | 10.44072 | Yes | Alcohol wash | Yes | Oxalic Acid | Yes | Fondant or sugar candy, Pollen substitute, Prairie Pride Vitamins & Electrolytes | none |
| 460 | 2019 | 10.44072 | Yes | Alcohol wash | Yes | Oxalic Acid | Yes | Fondant or sugar candy, Pollen substitute, Prairie Pride Vitamins & Electrolytes | none |
| 461 | 2019 | 10.44072 | Yes | Alcohol wash | Yes | Oxalic Acid | Yes | Fondant or sugar candy, Pollen substitute, Prairie Pride Vitamins & Electrolytes | none |
| 462 | 2019 | 10.44072 | Yes | Alcohol wash | Yes | Oxalic Acid | Yes | Fondant or sugar candy, Pollen substitute, Prairie Pride Vitamins & Electrolytes | none |
| 463 | 2019 | 23.32732 | Yes | 48 hr drop (sticky board), Alcohol wash | Yes | Formic Acid (Mite Away Quick Strips) | Yes | Fondant or sugar candy, Pollen substitute | none |
| 464 | 2019 | 23.32732 | Yes | 48 hr drop (sticky board), Alcohol wash | Yes | Formic Acid (Mite Away Quick Strips) | Yes | Fondant or sugar candy, Pollen substitute | none |
| 465 | 2019 | 6.243675 | Yes | Sugar roll, Alcohol wash | Yes | Apivar (Amitraz) | Yes | Fondant or sugar candy | sugar only |
| 466 | 2019 | 11.9954 | Yes | Sugar roll | Yes | Oxalic Acid | Yes | Dry sugar | sugar only |
| 467 | 2019 | 11.9954 | Yes | Sugar roll | Yes | Oxalic Acid | Yes | Dry sugar | sugar only |
| 468 | 2019 | 11.9954 | Yes | Sugar roll | Yes | Oxalic Acid | Yes | Dry sugar | sugar only |
| 469 | 2019 | 11.9954 | Yes | Sugar roll | Yes | Oxalic Acid | Yes | Dry sugar | sugar only |
| 470 | 2019 | 11.9954 | Yes | Sugar roll | Yes | Oxalic Acid | Yes | Dry sugar | sugar only |
| 471 | 2019 | 11.9954 | Yes | Sugar roll | Yes | Oxalic Acid | Yes | Dry sugar | sugar only |
| 472 | 2019 | 1.033168 | Yes | 48 hr drop (sticky board) | Yes | Formic Acid (Mite Away Quick Strips) | Yes | Fondant or sugar candy, Sugar syrup | none |
| 473 | 2019 | 1.033168 | Yes | 48 hr drop (sticky board) | Yes | Formic Acid (Mite Away Quick Strips) | Yes | Fondant or sugar candy, Sugar syrup | none |
| 474 | 2019 | 1.033168 | Yes | 48 hr drop (sticky board) | Yes | Formic Acid (Mite Away Quick Strips) | Yes | Fondant or sugar candy, Sugar syrup | none |
| 475 | 2019 | 1.033168 | Yes | 48 hr drop (sticky board) | Yes | Formic Acid (Mite Away Quick Strips) | Yes | Fondant or sugar candy, Sugar syrup | none |
| 476 | 2019 | 1.033168 | Yes | 48 hr drop (sticky board) | Yes | Formic Acid (Mite Away Quick Strips) | Yes | Fondant or sugar candy, Sugar syrup | none |
| 477 | 2019 | 1.033168 | Yes | 48 hr drop (sticky board) | Yes | Formic Acid (Mite Away Quick Strips) | Yes | Fondant or sugar candy, Sugar syrup | none |
| 478 | 2019 | 1.033168 | Yes | 48 hr drop (sticky board) | Yes | Formic Acid (Mite Away Quick Strips) | Yes | Fondant or sugar candy, Sugar syrup | none |
| 479 | 2019 | 1.033168 | Yes | 48 hr drop (sticky board) | Yes | Formic Acid (Mite Away Quick Strips) | Yes | Fondant or sugar candy, Sugar syrup | none |
| 480 | 2019 | 1.033168 | Yes | 48 hr drop (sticky board) | Yes | Formic Acid (Mite Away Quick Strips) | Yes | Fondant or sugar candy, Sugar syrup | none |
| 481 | 2019 | 1.478571 | Yes | Sugar roll | Yes | Apivar (Amitraz) | Yes | Fondant or sugar candy | sugar only |
| 482 | 2019 | 1.478571 | Yes | Sugar roll | Yes | Apivar (Amitraz) | Yes | Fondant or sugar candy | sugar only |
| 483 | 2019 | 1.478571 | Yes | Sugar roll | Yes | Apivar (Amitraz) | Yes | Fondant or sugar candy | sugar only |
| 484 | 2019 | 9.447087 | Yes | 48 hr drop (sticky board) | Yes | Oxalic Acid | Yes | Fondant or sugar candy, Pollen substitute, Honey from your own stock | none |
| 485 | 2019 | 9.447087 | Yes | 48 hr drop (sticky board) | Yes | Oxalic Acid | Yes | Fondant or sugar candy, Pollen substitute, Honey from your own stock | none |
| 486 | 2019 | 0.718391 | Yes | Drone brood inspection | Yes | Formic Acid (Mite Away Quick Strips) | Yes | Fondant or sugar candy, Pollen substitute | none |
| 487 | 2019 | 0.718391 | Yes | Drone brood inspection | Yes | Formic Acid (Mite Away Quick Strips) | Yes | Fondant or sugar candy, Pollen substitute | none |
| 488 | 2019 | 0.718391 | Yes | Drone brood inspection | Yes | Formic Acid (Mite Away Quick Strips) | Yes | Fondant or sugar candy, Pollen substitute | none |
| 489 | 2019 | 0.718391 | Yes | Drone brood inspection | Yes | Formic Acid (Mite Away Quick Strips) | Yes | Fondant or sugar candy, Pollen substitute | none |
| 490 | 2019 | 0.718391 | Yes | Drone brood inspection | Yes | Formic Acid (Mite Away Quick Strips) | Yes | Fondant or sugar candy, Pollen substitute | none |
| 491 | 2019 | 0.718391 | Yes | Drone brood inspection | Yes | Formic Acid (Mite Away Quick Strips) | Yes | Fondant or sugar candy, Pollen substitute | none |
| 492 | 2019 | 0.718391 | Yes | Drone brood inspection | Yes | Formic Acid (Mite Away Quick Strips) | Yes | Fondant or sugar candy, Pollen substitute | none |
| 493 | 2019 | 12.17561 | No | NA | Yes | Formic Acid (Mite Away Quick Strips) | Yes | Fondant or sugar candy | sugar only |
| 494 | 2019 | 12.17561 | No | NA | Yes | Formic Acid (Mite Away Quick Strips) | Yes | Fondant or sugar candy | sugar only |
| 495 | 2019 | 14.64422 | No | NA | Yes | Oxalic Acid | Yes | Dry sugar | sugar only |
| 496 | 2019 | 14.64422 | No | NA | Yes | Oxalic Acid | Yes | Dry sugar | sugar only |
| 497 | 2019 | 14.64422 | No | NA | Yes | Oxalic Acid | Yes | Dry sugar | sugar only |
| 498 | 2019 | 14.64422 | No | NA | Yes | Oxalic Acid | Yes | Dry sugar | sugar only |
| 499 | 2019 | 14.64422 | No | NA | Yes | Oxalic Acid | Yes | Dry sugar | sugar only |
| 500 | 2019 | 5.384734 | Yes | 48 hr drop (sticky board) | Yes | Formic Acid (Mite Away Quick Strips) | Yes | Fondant or sugar candy | sugar only |
|  |  |  |  |  |  |  |  |  |  |
| 501 | 2019 | 5.384734 | Yes | 48 hr drop (sticky board) | Yes | Formic Acid (Mite Away Quick Strips) | Yes | Fondant or sugar candy | sugar only |
| 502 | 2019 | 5.384734 | Yes | 48 hr drop (sticky board) | Yes | Formic Acid (Mite Away Quick Strips) | Yes | Fondant or sugar candy | sugar only |
| 503 | 2019 | 31.79394 | Yes | Sugar roll | Yes | Oxalic Acid | Yes | Fondant or sugar candy | sugar only |
| 504 | 2019 | 31.79394 | Yes | Sugar roll | Yes | Oxalic Acid | Yes | Fondant or sugar candy | sugar only |
| 505 | 2019 | 11.58686 | Yes | Drone brood inspection | Yes | Formic Acid (Mite Away Quick Strips) | Yes | Dry sugar | sugar only |
| 506 | 2019 | 11.58686 | Yes | Drone brood inspection | Yes | Formic Acid (Mite Away Quick Strips) | Yes | Dry sugar | sugar only |
| 507 | 2019 | 5.222123 | Yes | Sugar roll | Yes | Oxalic Acid | Yes | Fondant or sugar candy, Honey from your own stock, Probiotics, Commercially available supplements | none |
| 508 | 2019 | 5.222123 | Yes | Sugar roll | Yes | Oxalic Acid | Yes | Fondant or sugar candy, Honey from your own stock, Probiotics, Commercially available supplements | none |
| 509 | 2019 | 5.222123 | Yes | Sugar roll | Yes | Oxalic Acid | Yes | Fondant or sugar candy, Honey from your own stock, Probiotics, Commercially available supplements | none |
| 510 | 2019 | 5.222123 | Yes | Sugar roll | Yes | Oxalic Acid | Yes | Fondant or sugar candy, Honey from your own stock, Probiotics, Commercially available supplements | none |
| 511 | 2019 | 5.222123 | Yes | Sugar roll | Yes | Oxalic Acid | Yes | Fondant or sugar candy, Honey from your own stock, Probiotics, Commercially available supplements | none |
| 512 | 2019 | 5.222123 | Yes | Sugar roll | Yes | Oxalic Acid | Yes | Fondant or sugar candy, Honey from your own stock, Probiotics, Commercially available supplements | none |
| 513 | 2019 | 5.222123 | Yes | Sugar roll | Yes | Oxalic Acid | Yes | Fondant or sugar candy, Honey from your own stock, Probiotics, Commercially available supplements | none |
| 514 | 2019 | 5.222123 | Yes | Sugar roll | Yes | Oxalic Acid | Yes | Fondant or sugar candy, Honey from your own stock, Probiotics, Commercially available supplements | none |
| 515 | 2019 | 41.53953 | Yes | 48 hr drop (sticky board) | Yes | Formic Acid (Mite Away Quick Strips) | Yes | Fondant or sugar candy | sugar only |
| 516 | 2019 | 41.53953 | Yes | 48 hr drop (sticky board) | Yes | Formic Acid (Mite Away Quick Strips) | Yes | Fondant or sugar candy | sugar only |
| 517 | 2019 | 41.53953 | Yes | 48 hr drop (sticky board) | Yes | Formic Acid (Mite Away Quick Strips) | Yes | Fondant or sugar candy | sugar only |
| 518 | 2019 | 41.53953 | Yes | 48 hr drop (sticky board) | Yes | Formic Acid (Mite Away Quick Strips) | Yes | Fondant or sugar candy | sugar only |
| 519 | 2019 | 41.53953 | Yes | 48 hr drop (sticky board) | Yes | Formic Acid (Mite Away Quick Strips) | Yes | Fondant or sugar candy | sugar only |
| 520 | 2019 | 41.53953 | Yes | 48 hr drop (sticky board) | Yes | Formic Acid (Mite Away Quick Strips) | Yes | Fondant or sugar candy | sugar only |
| 521 | 2019 | 41.53953 | Yes | 48 hr drop (sticky board) | Yes | Formic Acid (Mite Away Quick Strips) | Yes | Fondant or sugar candy | sugar only |
| 522 | 2019 | 41.53953 | Yes | 48 hr drop (sticky board) | Yes | Formic Acid (Mite Away Quick Strips) | Yes | Fondant or sugar candy | sugar only |
| 523 | 2019 | 41.53953 | Yes | 48 hr drop (sticky board) | Yes | Formic Acid (Mite Away Quick Strips) | Yes | Fondant or sugar candy | sugar only |
| 524 | 2019 | 41.53953 | Yes | 48 hr drop (sticky board) | Yes | Formic Acid (Mite Away Quick Strips) | Yes | Fondant or sugar candy | sugar only |
| 525 | 2019 | 3.036468 | Yes | Sugar roll | Yes | Formic Acid (Mite Away Quick Strips) | Yes | Fondant or sugar candy | sugar only |
| 526 | 2019 | 19.99567 | Yes | Sugar roll | Yes | Formic Acid (Mite Away Quick Strips) | Yes | Fondant or sugar candy | sugar only |
| 527 | 2019 | 13.02057 | Yes | Alcohol wash | Yes | Oxalic Acid | Yes | Fondant or sugar candy, Pollen substitute | none |
| 528 | 2019 | 13.02057 | Yes | Alcohol wash | Yes | Oxalic Acid | Yes | Fondant or sugar candy, Pollen substitute | none |
| 529 | 2019 | 14.06455 | Yes | Sugar roll | Yes | Apivar (Amitraz) | Yes | Fondant or sugar candy, Dry sugar, Pollen substitute | none |
| 530 | 2019 | 14.06455 | Yes | Sugar roll | Yes | Apivar (Amitraz) | Yes | Fondant or sugar candy, Dry sugar, Pollen substitute | none |
| 531 | 2019 | 14.06455 | Yes | Sugar roll | Yes | Apivar (Amitraz) | Yes | Fondant or sugar candy, Dry sugar, Pollen substitute | none |
| 532 | 2019 | 14.06455 | Yes | Sugar roll | Yes | Apivar (Amitraz) | Yes | Fondant or sugar candy, Dry sugar, Pollen substitute | none |
| 533 | 2019 | 14.06455 | Yes | Sugar roll | Yes | Apivar (Amitraz) | Yes | Fondant or sugar candy, Dry sugar, Pollen substitute | none |
| 534 | 2019 | 14.06455 | Yes | Sugar roll | Yes | Apivar (Amitraz) | Yes | Fondant or sugar candy, Dry sugar, Pollen substitute | none |
| 535 | 2019 | 14.06455 | Yes | Sugar roll | Yes | Apivar (Amitraz) | Yes | Fondant or sugar candy, Dry sugar, Pollen substitute | none |
| 536 | 2019 | 1.431989 | No | NA | Yes | Formic Acid (Mite Away Quick Strips) | Yes | Fondant or sugar candy, Pollen substitute | none |
| 537 | 2019 | 1.431989 | No | NA | Yes | Formic Acid (Mite Away Quick Strips) | Yes | Fondant or sugar candy, Pollen substitute | none |
| 538 | 2019 | 1.431989 | No | NA | Yes | Formic Acid (Mite Away Quick Strips) | Yes | Fondant or sugar candy, Pollen substitute | none |
| 539 | 2019 | 0.733377 | No | NA | Yes | Oxalic Acid | Yes | Fondant or sugar candy, Sugar syrup | none |
| 540 | 2019 | 0.733377 | No | NA | Yes | Oxalic Acid | Yes | Fondant or sugar candy, Sugar syrup | none |
| 541 | 2019 | 0.733377 | No | NA | Yes | Oxalic Acid | Yes | Fondant or sugar candy, Sugar syrup | none |
| 542 | 2019 | 0.733377 | No | NA | Yes | Oxalic Acid | Yes | Fondant or sugar candy, Sugar syrup | none |
| 543 | 2019 | 13.76371 | Yes | Sugar roll, Drone brood inspection | Yes | Apistan | Yes | Sugar syrup, Honey from your own stock | none |
| 544 | 2019 | 13.76371 | Yes | Sugar roll, Drone brood inspection | Yes | Apistan | Yes | Sugar syrup, Honey from your own stock | none |
| 545 | 2019 | 13.76371 | Yes | Sugar roll, Drone brood inspection | Yes | Apistan | Yes | Sugar syrup, Honey from your own stock | none |
| 546 | 2019 | 7.955956 | Yes | Alcohol wash | Yes | Oxalic Acid | Yes | Fondant or sugar candy, Pollen substitute | none |
| 547 | 2019 | 7.955956 | Yes | Alcohol wash | Yes | Oxalic Acid | Yes | Fondant or sugar candy, Pollen substitute | none |
| 548 | 2019 | 7.955956 | Yes | Alcohol wash | Yes | Oxalic Acid | Yes | Fondant or sugar candy, Pollen substitute | none |
| 549 | 2019 | 7.955956 | Yes | Alcohol wash | Yes | Oxalic Acid | Yes | Fondant or sugar candy, Pollen substitute | none |
| 550 | 2019 | 7.955956 | Yes | Alcohol wash | Yes | Oxalic Acid | Yes | Fondant or sugar candy, Pollen substitute | none |
| 551 | 2019 | 7.955956 | Yes | Alcohol wash | Yes | Oxalic Acid | Yes | Fondant or sugar candy, Pollen substitute | none |
| 552 | 2019 | 1.439742 | No | NA | Yes | Oxalic Acid | Yes | Fondant or sugar candy, Pollen substitute | none |
| 553 | 2019 | 1.439742 | No | NA | Yes | Oxalic Acid | Yes | Fondant or sugar candy, Pollen substitute | none |
| 554 | 2019 | 1.439742 | No | NA | Yes | Oxalic Acid | Yes | Fondant or sugar candy, Pollen substitute | none |
| 555 | 2019 | 1.439742 | No | NA | Yes | Oxalic Acid | Yes | Fondant or sugar candy, Pollen substitute | none |
| 556 | 2019 | 10.16854 | Yes | Alcohol wash | Yes | Formic Acid (Mite Away Quick Strips) | Yes | Fondant or sugar candy, Pollen substitute, Honey from your own stock | none |
| 557 | 2019 | 10.16854 | Yes | Alcohol wash | Yes | Formic Acid (Mite Away Quick Strips) | Yes | Fondant or sugar candy, Pollen substitute, Honey from your own stock | none |
| 558 | 2019 | 1.351224 | Yes | Sugar roll | Yes | Formic Acid (Mite Away Quick Strips) | Yes | Fondant or sugar candy | sugar only |
| 559 | 2019 | 1.351224 | Yes | Sugar roll | Yes | Formic Acid (Mite Away Quick Strips) | Yes | Fondant or sugar candy | sugar only |
| 560 | 2019 | 1.351224 | Yes | Sugar roll | Yes | Formic Acid (Mite Away Quick Strips) | Yes | Fondant or sugar candy | sugar only |
| 561 | 2019 | 1.351224 | Yes | Sugar roll | Yes | Formic Acid (Mite Away Quick Strips) | Yes | Fondant or sugar candy | sugar only |
| 562 | 2019 | 1.351224 | Yes | Sugar roll | Yes | Formic Acid (Mite Away Quick Strips) | Yes | Fondant or sugar candy | sugar only |
| 563 | 2019 | 1.431474 | Yes | 48 hr drop (sticky board), Sugar roll, Drone brood inspection | Yes | Api life Var | Yes | Fondant or sugar candy, Sugar syrup, Dry sugar, Honey from your own stock | none |
| 564 | 2019 | 1.431474 | Yes | 48 hr drop (sticky board), Sugar roll, Drone brood inspection | Yes | Api life Var | Yes | Fondant or sugar candy, Sugar syrup, Dry sugar, Honey from your own stock | none |
| 565 | 2019 | 1.431474 | Yes | 48 hr drop (sticky board), Sugar roll, Drone brood inspection | Yes | Api life Var | Yes | Fondant or sugar candy, Sugar syrup, Dry sugar, Honey from your own stock | none |
| 566 | 2019 | 1.431474 | Yes | 48 hr drop (sticky board), Sugar roll, Drone brood inspection | Yes | Api life Var | Yes | Fondant or sugar candy, Sugar syrup, Dry sugar, Honey from your own stock | none |
| 567 | 2019 | 1.431474 | Yes | 48 hr drop (sticky board), Sugar roll, Drone brood inspection | Yes | Api life Var | Yes | Fondant or sugar candy, Sugar syrup, Dry sugar, Honey from your own stock | none |
| 568 | 2019 | 1.431474 | Yes | 48 hr drop (sticky board), Sugar roll, Drone brood inspection | Yes | Api life Var | Yes | Fondant or sugar candy, Sugar syrup, Dry sugar, Honey from your own stock | none |
| 569 | 2019 | 1.431474 | Yes | 48 hr drop (sticky board), Sugar roll, Drone brood inspection | Yes | Api life Var | Yes | Fondant or sugar candy, Sugar syrup, Dry sugar, Honey from your own stock | none |
| 570 | 2019 | 1.431474 | Yes | 48 hr drop (sticky board), Sugar roll, Drone brood inspection | Yes | Api life Var | Yes | Fondant or sugar candy, Sugar syrup, Dry sugar, Honey from your own stock | none |
| 571 | 2019 | 1.142582 | Yes | Sugar roll | Yes | Oxalic Acid | Yes | Dry sugar | sugar only |
| 572 | 2019 | 1.142582 | Yes | Sugar roll | Yes | Oxalic Acid | Yes | Dry sugar | sugar only |
| 573 | 2019 | 1.142582 | Yes | Sugar roll | Yes | Oxalic Acid | Yes | Dry sugar | sugar only |
| 574 | 2019 | 1.142582 | Yes | Sugar roll | Yes | Oxalic Acid | Yes | Dry sugar | sugar only |
| 575 | 2019 | 1.142582 | Yes | Sugar roll | Yes | Oxalic Acid | Yes | Dry sugar | sugar only |
| 576 | 2019 | 8.403781 | Yes | Alcohol wash | Yes | Oxalic Acid | Yes | Honey from your own stock | none |
| 577 | 2019 | 8.403781 | Yes | Alcohol wash | Yes | Oxalic Acid | Yes | Honey from your own stock | none |
| 578 | 2019 | 17.98401 | No | 48 hr drop (sticky board) | Yes | Oxalic Acid | Yes | Fondant or sugar candy, Dry sugar, Pollen substitute | none |
| 579 | 2019 | 23.36679 | Yes | Monitor IPS Board after Oxalic Acid treatments | Yes | Oxalic Acid | Yes | Fondant or sugar candy, Pollen substitute | none |
| 580 | 2019 | 23.36679 | Yes | Monitor IPS Board after Oxalic Acid treatments | Yes | Oxalic Acid | Yes | Fondant or sugar candy, Pollen substitute | none |
| 581 | 2019 | 23.36679 | Yes | Monitor IPS Board after Oxalic Acid treatments | Yes | Oxalic Acid | Yes | Fondant or sugar candy, Pollen substitute | none |
| 582 | 2019 | 23.36679 | Yes | Monitor IPS Board after Oxalic Acid treatments | Yes | Oxalic Acid | Yes | Fondant or sugar candy, Pollen substitute | none |
| 583 | 2019 | 23.36679 | Yes | Monitor IPS Board after Oxalic Acid treatments | Yes | Oxalic Acid | Yes | Fondant or sugar candy, Pollen substitute | none |
| 584 | 2019 | 23.36679 | Yes | Monitor IPS Board after Oxalic Acid treatments | Yes | Oxalic Acid | Yes | Fondant or sugar candy, Pollen substitute | none |
| 585 | 2019 | 23.36679 | Yes | Monitor IPS Board after Oxalic Acid treatments | Yes | Oxalic Acid | Yes | Fondant or sugar candy, Pollen substitute | none |
| 586 | 2019 | 23.36679 | Yes | Monitor IPS Board after Oxalic Acid treatments | Yes | Oxalic Acid | Yes | Fondant or sugar candy, Pollen substitute | none |
| 587 | 2019 | 23.36679 | Yes | Monitor IPS Board after Oxalic Acid treatments | Yes | Oxalic Acid | Yes | Fondant or sugar candy, Pollen substitute | none |
| 588 | 2019 | 23.36679 | Yes | Monitor IPS Board after Oxalic Acid treatments | Yes | Oxalic Acid | Yes | Fondant or sugar candy, Pollen substitute | none |
| 589 | 2019 | 23.36679 | Yes | Monitor IPS Board after Oxalic Acid treatments | Yes | Oxalic Acid | Yes | Fondant or sugar candy, Pollen substitute | none |
| 590 | 2019 | 23.36679 | Yes | Monitor IPS Board after Oxalic Acid treatments | Yes | Oxalic Acid | Yes | Fondant or sugar candy, Pollen substitute | none |
| 591 | 2019 | 23.36679 | Yes | Monitor IPS Board after Oxalic Acid treatments | Yes | Oxalic Acid | Yes | Fondant or sugar candy, Pollen substitute | none |
| 592 | 2019 | 23.36679 | Yes | Monitor IPS Board after Oxalic Acid treatments | Yes | Oxalic Acid | Yes | Fondant or sugar candy, Pollen substitute | none |
| 593 | 2019 | 1.657663 | Yes | 48 hr drop (sticky board) | Yes | Apivar (Amitraz) | Yes | Dry sugar | sugar only |
| 594 | 2019 | 30.79471 | Yes | 48 hr drop (sticky board), Alcohol wash | Yes | Oxalic Acid | Yes | Dry sugar | sugar only |
| 595 | 2019 | 30.79471 | Yes | 48 hr drop (sticky board), Alcohol wash | Yes | Oxalic Acid | Yes | Dry sugar | sugar only |
| 596 | 2019 | 30.79471 | Yes | 48 hr drop (sticky board), Alcohol wash | Yes | Oxalic Acid | Yes | Dry sugar | sugar only |
| 597 | 2019 | 30.79471 | Yes | 48 hr drop (sticky board), Alcohol wash | Yes | Oxalic Acid | Yes | Dry sugar | sugar only |
| 598 | 2019 | 30.79471 | Yes | 48 hr drop (sticky board), Alcohol wash | Yes | Oxalic Acid | Yes | Dry sugar | sugar only |
| 599 | 2019 | 30.79471 | Yes | 48 hr drop (sticky board), Alcohol wash | Yes | Oxalic Acid | Yes | Dry sugar | sugar only |
| 600 | 2019 | 30.79471 | Yes | 48 hr drop (sticky board), Alcohol wash | Yes | Oxalic Acid | Yes | Dry sugar | sugar only |
| 601 | 2019 | 30.79471 | Yes | 48 hr drop (sticky board), Alcohol wash | Yes | Oxalic Acid | Yes | Dry sugar | sugar only |
| 602 | 2019 | 30.79471 | Yes | 48 hr drop (sticky board), Alcohol wash | Yes | Oxalic Acid | Yes | Dry sugar | sugar only |
| 603 | 2019 | 1.667334 | Yes | 48 hr drop (sticky board) | Yes | Oxalic Acid | Yes | Fondant or sugar candy | sugar only |
| 604 | 2019 | 1.667334 | Yes | 48 hr drop (sticky board) | Yes | Oxalic Acid | Yes | Fondant or sugar candy | sugar only |
| 605 | 2019 | 1.667334 | Yes | 48 hr drop (sticky board) | Yes | Oxalic Acid | Yes | Fondant or sugar candy | sugar only |
| 606 | 2019 | 42.24082 | No | NA | Yes | Formic Acid (Mite Away Quick Strips) | Yes | Fondant or sugar candy | sugar only |
| 607 | 2019 | 42.24082 | No | NA | Yes | Formic Acid (Mite Away Quick Strips) | Yes | Fondant or sugar candy | sugar only |
| 608 | 2019 | 42.24082 | No | NA | Yes | Formic Acid (Mite Away Quick Strips) | Yes | Fondant or sugar candy | sugar only |
| 609 | 2019 | 42.24082 | No | NA | Yes | Formic Acid (Mite Away Quick Strips) | Yes | Fondant or sugar candy | sugar only |
| 610 | 2019 | 42.24082 | No | NA | Yes | Formic Acid (Mite Away Quick Strips) | Yes | Fondant or sugar candy | sugar only |
| 611 | 2019 | 42.24082 | No | NA | Yes | Formic Acid (Mite Away Quick Strips) | Yes | Fondant or sugar candy | sugar only |
| 612 | 2019 | 42.24082 | No | NA | Yes | Formic Acid (Mite Away Quick Strips) | Yes | Fondant or sugar candy | sugar only |
| 613 | 2019 | 2.587276 | Yes | Sugar roll | Yes | Api life Var | Yes | Fondant or sugar candy, Honey from your own stock | none |
| 614 | 2019 | 0.780273 | No | NA | Yes | Apivar (Amitraz) | Yes | Dry sugar | sugar only |
| 615 | 2019 | 0.780273 | No | NA | Yes | Apivar (Amitraz) | Yes | Dry sugar | sugar only |
| 616 | 2019 | 19.46553 | Yes | 48 hr drop (sticky board) | Yes | Oxalic Acid | Yes | Fondant or sugar candy, Pollen substitute | none |
| 617 | 2019 | 19.46553 | Yes | 48 hr drop (sticky board) | Yes | Oxalic Acid | Yes | Fondant or sugar candy, Pollen substitute | none |
| 618 | 2019 | 19.46553 | Yes | 48 hr drop (sticky board) | Yes | Oxalic Acid | Yes | Fondant or sugar candy, Pollen substitute | none |
| 619 | 2019 | 19.46553 | Yes | 48 hr drop (sticky board) | Yes | Oxalic Acid | Yes | Fondant or sugar candy, Pollen substitute | none |
| 620 | 2019 | 2.542764 | No | NA | Yes | Oxalic Acid | Yes | Fondant or sugar candy | sugar only |
| 621 | 2019 | 9.852422 | Yes | 48 hr drop (sticky board), Sugar roll | Yes | Formic Acid (Mite Away Quick Strips) | Yes | Fondant or sugar candy, Sugar syrup, Pollen substitute, Commercially available supplements | none |
| 622 | 2019 | 9.852422 | Yes | 48 hr drop (sticky board), Sugar roll | Yes | Formic Acid (Mite Away Quick Strips) | Yes | Fondant or sugar candy, Sugar syrup, Pollen substitute, Commercially available supplements | none |
| 623 | 2019 | 9.852422 | Yes | 48 hr drop (sticky board), Sugar roll | Yes | Formic Acid (Mite Away Quick Strips) | Yes | Fondant or sugar candy, Sugar syrup, Pollen substitute, Commercially available supplements | none |
| 624 | 2019 | 9.852422 | Yes | 48 hr drop (sticky board), Sugar roll | Yes | Formic Acid (Mite Away Quick Strips) | Yes | Fondant or sugar candy, Sugar syrup, Pollen substitute, Commercially available supplements | none |
| 625 | 2019 | 9.852422 | Yes | 48 hr drop (sticky board), Sugar roll | Yes | Formic Acid (Mite Away Quick Strips) | Yes | Fondant or sugar candy, Sugar syrup, Pollen substitute, Commercially available supplements | none |
| 626 | 2019 | 5.614225 | Yes | 48 hr drop (sticky board) | Yes | powdered sugar | Yes | Fondant or sugar candy | sugar only |
| 627 | 2019 | 7.603631 | Yes | count dead mites on the pull out board | Yes | Formic Acid (Mite Away Quick Strips) | Yes | Dry sugar | sugar only |
| 628 | 2019 | 7.603631 | Yes | count dead mites on the pull out board | Yes | Formic Acid (Mite Away Quick Strips) | Yes | Dry sugar | sugar only |
| 629 | 2019 | 7.603631 | Yes | count dead mites on the pull out board | Yes | Formic Acid (Mite Away Quick Strips) | Yes | Dry sugar | sugar only |
| 630 | 2019 | 7.603631 | Yes | count dead mites on the pull out board | Yes | Formic Acid (Mite Away Quick Strips) | Yes | Dry sugar | sugar only |
| 631 | 2019 | 7.603631 | Yes | count dead mites on the pull out board | Yes | Formic Acid (Mite Away Quick Strips) | Yes | Dry sugar | sugar only |
| 632 | 2019 | 7.603631 | Yes | count dead mites on the pull out board | Yes | Formic Acid (Mite Away Quick Strips) | Yes | Dry sugar | sugar only |
| 633 | 2019 | 7.603631 | Yes | count dead mites on the pull out board | Yes | Formic Acid (Mite Away Quick Strips) | Yes | Dry sugar | sugar only |
| 634 | 2019 | 7.603631 | Yes | count dead mites on the pull out board | Yes | Formic Acid (Mite Away Quick Strips) | Yes | Dry sugar | sugar only |
| 635 | 2019 | 7.603631 | Yes | count dead mites on the pull out board | Yes | Formic Acid (Mite Away Quick Strips) | Yes | Dry sugar | sugar only |
| 636 | 2019 | 23.63939 | Yes | 48 hr drop (sticky board), Drone brood inspection | Yes | Formic Acid (Mite Away Quick Strips) | Yes | Sugar syrup | none |
| 637 | 2019 | 23.63939 | Yes | 48 hr drop (sticky board), Drone brood inspection | Yes | Formic Acid (Mite Away Quick Strips) | Yes | Sugar syrup | none |
| 638 | 2019 | 23.63939 | Yes | 48 hr drop (sticky board), Drone brood inspection | Yes | Formic Acid (Mite Away Quick Strips) | Yes | Sugar syrup | none |
| 639 | 2019 | 23.63939 | Yes | 48 hr drop (sticky board), Drone brood inspection | Yes | Formic Acid (Mite Away Quick Strips) | Yes | Sugar syrup | none |
| 640 | 2019 | 23.63939 | Yes | 48 hr drop (sticky board), Drone brood inspection | Yes | Formic Acid (Mite Away Quick Strips) | Yes | Sugar syrup | none |
| 641 | 2019 | 7.290194 | Yes | Drone brood inspection | Yes | Oxalic Acid | Yes | Sugar syrup, Commercially available supplements | none |
| 642 | 2019 | 7.290194 | Yes | Drone brood inspection | Yes | Oxalic Acid | Yes | Sugar syrup, Commercially available supplements | none |
| 643 | 2019 | 7.290194 | Yes | Drone brood inspection | Yes | Oxalic Acid | Yes | Sugar syrup, Commercially available supplements | none |
| 644 | 2019 | 7.290194 | Yes | Drone brood inspection | Yes | Oxalic Acid | Yes | Sugar syrup, Commercially available supplements | none |
| 645 | 2019 | 7.290194 | Yes | Drone brood inspection | Yes | Oxalic Acid | Yes | Sugar syrup, Commercially available supplements | none |
| 646 | 2019 | 7.290194 | Yes | Drone brood inspection | Yes | Oxalic Acid | Yes | Sugar syrup, Commercially available supplements | none |
| 647 | 2019 | 7.290194 | Yes | Drone brood inspection | Yes | Oxalic Acid | Yes | Sugar syrup, Commercially available supplements | none |
| 648 | 2019 | 7.290194 | Yes | Drone brood inspection | Yes | Oxalic Acid | Yes | Sugar syrup, Commercially available supplements | none |
| 649 | 2019 | 7.290194 | Yes | Drone brood inspection | Yes | Oxalic Acid | Yes | Sugar syrup, Commercially available supplements | none |
| 650 | 2019 | 7.290194 | Yes | Drone brood inspection | Yes | Oxalic Acid | Yes | Sugar syrup, Commercially available supplements | none |
| 651 | 2019 | 7.290194 | Yes | Drone brood inspection | Yes | Oxalic Acid | Yes | Sugar syrup, Commercially available supplements | none |
| 652 | 2019 | 7.290194 | Yes | Drone brood inspection | Yes | Oxalic Acid | Yes | Sugar syrup, Commercially available supplements | none |
| 653 | 2019 | 2.566873 | Yes | Sugar roll | Yes | Api life Var | Yes | Sugar syrup | none |
| 654 | 2019 | 3.943314 | Yes | Sugar roll | Yes | Apivar (Amitraz) | Yes | Fondant or sugar candy | sugar only |
| 655 | 2019 | 3.943314 | Yes | Sugar roll | Yes | Apivar (Amitraz) | Yes | Fondant or sugar candy | sugar only |
| 656 | 2019 | 3.943314 | Yes | Sugar roll | Yes | Apivar (Amitraz) | Yes | Fondant or sugar candy | sugar only |
| 657 | 2019 | 3.943314 | Yes | Sugar roll | Yes | Apivar (Amitraz) | Yes | Fondant or sugar candy | sugar only |
| 658 | 2019 | 3.943314 | Yes | Sugar roll | Yes | Apivar (Amitraz) | Yes | Fondant or sugar candy | sugar only |
| 659 | 2019 | 6.155605 | Yes | Alcohol wash | Yes | Oxalic Acid | Yes | Sugar syrup | none |
| 660 | 2019 | 6.155605 | Yes | Alcohol wash | Yes | Oxalic Acid | Yes | Sugar syrup | none |
| 661 | 2019 | 6.155605 | Yes | Alcohol wash | Yes | Oxalic Acid | Yes | Sugar syrup | none |
| 662 | 2019 | 18.19132 | Yes | Alcohol wash, Drone brood inspection | Yes | Apivar (Amitraz) | Yes | Dry sugar | sugar only |
| 663 | 2019 | 18.19132 | Yes | Alcohol wash, Drone brood inspection | Yes | Apivar (Amitraz) | Yes | Dry sugar | sugar only |
| 664 | 2019 | 41.50993 | No | NA | Yes | Formic Acid (Mite Away Quick Strips) | Yes | Fondant or sugar candy | sugar only |
| 665 | 2019 | 41.50993 | No | NA | Yes | Formic Acid (Mite Away Quick Strips) | Yes | Fondant or sugar candy | sugar only |
| 666 | 2019 | 2.137471 | Yes | Drone brood inspection | Yes | Oxalic Acid | Yes | Fondant or sugar candy | sugar only |
| 667 | 2019 | 2.137471 | Yes | Drone brood inspection | Yes | Oxalic Acid | Yes | Fondant or sugar candy | sugar only |
| 668 | 2019 | 10.48784 | Yes | 48 hr drop (sticky board) | Yes | Formic Acid (Mite Away Quick Strips) | Yes | Fondant or sugar candy | sugar only |
| 669 | 2019 | 2.018856 | Yes | Sugar roll, Alcohol wash, Drone brood inspection | Yes | Formic Acid (Mite Away Quick Strips) | Yes | Dry sugar | sugar only |
| 670 | 2019 | 2.018856 | Yes | Sugar roll, Alcohol wash, Drone brood inspection | Yes | Formic Acid (Mite Away Quick Strips) | Yes | Dry sugar | sugar only |
| 671 | 2019 | 2.018856 | Yes | Sugar roll, Alcohol wash, Drone brood inspection | Yes | Formic Acid (Mite Away Quick Strips) | Yes | Dry sugar | sugar only |
| 672 | 2019 | 2.018856 | Yes | Sugar roll, Alcohol wash, Drone brood inspection | Yes | Formic Acid (Mite Away Quick Strips) | Yes | Dry sugar | sugar only |
| 673 | 2019 | 2.018856 | Yes | Sugar roll, Alcohol wash, Drone brood inspection | Yes | Formic Acid (Mite Away Quick Strips) | Yes | Dry sugar | sugar only |
| 674 | 2019 | 2.018856 | Yes | Sugar roll, Alcohol wash, Drone brood inspection | Yes | Formic Acid (Mite Away Quick Strips) | Yes | Dry sugar | sugar only |
| 675 | 2019 | 2.018856 | Yes | Sugar roll, Alcohol wash, Drone brood inspection | Yes | Formic Acid (Mite Away Quick Strips) | Yes | Dry sugar | sugar only |
| 676 | 2019 | 2.018856 | Yes | Sugar roll, Alcohol wash, Drone brood inspection | Yes | Formic Acid (Mite Away Quick Strips) | Yes | Dry sugar | sugar only |
| 677 | 2019 | 8.718992 | Yes | 48 hr drop (sticky board), Drone brood inspection | Yes | Formic Acid (Mite Away Quick Strips) | Yes | Fondant or sugar candy | sugar only |
| 678 | 2019 | 4.064103 | No | NA | Yes | Oxalic Acid | Yes | Fondant or sugar candy | sugar only |
| 679 | 2019 | 4.064103 | No | NA | Yes | Oxalic Acid | Yes | Fondant or sugar candy | sugar only |
| 680 | 2019 | 4.064103 | No | NA | Yes | Oxalic Acid | Yes | Fondant or sugar candy | sugar only |
| 681 | 2019 | 4.064103 | No | NA | Yes | Oxalic Acid | Yes | Fondant or sugar candy | sugar only |
| 682 | 2019 | 4.064103 | No | NA | Yes | Oxalic Acid | Yes | Fondant or sugar candy | sugar only |
| 683 | 2019 | 4.064103 | No | NA | Yes | Oxalic Acid | Yes | Fondant or sugar candy | sugar only |
| 684 | 2019 | 4.064103 | No | NA | Yes | Oxalic Acid | Yes | Fondant or sugar candy | sugar only |
| 685 | 2019 | 4.064103 | No | NA | Yes | Oxalic Acid | Yes | Fondant or sugar candy | sugar only |
| 686 | 2019 | 4.064103 | No | NA | Yes | Oxalic Acid | Yes | Fondant or sugar candy | sugar only |
| 687 | 2019 | 4.064103 | No | NA | Yes | Oxalic Acid | Yes | Fondant or sugar candy | sugar only |
| 688 | 2019 | 4.064103 | No | NA | Yes | Oxalic Acid | Yes | Fondant or sugar candy | sugar only |
| 689 | 2019 | 4.064103 | No | NA | Yes | Oxalic Acid | Yes | Fondant or sugar candy | sugar only |
| 690 | 2019 | 4.064103 | No | NA | Yes | Oxalic Acid | Yes | Fondant or sugar candy | sugar only |
| 691 | 2019 | 4.064103 | No | NA | Yes | Oxalic Acid | Yes | Fondant or sugar candy | sugar only |
| 692 | 2019 | 4.064103 | No | NA | Yes | Oxalic Acid | Yes | Fondant or sugar candy | sugar only |
| 693 | 2019 | 4.064103 | No | NA | Yes | Oxalic Acid | Yes | Fondant or sugar candy | sugar only |
| 694 | 2019 | 4.064103 | No | NA | Yes | Oxalic Acid | Yes | Fondant or sugar candy | sugar only |
| 695 | 2019 | 4.064103 | No | NA | Yes | Oxalic Acid | Yes | Fondant or sugar candy | sugar only |
| 696 | 2019 | 4.064103 | No | NA | Yes | Oxalic Acid | Yes | Fondant or sugar candy | sugar only |
| 697 | 2019 | 4.064103 | No | NA | Yes | Oxalic Acid | Yes | Fondant or sugar candy | sugar only |
| 698 | 2019 | 4.064103 | No | NA | Yes | Oxalic Acid | Yes | Fondant or sugar candy | sugar only |
| 699 | 2019 | 4.064103 | No | NA | Yes | Oxalic Acid | Yes | Fondant or sugar candy | sugar only |
| 700 | 2019 | 4.064103 | No | NA | Yes | Oxalic Acid | Yes | Fondant or sugar candy | sugar only |
| 701 | 2019 | 4.064103 | No | NA | Yes | Oxalic Acid | Yes | Fondant or sugar candy | sugar only |
| 702 | 2019 | 21.48214 | Yes | NA | Yes | Formic Acid (Mite Away Quick Strips) | Yes | Fondant or sugar candy | sugar only |
| 703 | 2019 | 21.48214 | Yes | NA | Yes | Formic Acid (Mite Away Quick Strips) | Yes | Fondant or sugar candy | sugar only |
| 704 | 2019 | 21.48214 | Yes | NA | Yes | Formic Acid (Mite Away Quick Strips) | Yes | Fondant or sugar candy | sugar only |
| 705 | 2019 | 21.48214 | Yes | NA | Yes | Formic Acid (Mite Away Quick Strips) | Yes | Fondant or sugar candy | sugar only |
| 706 | 2019 | 1.11176 | Yes | Sugar roll | Yes | Oxalic Acid | Yes | Honey from your own stock | none |
| 707 | 2019 | 1.11176 | Yes | Sugar roll | Yes | Oxalic Acid | Yes | Honey from your own stock | none |
| 708 | 2019 | 1.11176 | Yes | Sugar roll | Yes | Oxalic Acid | Yes | Honey from your own stock | none |
| 709 | 2019 | 1.11176 | Yes | Sugar roll | Yes | Oxalic Acid | Yes | Honey from your own stock | none |
| 710 | 2019 | 2.160808 | Yes | 48 hr drop (sticky board), Sugar roll, Drone brood inspection | Yes | Oxalic Acid | Yes | Fondant or sugar candy | sugar only |
| 711 | 2019 | 2.160808 | Yes | 48 hr drop (sticky board), Sugar roll, Drone brood inspection | Yes | Oxalic Acid | Yes | Fondant or sugar candy | sugar only |
| 712 | 2019 | 2.160808 | Yes | 48 hr drop (sticky board), Sugar roll, Drone brood inspection | Yes | Oxalic Acid | Yes | Fondant or sugar candy | sugar only |
| 713 | 2019 | 2.160808 | Yes | 48 hr drop (sticky board), Sugar roll, Drone brood inspection | Yes | Oxalic Acid | Yes | Fondant or sugar candy | sugar only |
| 714 | 2019 | 2.160808 | Yes | 48 hr drop (sticky board), Sugar roll, Drone brood inspection | Yes | Oxalic Acid | Yes | Fondant or sugar candy | sugar only |
| 715 | 2019 | 1.077808 | Yes | Drone brood inspection | Yes | Apistan | Yes | Fondant or sugar candy, Sugar syrup, Pollen substitute | none |
| 716 | 2019 | 1.077808 | Yes | Drone brood inspection | Yes | Apistan | Yes | Fondant or sugar candy, Sugar syrup, Pollen substitute | none |
| 717 | 2019 | 4.125677 | Yes | visual, but need to start using a better method | Yes | Formic Acid (Mite Away Quick Strips) | Yes | Fondant or sugar candy | sugar only |
| 718 | 2019 | 4.125677 | Yes | visual, but need to start using a better method | Yes | Formic Acid (Mite Away Quick Strips) | Yes | Fondant or sugar candy | sugar only |
| 719 | 2019 | 4.125677 | Yes | visual, but need to start using a better method | Yes | Formic Acid (Mite Away Quick Strips) | Yes | Fondant or sugar candy | sugar only |
| 720 | 2019 | 1.466242 | Yes | Sugar roll | Yes | Apivar (Amitraz) | Yes | Fondant or sugar candy, Pollen substitute | none |
| 721 | 2019 | 1.466242 | Yes | Sugar roll | Yes | Apivar (Amitraz) | Yes | Fondant or sugar candy, Pollen substitute | none |
| 722 | 2019 | 2.276362 | Yes | Alcohol wash | Yes | Formic Acid (Mite Away Quick Strips) | Yes | Fondant or sugar candy, Dry sugar, Pollen substitute | none |
| 723 | 2019 | 2.276362 | Yes | Alcohol wash | Yes | Formic Acid (Mite Away Quick Strips) | Yes | Fondant or sugar candy, Dry sugar, Pollen substitute | none |
| 724 | 2019 | 2.276362 | Yes | Alcohol wash | Yes | Formic Acid (Mite Away Quick Strips) | Yes | Fondant or sugar candy, Dry sugar, Pollen substitute | none |
| 725 | 2019 | 2.276362 | Yes | Alcohol wash | Yes | Formic Acid (Mite Away Quick Strips) | Yes | Fondant or sugar candy, Dry sugar, Pollen substitute | none |
| 726 | 2019 | 2.276362 | Yes | Alcohol wash | Yes | Formic Acid (Mite Away Quick Strips) | Yes | Fondant or sugar candy, Dry sugar, Pollen substitute | none |
| 727 | 2019 | 2.276362 | Yes | Alcohol wash | Yes | Formic Acid (Mite Away Quick Strips) | Yes | Fondant or sugar candy, Dry sugar, Pollen substitute | none |
| 728 | 2019 | 2.276362 | Yes | Alcohol wash | Yes | Formic Acid (Mite Away Quick Strips) | Yes | Fondant or sugar candy, Dry sugar, Pollen substitute | none |
| 729 | 2019 | 2.276362 | Yes | Alcohol wash | Yes | Formic Acid (Mite Away Quick Strips) | Yes | Fondant or sugar candy, Dry sugar, Pollen substitute | none |
| 730 | 2019 | 17.94177 | Yes | Sugar roll | Yes | Apivar (Amitraz) | Yes | Fondant or sugar candy, Pollen substitute, Honey from your own stock | none |
| 731 | 2019 | 17.94177 | Yes | Sugar roll | Yes | Apivar (Amitraz) | Yes | Fondant or sugar candy, Pollen substitute, Honey from your own stock | none |
| 732 | 2019 | 17.94177 | Yes | Sugar roll | Yes | Apivar (Amitraz) | Yes | Fondant or sugar candy, Pollen substitute, Honey from your own stock | none |
| 733 | 2019 | 3.250251 | Yes | NA | Yes | Apiguard (thymol) | Yes | Honey from your own stock | none |
| 734 | 2019 | 3.250251 | Yes | NA | Yes | Apiguard (thymol) | Yes | Honey from your own stock | none |
| 735 | 2019 | 3.873344 | Yes | Alcohol wash | Yes | Formic Acid (Mite Away Quick Strips) | Yes | Fondant or sugar candy | sugar only |
| 736 | 2019 | 3.873344 | Yes | Alcohol wash | Yes | Formic Acid (Mite Away Quick Strips) | Yes | Fondant or sugar candy | sugar only |
| 737 | 2019 | 3.873344 | Yes | Alcohol wash | Yes | Formic Acid (Mite Away Quick Strips) | Yes | Fondant or sugar candy | sugar only |
| 738 | 2019 | 3.873344 | Yes | Alcohol wash | Yes | Formic Acid (Mite Away Quick Strips) | Yes | Fondant or sugar candy | sugar only |
| 739 | 2019 | 3.873344 | Yes | Alcohol wash | Yes | Formic Acid (Mite Away Quick Strips) | Yes | Fondant or sugar candy | sugar only |
| 740 | 2019 | 3.873344 | Yes | Alcohol wash | Yes | Formic Acid (Mite Away Quick Strips) | Yes | Fondant or sugar candy | sugar only |
| 741 | 2019 | 3.873344 | Yes | Alcohol wash | Yes | Formic Acid (Mite Away Quick Strips) | Yes | Fondant or sugar candy | sugar only |
| 742 | 2019 | 3.873344 | Yes | Alcohol wash | Yes | Formic Acid (Mite Away Quick Strips) | Yes | Fondant or sugar candy | sugar only |
| 743 | 2019 | 3.873344 | Yes | Alcohol wash | Yes | Formic Acid (Mite Away Quick Strips) | Yes | Fondant or sugar candy | sugar only |
| 744 | 2019 | 3.873344 | Yes | Alcohol wash | Yes | Formic Acid (Mite Away Quick Strips) | Yes | Fondant or sugar candy | sugar only |
| 745 | 2019 | 3.873344 | Yes | Alcohol wash | Yes | Formic Acid (Mite Away Quick Strips) | Yes | Fondant or sugar candy | sugar only |
| 746 | 2019 | 5.575694 | Yes | Alcohol wash | Yes | Api life Var | Yes | Dry sugar, Pollen substitute | none |
| 747 | 2019 | 5.575694 | Yes | Alcohol wash | Yes | Api life Var | Yes | Dry sugar, Pollen substitute | none |
| 748 | 2019 | 5.575694 | Yes | Alcohol wash | Yes | Api life Var | Yes | Dry sugar, Pollen substitute | none |
| 749 | 2019 | 5.575694 | Yes | Alcohol wash | Yes | Api life Var | Yes | Dry sugar, Pollen substitute | none |
| 750 | 2019 | 5.575694 | Yes | Alcohol wash | Yes | Api life Var | Yes | Dry sugar, Pollen substitute | none |
| 751 | 2019 | 5.575694 | Yes | Alcohol wash | Yes | Api life Var | Yes | Dry sugar, Pollen substitute | none |
| 752 | 2019 | 5.575694 | Yes | Alcohol wash | Yes | Api life Var | Yes | Dry sugar, Pollen substitute | none |
| 753 | 2019 | 30.19332 | Yes | 48 hr drop (sticky board) | Yes | Apivar (Amitraz) | Yes | Pollen substitute, Honey from your own stock | none |
| 754 | 2019 | 30.19332 | Yes | 48 hr drop (sticky board) | Yes | Apivar (Amitraz) | Yes | Pollen substitute, Honey from your own stock | none |
| 755 | 2019 | 30.19332 | Yes | 48 hr drop (sticky board) | Yes | Apivar (Amitraz) | Yes | Pollen substitute, Honey from your own stock | none |
| 756 | 2019 | 12.6242 | Yes | Sugar roll | Yes | Oxalic Acid | Yes | Fondant or sugar candy, Pollen substitute | none |
| 757 | 2019 | 31.24304 | Yes | 48 hr drop (sticky board), Sugar roll | Yes | Hopguard | Yes | Honey from your own stock, winter patties | none |
| 758 | 2019 | 31.24304 | Yes | 48 hr drop (sticky board), Sugar roll | Yes | Hopguard | Yes | Honey from your own stock, winter patties | none |
| 759 | 2019 | 31.24304 | Yes | 48 hr drop (sticky board), Sugar roll | Yes | Hopguard | Yes | Honey from your own stock, winter patties | none |
| 760 | 2019 | 0.822144 | No | NA | Yes | Mite away | Yes | Fondant or sugar candy, Sugar syrup | none |
| 761 | 2019 | 0.822144 | No | NA | Yes | Mite away | Yes | Fondant or sugar candy, Sugar syrup | none |
| 762 | 2019 | 0.822144 | No | NA | Yes | Mite away | Yes | Fondant or sugar candy, Sugar syrup | none |
| 763 | 2019 | 0.822144 | No | NA | Yes | Mite away | Yes | Fondant or sugar candy, Sugar syrup | none |
| 764 | 2019 | 34.57663 | No | NA | Yes | Oxalic Acid | Yes | Fondant or sugar candy, Dry sugar, pollen patties | none |
| 765 | 2019 | 3.419697 | Yes | Sugar roll | Yes | Formic Acid (Mite Away Quick Strips) | Yes | Sugar syrup | none |
| 766 | 2019 | 3.419697 | Yes | Sugar roll | Yes | Formic Acid (Mite Away Quick Strips) | Yes | Sugar syrup | none |
| 767 | 2019 | 57.89965 | Yes | Sugar roll | Yes | Oxalic Acid | Yes | Fondant or sugar candy, Honey from your own stock | none |
| 768 | 2019 | 21.9357 | Yes | Sugar roll | Yes | Formic Acid (Mite Away Quick Strips) | Yes | Dry sugar, Homemade "Honey-Bee Healthy" Essential Oil Combination | none |
| 769 | 2019 | 21.9357 | Yes | Sugar roll | Yes | Formic Acid (Mite Away Quick Strips) | Yes | Dry sugar, Homemade "Honey-Bee Healthy" Essential Oil Combination | none |
| 770 | 2019 | 21.9357 | Yes | Sugar roll | Yes | Formic Acid (Mite Away Quick Strips) | Yes | Dry sugar, Homemade "Honey-Bee Healthy" Essential Oil Combination | none |
| 771 | 2019 | 1.224393 | Yes | Alcohol wash | Yes | Apivar (Amitraz) | Yes | Fondant or sugar candy | sugar only |
| 772 | 2019 | 1.224393 | Yes | Alcohol wash | Yes | Apivar (Amitraz) | Yes | Fondant or sugar candy | sugar only |
| 773 | 2019 | 2.881591 | Yes | Drone brood inspection | Yes | Formic Acid (Mite Away Quick Strips) | Yes | Honey from your own stock, Probiotics, Commercially available supplements | none |
| 774 | 2019 | 2.881591 | Yes | Drone brood inspection | Yes | Formic Acid (Mite Away Quick Strips) | Yes | Honey from your own stock, Probiotics, Commercially available supplements | none |
| 775 | 2019 | 2.881591 | Yes | Drone brood inspection | Yes | Formic Acid (Mite Away Quick Strips) | Yes | Honey from your own stock, Probiotics, Commercially available supplements | none |
| 776 | 2019 | 2.881591 | Yes | Drone brood inspection | Yes | Formic Acid (Mite Away Quick Strips) | Yes | Honey from your own stock, Probiotics, Commercially available supplements | none |
| 777 | 2019 | 2.881591 | Yes | Drone brood inspection | Yes | Formic Acid (Mite Away Quick Strips) | Yes | Honey from your own stock, Probiotics, Commercially available supplements | none |
| 778 | 2019 | 2.881591 | Yes | Drone brood inspection | Yes | Formic Acid (Mite Away Quick Strips) | Yes | Honey from your own stock, Probiotics, Commercially available supplements | none |
| 779 | 2019 | 2.881591 | Yes | Drone brood inspection | Yes | Formic Acid (Mite Away Quick Strips) | Yes | Honey from your own stock, Probiotics, Commercially available supplements | none |
| 780 | 2019 | 2.881591 | Yes | Drone brood inspection | Yes | Formic Acid (Mite Away Quick Strips) | Yes | Honey from your own stock, Probiotics, Commercially available supplements | none |
| 781 | 2019 | 2.881591 | Yes | Drone brood inspection | Yes | Formic Acid (Mite Away Quick Strips) | Yes | Honey from your own stock, Probiotics, Commercially available supplements | none |
| 782 | 2019 | 2.881591 | Yes | Drone brood inspection | Yes | Formic Acid (Mite Away Quick Strips) | Yes | Honey from your own stock, Probiotics, Commercially available supplements | none |
| 783 | 2019 | 2.881591 | Yes | Drone brood inspection | Yes | Formic Acid (Mite Away Quick Strips) | Yes | Honey from your own stock, Probiotics, Commercially available supplements | none |
| 784 | 2019 | 4.067659 | Yes | Sugar roll, Drone brood inspection | Yes | Formic Acid (Mite Away Quick Strips) | No | Cane sugar candyboard made with H2O and one Tbs Vinegar | none |
| 785 | 2019 | 4.067659 | Yes | Sugar roll, Drone brood inspection | Yes | Formic Acid (Mite Away Quick Strips) | No | Cane sugar candyboard made with H2O and one Tbs Vinegar | none |
| 786 | 2019 | 5.888852 | No | NA | Yes | Oxalic Acid | Yes | Fondant or sugar candy | sugar only |
| 787 | 2019 | 5.888852 | No | NA | Yes | Oxalic Acid | Yes | Fondant or sugar candy | sugar only |
| 788 | 2019 | 5.888852 | No | NA | Yes | Oxalic Acid | Yes | Fondant or sugar candy | sugar only |
| 789 | 2019 | 5.477108 | Yes | Sugar roll | Yes | Apivar (Amitraz) | Yes | Fondant or sugar candy, Dry sugar, Honey from your own stock, Pollen from your own stock | none |
| 790 | 2019 | 5.477108 | Yes | Sugar roll | Yes | Apivar (Amitraz) | Yes | Fondant or sugar candy, Dry sugar, Honey from your own stock, Pollen from your own stock | none |
| 791 | 2019 | 5.477108 | Yes | Sugar roll | Yes | Apivar (Amitraz) | Yes | Fondant or sugar candy, Dry sugar, Honey from your own stock, Pollen from your own stock | none |
| 792 | 2019 | 5.477108 | Yes | Sugar roll | Yes | Apivar (Amitraz) | Yes | Fondant or sugar candy, Dry sugar, Honey from your own stock, Pollen from your own stock | none |
| 793 | 2019 | 1.534616 | Yes | 48 hr drop (sticky board) | Yes | Oxalic Acid | Yes | Dry sugar | sugar only |
| 794 | 2019 | 1.534616 | Yes | 48 hr drop (sticky board) | Yes | Oxalic Acid | Yes | Dry sugar | sugar only |
| 795 | 2019 | 1.534616 | Yes | 48 hr drop (sticky board) | Yes | Oxalic Acid | Yes | Dry sugar | sugar only |
| 796 | 2019 | 1.534616 | Yes | 48 hr drop (sticky board) | Yes | Oxalic Acid | Yes | Dry sugar | sugar only |
| 797 | 2019 | 1.534616 | Yes | 48 hr drop (sticky board) | Yes | Oxalic Acid | Yes | Dry sugar | sugar only |
| 798 | 2019 | 1.534616 | Yes | 48 hr drop (sticky board) | Yes | Oxalic Acid | Yes | Dry sugar | sugar only |
| 799 | 2019 | 1.534616 | Yes | 48 hr drop (sticky board) | Yes | Oxalic Acid | Yes | Dry sugar | sugar only |
| 800 | 2019 | 1.534616 | Yes | 48 hr drop (sticky board) | Yes | Oxalic Acid | Yes | Dry sugar | sugar only |
| 801 | 2019 | 1.534616 | Yes | 48 hr drop (sticky board) | Yes | Oxalic Acid | Yes | Dry sugar | sugar only |
| 802 | 2019 | 1.534616 | Yes | 48 hr drop (sticky board) | Yes | Oxalic Acid | Yes | Dry sugar | sugar only |
| 803 | 2019 | 1.534616 | Yes | 48 hr drop (sticky board) | Yes | Oxalic Acid | Yes | Dry sugar | sugar only |
| 804 | 2019 | 1.534616 | Yes | 48 hr drop (sticky board) | Yes | Oxalic Acid | Yes | Dry sugar | sugar only |
| 805 | 2019 | 1.534616 | Yes | 48 hr drop (sticky board) | Yes | Oxalic Acid | Yes | Dry sugar | sugar only |
| 806 | 2019 | 1.534616 | Yes | 48 hr drop (sticky board) | Yes | Oxalic Acid | Yes | Dry sugar | sugar only |
| 807 | 2019 | 1.534616 | Yes | 48 hr drop (sticky board) | Yes | Oxalic Acid | Yes | Dry sugar | sugar only |
| 808 | 2019 | 10.00196 | Yes | Alcohol wash | Yes | Oxalic Acid | Yes | Fondant or sugar candy | sugar only |
| 809 | 2019 | 10.00196 | Yes | Alcohol wash | Yes | Oxalic Acid | Yes | Fondant or sugar candy | sugar only |
| 810 | 2019 | 2.134732 | Yes | Alcohol wash | Yes | Apivar (Amitraz) | Yes | Dry sugar, Pollen substitute | none |
| 811 | 2019 | 2.134732 | Yes | Alcohol wash | Yes | Apivar (Amitraz) | Yes | Dry sugar, Pollen substitute | none |
| 812 | 2019 | 2.134732 | Yes | Alcohol wash | Yes | Apivar (Amitraz) | Yes | Dry sugar, Pollen substitute | none |
| 813 | 2019 | 2.134732 | Yes | Alcohol wash | Yes | Apivar (Amitraz) | Yes | Dry sugar, Pollen substitute | none |
| 814 | 2019 | 2.134732 | Yes | Alcohol wash | Yes | Apivar (Amitraz) | Yes | Dry sugar, Pollen substitute | none |
| 815 | 2019 | 2.819187 | Yes | Sugar roll | Yes | Formic Acid (Mite Away Quick Strips) | Yes | Fondant or sugar candy | sugar only |
| 816 | 2019 | 2.819187 | Yes | Sugar roll | Yes | Formic Acid (Mite Away Quick Strips) | Yes | Fondant or sugar candy | sugar only |
| 817 | 2019 | 2.819187 | Yes | Sugar roll | Yes | Formic Acid (Mite Away Quick Strips) | Yes | Fondant or sugar candy | sugar only |
| 818 | 2019 | 2.819187 | Yes | Sugar roll | Yes | Formic Acid (Mite Away Quick Strips) | Yes | Fondant or sugar candy | sugar only |
| 819 | 2019 | 2.819187 | Yes | Sugar roll | Yes | Formic Acid (Mite Away Quick Strips) | Yes | Fondant or sugar candy | sugar only |
| 820 | 2019 | 2.819187 | Yes | Sugar roll | Yes | Formic Acid (Mite Away Quick Strips) | Yes | Fondant or sugar candy | sugar only |
| 821 | 2019 | 2.819187 | Yes | Sugar roll | Yes | Formic Acid (Mite Away Quick Strips) | Yes | Fondant or sugar candy | sugar only |
| 822 | 2019 | 2.819187 | Yes | Sugar roll | Yes | Formic Acid (Mite Away Quick Strips) | Yes | Fondant or sugar candy | sugar only |
| 823 | 2019 | 2.819187 | Yes | Sugar roll | Yes | Formic Acid (Mite Away Quick Strips) | Yes | Fondant or sugar candy | sugar only |
| 824 | 2019 | 2.819187 | Yes | Sugar roll | Yes | Formic Acid (Mite Away Quick Strips) | Yes | Fondant or sugar candy | sugar only |
| 825 | 2019 | 2.819187 | Yes | Sugar roll | Yes | Formic Acid (Mite Away Quick Strips) | Yes | Fondant or sugar candy | sugar only |
| 826 | 2019 | 2.819187 | Yes | Sugar roll | Yes | Formic Acid (Mite Away Quick Strips) | Yes | Fondant or sugar candy | sugar only |
| 827 | 2019 | 2.819187 | Yes | Sugar roll | Yes | Formic Acid (Mite Away Quick Strips) | Yes | Fondant or sugar candy | sugar only |
| 828 | 2019 | 2.819187 | Yes | Sugar roll | Yes | Formic Acid (Mite Away Quick Strips) | Yes | Fondant or sugar candy | sugar only |
| 829 | 2019 | 2.819187 | Yes | Sugar roll | Yes | Formic Acid (Mite Away Quick Strips) | Yes | Fondant or sugar candy | sugar only |
| 830 | 2019 | 2.819187 | Yes | Sugar roll | Yes | Formic Acid (Mite Away Quick Strips) | Yes | Fondant or sugar candy | sugar only |
| 831 | 2019 | 2.819187 | Yes | Sugar roll | Yes | Formic Acid (Mite Away Quick Strips) | Yes | Fondant or sugar candy | sugar only |
| 832 | 2019 | 12.50801 | Yes | 48 hr drop (sticky board) | Yes | Oxalic Acid | Yes | Sugar syrup, Dry sugar | none |
| 833 | 2019 | 12.50801 | Yes | 48 hr drop (sticky board) | Yes | Oxalic Acid | Yes | Sugar syrup, Dry sugar | none |
| 834 | 2019 | 1.065776 | No | NA | Yes | Oxalic Acid | Yes | Fondant or sugar candy, Sugar syrup, Pollen substitute | none |
| 835 | 2019 | 1.065776 | No | NA | Yes | Oxalic Acid | Yes | Fondant or sugar candy, Sugar syrup, Pollen substitute | none |
| 836 | 2019 | 1.065776 | No | NA | Yes | Oxalic Acid | Yes | Fondant or sugar candy, Sugar syrup, Pollen substitute | none |
| 837 | 2019 | 1.065776 | No | NA | Yes | Oxalic Acid | Yes | Fondant or sugar candy, Sugar syrup, Pollen substitute | none |
| 838 | 2019 | 1.065776 | No | NA | Yes | Oxalic Acid | Yes | Fondant or sugar candy, Sugar syrup, Pollen substitute | none |
| 839 | 2019 | 1.065776 | No | NA | Yes | Oxalic Acid | Yes | Fondant or sugar candy, Sugar syrup, Pollen substitute | none |
| 840 | 2019 | 1.065776 | No | NA | Yes | Oxalic Acid | Yes | Fondant or sugar candy, Sugar syrup, Pollen substitute | none |
| 841 | 2019 | 1.065776 | No | NA | Yes | Oxalic Acid | Yes | Fondant or sugar candy, Sugar syrup, Pollen substitute | none |
| 842 | 2019 | 1.065776 | No | NA | Yes | Oxalic Acid | Yes | Fondant or sugar candy, Sugar syrup, Pollen substitute | none |
| 843 | 2019 | 1.065776 | No | NA | Yes | Oxalic Acid | Yes | Fondant or sugar candy, Sugar syrup, Pollen substitute | none |
| 844 | 2019 | 1.065776 | No | NA | Yes | Oxalic Acid | Yes | Fondant or sugar candy, Sugar syrup, Pollen substitute | none |
| 845 | 2019 | 24.66425 | Yes | Alcohol wash | Yes | Formic Acid (Mite Away Quick Strips) | Yes | Fondant or sugar candy | sugar only |
| 846 | 2019 | 2.458251 | Yes | Sugar roll | Yes | Oxalic Acid | Yes | Fondant or sugar candy, Pollen substitute | none |
| 847 | 2019 | 2.458251 | Yes | Sugar roll | Yes | Oxalic Acid | Yes | Fondant or sugar candy, Pollen substitute | none |
| 848 | 2019 | 0.396755 | Yes | EITHER SPRAY | Yes | Formic Acid (Mite Away Quick Strips) | Yes | Fondant or sugar candy | sugar only |
| 849 | 2019 | 0.396755 | Yes | EITHER SPRAY | Yes | Formic Acid (Mite Away Quick Strips) | Yes | Fondant or sugar candy | sugar only |
| 850 | 2019 | 0.396755 | Yes | EITHER SPRAY | Yes | Formic Acid (Mite Away Quick Strips) | Yes | Fondant or sugar candy | sugar only |
| 851 | 2019 | 0.396755 | Yes | EITHER SPRAY | Yes | Formic Acid (Mite Away Quick Strips) | Yes | Fondant or sugar candy | sugar only |
| 852 | 2019 | 0.396755 | Yes | EITHER SPRAY | Yes | Formic Acid (Mite Away Quick Strips) | Yes | Fondant or sugar candy | sugar only |
| 853 | 2019 | 0.396755 | Yes | EITHER SPRAY | Yes | Formic Acid (Mite Away Quick Strips) | Yes | Fondant or sugar candy | sugar only |
| 854 | 2019 | 0.396755 | Yes | EITHER SPRAY | Yes | Formic Acid (Mite Away Quick Strips) | Yes | Fondant or sugar candy | sugar only |
| 855 | 2019 | 0.396755 | Yes | EITHER SPRAY | Yes | Formic Acid (Mite Away Quick Strips) | Yes | Fondant or sugar candy | sugar only |
| 856 | 2019 | 0.396755 | Yes | EITHER SPRAY | Yes | Formic Acid (Mite Away Quick Strips) | Yes | Fondant or sugar candy | sugar only |
| 857 | 2019 | 0.396755 | Yes | EITHER SPRAY | Yes | Formic Acid (Mite Away Quick Strips) | Yes | Fondant or sugar candy | sugar only |
| 858 | 2019 | 0.396755 | Yes | EITHER SPRAY | Yes | Formic Acid (Mite Away Quick Strips) | Yes | Fondant or sugar candy | sugar only |
| 859 | 2019 | 0.396755 | Yes | EITHER SPRAY | Yes | Formic Acid (Mite Away Quick Strips) | Yes | Fondant or sugar candy | sugar only |
| 860 | 2019 | 0.396755 | Yes | EITHER SPRAY | Yes | Formic Acid (Mite Away Quick Strips) | Yes | Fondant or sugar candy | sugar only |
| 861 | 2019 | 0.396755 | Yes | EITHER SPRAY | Yes | Formic Acid (Mite Away Quick Strips) | Yes | Fondant or sugar candy | sugar only |
| 862 | 2019 | 0.396755 | Yes | EITHER SPRAY | Yes | Formic Acid (Mite Away Quick Strips) | Yes | Fondant or sugar candy | sugar only |
| 863 | 2019 | 0.396755 | Yes | EITHER SPRAY | Yes | Formic Acid (Mite Away Quick Strips) | Yes | Fondant or sugar candy | sugar only |
| 864 | 2019 | 3.172432 | Yes | Sugar roll | Yes | Formic Acid (Mite Away Quick Strips) | Yes | Fondant or sugar candy | sugar only |
| 865 | 2019 | 3.172432 | Yes | Sugar roll | Yes | Formic Acid (Mite Away Quick Strips) | Yes | Fondant or sugar candy | sugar only |
| 866 | 2019 | 3.172432 | Yes | Sugar roll | Yes | Formic Acid (Mite Away Quick Strips) | Yes | Fondant or sugar candy | sugar only |
| 867 | 2019 | 3.172432 | Yes | Sugar roll | Yes | Formic Acid (Mite Away Quick Strips) | Yes | Fondant or sugar candy | sugar only |
| 868 | 2019 | 3.172432 | Yes | Sugar roll | Yes | Formic Acid (Mite Away Quick Strips) | Yes | Fondant or sugar candy | sugar only |
| 869 | 2019 | 1.204323 | Yes | Alcohol wash | Yes | Apivar (Amitraz) | Yes | Fondant or sugar candy | sugar only |
| 870 | 2019 | 1.204323 | Yes | Alcohol wash | Yes | Apivar (Amitraz) | Yes | Fondant or sugar candy | sugar only |
| 871 | 2019 | 1.204323 | Yes | Alcohol wash | Yes | Apivar (Amitraz) | Yes | Fondant or sugar candy | sugar only |
| 872 | 2019 | 1.117046 | Yes | 48 hr drop (sticky board), Alcohol wash | Yes | Formic Acid (Mite Away Quick Strips) | Yes | Fondant or sugar candy, Pollen substitute | none |
| 873 | 2019 | 1.117046 | Yes | 48 hr drop (sticky board), Alcohol wash | Yes | Formic Acid (Mite Away Quick Strips) | Yes | Fondant or sugar candy, Pollen substitute | none |
| 874 | 2019 | 1.117046 | Yes | 48 hr drop (sticky board), Alcohol wash | Yes | Formic Acid (Mite Away Quick Strips) | Yes | Fondant or sugar candy, Pollen substitute | none |
| 875 | 2019 | 1.117046 | Yes | 48 hr drop (sticky board), Alcohol wash | Yes | Formic Acid (Mite Away Quick Strips) | Yes | Fondant or sugar candy, Pollen substitute | none |
| 876 | 2019 | 1.117046 | Yes | 48 hr drop (sticky board), Alcohol wash | Yes | Formic Acid (Mite Away Quick Strips) | Yes | Fondant or sugar candy, Pollen substitute | none |
| 877 | 2019 | 1.117046 | Yes | 48 hr drop (sticky board), Alcohol wash | Yes | Formic Acid (Mite Away Quick Strips) | Yes | Fondant or sugar candy, Pollen substitute | none |
| 878 | 2019 | 1.117046 | Yes | 48 hr drop (sticky board), Alcohol wash | Yes | Formic Acid (Mite Away Quick Strips) | Yes | Fondant or sugar candy, Pollen substitute | none |
| 879 | 2019 | 4.552475 | Yes | Sugar roll | Yes | Oxalic Acid | Yes | Fondant or sugar candy | sugar only |
| 880 | 2019 | 4.552475 | Yes | Sugar roll | Yes | Oxalic Acid | Yes | Fondant or sugar candy | sugar only |
| 881 | 2019 | 3.392435 | Yes | 48 hr drop (sticky board) | Yes | Apivar (Amitraz) | Yes | Sugar syrup | none |
| 882 | 2019 | 2.104879 | Yes | Alcohol wash | Yes | Formic Acid (Mite Away Quick Strips) | Yes | Fondant or sugar candy | sugar only |
| 883 | 2019 | 2.104879 | Yes | Alcohol wash | Yes | Formic Acid (Mite Away Quick Strips) | Yes | Fondant or sugar candy | sugar only |
| 884 | 2019 | 2.104879 | Yes | Alcohol wash | Yes | Formic Acid (Mite Away Quick Strips) | Yes | Fondant or sugar candy | sugar only |
| 885 | 2019 | 2.104879 | Yes | Alcohol wash | Yes | Formic Acid (Mite Away Quick Strips) | Yes | Fondant or sugar candy | sugar only |
| 886 | 2019 | 2.104879 | Yes | Alcohol wash | Yes | Formic Acid (Mite Away Quick Strips) | Yes | Fondant or sugar candy | sugar only |
| 887 | 2019 | 10.76876 | No | NA | Yes | Apivar (Amitraz) | Yes | Dry sugar | sugar only |
| 888 | 2019 | 10.76876 | No | NA | Yes | Apivar (Amitraz) | Yes | Dry sugar | sugar only |
| 889 | 2020 | 3.235273 | Yes | Drone brood inspection | Yes | Apivar (Amitraz) | Yes | Honey from your own stock | none |
| 890 | 2020 | 11.9477 | Yes | Sugar roll | Yes | Formic Acid (Mite Away Quick Strips) | Yes | Fondant or sugar candy | sugar only |
| 891 | 2020 | 5.354176 | Yes | Drone brood inspection | Yes | Formic Acid (Mite Away Quick Strips) | Yes | Fondant or sugar candy;Pollen substitute | none |
| 892 | 2020 | 5.354176 | Yes | Drone brood inspection | Yes | Formic Acid (Mite Away Quick Strips) | Yes | Fondant or sugar candy;Pollen substitute | none |
| 893 | 2020 | 5.354176 | Yes | Drone brood inspection | Yes | Formic Acid (Mite Away Quick Strips) | Yes | Fondant or sugar candy;Pollen substitute | none |
| 894 | 2020 | 5.354176 | Yes | Drone brood inspection | Yes | Formic Acid (Mite Away Quick Strips) | Yes | Fondant or sugar candy;Pollen substitute | none |
| 895 | 2020 | 0.917927 | Yes | Sugar roll | Yes | Apivar (Amitraz) | Yes | Fondant or sugar candy | sugar only |
| 896 | 2020 | 0.917927 | Yes | Sugar roll | Yes | Apivar (Amitraz) | Yes | Fondant or sugar candy | sugar only |
| 897 | 2020 | 0.739574 | Yes | 48 hr drop (sticky board);Sugar roll | Yes | Formic Acid (Mite Away Quick Strips) | Yes | Honey from your own stock | none |
| 898 | 2020 | 0.739574 | Yes | 48 hr drop (sticky board);Sugar roll | Yes | Formic Acid (Mite Away Quick Strips) | Yes | Honey from your own stock | none |
| 899 | 2020 | 0.739574 | Yes | 48 hr drop (sticky board);Sugar roll | Yes | Formic Acid (Mite Away Quick Strips) | Yes | Honey from your own stock | none |
| 900 | 2020 | 0.739574 | Yes | 48 hr drop (sticky board);Sugar roll | Yes | Formic Acid (Mite Away Quick Strips) | Yes | Honey from your own stock | none |
| 901 | 2020 | 0.739574 | Yes | 48 hr drop (sticky board);Sugar roll | Yes | Formic Acid (Mite Away Quick Strips) | Yes | Honey from your own stock | none |
| 902 | 2020 | 0.739574 | Yes | 48 hr drop (sticky board);Sugar roll | Yes | Formic Acid (Mite Away Quick Strips) | Yes | Honey from your own stock | none |
| 903 | 2020 | 0.739574 | Yes | 48 hr drop (sticky board);Sugar roll | Yes | Formic Acid (Mite Away Quick Strips) | Yes | Honey from your own stock | none |
| 904 | 2020 | 0.739574 | Yes | 48 hr drop (sticky board);Sugar roll | Yes | Formic Acid (Mite Away Quick Strips) | Yes | Honey from your own stock | none |
| 905 | 2020 | 0.739574 | Yes | 48 hr drop (sticky board);Sugar roll | Yes | Formic Acid (Mite Away Quick Strips) | Yes | Honey from your own stock | none |
| 906 | 2020 | 0.739574 | Yes | 48 hr drop (sticky board);Sugar roll | Yes | Formic Acid (Mite Away Quick Strips) | Yes | Honey from your own stock | none |
| 907 | 2020 | 0.739574 | Yes | 48 hr drop (sticky board);Sugar roll | Yes | Formic Acid (Mite Away Quick Strips) | Yes | Honey from your own stock | none |
| 908 | 2020 | 0.739574 | Yes | 48 hr drop (sticky board);Sugar roll | Yes | Formic Acid (Mite Away Quick Strips) | Yes | Honey from your own stock | none |
| 909 | 2020 | 0.739574 | Yes | 48 hr drop (sticky board);Sugar roll | Yes | Formic Acid (Mite Away Quick Strips) | Yes | Honey from your own stock | none |
| 910 | 2020 | 0.739574 | Yes | 48 hr drop (sticky board);Sugar roll | Yes | Formic Acid (Mite Away Quick Strips) | Yes | Honey from your own stock | none |
| 911 | 2020 | 5.506286 | Yes | 48 hr drop (sticky board) | Yes | Oxalic Acid (Vapor) | Yes | Fondant or sugar candy | sugar only |
| 912 | 2020 | 5.506286 | Yes | 48 hr drop (sticky board) | Yes | Oxalic Acid (Vapor) | Yes | Fondant or sugar candy | sugar only |
| 913 | 2020 | 5.506286 | Yes | 48 hr drop (sticky board) | Yes | Oxalic Acid (Vapor) | Yes | Fondant or sugar candy | sugar only |
| 914 | 2020 | 1.122071 | Yes | Alcohol wash | Yes | Oxalic Acid (Vapor) | Yes | Fondant or sugar candy;Honey from your own stock | none |
| 915 | 2020 | 1.122071 | Yes | Alcohol wash | Yes | Oxalic Acid (Vapor) | Yes | Fondant or sugar candy;Honey from your own stock | none |
| 916 | 2020 | 1.122071 | Yes | Alcohol wash | Yes | Oxalic Acid (Vapor) | Yes | Fondant or sugar candy;Honey from your own stock | none |
| 917 | 2020 | 1.122071 | Yes | Alcohol wash | Yes | Oxalic Acid (Vapor) | Yes | Fondant or sugar candy;Honey from your own stock | none |
| 918 | 2020 | 1.122071 | Yes | Alcohol wash | Yes | Oxalic Acid (Vapor) | Yes | Fondant or sugar candy;Honey from your own stock | none |
| 919 | 2020 | 1.122071 | Yes | Alcohol wash | Yes | Oxalic Acid (Vapor) | Yes | Fondant or sugar candy;Honey from your own stock | none |
| 920 | 2020 | 1.122071 | Yes | Alcohol wash | Yes | Oxalic Acid (Vapor) | Yes | Fondant or sugar candy;Honey from your own stock | none |
| 921 | 2020 | 4.33442 | Yes | Sugar roll | Yes | Organic: Powder Sugar and Garlic | Yes | Fondant or sugar candy | sugar only |
| 922 | 2020 | 0.378544 | Yes | site inspection | Yes | Formic Acid (Mite Away Quick Strips) | Yes | Fondant or sugar candy | sugar only |
| 923 | 2020 | 0.378544 | Yes | site inspection | Yes | Formic Acid (Mite Away Quick Strips) | Yes | Fondant or sugar candy | sugar only |
| 924 | 2020 | 0.378544 | Yes | site inspection | Yes | Formic Acid (Mite Away Quick Strips) | Yes | Fondant or sugar candy | sugar only |
| 925 | 2020 | 0.378544 | Yes | site inspection | Yes | Formic Acid (Mite Away Quick Strips) | Yes | Fondant or sugar candy | sugar only |
| 926 | 2020 | 0.378544 | Yes | site inspection | Yes | Formic Acid (Mite Away Quick Strips) | Yes | Fondant or sugar candy | sugar only |
| 927 | 2020 | 0.378544 | Yes | site inspection | Yes | Formic Acid (Mite Away Quick Strips) | Yes | Fondant or sugar candy | sugar only |
| 928 | 2020 | 0.378544 | Yes | site inspection | Yes | Formic Acid (Mite Away Quick Strips) | Yes | Fondant or sugar candy | sugar only |
| 929 | 2020 | 0.378544 | Yes | site inspection | Yes | Formic Acid (Mite Away Quick Strips) | Yes | Fondant or sugar candy | sugar only |
| 930 | 2020 | 0.378544 | Yes | site inspection | Yes | Formic Acid (Mite Away Quick Strips) | Yes | Fondant or sugar candy | sugar only |
| 931 | 2020 | 0.378544 | Yes | site inspection | Yes | Formic Acid (Mite Away Quick Strips) | Yes | Fondant or sugar candy | sugar only |
| 932 | 2020 | 0.378544 | Yes | site inspection | Yes | Formic Acid (Mite Away Quick Strips) | Yes | Fondant or sugar candy | sugar only |
| 933 | 2020 | 38.76909 | Yes | Alcohol wash | Yes | Formic Acid (Mite Away Quick Strips) | Yes | Sugar syrup | none |
| 934 | 2020 | 38.76909 | Yes | Alcohol wash | Yes | Formic Acid (Mite Away Quick Strips) | Yes | Sugar syrup | none |
| 935 | 2020 | 38.76909 | Yes | Alcohol wash | Yes | Formic Acid (Mite Away Quick Strips) | Yes | Sugar syrup | none |
| 936 | 2020 | 1.988748 | Yes | Drone brood inspection | Yes | Oxalic Acid (Vapor) | Yes | Honey from your own stock;Commercially available supplements | none |
| 937 | 2020 | 1.988748 | Yes | Drone brood inspection | Yes | Oxalic Acid (Vapor) | Yes | Honey from your own stock;Commercially available supplements | none |
| 938 | 2020 | 1.988748 | Yes | Drone brood inspection | Yes | Oxalic Acid (Vapor) | Yes | Honey from your own stock;Commercially available supplements | none |
| 939 | 2020 | 1.988748 | Yes | Drone brood inspection | Yes | Oxalic Acid (Vapor) | Yes | Honey from your own stock;Commercially available supplements | none |
| 940 | 2020 | 1.988748 | Yes | Drone brood inspection | Yes | Oxalic Acid (Vapor) | Yes | Honey from your own stock;Commercially available supplements | none |
| 941 | 2020 | 1.988748 | Yes | Drone brood inspection | Yes | Oxalic Acid (Vapor) | Yes | Honey from your own stock;Commercially available supplements | none |
| 942 | 2020 | 1.988748 | Yes | Drone brood inspection | Yes | Oxalic Acid (Vapor) | Yes | Honey from your own stock;Commercially available supplements | none |
| 943 | 2020 | 1.988748 | Yes | Drone brood inspection | Yes | Oxalic Acid (Vapor) | Yes | Honey from your own stock;Commercially available supplements | none |
| 944 | 2020 | 1.988748 | Yes | Drone brood inspection | Yes | Oxalic Acid (Vapor) | Yes | Honey from your own stock;Commercially available supplements | none |
| 945 | 2020 | 1.988748 | Yes | Drone brood inspection | Yes | Oxalic Acid (Vapor) | Yes | Honey from your own stock;Commercially available supplements | none |
| 946 | 2020 | 1.988748 | Yes | Drone brood inspection | Yes | Oxalic Acid (Vapor) | Yes | Honey from your own stock;Commercially available supplements | none |
| 947 | 2020 | 1.988748 | Yes | Drone brood inspection | Yes | Oxalic Acid (Vapor) | Yes | Honey from your own stock;Commercially available supplements | none |
| 948 | 2020 | 0.80614 | Yes | Sugar roll | Yes | Formic Acid (Mite Away Quick Strips) | Yes | Sugar syrup;Grease patties | none |
| 949 | 2020 | 0.80614 | Yes | Sugar roll | Yes | Formic Acid (Mite Away Quick Strips) | Yes | Sugar syrup;Grease patties | none |
| 950 | 2020 | 0.80614 | Yes | Sugar roll | Yes | Formic Acid (Mite Away Quick Strips) | Yes | Sugar syrup;Grease patties | none |
| 951 | 2020 | 0.80614 | Yes | Sugar roll | Yes | Formic Acid (Mite Away Quick Strips) | Yes | Sugar syrup;Grease patties | none |
| 952 | 2020 | 0.80614 | Yes | Sugar roll | Yes | Formic Acid (Mite Away Quick Strips) | Yes | Sugar syrup;Grease patties | none |
| 953 | 2020 | 0.80614 | Yes | Sugar roll | Yes | Formic Acid (Mite Away Quick Strips) | Yes | Sugar syrup;Grease patties | none |
| 954 | 2020 | 0.80614 | Yes | Sugar roll | Yes | Formic Acid (Mite Away Quick Strips) | Yes | Sugar syrup;Grease patties | none |
| 955 | 2020 | 0.80614 | Yes | Sugar roll | Yes | Formic Acid (Mite Away Quick Strips) | Yes | Sugar syrup;Grease patties | none |
| 956 | 2020 | 0.80614 | Yes | Sugar roll | Yes | Formic Acid (Mite Away Quick Strips) | Yes | Sugar syrup;Grease patties | none |
| 957 | 2020 | 0.80614 | Yes | Sugar roll | Yes | Formic Acid (Mite Away Quick Strips) | Yes | Sugar syrup;Grease patties | none |
| 958 | 2020 | 0.80614 | Yes | Sugar roll | Yes | Formic Acid (Mite Away Quick Strips) | Yes | Sugar syrup;Grease patties | none |
| 959 | 2020 | 0.80614 | Yes | Sugar roll | Yes | Formic Acid (Mite Away Quick Strips) | Yes | Sugar syrup;Grease patties | none |
| 960 | 2020 | 14.67821 | No | NA | Yes | Formic Acid (Mite Away Quick Strips) | Yes | Fondant or sugar candy | sugar only |
| 961 | 2020 | 14.67821 | No | NA | Yes | Formic Acid (Mite Away Quick Strips) | Yes | Fondant or sugar candy | sugar only |
| 962 | 2020 | 14.67821 | No | NA | Yes | Formic Acid (Mite Away Quick Strips) | Yes | Fondant or sugar candy | sugar only |
| 963 | 2020 | 9.146971 | Yes | Alcohol wash | Yes | Formic Acid (Mite Away Quick Strips) | Yes | Fondant or sugar candy;Sugar syrup;Honey from your own stock | none |
| 964 | 2020 | 9.146971 | Yes | Alcohol wash | Yes | Formic Acid (Mite Away Quick Strips) | Yes | Fondant or sugar candy;Sugar syrup;Honey from your own stock | none |
| 965 | 2020 | 9.146971 | Yes | Alcohol wash | Yes | Formic Acid (Mite Away Quick Strips) | Yes | Fondant or sugar candy;Sugar syrup;Honey from your own stock | none |
| 966 | 2020 | 9.365107 | Yes | Drone brood inspection | Yes | Formic Acid (Mite Away Quick Strips) | Yes | Fondant or sugar candy;Honey from your own stock | none |
| 967 | 2020 | 9.365107 | Yes | Drone brood inspection | Yes | Formic Acid (Mite Away Quick Strips) | Yes | Fondant or sugar candy;Honey from your own stock | none |
| 968 | 2020 | 9.365107 | Yes | Drone brood inspection | Yes | Formic Acid (Mite Away Quick Strips) | Yes | Fondant or sugar candy;Honey from your own stock | none |
| 969 | 2020 | 2.420878 | Yes | Sugar roll;Drone brood inspection | Yes | Formic Acid (Mite Away Quick Strips) | Yes | Dry sugar;Pollen substitute | none |
| 970 | 2020 | 2.420878 | Yes | Sugar roll;Drone brood inspection | Yes | Formic Acid (Mite Away Quick Strips) | Yes | Dry sugar;Pollen substitute | none |
| 971 | 2020 | 2.420878 | Yes | Sugar roll;Drone brood inspection | Yes | Formic Acid (Mite Away Quick Strips) | Yes | Dry sugar;Pollen substitute | none |
| 972 | 2020 | 2.420878 | Yes | Sugar roll;Drone brood inspection | Yes | Formic Acid (Mite Away Quick Strips) | Yes | Dry sugar;Pollen substitute | none |
| 973 | 2020 | 4.295717 | Yes | Sugar roll | Yes | Apivar (Amitraz) | Yes | Fondant or sugar candy;Sugar syrup | none |
| 974 | 2020 | 4.295717 | Yes | Sugar roll | Yes | Apivar (Amitraz) | Yes | Fondant or sugar candy;Sugar syrup | none |
| 975 | 2020 | 4.295717 | Yes | Sugar roll | Yes | Apivar (Amitraz) | Yes | Fondant or sugar candy;Sugar syrup | none |
| 976 | 2020 | 4.295717 | Yes | Sugar roll | Yes | Apivar (Amitraz) | Yes | Fondant or sugar candy;Sugar syrup | none |
| 977 | 2020 | 12.03329 | Yes | Alcohol wash | Yes | Oxalic Acid (Vapor) | Yes | Dry sugar;Pollen substitute | none |
| 978 | 2020 | 12.03329 | Yes | Alcohol wash | Yes | Oxalic Acid (Vapor) | Yes | Dry sugar;Pollen substitute | none |
| 979 | 2020 | 12.03329 | Yes | Alcohol wash | Yes | Oxalic Acid (Vapor) | Yes | Dry sugar;Pollen substitute | none |
| 980 | 2020 | 12.03329 | Yes | Alcohol wash | Yes | Oxalic Acid (Vapor) | Yes | Dry sugar;Pollen substitute | none |
| 981 | 2020 | 0.737797 | Yes | Alcohol wash | Yes | Formic Acid (Mite Away Quick Strips) | Yes | Fondant or sugar candy;Sugar syrup | none |
| 982 | 2020 | 0.737797 | Yes | Alcohol wash | Yes | Formic Acid (Mite Away Quick Strips) | Yes | Fondant or sugar candy;Sugar syrup | none |
| 983 | 2020 | 0.737797 | Yes | Alcohol wash | Yes | Formic Acid (Mite Away Quick Strips) | Yes | Fondant or sugar candy;Sugar syrup | none |
| 984 | 2020 | 0.737797 | Yes | Alcohol wash | Yes | Formic Acid (Mite Away Quick Strips) | Yes | Fondant or sugar candy;Sugar syrup | none |
| 985 | 2020 | 0.737797 | Yes | Alcohol wash | Yes | Formic Acid (Mite Away Quick Strips) | Yes | Fondant or sugar candy;Sugar syrup | none |
| 986 | 2020 | 0.737797 | Yes | Alcohol wash | Yes | Formic Acid (Mite Away Quick Strips) | Yes | Fondant or sugar candy;Sugar syrup | none |
| 987 | 2020 | 0.737797 | Yes | Alcohol wash | Yes | Formic Acid (Mite Away Quick Strips) | Yes | Fondant or sugar candy;Sugar syrup | none |
| 988 | 2020 | 3.351722 | No | NA | Yes | Apivar (Amitraz) | Yes | Fondant or sugar candy;Pollen substitute | none |
| 989 | 2020 | 3.351722 | No | NA | Yes | Apivar (Amitraz) | Yes | Fondant or sugar candy;Pollen substitute | none |
| 990 | 2020 | 3.351722 | No | NA | Yes | Apivar (Amitraz) | Yes | Fondant or sugar candy;Pollen substitute | none |
| 991 | 2020 | 27.62118 | Yes | Alcohol wash | Yes | Oxalic Acid (Vapor) | Yes | Fondant or sugar candy;Dry sugar;Pollen substitute | none |
| 992 | 2020 | 27.62118 | Yes | Alcohol wash | Yes | Oxalic Acid (Vapor) | Yes | Fondant or sugar candy;Dry sugar;Pollen substitute | none |
| 993 | 2020 | 16.91923 | Yes | Alcohol wash | Yes | Apivar (Amitraz) | Yes | Sugar board with embedded pollen patty | none |
| 994 | 2020 | 16.91923 | Yes | Alcohol wash | Yes | Apivar (Amitraz) | Yes | Sugar board with embedded pollen patty | none |
| 995 | 2020 | 16.91923 | Yes | Alcohol wash | Yes | Apivar (Amitraz) | Yes | Sugar board with embedded pollen patty | none |
| 996 | 2020 | 10.73352 | Yes | 48 hr drop (sticky board);Sugar roll | Yes | Oxalic Acid (Vapor) | Yes | Fondant or sugar candy | sugar only |
| 997 | 2020 | 10.73352 | Yes | 48 hr drop (sticky board);Sugar roll | Yes | Oxalic Acid (Vapor) | Yes | Fondant or sugar candy | sugar only |
| 998 | 2020 | 10.73352 | Yes | 48 hr drop (sticky board);Sugar roll | Yes | Oxalic Acid (Vapor) | Yes | Fondant or sugar candy | sugar only |
| 999 | 2020 | 17.93897 | Yes | 48 hr drop (sticky board) | Yes | Formic Acid (Mite Away Quick Strips) | Yes | Honey from your own stock | none |
| 1000 | 2020 | 17.93897 | Yes | 48 hr drop (sticky board) | Yes | Formic Acid (Mite Away Quick Strips) | Yes | Honey from your own stock | none |
|  |  |  |  |  |  |  |  |  |  |
| 1000 | 2020 | 17.93897 | Yes | 48 hr drop (sticky board) | Yes | Formic Acid (Mite Away Quick Strips) | Yes | Honey from your own stock | none |
| 1001 | 2020 | 17.93897 | Yes | 48 hr drop (sticky board) | Yes | Formic Acid (Mite Away Quick Strips) | Yes | Honey from your own stock | none |
| 1002 | 2020 | 1.186712 | Yes | Sugar roll | Yes | Oxalic Acid (Dribble) | Yes | Fondant or sugar candy | sugar only |
| 1003 | 2020 | 1.186712 | Yes | Sugar roll | Yes | Oxalic Acid (Dribble) | Yes | Fondant or sugar candy | sugar only |
| 1004 | 2020 | 1.186712 | Yes | Sugar roll | Yes | Oxalic Acid (Dribble) | Yes | Fondant or sugar candy | sugar only |
| 1005 | 2020 | 1.186712 | Yes | Sugar roll | Yes | Oxalic Acid (Dribble) | Yes | Fondant or sugar candy | sugar only |
| 1006 | 2020 | 1.186712 | Yes | Sugar roll | Yes | Oxalic Acid (Dribble) | Yes | Fondant or sugar candy | sugar only |
| 1007 | 2020 | 1.186712 | Yes | Sugar roll | Yes | Oxalic Acid (Dribble) | Yes | Fondant or sugar candy | sugar only |
| 1008 | 2020 | 1.186712 | Yes | Sugar roll | Yes | Oxalic Acid (Dribble) | Yes | Fondant or sugar candy | sugar only |
| 1009 | 2020 | 8.811454 | Yes | Visual inspection with regular, timed treatments | Yes | Formic Acid (Mite Away Quick Strips) | Yes | Fondant or sugar candy;Pollen substitute | none |
| 1010 | 2020 | 8.811454 | Yes | Visual inspection with regular, timed treatments | Yes | Formic Acid (Mite Away Quick Strips) | Yes | Fondant or sugar candy;Pollen substitute | none |
| 1011 | 2020 | 8.811454 | Yes | Visual inspection with regular, timed treatments | Yes | Formic Acid (Mite Away Quick Strips) | Yes | Fondant or sugar candy;Pollen substitute | none |
| 1012 | 2020 | 8.811454 | Yes | Visual inspection with regular, timed treatments | Yes | Formic Acid (Mite Away Quick Strips) | Yes | Fondant or sugar candy;Pollen substitute | none |
| 1013 | 2020 | 8.811454 | Yes | Visual inspection with regular, timed treatments | Yes | Formic Acid (Mite Away Quick Strips) | Yes | Fondant or sugar candy;Pollen substitute | none |
| 1014 | 2020 | 8.811454 | Yes | Visual inspection with regular, timed treatments | Yes | Formic Acid (Mite Away Quick Strips) | Yes | Fondant or sugar candy;Pollen substitute | none |
| 1015 | 2020 | 14.46301 | Yes | Alcohol wash;Drone brood inspection | Yes | Api life Var | Yes | Fondant or sugar candy;Honey from your own stock;ProSweet | none |
| 1016 | 2020 | 14.46301 | Yes | Alcohol wash;Drone brood inspection | Yes | Api life Var | Yes | Fondant or sugar candy;Honey from your own stock;ProSweet | none |
| 1017 | 2020 | 5.437431 | Yes | Drone brood inspection | Yes | Formic Acid (Mite Away Quick Strips) | Yes | Dadant Winter Patties | none |
| 1018 | 2020 | 5.437431 | Yes | Drone brood inspection | Yes | Formic Acid (Mite Away Quick Strips) | Yes | Dadant Winter Patties | none |
| 1019 | 2020 | 5.437431 | Yes | Drone brood inspection | Yes | Formic Acid (Mite Away Quick Strips) | Yes | Dadant Winter Patties | none |
| 1020 | 2020 | 5.437431 | Yes | Drone brood inspection | Yes | Formic Acid (Mite Away Quick Strips) | Yes | Dadant Winter Patties | none |
| 1021 | 2020 | 5.437431 | Yes | Drone brood inspection | Yes | Formic Acid (Mite Away Quick Strips) | Yes | Dadant Winter Patties | none |
| 1022 | 2020 | 5.437431 | Yes | Drone brood inspection | Yes | Formic Acid (Mite Away Quick Strips) | Yes | Dadant Winter Patties | none |
| 1023 | 2020 | 5.437431 | Yes | Drone brood inspection | Yes | Formic Acid (Mite Away Quick Strips) | Yes | Dadant Winter Patties | none |
| 1024 | 2020 | 22.89714 | Yes | 48 hr drop (sticky board);Sugar roll | Yes | Formic Acid (Mite Away Quick Strips) | Yes | Fondant or sugar candy | sugar only |
| 1025 | 2020 | 22.89714 | Yes | 48 hr drop (sticky board);Sugar roll | Yes | Formic Acid (Mite Away Quick Strips) | Yes | Fondant or sugar candy | sugar only |
| 1026 | 2020 | 11.10193 | Yes | Alcohol wash | Yes | Oxalic Acid (Vapor) | Yes | Sugar syrup;Pollen substitute | none |
| 1027 | 2020 | 10.61364 | Yes | Sugar roll | Yes | Formic Acid (Mite Away Quick Strips) | Yes | Dry sugar | sugar only |
| 1028 | 2020 | 10.61364 | Yes | Sugar roll | Yes | Formic Acid (Mite Away Quick Strips) | Yes | Dry sugar | sugar only |
| 1029 | 2020 | 10.61364 | Yes | Sugar roll | Yes | Formic Acid (Mite Away Quick Strips) | Yes | Dry sugar | sugar only |
| 1030 | 2020 | 10.61364 | Yes | Sugar roll | Yes | Formic Acid (Mite Away Quick Strips) | Yes | Dry sugar | sugar only |
| 1031 | 2020 | 10.61364 | Yes | Sugar roll | Yes | Formic Acid (Mite Away Quick Strips) | Yes | Dry sugar | sugar only |
| 1032 | 2020 | 10.61364 | Yes | Sugar roll | Yes | Formic Acid (Mite Away Quick Strips) | Yes | Dry sugar | sugar only |
| 1033 | 2020 | 10.61364 | Yes | Sugar roll | Yes | Formic Acid (Mite Away Quick Strips) | Yes | Dry sugar | sugar only |
| 1034 | 2020 | 10.61364 | Yes | Sugar roll | Yes | Formic Acid (Mite Away Quick Strips) | Yes | Dry sugar | sugar only |
| 1035 | 2020 | 10.61364 | Yes | Sugar roll | Yes | Formic Acid (Mite Away Quick Strips) | Yes | Dry sugar | sugar only |
| 1036 | 2020 | 11.1254 | Yes | Alcohol wash | Yes | Formic Acid (Mite Away Quick Strips) | Yes | Fondant or sugar candy | sugar only |
| 1037 | 2020 | 11.1254 | Yes | Alcohol wash | Yes | Formic Acid (Mite Away Quick Strips) | Yes | Fondant or sugar candy | sugar only |
| 1038 | 2020 | 11.1254 | Yes | Alcohol wash | Yes | Formic Acid (Mite Away Quick Strips) | Yes | Fondant or sugar candy | sugar only |
| 1039 | 2020 | 1.005407 | Yes | 48 hr drop (sticky board) | Yes | Formic Acid (Mite Away Quick Strips) | Yes | Winter patties 3lb per hive | none |
| 1040 | 2020 | 1.005407 | Yes | 48 hr drop (sticky board) | Yes | Formic Acid (Mite Away Quick Strips) | Yes | Winter patties 3lb per hive | none |
| 1041 | 2020 | 1.005407 | Yes | 48 hr drop (sticky board) | Yes | Formic Acid (Mite Away Quick Strips) | Yes | Winter patties 3lb per hive | none |
| 1042 | 2020 | 2.230315 | Yes | 48 hr drop (sticky board) | Yes | Oxalic Acid (Vapor) | Yes | Fondant or sugar candy | sugar only |
| 1043 | 2020 | 1.482163 | No | NA | Yes | Formic Acid (Mite Away Quick Strips) | Yes | Fondant or sugar candy;Honey from your own stock;Commercially available supplements | none |
| 1044 | 2020 | 1.482163 | No | NA | Yes | Formic Acid (Mite Away Quick Strips) | Yes | Fondant or sugar candy;Honey from your own stock;Commercially available supplements | none |
| 1045 | 2020 | 1.482163 | No | NA | Yes | Formic Acid (Mite Away Quick Strips) | Yes | Fondant or sugar candy;Honey from your own stock;Commercially available supplements | none |
| 1046 | 2020 | 1.482163 | No | NA | Yes | Formic Acid (Mite Away Quick Strips) | Yes | Fondant or sugar candy;Honey from your own stock;Commercially available supplements | none |
| 1047 | 2020 | 7.501137 | No | NA | Yes | Api life Var | Yes | Fondant or sugar candy;Pollen from your own stock | none |
| 1048 | 2020 | 4.338297 | Yes | Sugar roll | Yes | Formic Acid (Mite Away Quick Strips) | Yes | Fondant or sugar candy | sugar only |
| 1049 | 2020 | 4.338297 | Yes | Sugar roll | Yes | Formic Acid (Mite Away Quick Strips) | Yes | Fondant or sugar candy | sugar only |
| 1050 | 2020 | 4.338297 | Yes | Sugar roll | Yes | Formic Acid (Mite Away Quick Strips) | Yes | Fondant or sugar candy | sugar only |
| 1051 | 2020 | 4.338297 | Yes | Sugar roll | Yes | Formic Acid (Mite Away Quick Strips) | Yes | Fondant or sugar candy | sugar only |
| 1052 | 2020 | 4.338297 | Yes | Sugar roll | Yes | Formic Acid (Mite Away Quick Strips) | Yes | Fondant or sugar candy | sugar only |
| 1053 | 2020 | 4.338297 | Yes | Sugar roll | Yes | Formic Acid (Mite Away Quick Strips) | Yes | Fondant or sugar candy | sugar only |
| 1054 | 2020 | 4.338297 | Yes | Sugar roll | Yes | Formic Acid (Mite Away Quick Strips) | Yes | Fondant or sugar candy | sugar only |
| 1055 | 2020 | 4.338297 | Yes | Sugar roll | Yes | Formic Acid (Mite Away Quick Strips) | Yes | Fondant or sugar candy | sugar only |
| 1056 | 2020 | 1.000594 | Yes | 48 hr drop (sticky board);Sugar roll | Yes | Apistan | Yes | Fondant or sugar candy;Honey from your own stock | none |
| 1057 | 2020 | 1.000594 | Yes | 48 hr drop (sticky board);Sugar roll | Yes | Apistan | Yes | Fondant or sugar candy;Honey from your own stock | none |
| 1058 | 2020 | 3.72417 | Yes | mite away quick strips | Yes | Formic Acid (Mite Away Quick Strips) | Yes | Fondant or sugar candy;Honey from your own stock | none |
| 1059 | 2020 | 3.72417 | Yes | mite away quick strips | Yes | Formic Acid (Mite Away Quick Strips) | Yes | Fondant or sugar candy;Honey from your own stock | none |
| 1060 | 2020 | 3.72417 | Yes | mite away quick strips | Yes | Formic Acid (Mite Away Quick Strips) | Yes | Fondant or sugar candy;Honey from your own stock | none |
| 1061 | 2020 | 3.72417 | Yes | mite away quick strips | Yes | Formic Acid (Mite Away Quick Strips) | Yes | Fondant or sugar candy;Honey from your own stock | none |
| 1062 | 2020 | 0.734856 | Yes | Alcohol wash | Yes | Oxalic Acid (Vapor) | Yes | Fondant or sugar candy | sugar only |
| 1063 | 2020 | 0.734856 | Yes | Alcohol wash | Yes | Oxalic Acid (Vapor) | Yes | Fondant or sugar candy | sugar only |
| 1064 | 2020 | 0.734856 | Yes | Alcohol wash | Yes | Oxalic Acid (Vapor) | Yes | Fondant or sugar candy | sugar only |
| 1065 | 2020 | 0.472779 | Yes | Alcohol wash | Yes | Apivar (Amitraz) | Yes | Dry sugar;Pollen substitute | none |
| 1066 | 2020 | 0.472779 | Yes | Alcohol wash | Yes | Apivar (Amitraz) | Yes | Dry sugar;Pollen substitute | none |
| 1067 | 2020 | 14.3861 | Yes | 48 hr drop (sticky board) | Yes | Formic Pro 14-day treatment option | Yes | Fondant or sugar candy;Pollen substitute | none |
| 1068 | 2020 | 3.79204 | Yes | Alcohol wash;Drone brood inspection | Yes | Oxalic Acid (Dribble) | Yes | Dry sugar | sugar only |
| 1069 | 2020 | 3.79204 | Yes | Alcohol wash;Drone brood inspection | Yes | Oxalic Acid (Dribble) | Yes | Dry sugar | sugar only |
| 1070 | 2020 | 3.79204 | Yes | Alcohol wash;Drone brood inspection | Yes | Oxalic Acid (Dribble) | Yes | Dry sugar | sugar only |
| 1071 | 2020 | 4.186873 | No | NA | Yes | Apistan | Yes | Dry sugar | sugar only |
| 1072 | 2020 | 4.186873 | No | NA | Yes | Apistan | Yes | Dry sugar | sugar only |
| 1073 | 2020 | 12.46534 | No | NA | Yes | Oxalic Acid (Vapor) | Yes | Fondant or sugar candy;Sugar syrup | none |
| 1074 | 2020 | 12.46534 | No | NA | Yes | Oxalic Acid (Vapor) | Yes | Fondant or sugar candy;Sugar syrup | none |
| 1075 | 2020 | 12.46534 | No | NA | Yes | Oxalic Acid (Vapor) | Yes | Fondant or sugar candy;Sugar syrup | none |
| 1076 | 2020 | 12.46534 | No | NA | Yes | Oxalic Acid (Vapor) | Yes | Fondant or sugar candy;Sugar syrup | none |
| 1077 | 2020 | 5.06786 | No | NA | Yes | Apiguard | Yes | Fondant or sugar candy | sugar only |
| 1078 | 2020 | 5.06786 | No | NA | Yes | Apiguard | Yes | Fondant or sugar candy | sugar only |
| 1079 | 2020 | 5.06786 | No | NA | Yes | Apiguard | Yes | Fondant or sugar candy | sugar only |
| 1080 | 2020 | 5.06786 | No | NA | Yes | Apiguard | Yes | Fondant or sugar candy | sugar only |
| 1081 | 2020 | 11.71525 | Yes | 48 hr drop (sticky board) | Yes | Formic Acid (Mite Away Quick Strips) | Yes | Sugar syrup;Dry sugar | none |
| 1082 | 2020 | 11.71525 | Yes | 48 hr drop (sticky board) | Yes | Formic Acid (Mite Away Quick Strips) | Yes | Sugar syrup;Dry sugar | none |
| 1083 | 2020 | 7.280345 | Yes | Drone brood inspection | Yes | Apivar (Amitraz) | Yes | Fondant or sugar candy;Dry sugar;Honey from your own stock;Pollen from your own stock | none |
| 1084 | 2020 | 7.280345 | Yes | Drone brood inspection | Yes | Apivar (Amitraz) | Yes | Fondant or sugar candy;Dry sugar;Honey from your own stock;Pollen from your own stock | none |
| 1085 | 2020 | 2.099058 | Yes | Sugar roll | Yes | Oxalic Acid (Vapor) | Yes | Dry sugar | sugar only |
| 1086 | 2020 | 2.099058 | Yes | Sugar roll | Yes | Oxalic Acid (Vapor) | Yes | Dry sugar | sugar only |
| 1087 | 2020 | 2.099058 | Yes | Sugar roll | Yes | Oxalic Acid (Vapor) | Yes | Dry sugar | sugar only |
| 1088 | 2020 | 2.099058 | Yes | Sugar roll | Yes | Oxalic Acid (Vapor) | Yes | Dry sugar | sugar only |
| 1089 | 2020 | 2.099058 | Yes | Sugar roll | Yes | Oxalic Acid (Vapor) | Yes | Dry sugar | sugar only |
| 1090 | 2020 | 0.948677 | Yes | Alcohol wash | Yes | Formalic Pro early Oct 2019 | Yes | Fondant or sugar candy;Bee Fondant sold on Amazon | none |
| 1091 | 2020 | 0.948677 | Yes | Alcohol wash | Yes | Formalic Pro early Oct 2019 | Yes | Fondant or sugar candy;Bee Fondant sold on Amazon | none |
| 1092 | 2020 | 6.430027 | Yes | 48 hr drop (sticky board);Sugar roll | Yes | Oxalic Acid (Dribble) | Yes | Fondant or sugar candy;Sugar syrup | none |
| 1093 | 2020 | 6.430027 | Yes | 48 hr drop (sticky board);Sugar roll | Yes | Oxalic Acid (Dribble) | Yes | Fondant or sugar candy;Sugar syrup | none |
| 1094 | 2020 | 6.158187 | Yes | Alcohol wash | Yes | Formic Acid (Mite Away Quick Strips) | Yes | Dry sugar;Honey from your own stock | none |
| 1095 | 2020 | 11.22405 | Yes | Alcohol wash | Yes | Formic Acid (Mite Away Quick Strips) | Yes | Commercially available supplements | none |
| 1096 | 2020 | 11.22405 | Yes | Alcohol wash | Yes | Formic Acid (Mite Away Quick Strips) | Yes | Commercially available supplements | none |
| 1097 | 2020 | 0.563281 | Yes | Drone brood inspection | Yes | Formic Acid (Mite Away Quick Strips) | Yes | Dry sugar;Pollen substitute;Honey from your own stock | none |
| 1098 | 2020 | 0.563281 | Yes | Drone brood inspection | Yes | Formic Acid (Mite Away Quick Strips) | Yes | Dry sugar;Pollen substitute;Honey from your own stock | none |
| 1099 | 2020 | 0.563281 | Yes | Drone brood inspection | Yes | Formic Acid (Mite Away Quick Strips) | Yes | Dry sugar;Pollen substitute;Honey from your own stock | none |
| 1100 | 2020 | 0.563281 | Yes | Drone brood inspection | Yes | Formic Acid (Mite Away Quick Strips) | Yes | Dry sugar;Pollen substitute;Honey from your own stock | none |
| 1101 | 2020 | 0.563281 | Yes | Drone brood inspection | Yes | Formic Acid (Mite Away Quick Strips) | Yes | Dry sugar;Pollen substitute;Honey from your own stock | none |
| 1102 | 2020 | 1.338395 | Yes | Sugar roll | Yes | Formic Acid (Mite Away Quick Strips) | Yes | Fondant or sugar candy | sugar only |
| 1103 | 2020 | 1.338395 | Yes | Sugar roll | Yes | Formic Acid (Mite Away Quick Strips) | Yes | Fondant or sugar candy | sugar only |
| 1104 | 2020 | 17.38766 | Yes | Alcohol wash | Yes | Oxalic Acid (Vapor) | Yes | Fondant or sugar candy;Pollen substitute | none |
| 1105 | 2020 | 17.38766 | Yes | Alcohol wash | Yes | Oxalic Acid (Vapor) | Yes | Fondant or sugar candy;Pollen substitute | none |
| 1106 | 2020 | 17.38766 | Yes | Alcohol wash | Yes | Oxalic Acid (Vapor) | Yes | Fondant or sugar candy;Pollen substitute | none |
| 1107 | 2020 | 17.38766 | Yes | Alcohol wash | Yes | Oxalic Acid (Vapor) | Yes | Fondant or sugar candy;Pollen substitute | none |
| 1108 | 2020 | 17.38766 | Yes | Alcohol wash | Yes | Oxalic Acid (Vapor) | Yes | Fondant or sugar candy;Pollen substitute | none |
| 1109 | 2020 | 4.500118 | Yes | Oxalic acid vaporizor | Yes | Oxalic Acid (Vapor) | Yes | Fondant or sugar candy | sugar only |
| 1110 | 2020 | 4.500118 | Yes | Oxalic acid vaporizor | Yes | Oxalic Acid (Vapor) | Yes | Fondant or sugar candy | sugar only |
| 1111 | 2020 | 4.500118 | Yes | Oxalic acid vaporizor | Yes | Oxalic Acid (Vapor) | Yes | Fondant or sugar candy | sugar only |
| 1112 | 2020 | 4.500118 | Yes | Oxalic acid vaporizor | Yes | Oxalic Acid (Vapor) | Yes | Fondant or sugar candy | sugar only |
| 1113 | 2020 | 1.259782 | Yes | Sugar roll | Yes | Oxalic Acid (Vapor) | Yes | Fondant or sugar candy | sugar only |
| 1114 | 2020 | 1.259782 | Yes | Sugar roll | Yes | Oxalic Acid (Vapor) | Yes | Fondant or sugar candy | sugar only |
| 1115 | 2020 | 1.259782 | Yes | Sugar roll | Yes | Oxalic Acid (Vapor) | Yes | Fondant or sugar candy | sugar only |
| 1116 | 2020 | 2.446909 | Yes | Sugar roll | Yes | Oxalic Acid (Vapor) | Yes | Fondant or sugar candy;Honey from your own stock | none |
| 1117 | 2020 | 2.446909 | Yes | Sugar roll | Yes | Oxalic Acid (Vapor) | Yes | Fondant or sugar candy;Honey from your own stock | none |
| 1118 | 2020 | 2.446909 | Yes | Sugar roll | Yes | Oxalic Acid (Vapor) | Yes | Fondant or sugar candy;Honey from your own stock | none |
| 1119 | 2020 | 2.446909 | Yes | Sugar roll | Yes | Oxalic Acid (Vapor) | Yes | Fondant or sugar candy;Honey from your own stock | none |
| 1120 | 2020 | 12.84251 | Yes | Alcohol wash | Yes | Formic Acid (Mite Away Quick Strips) | Yes | Fondant or sugar candy | sugar only |
| 1121 | 2020 | 12.84251 | Yes | Alcohol wash | Yes | Formic Acid (Mite Away Quick Strips) | Yes | Fondant or sugar candy | sugar only |
| 1122 | 2020 | 12.84251 | Yes | Alcohol wash | Yes | Formic Acid (Mite Away Quick Strips) | Yes | Fondant or sugar candy | sugar only |
| 1123 | 2020 | 12.84251 | Yes | Alcohol wash | Yes | Formic Acid (Mite Away Quick Strips) | Yes | Fondant or sugar candy | sugar only |
| 1124 | 2020 | 12.84251 | Yes | Alcohol wash | Yes | Formic Acid (Mite Away Quick Strips) | Yes | Fondant or sugar candy | sugar only |
| 1125 | 2020 | 10.46792 | Yes | Alcohol wash | Yes | Oxalic Acid (Vapor) | Yes | Fondant or sugar candy;Pollen substitute | none |
| 1126 | 2020 | 10.46792 | Yes | Alcohol wash | Yes | Oxalic Acid (Vapor) | Yes | Fondant or sugar candy;Pollen substitute | none |
| 1127 | 2020 | 10.46792 | Yes | Alcohol wash | Yes | Oxalic Acid (Vapor) | Yes | Fondant or sugar candy;Pollen substitute | none |
| 1128 | 2020 | 10.46792 | Yes | Alcohol wash | Yes | Oxalic Acid (Vapor) | Yes | Fondant or sugar candy;Pollen substitute | none |
| 1129 | 2020 | 10.46792 | Yes | Alcohol wash | Yes | Oxalic Acid (Vapor) | Yes | Fondant or sugar candy;Pollen substitute | none |
| 1130 | 2020 | 10.46792 | Yes | Alcohol wash | Yes | Oxalic Acid (Vapor) | Yes | Fondant or sugar candy;Pollen substitute | none |
| 1131 | 2020 | 10.46792 | Yes | Alcohol wash | Yes | Oxalic Acid (Vapor) | Yes | Fondant or sugar candy;Pollen substitute | none |
| 1132 | 2020 | 8.460833 | Yes | Sugar roll | Yes | Oxalic Acid (Vapor) | Yes | Fondant or sugar candy;Pollen substitute | none |
| 1133 | 2020 | 8.460833 | Yes | Sugar roll | Yes | Oxalic Acid (Vapor) | Yes | Fondant or sugar candy;Pollen substitute | none |
| 1134 | 2020 | 8.460833 | Yes | Sugar roll | Yes | Oxalic Acid (Vapor) | Yes | Fondant or sugar candy;Pollen substitute | none |
| 1135 | 2020 | 8.460833 | Yes | Sugar roll | Yes | Oxalic Acid (Vapor) | Yes | Fondant or sugar candy;Pollen substitute | none |
| 1136 | 2020 | 8.460833 | Yes | Sugar roll | Yes | Oxalic Acid (Vapor) | Yes | Fondant or sugar candy;Pollen substitute | none |
| 1137 | 2020 | 8.460833 | Yes | Sugar roll | Yes | Oxalic Acid (Vapor) | Yes | Fondant or sugar candy;Pollen substitute | none |
| 1138 | 2020 | 13.99496 | No | NA | Yes | Formic Acid (Mite Away Quick Strips) | Yes | Fondant or sugar candy;Honey from your own stock;Commercially available supplements | none |
| 1139 | 2020 | 13.99496 | No | NA | Yes | Formic Acid (Mite Away Quick Strips) | Yes | Fondant or sugar candy;Honey from your own stock;Commercially available supplements | none |
| 1140 | 2020 | 13.99496 | No | NA | Yes | Formic Acid (Mite Away Quick Strips) | Yes | Fondant or sugar candy;Honey from your own stock;Commercially available supplements | none |
| 1141 | 2020 | 2.472156 | Yes | Sugar roll | Yes | Formic Acid (Mite Away Quick Strips) | Yes | Fondant or sugar candy | sugar only |
| 1142 | 2020 | 2.472156 | Yes | Sugar roll | Yes | Formic Acid (Mite Away Quick Strips) | Yes | Fondant or sugar candy | sugar only |
| 1143 | 2020 | 2.472156 | Yes | Sugar roll | Yes | Formic Acid (Mite Away Quick Strips) | Yes | Fondant or sugar candy | sugar only |
| 1144 | 2020 | 2.472156 | Yes | Sugar roll | Yes | Formic Acid (Mite Away Quick Strips) | Yes | Fondant or sugar candy | sugar only |
| 1145 | 2020 | 2.472156 | Yes | Sugar roll | Yes | Formic Acid (Mite Away Quick Strips) | Yes | Fondant or sugar candy | sugar only |
| 1146 | 2020 | 2.472156 | Yes | Sugar roll | Yes | Formic Acid (Mite Away Quick Strips) | Yes | Fondant or sugar candy | sugar only |
| 1147 | 2020 | 9.487416 | No | NA | Yes | Apivar (Amitraz) | Yes | Fondant or sugar candy | sugar only |
| 1148 | 2020 | 9.487416 | No | NA | Yes | Apivar (Amitraz) | Yes | Fondant or sugar candy | sugar only |
| 1149 | 2020 | 4.352735 | Yes | Sugar roll | Yes | Formic Acid (Mite Away Quick Strips) | Yes | Sugar syrup | none |
| 1150 | 2020 | 4.352735 | Yes | Sugar roll | Yes | Formic Acid (Mite Away Quick Strips) | Yes | Sugar syrup | none |
| 1151 | 2020 | 4.352735 | Yes | Sugar roll | Yes | Formic Acid (Mite Away Quick Strips) | Yes | Sugar syrup | none |
| 1152 | 2020 | 14.21471 | Yes | Sugar roll | Yes | Oxalic Acid (Dribble) | Yes | Sugar syrup;Pollen substitute | none |
| 1153 | 2020 | 27.17809 | Yes | Alcohol wash | Yes | Oxalic Acid (Dribble) | Yes | Fondant or sugar candy | sugar only |
| 1154 | 2020 | 27.17809 | Yes | Alcohol wash | Yes | Oxalic Acid (Dribble) | Yes | Fondant or sugar candy | sugar only |
| 1155 | 2020 | 0.787285 | Yes | Drone brood inspection | Yes | Oxalic Acid (Vapor) | Yes | Dry sugar;Pollen substitute | none |
| 1156 | 2020 | 0.787285 | Yes | Drone brood inspection | Yes | Oxalic Acid (Vapor) | Yes | Dry sugar;Pollen substitute | none |
| 1157 | 2020 | 0.787285 | Yes | Drone brood inspection | Yes | Oxalic Acid (Vapor) | Yes | Dry sugar;Pollen substitute | none |
| 1158 | 2020 | 2.129803 | Yes | Alcohol wash | Yes | Formic Pro | Yes | Fondant or sugar candy | sugar only |
| 1159 | 2020 | 2.129803 | Yes | Alcohol wash | Yes | Formic Pro | Yes | Fondant or sugar candy | sugar only |
| 1160 | 2020 | 2.129803 | Yes | Alcohol wash | Yes | Formic Pro | Yes | Fondant or sugar candy | sugar only |
| 1161 | 2020 | 2.129803 | Yes | Alcohol wash | Yes | Formic Pro | Yes | Fondant or sugar candy | sugar only |
| 1162 | 2020 | 6.118614 | Yes | Sugar roll | Yes | Formic Acid (Mite Away Quick Strips) | Yes | Fondant or sugar candy | sugar only |
| 1163 | 2020 | 5.349672 | Yes | 48 hr drop (sticky board) | Yes | Formic Acid (Mite Away Quick Strips) | Yes | Fondant or sugar candy;Pollen substitute | none |
| 1164 | 2020 | 5.349672 | Yes | 48 hr drop (sticky board) | Yes | Formic Acid (Mite Away Quick Strips) | Yes | Fondant or sugar candy;Pollen substitute | none |
| 1165 | 2020 | 5.349672 | Yes | 48 hr drop (sticky board) | Yes | Formic Acid (Mite Away Quick Strips) | Yes | Fondant or sugar candy;Pollen substitute | none |
| 1166 | 2020 | 3.877773 | Yes | Sugar roll | Yes | Formic Acid (Mite Away Quick Strips) | Yes | Fondant or sugar candy | sugar only |
| 1167 | 2020 | 3.877773 | Yes | Sugar roll | Yes | Formic Acid (Mite Away Quick Strips) | Yes | Fondant or sugar candy | sugar only |
| 1168 | 2020 | 3.877773 | Yes | Sugar roll | Yes | Formic Acid (Mite Away Quick Strips) | Yes | Fondant or sugar candy | sugar only |
| 1169 | 2020 | 3.877773 | Yes | Sugar roll | Yes | Formic Acid (Mite Away Quick Strips) | Yes | Fondant or sugar candy | sugar only |
| 1170 | 2020 | 3.877773 | Yes | Sugar roll | Yes | Formic Acid (Mite Away Quick Strips) | Yes | Fondant or sugar candy | sugar only |
| 1171 | 2020 | 3.877773 | Yes | Sugar roll | Yes | Formic Acid (Mite Away Quick Strips) | Yes | Fondant or sugar candy | sugar only |
| 1172 | 2020 | 3.877773 | Yes | Sugar roll | Yes | Formic Acid (Mite Away Quick Strips) | Yes | Fondant or sugar candy | sugar only |
| 1173 | 2020 | 3.877773 | Yes | Sugar roll | Yes | Formic Acid (Mite Away Quick Strips) | Yes | Fondant or sugar candy | sugar only |
| 1174 | 2020 | 3.877773 | Yes | Sugar roll | Yes | Formic Acid (Mite Away Quick Strips) | Yes | Fondant or sugar candy | sugar only |
| 1175 | 2020 | 13.47158 | No | NA | Yes | Oxalic Acid (Vapor) | Yes | Fondant or sugar candy;Sugar syrup;Dry sugar | none |
| 1176 | 2020 | 13.47158 | No | NA | Yes | Oxalic Acid (Vapor) | Yes | Fondant or sugar candy;Sugar syrup;Dry sugar | none |
| 1177 | 2020 | 13.47158 | No | NA | Yes | Oxalic Acid (Vapor) | Yes | Fondant or sugar candy;Sugar syrup;Dry sugar | none |
| 1178 | 2020 | 13.47158 | No | NA | Yes | Oxalic Acid (Vapor) | Yes | Fondant or sugar candy;Sugar syrup;Dry sugar | none |
| 1179 | 2020 | 18.22957 | No | NA | Yes | Apivar (Amitraz) | Yes | Fondant or sugar candy | sugar only |
| 1180 | 2020 | 18.22957 | No | NA | Yes | Apivar (Amitraz) | Yes | Fondant or sugar candy | sugar only |
| 1181 | 2020 | 18.22957 | No | NA | Yes | Apivar (Amitraz) | Yes | Fondant or sugar candy | sugar only |
| 1182 | 2020 | 18.22957 | No | NA | Yes | Apivar (Amitraz) | Yes | Fondant or sugar candy | sugar only |
| 1183 | 2020 | 15.09605 | Yes | Alcohol wash | Yes | Api life Var | Yes | Sugar syrup | none |
| 1184 | 2020 | 15.09605 | Yes | Alcohol wash | Yes | Api life Var | Yes | Sugar syrup | none |
| 1185 | 2020 | 1.076914 | Yes | 48 hr drop (sticky board);Sugar roll | Yes | Formic Acid (Mite Away Quick Strips) | Yes | Fondant or sugar candy;Pollen substitute | none |
| 1186 | 2020 | 1.076914 | Yes | 48 hr drop (sticky board);Sugar roll | Yes | Formic Acid (Mite Away Quick Strips) | Yes | Fondant or sugar candy;Pollen substitute | none |
| 1187 | 2020 | 1.076914 | Yes | 48 hr drop (sticky board);Sugar roll | Yes | Formic Acid (Mite Away Quick Strips) | Yes | Fondant or sugar candy;Pollen substitute | none |
| 1188 | 2020 | 30.19332 | Yes | 48 hr drop (sticky board) | Yes | Apivar (Amitraz) | Yes | Honey from your own stock;Pollen from your own stock | none |
| 1189 | 2020 | 30.19332 | Yes | 48 hr drop (sticky board) | Yes | Apivar (Amitraz) | Yes | Honey from your own stock;Pollen from your own stock | none |
| 1190 | 2020 | 30.19332 | Yes | 48 hr drop (sticky board) | Yes | Apivar (Amitraz) | Yes | Honey from your own stock;Pollen from your own stock | none |
| 1191 | 2020 | 12.29912 | Yes | Alcohol wash | Yes | Oxalic Acid (Vapor) | Yes | Fondant or sugar candy | sugar only |
| 1192 | 2020 | 12.29912 | Yes | Alcohol wash | Yes | Oxalic Acid (Vapor) | Yes | Fondant or sugar candy | sugar only |
| 1193 | 2020 | 5.059035 | Yes | Drone brood inspection | Yes | Oxalic Acid (Vapor) | Yes | Dry sugar | sugar only |
| 1194 | 2020 | 5.059035 | Yes | Drone brood inspection | Yes | Oxalic Acid (Vapor) | Yes | Dry sugar | sugar only |
| 1195 | 2020 | 5.059035 | Yes | Drone brood inspection | Yes | Oxalic Acid (Vapor) | Yes | Dry sugar | sugar only |
| 1196 | 2020 | 5.059035 | Yes | Drone brood inspection | Yes | Oxalic Acid (Vapor) | Yes | Dry sugar | sugar only |
| 1197 | 2020 | 5.059035 | Yes | Drone brood inspection | Yes | Oxalic Acid (Vapor) | Yes | Dry sugar | sugar only |
| 1198 | 2020 | 5.116174 | Yes | Sugar roll | Yes | Formic Acid (Mite Away Quick Strips) | Yes | Sugar syrup | none |
| 1199 | 2020 | 4.332409 | No | Drone brood inspection | Yes | Oxalic Acid (Vapor) | Yes | Commercially available supplements | none |
| 1200 | 2020 | 4.332409 | No | Drone brood inspection | Yes | Oxalic Acid (Vapor) | Yes | Commercially available supplements | none |
| 1201 | 2020 | 4.647586 | Yes | Drone brood inspection | Yes | Formic Acid (Mite Away Quick Strips) | Yes | Honey from your own stock | none |
| 1202 | 2020 | 4.647586 | Yes | Drone brood inspection | Yes | Formic Acid (Mite Away Quick Strips) | Yes | Honey from your own stock | none |
| 1203 | 2020 | 10.91108 | Yes | Sugar roll | Yes | Apiguard thymol gel | Yes | Pollen substitute;Honey from your own stock | none |
| 1204 | 2020 | 10.91108 | Yes | Sugar roll | Yes | Apiguard thymol gel | Yes | Pollen substitute;Honey from your own stock | none |
| 1205 | 2020 | 9.416063 | Yes | Sugar roll | Yes | Formic Acid (Mite Away Quick Strips) | Yes | Honey from your own stock;Commercially available supplements | none |
| 1206 | 2020 | 9.416063 | Yes | Sugar roll | Yes | Formic Acid (Mite Away Quick Strips) | Yes | Honey from your own stock;Commercially available supplements | none |
| 1207 | 2020 | 9.416063 | Yes | Sugar roll | Yes | Formic Acid (Mite Away Quick Strips) | Yes | Honey from your own stock;Commercially available supplements | none |
| 1208 | 2020 | 9.416063 | Yes | Sugar roll | Yes | Formic Acid (Mite Away Quick Strips) | Yes | Honey from your own stock;Commercially available supplements | none |
| 1209 | 2020 | 9.416063 | Yes | Sugar roll | Yes | Formic Acid (Mite Away Quick Strips) | Yes | Honey from your own stock;Commercially available supplements | none |
| 1210 | 2020 | 9.416063 | Yes | Sugar roll | Yes | Formic Acid (Mite Away Quick Strips) | Yes | Honey from your own stock;Commercially available supplements | none |
| 1211 | 2020 | 9.416063 | Yes | Sugar roll | Yes | Formic Acid (Mite Away Quick Strips) | Yes | Honey from your own stock;Commercially available supplements | none |
| 1212 | 2020 | 9.416063 | Yes | Sugar roll | Yes | Formic Acid (Mite Away Quick Strips) | Yes | Honey from your own stock;Commercially available supplements | none |
| 1213 | 2020 | 9.416063 | Yes | Sugar roll | Yes | Formic Acid (Mite Away Quick Strips) | Yes | Honey from your own stock;Commercially available supplements | none |
| 1214 | 2020 | 9.416063 | Yes | Sugar roll | Yes | Formic Acid (Mite Away Quick Strips) | Yes | Honey from your own stock;Commercially available supplements | none |
| 1215 | 2020 | 9.416063 | Yes | Sugar roll | Yes | Formic Acid (Mite Away Quick Strips) | Yes | Honey from your own stock;Commercially available supplements | none |
| 1216 | 2020 | 9.416063 | Yes | Sugar roll | Yes | Formic Acid (Mite Away Quick Strips) | Yes | Honey from your own stock;Commercially available supplements | none |
| 1217 | 2020 | 9.416063 | Yes | Sugar roll | Yes | Formic Acid (Mite Away Quick Strips) | Yes | Honey from your own stock;Commercially available supplements | none |
| 1218 | 2020 | 9.416063 | Yes | Sugar roll | Yes | Formic Acid (Mite Away Quick Strips) | Yes | Honey from your own stock;Commercially available supplements | none |
| 1219 | 2020 | 9.416063 | Yes | Sugar roll | Yes | Formic Acid (Mite Away Quick Strips) | Yes | Honey from your own stock;Commercially available supplements | none |
| 1220 | 2020 | 22.53025 | No | NA | Yes | Apivar (Amitraz) | Yes | Fondant or sugar candy | sugar only |
| 1221 | 2020 | 8.709937 | Yes | Alcohol wash | Yes | Hopguard | Yes | Fondant or sugar candy | sugar only |
| 1222 | 2020 | 5.899641 | Yes | Sugar roll;Drone brood inspection | Yes | Oxalic Acid (Dribble) | Yes | Fondant or sugar candy | sugar only |
| 1223 | 2020 | 5.899641 | Yes | Sugar roll;Drone brood inspection | Yes | Oxalic Acid (Dribble) | Yes | Fondant or sugar candy | sugar only |
| 1224 | 2020 | 5.899641 | Yes | Sugar roll;Drone brood inspection | Yes | Oxalic Acid (Dribble) | Yes | Fondant or sugar candy | sugar only |
| 1225 | 2020 | 1.930105 | No | NA | Yes | Api life Var | Yes | Sugar syrup;Dry sugar | none |
| 1226 | 2020 | 1.930105 | No | NA | Yes | Api life Var | Yes | Sugar syrup;Dry sugar | none |
| 1227 | 2020 | 1.930105 | No | NA | Yes | Api life Var | Yes | Sugar syrup;Dry sugar | none |
| 1228 | 2020 | 1.930105 | No | NA | Yes | Api life Var | Yes | Sugar syrup;Dry sugar | none |
| 1229 | 2020 | 30.14119 | No | NA | Yes | Formic Acid (Mite Away Quick Strips) | Yes | Honey from your own stock | none |
| 1230 | 2020 | 30.14119 | No | NA | Yes | Formic Acid (Mite Away Quick Strips) | Yes | Honey from your own stock | none |
| 1231 | 2020 | 0.336536 | Yes | Alcohol wash | Yes | Formic Acid (Mite Away Quick Strips) | Yes | Dry sugar;Pollen substitute | none |
| 1232 | 2020 | 0.336536 | Yes | Alcohol wash | Yes | Formic Acid (Mite Away Quick Strips) | Yes | Dry sugar;Pollen substitute | none |
| 1233 | 2020 | 0.336536 | Yes | Alcohol wash | Yes | Formic Acid (Mite Away Quick Strips) | Yes | Dry sugar;Pollen substitute | none |
| 1234 | 2020 | 0.336536 | Yes | Alcohol wash | Yes | Formic Acid (Mite Away Quick Strips) | Yes | Dry sugar;Pollen substitute | none |
| 1235 | 2020 | 0.336536 | Yes | Alcohol wash | Yes | Formic Acid (Mite Away Quick Strips) | Yes | Dry sugar;Pollen substitute | none |
| 1236 | 2020 | 0.336536 | Yes | Alcohol wash | Yes | Formic Acid (Mite Away Quick Strips) | Yes | Dry sugar;Pollen substitute | none |
| 1237 | 2020 | 0.336536 | Yes | Alcohol wash | Yes | Formic Acid (Mite Away Quick Strips) | Yes | Dry sugar;Pollen substitute | none |
| 1238 | 2020 | 0.970326 | Yes | Apivar and Mite-Away Quick Strips | Yes | Formic Acid (Mite Away Quick Strips) | Yes | Fondant or sugar candy;Pollen substitute | none |
| 1239 | 2020 | 0.970326 | Yes | Apivar and Mite-Away Quick Strips | Yes | Formic Acid (Mite Away Quick Strips) | Yes | Fondant or sugar candy;Pollen substitute | none |
| 1240 | 2020 | 0.970326 | Yes | Apivar and Mite-Away Quick Strips | Yes | Formic Acid (Mite Away Quick Strips) | Yes | Fondant or sugar candy;Pollen substitute | none |
| 1241 | 2020 | 5.43227 | Yes | Alcohol wash | Yes | Oxalic Acid (Dribble) | Yes | Fondant or sugar candy;Honey from your own stock | none |
| 1242 | 2020 | 5.43227 | Yes | Alcohol wash | Yes | Oxalic Acid (Dribble) | Yes | Fondant or sugar candy;Honey from your own stock | none |
| 1243 | 2020 | 5.43227 | Yes | Alcohol wash | Yes | Oxalic Acid (Dribble) | Yes | Fondant or sugar candy;Honey from your own stock | none |
| 1244 | 2020 | 5.43227 | Yes | Alcohol wash | Yes | Oxalic Acid (Dribble) | Yes | Fondant or sugar candy;Honey from your own stock | none |
| 1245 | 2020 | 5.43227 | Yes | Alcohol wash | Yes | Oxalic Acid (Dribble) | Yes | Fondant or sugar candy;Honey from your own stock | none |
| 1246 | 2020 | 5.43227 | Yes | Alcohol wash | Yes | Oxalic Acid (Dribble) | Yes | Fondant or sugar candy;Honey from your own stock | none |
| 1247 | 2020 | 45.00786 | Yes | Drone brood inspection | Yes | Oxalic Acid (Dribble) | Yes | Sugar syrup | none |
| 1248 | 2020 | 13.55544 | No | NA | Yes | Formic Acid (Mite Away Quick Strips) | Yes | Fondant or sugar candy | sugar only |
| 1249 | 2020 | 13.55544 | No | NA | Yes | Formic Acid (Mite Away Quick Strips) | Yes | Fondant or sugar candy | sugar only |
| 1250 | 2020 | 13.61962 | Yes | 48 hr drop (sticky board) | Yes | Oxalic Acid (Vapor) | Yes | Fondant or sugar candy;Sugar syrup;Dry sugar | none |
| 1251 | 2020 | 13.61962 | Yes | 48 hr drop (sticky board) | Yes | Oxalic Acid (Vapor) | Yes | Fondant or sugar candy;Sugar syrup;Dry sugar | none |
| 1252 | 2020 | 13.61962 | Yes | 48 hr drop (sticky board) | Yes | Oxalic Acid (Vapor) | Yes | Fondant or sugar candy;Sugar syrup;Dry sugar | none |
| 1253 | 2020 | 13.61962 | Yes | 48 hr drop (sticky board) | Yes | Oxalic Acid (Vapor) | Yes | Fondant or sugar candy;Sugar syrup;Dry sugar | none |
| 1254 | 2020 | 13.61962 | Yes | 48 hr drop (sticky board) | Yes | Oxalic Acid (Vapor) | Yes | Fondant or sugar candy;Sugar syrup;Dry sugar | none |
| 1255 | 2020 | 1.100481 | No | NA | Yes | Formic Acid (Mite Away Quick Strips) | Yes | Fondant or sugar candy;Dry sugar;Pollen substitute;High Fructose Corn syrup | none |
| 1256 | 2020 | 1.100481 | No | NA | Yes | Formic Acid (Mite Away Quick Strips) | Yes | Fondant or sugar candy;Dry sugar;Pollen substitute;High Fructose Corn syrup | none |
| 1257 | 2020 | 1.100481 | No | NA | Yes | Formic Acid (Mite Away Quick Strips) | Yes | Fondant or sugar candy;Dry sugar;Pollen substitute;High Fructose Corn syrup | none |
| 1258 | 2020 | 1.100481 | No | NA | Yes | Formic Acid (Mite Away Quick Strips) | Yes | Fondant or sugar candy;Dry sugar;Pollen substitute;High Fructose Corn syrup | none |
| 1259 | 2020 | 1.100481 | No | NA | Yes | Formic Acid (Mite Away Quick Strips) | Yes | Fondant or sugar candy;Dry sugar;Pollen substitute;High Fructose Corn syrup | none |
| 1260 | 2020 | 1.100481 | No | NA | Yes | Formic Acid (Mite Away Quick Strips) | Yes | Fondant or sugar candy;Dry sugar;Pollen substitute;High Fructose Corn syrup | none |
| 1261 | 2020 | 31.68091 | Yes | Sugar roll | Yes | Apivar (Amitraz) | Yes | Fondant or sugar candy;Dry sugar;Pollen substitute | none |
| 1262 | 2020 | 31.68091 | Yes | Sugar roll | Yes | Apivar (Amitraz) | Yes | Fondant or sugar candy;Dry sugar;Pollen substitute | none |
| 1263 | 2020 | 31.68091 | Yes | Sugar roll | Yes | Apivar (Amitraz) | Yes | Fondant or sugar candy;Dry sugar;Pollen substitute | none |
| 1264 | 2020 | 1.430468 | No | NA | Yes | Oxalic Acid (Vapor) | Yes | Fondant or sugar candy | sugar only |
| 1265 | 2020 | 1.430468 | No | NA | Yes | Oxalic Acid (Vapor) | Yes | Fondant or sugar candy | sugar only |
| 1266 | 2020 | 1.430468 | No | NA | Yes | Oxalic Acid (Vapor) | Yes | Fondant or sugar candy | sugar only |
| 1267 | 2020 | 1.430468 | No | NA | Yes | Oxalic Acid (Vapor) | Yes | Fondant or sugar candy | sugar only |
| 1268 | 2020 | 1.430468 | No | NA | Yes | Oxalic Acid (Vapor) | Yes | Fondant or sugar candy | sugar only |
| 1269 | 2020 | 1.430468 | No | NA | Yes | Oxalic Acid (Vapor) | Yes | Fondant or sugar candy | sugar only |
| 1270 | 2020 | 10.47139 | Yes | sticky board 24 hr drop | Yes | Formic Acid (Mite Away Quick Strips) | Yes | Commercially available supplements;Commercially available winter food from Oct -March then Pollen patties in the spring | none |
| 1271 | 2020 | 10.47139 | Yes | sticky board 24 hr drop | Yes | Formic Acid (Mite Away Quick Strips) | Yes | Commercially available supplements;Commercially available winter food from Oct -March then Pollen patties in the spring | none |
| 1272 | 2020 | 27.17093 | No | NA | Yes | Formic Acid (Mite Away Quick Strips) | Yes | Fondant or sugar candy | sugar only |
| 1273 | 2020 | 27.17093 | No | NA | Yes | Formic Acid (Mite Away Quick Strips) | Yes | Fondant or sugar candy | sugar only |
| 1274 | 2020 | 27.17093 | No | NA | Yes | Formic Acid (Mite Away Quick Strips) | Yes | Fondant or sugar candy | sugar only |
| 1275 | 2020 | 27.17093 | No | NA | Yes | Formic Acid (Mite Away Quick Strips) | Yes | Fondant or sugar candy | sugar only |
| 1276 | 2020 | 1.220931 | Yes | Alcohol wash | Yes | Oxalic Acid (Vapor) | Yes | Fondant or sugar candy;Commercially available supplements | none |
| 1277 | 2020 | 1.220931 | Yes | Alcohol wash | Yes | Oxalic Acid (Vapor) | Yes | Fondant or sugar candy;Commercially available supplements | none |
| 1278 | 2020 | 1.220931 | Yes | Alcohol wash | Yes | Oxalic Acid (Vapor) | Yes | Fondant or sugar candy;Commercially available supplements | none |
| 1279 | 2020 | 1.220931 | Yes | Alcohol wash | Yes | Oxalic Acid (Vapor) | Yes | Fondant or sugar candy;Commercially available supplements | none |
| 1280 | 2020 | 1.220931 | Yes | Alcohol wash | Yes | Oxalic Acid (Vapor) | Yes | Fondant or sugar candy;Commercially available supplements | none |
| 1281 | 2020 | 1.220931 | Yes | Alcohol wash | Yes | Oxalic Acid (Vapor) | Yes | Fondant or sugar candy;Commercially available supplements | none |
| 1282 | 2020 | 1.220931 | Yes | Alcohol wash | Yes | Oxalic Acid (Vapor) | Yes | Fondant or sugar candy;Commercially available supplements | none |
| 1283 | 2020 | 1.220931 | Yes | Alcohol wash | Yes | Oxalic Acid (Vapor) | Yes | Fondant or sugar candy;Commercially available supplements | none |
| 1284 | 2020 | 1.220931 | Yes | Alcohol wash | Yes | Oxalic Acid (Vapor) | Yes | Fondant or sugar candy;Commercially available supplements | none |
| 1285 | 2020 | 1.220931 | Yes | Alcohol wash | Yes | Oxalic Acid (Vapor) | Yes | Fondant or sugar candy;Commercially available supplements | none |
| 1286 | 2020 | 1.220931 | Yes | Alcohol wash | Yes | Oxalic Acid (Vapor) | Yes | Fondant or sugar candy;Commercially available supplements | none |
| 1287 | 2020 | 1.220931 | Yes | Alcohol wash | Yes | Oxalic Acid (Vapor) | Yes | Fondant or sugar candy;Commercially available supplements | none |
| 1288 | 2020 | 1.220931 | Yes | Alcohol wash | Yes | Oxalic Acid (Vapor) | Yes | Fondant or sugar candy;Commercially available supplements | none |
| 1289 | 2020 | 1.220931 | Yes | Alcohol wash | Yes | Oxalic Acid (Vapor) | Yes | Fondant or sugar candy;Commercially available supplements | none |
| 1290 | 2020 | 1.220931 | Yes | Alcohol wash | Yes | Oxalic Acid (Vapor) | Yes | Fondant or sugar candy;Commercially available supplements | none |
| 1291 | 2020 | 1.220931 | Yes | Alcohol wash | Yes | Oxalic Acid (Vapor) | Yes | Fondant or sugar candy;Commercially available supplements | none |
| 1292 | 2020 | 1.220931 | Yes | Alcohol wash | Yes | Oxalic Acid (Vapor) | Yes | Fondant or sugar candy;Commercially available supplements | none |
| 1293 | 2020 | 1.220931 | Yes | Alcohol wash | Yes | Oxalic Acid (Vapor) | Yes | Fondant or sugar candy;Commercially available supplements | none |
| 1294 | 2020 | 1.220931 | Yes | Alcohol wash | Yes | Oxalic Acid (Vapor) | Yes | Fondant or sugar candy;Commercially available supplements | none |
| 1295 | 2020 | 1.220931 | Yes | Alcohol wash | Yes | Oxalic Acid (Vapor) | Yes | Fondant or sugar candy;Commercially available supplements | none |
| 1296 | 2020 | 1.220931 | Yes | Alcohol wash | Yes | Oxalic Acid (Vapor) | Yes | Fondant or sugar candy;Commercially available supplements | none |
| 1297 | 2020 | 1.220931 | Yes | Alcohol wash | Yes | Oxalic Acid (Vapor) | Yes | Fondant or sugar candy;Commercially available supplements | none |
| 1298 | 2020 | 1.220931 | Yes | Alcohol wash | Yes | Oxalic Acid (Vapor) | Yes | Fondant or sugar candy;Commercially available supplements | none |
| 1299 | 2020 | 1.220931 | Yes | Alcohol wash | Yes | Oxalic Acid (Vapor) | Yes | Fondant or sugar candy;Commercially available supplements | none |
| 1300 | 2020 | 1.220931 | Yes | Alcohol wash | Yes | Oxalic Acid (Vapor) | Yes | Fondant or sugar candy;Commercially available supplements | none |
| 1301 | 2020 | 2.920038 | Yes | 48 hr drop (sticky board) | Yes | Oxalic Acid (Vapor) | Yes | Fondant or sugar candy | sugar only |
| 1302 | 2020 | 2.920038 | Yes | 48 hr drop (sticky board) | Yes | Oxalic Acid (Vapor) | Yes | Fondant or sugar candy | sugar only |
| 1303 | 2020 | 10.98498 | No | NA | Yes | FormicPro | Yes | Fondant or sugar candy;Sugar syrup | none |
| 1304 | 2020 | 1.868888 | Yes | NA | Yes | Oxalic Acid (Vapor) | Yes | Fondant or sugar candy;Pollen substitute | none |
| 1305 | 2020 | 1.868888 | Yes | NA | Yes | Oxalic Acid (Vapor) | Yes | Fondant or sugar candy;Pollen substitute | none |
| 1306 | 2020 | 8.437764 | No | NA | Yes | Formic Acid (Mite Away Quick Strips) | Yes | Fondant or sugar candy;Pollen substitute;Honey from your own stock;Probiotics | none |
| 1307 | 2020 | 8.437764 | No | NA | Yes | Formic Acid (Mite Away Quick Strips) | Yes | Fondant or sugar candy;Pollen substitute;Honey from your own stock;Probiotics | none |
| 1308 | 2020 | 8.437764 | No | NA | Yes | Formic Acid (Mite Away Quick Strips) | Yes | Fondant or sugar candy;Pollen substitute;Honey from your own stock;Probiotics | none |
| 1309 | 2020 | 8.437764 | No | NA | Yes | Formic Acid (Mite Away Quick Strips) | Yes | Fondant or sugar candy;Pollen substitute;Honey from your own stock;Probiotics | none |
| 1310 | 2020 | 2.639258 | Yes | NA | Yes | Hopguard | Yes | Dry sugar | sugar only |
| 1311 | 2020 | 2.129097 | Yes | Alcohol wash | Yes | Oxalic Acid (Vapor) | Yes | Fondant or sugar candy;Commercially available supplements | none |
| 1312 | 2020 | 2.129097 | Yes | Alcohol wash | Yes | Oxalic Acid (Vapor) | Yes | Fondant or sugar candy;Commercially available supplements | none |
| 1313 | 2020 | 2.129097 | Yes | Alcohol wash | Yes | Oxalic Acid (Vapor) | Yes | Fondant or sugar candy;Commercially available supplements | none |
| 1314 | 2020 | 2.129097 | Yes | Alcohol wash | Yes | Oxalic Acid (Vapor) | Yes | Fondant or sugar candy;Commercially available supplements | none |
| 1315 | 2020 | 1.721453 | Yes | Alcohol wash | Yes | Oxalic Acid (Vapor) | Yes | Fondant or sugar candy | sugar only |
| 1316 | 2020 | 1.721453 | Yes | Alcohol wash | Yes | Oxalic Acid (Vapor) | Yes | Fondant or sugar candy | sugar only |
| 1317 | 2020 | 1.721453 | Yes | Alcohol wash | Yes | Oxalic Acid (Vapor) | Yes | Fondant or sugar candy | sugar only |
| 1318 | 2020 | 1.721453 | Yes | Alcohol wash | Yes | Oxalic Acid (Vapor) | Yes | Fondant or sugar candy | sugar only |
| 1319 | 2020 | 1.721453 | Yes | Alcohol wash | Yes | Oxalic Acid (Vapor) | Yes | Fondant or sugar candy | sugar only |
| 1320 | 2020 | 1.721453 | Yes | Alcohol wash | Yes | Oxalic Acid (Vapor) | Yes | Fondant or sugar candy | sugar only |
| 1321 | 2020 | 1.721453 | Yes | Alcohol wash | Yes | Oxalic Acid (Vapor) | Yes | Fondant or sugar candy | sugar only |
| 1322 | 2020 | 1.721453 | Yes | Alcohol wash | Yes | Oxalic Acid (Vapor) | Yes | Fondant or sugar candy | sugar only |
| 1323 | 2020 | 0.693862 | Yes | Sugar roll | Yes | Oxalic Acid (Vapor) | Yes | Dry sugar;Honey from your own stock | none |
| 1324 | 2020 | 0.693862 | Yes | Sugar roll | Yes | Oxalic Acid (Vapor) | Yes | Dry sugar;Honey from your own stock | none |
| 1325 | 2020 | 16.11602 | Yes | Sugar roll | Yes | Apivar (Amitraz) | Yes | Commercially available supplements | none |
| 1326 | 2020 | 2.582198 | No | NA | Yes | Formic Acid (Mite Away Quick Strips) | Yes | Honey from your own stock;Pollen from your own stock | none |
| 1327 | 2020 | 2.582198 | No | NA | Yes | Formic Acid (Mite Away Quick Strips) | Yes | Honey from your own stock;Pollen from your own stock | none |
| 1328 | 2020 | 14.70972 | No | NA | Yes | Oxalic Acid (Dribble) | Yes | Fondant or sugar candy;Pollen substitute | none |
| 1329 | 2020 | 4.102356 | Yes | Drone brood inspection | Yes | Apivar (Amitraz) | Yes | Dry sugar | sugar only |
| 1330 | 2020 | 4.102356 | Yes | Drone brood inspection | Yes | Apivar (Amitraz) | Yes | Dry sugar | sugar only |
| 1331 | 2020 | 4.116684 | Yes | 48 hr drop (sticky board) | Yes | Formic Acid (Mite Away Quick Strips) | Yes | Fondant or sugar candy | sugar only |
| 1332 | 2020 | 4.116684 | Yes | 48 hr drop (sticky board) | Yes | Formic Acid (Mite Away Quick Strips) | Yes | Fondant or sugar candy | sugar only |
| 1333 | 2020 | 3.47886 | Yes | Alcohol wash;Drone brood inspection | Yes | Oxalic Acid (Vapor) | Yes | Dry sugar | sugar only |
| 1334 | 2020 | 3.47886 | Yes | Alcohol wash;Drone brood inspection | Yes | Oxalic Acid (Vapor) | Yes | Dry sugar | sugar only |
| 1335 | 2020 | 3.47886 | Yes | Alcohol wash;Drone brood inspection | Yes | Oxalic Acid (Vapor) | Yes | Dry sugar | sugar only |
| 1336 | 2020 | 3.47886 | Yes | Alcohol wash;Drone brood inspection | Yes | Oxalic Acid (Vapor) | Yes | Dry sugar | sugar only |
| 1337 | 2020 | 3.47886 | Yes | Alcohol wash;Drone brood inspection | Yes | Oxalic Acid (Vapor) | Yes | Dry sugar | sugar only |
| 1338 | 2020 | 3.47886 | Yes | Alcohol wash;Drone brood inspection | Yes | Oxalic Acid (Vapor) | Yes | Dry sugar | sugar only |
| 1339 | 2020 | 3.47886 | Yes | Alcohol wash;Drone brood inspection | Yes | Oxalic Acid (Vapor) | Yes | Dry sugar | sugar only |
| 1340 | 2020 | 3.47886 | Yes | Alcohol wash;Drone brood inspection | Yes | Oxalic Acid (Vapor) | Yes | Dry sugar | sugar only |
| 1341 | 2020 | 3.47886 | Yes | Alcohol wash;Drone brood inspection | Yes | Oxalic Acid (Vapor) | Yes | Dry sugar | sugar only |
| 1342 | 2020 | 3.47886 | Yes | Alcohol wash;Drone brood inspection | Yes | Oxalic Acid (Vapor) | Yes | Dry sugar | sugar only |
| 1343 | 2020 | 3.47886 | Yes | Alcohol wash;Drone brood inspection | Yes | Oxalic Acid (Vapor) | Yes | Dry sugar | sugar only |
| 1344 | 2020 | 3.47886 | Yes | Alcohol wash;Drone brood inspection | Yes | Oxalic Acid (Vapor) | Yes | Dry sugar | sugar only |
| 1345 | 2020 | 8.775075 | No | NA | Yes | Api life Var | Yes | Dry sugar | sugar only |
| 1346 | 2020 | 8.775075 | No | NA | Yes | Api life Var | Yes | Dry sugar | sugar only |
| 1347 | 2020 | 9.844322 | No | NA | Yes | Apivar (Amitraz) | Yes | Pollen substitute | pollen only |
| 1348 | 2020 | 8.484874 | Yes | Sugar roll | Yes | Oxalic Acid (Vapor) | Yes | Fondant or sugar candy;Pollen substitute | none |
| 1349 | 2020 | 8.484874 | Yes | Sugar roll | Yes | Oxalic Acid (Vapor) | Yes | Fondant or sugar candy;Pollen substitute | none |
| 1350 | 2020 | 8.484874 | Yes | Sugar roll | Yes | Oxalic Acid (Vapor) | Yes | Fondant or sugar candy;Pollen substitute | none |
| 1351 | 2020 | 8.484874 | Yes | Sugar roll | Yes | Oxalic Acid (Vapor) | Yes | Fondant or sugar candy;Pollen substitute | none |
| 1352 | 2020 | 2.139483 | No | NA | Yes | Oxalic Acid (Vapor) | Yes | Fondant or sugar candy;Pollen substitute | none |
| 1353 | 2020 | 2.139483 | No | NA | Yes | Oxalic Acid (Vapor) | Yes | Fondant or sugar candy;Pollen substitute | none |
| 1354 | 2020 | 2.139483 | No | NA | Yes | Oxalic Acid (Vapor) | Yes | Fondant or sugar candy;Pollen substitute | none |
| 1355 | 2020 | 2.139483 | No | NA | Yes | Oxalic Acid (Vapor) | Yes | Fondant or sugar candy;Pollen substitute | none |
| 1356 | 2020 | 2.139483 | No | NA | Yes | Oxalic Acid (Vapor) | Yes | Fondant or sugar candy;Pollen substitute | none |
| 1357 | 2020 | 2.139483 | No | NA | Yes | Oxalic Acid (Vapor) | Yes | Fondant or sugar candy;Pollen substitute | none |
| 1358 | 2020 | 2.139483 | No | NA | Yes | Oxalic Acid (Vapor) | Yes | Fondant or sugar candy;Pollen substitute | none |
| 1359 | 2020 | 2.139483 | No | NA | Yes | Oxalic Acid (Vapor) | Yes | Fondant or sugar candy;Pollen substitute | none |
| 1360 | 2020 | 11.90535 | Yes | Alcohol wash;Drone brood inspection | Yes | Formic Acid (Mite Away Quick Strips) | Yes | Fondant or sugar candy | sugar only |
| 1361 | 2020 | 11.90535 | Yes | Alcohol wash;Drone brood inspection | Yes | Formic Acid (Mite Away Quick Strips) | Yes | Fondant or sugar candy | sugar only |
| 1362 | 2020 | 11.90535 | Yes | Alcohol wash;Drone brood inspection | Yes | Formic Acid (Mite Away Quick Strips) | Yes | Fondant or sugar candy | sugar only |
| 1363 | 2020 | 11.90535 | Yes | Alcohol wash;Drone brood inspection | Yes | Formic Acid (Mite Away Quick Strips) | Yes | Fondant or sugar candy | sugar only |
| 1364 | 2020 | 3.14562 | Yes | Alcohol wash;Drone brood inspection | Yes | Formic Acid (Mite Away Quick Strips) | Yes | Fondant or sugar candy | sugar only |
| 1365 | 2020 | 3.14562 | Yes | Alcohol wash;Drone brood inspection | Yes | Formic Acid (Mite Away Quick Strips) | Yes | Fondant or sugar candy | sugar only |
| 1366 | 2020 | 3.14562 | Yes | Alcohol wash;Drone brood inspection | Yes | Formic Acid (Mite Away Quick Strips) | Yes | Fondant or sugar candy | sugar only |
| 1367 | 2020 | 3.14562 | Yes | Alcohol wash;Drone brood inspection | Yes | Formic Acid (Mite Away Quick Strips) | Yes | Fondant or sugar candy | sugar only |
| 1368 | 2020 | 3.14562 | Yes | Alcohol wash;Drone brood inspection | Yes | Formic Acid (Mite Away Quick Strips) | Yes | Fondant or sugar candy | sugar only |
| 1369 | 2020 | 3.14562 | Yes | Alcohol wash;Drone brood inspection | Yes | Formic Acid (Mite Away Quick Strips) | Yes | Fondant or sugar candy | sugar only |
| 1370 | 2020 | 3.14562 | Yes | Alcohol wash;Drone brood inspection | Yes | Formic Acid (Mite Away Quick Strips) | Yes | Fondant or sugar candy | sugar only |
| 1371 | 2020 | 5.135203 | No | NA | Yes | Apivar (Amitraz) | Yes | Dry sugar;Pollen substitute;they had plenty of honey, much still left | none |
| 1372 | 2020 | 2.830042 | No | Novice; have a white board & Would use sugar roll | Yes | Formic Acid (Mite Away Quick Strips) | Yes | Dry sugar | sugar only |
| 1373 | 2020 | 10.42361 | No | NA | Yes | Hopguard | Yes | Honey from your own stock | none |
| 1374 | 2020 | 10.42361 | No | NA | Yes | Hopguard | Yes | Honey from your own stock | none |
| 1375 | 2020 | 7.387166 | Yes | Drone brood inspection | Yes | Oxalic Acid (Vapor) | Yes | Dry sugar;mann lake winter patties | none |
| 1376 | 2020 | 7.387166 | Yes | Drone brood inspection | Yes | Oxalic Acid (Vapor) | Yes | Dry sugar;mann lake winter patties | none |
| 1377 | 2020 | 7.387166 | Yes | Drone brood inspection | Yes | Oxalic Acid (Vapor) | Yes | Dry sugar;mann lake winter patties | none |
| 1378 | 2020 | 7.387166 | Yes | Drone brood inspection | Yes | Oxalic Acid (Vapor) | Yes | Dry sugar;mann lake winter patties | none |
| 1379 | 2020 | 7.387166 | Yes | Drone brood inspection | Yes | Oxalic Acid (Vapor) | Yes | Dry sugar;mann lake winter patties | none |
| 1380 | 2020 | 7.387166 | Yes | Drone brood inspection | Yes | Oxalic Acid (Vapor) | Yes | Dry sugar;mann lake winter patties | none |
| 1381 | 2020 | 7.387166 | Yes | Drone brood inspection | Yes | Oxalic Acid (Vapor) | Yes | Dry sugar;mann lake winter patties | none |
| 1382 | 2020 | 7.387166 | Yes | Drone brood inspection | Yes | Oxalic Acid (Vapor) | Yes | Dry sugar;mann lake winter patties | none |
| 1383 | 2020 | 7.387166 | Yes | Drone brood inspection | Yes | Oxalic Acid (Vapor) | Yes | Dry sugar;mann lake winter patties | none |
| 1384 | 2020 | 7.387166 | Yes | Drone brood inspection | Yes | Oxalic Acid (Vapor) | Yes | Dry sugar;mann lake winter patties | none |
| 1385 | 2020 | 7.387166 | Yes | Drone brood inspection | Yes | Oxalic Acid (Vapor) | Yes | Dry sugar;mann lake winter patties | none |
| 1386 | 2020 | 7.387166 | Yes | Drone brood inspection | Yes | Oxalic Acid (Vapor) | Yes | Dry sugar;mann lake winter patties | none |
| 1387 | 2020 | 7.387166 | Yes | Drone brood inspection | Yes | Oxalic Acid (Vapor) | Yes | Dry sugar;mann lake winter patties | none |
| 1388 | 2020 | 2.803712 | No | NA | Yes | Oxalic Acid (Vapor) | Yes | Fondant or sugar candy | sugar only |
| 1389 | 2020 | 2.803712 | No | NA | Yes | Oxalic Acid (Vapor) | Yes | Fondant or sugar candy | sugar only |
| 1390 | 2020 | 2.803712 | No | NA | Yes | Oxalic Acid (Vapor) | Yes | Fondant or sugar candy | sugar only |
| 1391 | 2020 | 7.817161 | Yes | Alcohol wash | Yes | Formic Acid (Mite Away Quick Strips) | Yes | Fondant or sugar candy | sugar only |
| 1392 | 2020 | 7.817161 | Yes | Alcohol wash | Yes | Formic Acid (Mite Away Quick Strips) | Yes | Fondant or sugar candy | sugar only |
| 1393 | 2020 | 7.817161 | Yes | Alcohol wash | Yes | Formic Acid (Mite Away Quick Strips) | Yes | Fondant or sugar candy | sugar only |
| 1394 | 2020 | 7.817161 | Yes | Alcohol wash | Yes | Formic Acid (Mite Away Quick Strips) | Yes | Fondant or sugar candy | sugar only |
| 1395 | 2020 | 4.687467 | Yes | Alcohol wash | Yes | Oxalic Acid (Vapor) | Yes | Fondant or sugar candy | sugar only |
| 1396 | 2020 | 4.687467 | Yes | Alcohol wash | Yes | Oxalic Acid (Vapor) | Yes | Fondant or sugar candy | sugar only |
| 1397 | 2020 | 4.687467 | Yes | Alcohol wash | Yes | Oxalic Acid (Vapor) | Yes | Fondant or sugar candy | sugar only |
| 1398 | 2020 | 4.687467 | Yes | Alcohol wash | Yes | Oxalic Acid (Vapor) | Yes | Fondant or sugar candy | sugar only |
| 1399 | 2020 | 4.687467 | Yes | Alcohol wash | Yes | Oxalic Acid (Vapor) | Yes | Fondant or sugar candy | sugar only |
| 1400 | 2020 | 4.687467 | Yes | Alcohol wash | Yes | Oxalic Acid (Vapor) | Yes | Fondant or sugar candy | sugar only |
| 1401 | 2020 | 4.687467 | Yes | Alcohol wash | Yes | Oxalic Acid (Vapor) | Yes | Fondant or sugar candy | sugar only |
| 1402 | 2020 | 4.687467 | Yes | Alcohol wash | Yes | Oxalic Acid (Vapor) | Yes | Fondant or sugar candy | sugar only |
| 1403 | 2020 | 4.687467 | Yes | Alcohol wash | Yes | Oxalic Acid (Vapor) | Yes | Fondant or sugar candy | sugar only |
| 1404 | 2020 | 4.687467 | Yes | Alcohol wash | Yes | Oxalic Acid (Vapor) | Yes | Fondant or sugar candy | sugar only |
| 1405 | 2020 | 4.687467 | Yes | Alcohol wash | Yes | Oxalic Acid (Vapor) | Yes | Fondant or sugar candy | sugar only |
| 1406 | 2020 | 4.687467 | Yes | Alcohol wash | Yes | Oxalic Acid (Vapor) | Yes | Fondant or sugar candy | sugar only |
| 1407 | 2020 | 4.687467 | Yes | Alcohol wash | Yes | Oxalic Acid (Vapor) | Yes | Fondant or sugar candy | sugar only |
| 1408 | 2020 | 4.687467 | Yes | Alcohol wash | Yes | Oxalic Acid (Vapor) | Yes | Fondant or sugar candy | sugar only |
| 1409 | 2020 | 4.687467 | Yes | Alcohol wash | Yes | Oxalic Acid (Vapor) | Yes | Fondant or sugar candy | sugar only |
| 1410 | 2020 | 4.687467 | Yes | Alcohol wash | Yes | Oxalic Acid (Vapor) | Yes | Fondant or sugar candy | sugar only |
| 1411 | 2020 | 4.687467 | Yes | Alcohol wash | Yes | Oxalic Acid (Vapor) | Yes | Fondant or sugar candy | sugar only |
| 1412 | 2020 | 4.687467 | Yes | Alcohol wash | Yes | Oxalic Acid (Vapor) | Yes | Fondant or sugar candy | sugar only |
| 1413 | 2020 | 4.687467 | Yes | Alcohol wash | Yes | Oxalic Acid (Vapor) | Yes | Fondant or sugar candy | sugar only |
| 1414 | 2020 | 4.687467 | Yes | Alcohol wash | Yes | Oxalic Acid (Vapor) | Yes | Fondant or sugar candy | sugar only |
| 1415 | 2020 | 4.687467 | Yes | Alcohol wash | Yes | Oxalic Acid (Vapor) | Yes | Fondant or sugar candy | sugar only |
| 1416 | 2020 | 4.687467 | Yes | Alcohol wash | Yes | Oxalic Acid (Vapor) | Yes | Fondant or sugar candy | sugar only |
| 1417 | 2020 | 6.701776 | No | NA | Yes | Formic Acid (Mite Away Quick Strips) | Yes | Fondant or sugar candy;Sugar syrup | none |
| 1418 | 2020 | 6.701776 | No | NA | Yes | Formic Acid (Mite Away Quick Strips) | Yes | Fondant or sugar candy;Sugar syrup | none |
| 1419 | 2020 | 6.701776 | No | NA | Yes | Formic Acid (Mite Away Quick Strips) | Yes | Fondant or sugar candy;Sugar syrup | none |
| 1420 | 2020 | 6.701776 | No | NA | Yes | Formic Acid (Mite Away Quick Strips) | Yes | Fondant or sugar candy;Sugar syrup | none |
| 1421 | 2020 | 15.45185 | Yes | Sugar roll | Yes | Oxalic Acid (Vapor) | Yes | Dry sugar | sugar only |
| 1422 | 2020 | 15.45185 | Yes | Sugar roll | Yes | Oxalic Acid (Vapor) | Yes | Dry sugar | sugar only |
| 1423 | 2020 | 7.464406 | Yes | Alcohol wash | Yes | Formic Acid (Mite Away Quick Strips) | Yes | Fondant or sugar candy | sugar only |
| 1424 | 2020 | 7.464406 | Yes | Alcohol wash | Yes | Formic Acid (Mite Away Quick Strips) | Yes | Fondant or sugar candy | sugar only |
| 1425 | 2020 | 13.82417 | Yes | Alcohol wash | Yes | Apivar (Amitraz) | Yes | Fondant or sugar candy;Pollen substitute;Probiotics | none |
| 1426 | 2020 | 13.82417 | Yes | Alcohol wash | Yes | Apivar (Amitraz) | Yes | Fondant or sugar candy;Pollen substitute;Probiotics | none |
| 1427 | 2020 | 13.82417 | Yes | Alcohol wash | Yes | Apivar (Amitraz) | Yes | Fondant or sugar candy;Pollen substitute;Probiotics | none |
| 1428 | 2020 | 28.23876 | No | NA | Yes | Formic Acid (Mite Away Quick Strips) | Yes | Commercially available supplements | none |
| 1429 | 2020 | 28.23876 | No | NA | Yes | Formic Acid (Mite Away Quick Strips) | Yes | Commercially available supplements | none |
| 1430 | 2020 | 4.386499 | Yes | Sugar roll | Yes | Formic Acid (Mite Away Quick Strips) | Yes | Honey from your own stock | none |
| 1431 | 2020 | 4.386499 | Yes | Sugar roll | Yes | Formic Acid (Mite Away Quick Strips) | Yes | Honey from your own stock | none |
| 1432 | 2020 | 4.386499 | Yes | Sugar roll | Yes | Formic Acid (Mite Away Quick Strips) | Yes | Honey from your own stock | none |
| 1433 | 2020 | 4.386499 | Yes | Sugar roll | Yes | Formic Acid (Mite Away Quick Strips) | Yes | Honey from your own stock | none |
| 1434 | 2020 | 4.386499 | Yes | Sugar roll | Yes | Formic Acid (Mite Away Quick Strips) | Yes | Honey from your own stock | none |
| 1435 | 2020 | 4.386499 | Yes | Sugar roll | Yes | Formic Acid (Mite Away Quick Strips) | Yes | Honey from your own stock | none |
| 1436 | 2020 | 4.386499 | Yes | Sugar roll | Yes | Formic Acid (Mite Away Quick Strips) | Yes | Honey from your own stock | none |
| 1437 | 2020 | 8.787362 | No | NA | Yes | Apivar (Amitraz) | Yes | Honey from your own stock | none |
| 1438 | 2020 | 8.787362 | No | NA | Yes | Apivar (Amitraz) | Yes | Honey from your own stock | none |
| 1439 | 2020 | 20.75427 | Yes | Alcohol wash | Yes | Formic Acid (Mite Away Quick Strips) | Yes | Fondant or sugar candy | sugar only |
| 1440 | 2020 | 16.12601 | Yes | 48 hr drop (sticky board) | Yes | Oxalic Acid (Vapor) | Yes | Fondant or sugar candy;Pollen substitute | none |
| 1441 | 2020 | 16.12601 | Yes | 48 hr drop (sticky board) | Yes | Oxalic Acid (Vapor) | Yes | Fondant or sugar candy;Pollen substitute | none |
| 1442 | 2020 | 16.12601 | Yes | 48 hr drop (sticky board) | Yes | Oxalic Acid (Vapor) | Yes | Fondant or sugar candy;Pollen substitute | none |
| 1443 | 2020 | 16.12601 | Yes | 48 hr drop (sticky board) | Yes | Oxalic Acid (Vapor) | Yes | Fondant or sugar candy;Pollen substitute | none |
| 1444 | 2020 | 16.12601 | Yes | 48 hr drop (sticky board) | Yes | Oxalic Acid (Vapor) | Yes | Fondant or sugar candy;Pollen substitute | none |
| 1445 | 2020 | 16.12601 | Yes | 48 hr drop (sticky board) | Yes | Oxalic Acid (Vapor) | Yes | Fondant or sugar candy;Pollen substitute | none |
| 1446 | 2020 | 12.26317 | Yes | Sugar roll | Yes | Formic Acid (Mite Away Quick Strips) | Yes | Fondant or sugar candy;Dry sugar;Pollen substitute | none |
| 1447 | 2020 | 8.600057 | Yes | 24 hour sticky board | Yes | Oxalic Acid (Vapor) | Yes | Fondant or sugar candy | sugar only |
| 1448 | 2020 | 11.71187 | Yes | Sugar roll | Yes | Oxalic Acid (Dribble) | Yes | Fondant or sugar candy;Pollen substitute | none |
| 1449 | 2020 | 11.71187 | Yes | Sugar roll | Yes | Oxalic Acid (Dribble) | Yes | Fondant or sugar candy;Pollen substitute | none |
| 1450 | 2020 | 11.71187 | Yes | Sugar roll | Yes | Oxalic Acid (Dribble) | Yes | Fondant or sugar candy;Pollen substitute | none |
| 1451 | 2020 | 11.71187 | Yes | Sugar roll | Yes | Oxalic Acid (Dribble) | Yes | Fondant or sugar candy;Pollen substitute | none |
| 1452 | 2020 | 11.71187 | Yes | Sugar roll | Yes | Oxalic Acid (Dribble) | Yes | Fondant or sugar candy;Pollen substitute | none |
| 1453 | 2020 | 11.71187 | Yes | Sugar roll | Yes | Oxalic Acid (Dribble) | Yes | Fondant or sugar candy;Pollen substitute | none |
| 1454 | 2020 | 20.87362 | No | NA | Yes | Formic Acid (Mite Away Quick Strips) | Yes | Fondant or sugar candy | sugar only |
| 1455 | 2020 | 20.87362 | No | NA | Yes | Formic Acid (Mite Away Quick Strips) | Yes | Fondant or sugar candy | sugar only |
| 1456 | 2020 | 0.292809 | Yes | Sugar roll | Yes | Oxalic Acid (Vapor) | Yes | Sugar syrup;Dry sugar | none |
| 1457 | 2020 | 15.71854 | Yes | Non-sticky mite board | Yes | Formic Acid (Mite Away Quick Strips) | Yes | Fondant or sugar candy | sugar only |
| 1458 | 2020 | 15.71854 | Yes | Non-sticky mite board | Yes | Formic Acid (Mite Away Quick Strips) | Yes | Fondant or sugar candy | sugar only |
| 1459 | 2020 | 15.2713 | No | NA | Yes | Api life Var | Yes | Sugar syrup;Pollen substitute | none |
| 1460 | 2020 | 3.817966 | No | NA | Yes | Hopguard | Yes | Honey from your own stock | none |
| 1461 | 2020 | 3.817966 | No | NA | Yes | Hopguard | Yes | Honey from your own stock | none |
| 1462 | 2020 | 3.817966 | No | NA | Yes | Hopguard | Yes | Honey from your own stock | none |
| 1463 | 2020 | 3.817966 | No | NA | Yes | Hopguard | Yes | Honey from your own stock | none |
| 1464 | 2020 | 3.817966 | No | NA | Yes | Hopguard | Yes | Honey from your own stock | none |
| 1465 | 2020 | 3.817966 | No | NA | Yes | Hopguard | Yes | Honey from your own stock | none |
| 1466 | 2020 | 3.817966 | No | NA | Yes | Hopguard | Yes | Honey from your own stock | none |
| 1467 | 2020 | 21.79751 | Yes | Grid board | Yes | Apivar (Amitraz) | Yes | Sugar syrup;Pollen substitute | none |
| 1468 | 2020 | 21.79751 | Yes | Grid board | Yes | Apivar (Amitraz) | Yes | Sugar syrup;Pollen substitute | none |
| 1469 | 2020 | 21.79751 | Yes | Grid board | Yes | Apivar (Amitraz) | Yes | Sugar syrup;Pollen substitute | none |
| 1470 | 2020 | 2.705462 | Yes | Alcohol wash | Yes | Formic Acid (Mite Away Quick Strips) | Yes | Dry sugar | sugar only |
| 1471 | 2020 | 2.705462 | Yes | Alcohol wash | Yes | Formic Acid (Mite Away Quick Strips) | Yes | Dry sugar | sugar only |
| 1472 | 2020 | 2.705462 | Yes | Alcohol wash | Yes | Formic Acid (Mite Away Quick Strips) | Yes | Dry sugar | sugar only |
| 1473 | 2020 | 2.705462 | Yes | Alcohol wash | Yes | Formic Acid (Mite Away Quick Strips) | Yes | Dry sugar | sugar only |
| 1474 | 2020 | 2.705462 | Yes | Alcohol wash | Yes | Formic Acid (Mite Away Quick Strips) | Yes | Dry sugar | sugar only |
| 1475 | 2020 | 2.705462 | Yes | Alcohol wash | Yes | Formic Acid (Mite Away Quick Strips) | Yes | Dry sugar | sugar only |
| 1476 | 2020 | 2.705462 | Yes | Alcohol wash | Yes | Formic Acid (Mite Away Quick Strips) | Yes | Dry sugar | sugar only |
| 1477 | 2020 | 2.705462 | Yes | Alcohol wash | Yes | Formic Acid (Mite Away Quick Strips) | Yes | Dry sugar | sugar only |
| 1478 | 2020 | 2.705462 | Yes | Alcohol wash | Yes | Formic Acid (Mite Away Quick Strips) | Yes | Dry sugar | sugar only |
| 1479 | 2020 | 2.705462 | Yes | Alcohol wash | Yes | Formic Acid (Mite Away Quick Strips) | Yes | Dry sugar | sugar only |
| 1480 | 2020 | 40.5057 | No | NA | Yes | Apiguard | Yes | Fondant or sugar candy | sugar only |
| 1481 | 2020 | 40.5057 | No | NA | Yes | Apiguard | Yes | Fondant or sugar candy | sugar only |
| 1482 | 2020 | 36.86547 | Yes | Drone brood inspection | Yes | Hopguard | Yes | Fondant or sugar candy | sugar only |
| 1483 | 2020 | 36.86547 | Yes | Drone brood inspection | Yes | Hopguard | Yes | Fondant or sugar candy | sugar only |
| 1484 | 2020 | 1.5032 | No | NA | Yes | Oxalic Acid (Vapor) | Yes | Dry sugar | sugar only |
| 1485 | 2020 | 1.5032 | No | NA | Yes | Oxalic Acid (Vapor) | Yes | Dry sugar | sugar only |
| 1486 | 2020 | 41.66911 | Yes | Sugar roll | Yes | Oxalic Acid (Vapor) | Yes | Dry sugar | sugar only |
| 1487 | 2020 | 41.66911 | Yes | Sugar roll | Yes | Oxalic Acid (Vapor) | Yes | Dry sugar | sugar only |
| 1488 | 2020 | 41.66911 | Yes | Sugar roll | Yes | Oxalic Acid (Vapor) | Yes | Dry sugar | sugar only |
| 1489 | 2020 | 41.66911 | Yes | Sugar roll | Yes | Oxalic Acid (Vapor) | Yes | Dry sugar | sugar only |
| 1490 | 2020 | 41.66911 | Yes | Sugar roll | Yes | Oxalic Acid (Vapor) | Yes | Dry sugar | sugar only |
| 1491 | 2020 | 41.66911 | Yes | Sugar roll | Yes | Oxalic Acid (Vapor) | Yes | Dry sugar | sugar only |
| 1492 | 2020 | 41.66911 | Yes | Sugar roll | Yes | Oxalic Acid (Vapor) | Yes | Dry sugar | sugar only |
| 1493 | 2020 | 23.60663 | Yes | Alcohol wash | Yes | Formic Acid (Mite Away Quick Strips) | Yes | Fondant or sugar candy | sugar only |
| 1494 | 2020 | 23.60663 | Yes | Alcohol wash | Yes | Formic Acid (Mite Away Quick Strips) | Yes | Fondant or sugar candy | sugar only |
| 1495 | 2020 | 23.60663 | Yes | Alcohol wash | Yes | Formic Acid (Mite Away Quick Strips) | Yes | Fondant or sugar candy | sugar only |
| 1496 | 2020 | 23.60663 | Yes | Alcohol wash | Yes | Formic Acid (Mite Away Quick Strips) | Yes | Fondant or sugar candy | sugar only |
| 1497 | 2020 | 9.804245 | Yes | Alcohol wash | Yes | Oxalic Acid (Vapor) | Yes | Honey from your own stock;Homemade fondant with pollen substitute | none |
| 1498 | 2020 | 9.804245 | Yes | Alcohol wash | Yes | Oxalic Acid (Vapor) | Yes | Honey from your own stock;Homemade fondant with pollen substitute | none |
| 1499 | 2020 | 9.804245 | Yes | Alcohol wash | Yes | Oxalic Acid (Vapor) | Yes | Honey from your own stock;Homemade fondant with pollen substitute | none |
| 1500 | 2020 | 9.804245 | Yes | Alcohol wash | Yes | Oxalic Acid (Vapor) | Yes | Honey from your own stock;Homemade fondant with pollen substitute | none |
|  |  |  |  |  |  |  |  |  |  |
| 1501 | 2020 | 9.804245 | Yes | Alcohol wash | Yes | Oxalic Acid (Vapor) | Yes | Honey from your own stock;Homemade fondant with pollen substitute | none |
| 1502 | 2020 | 9.804245 | Yes | Alcohol wash | Yes | Oxalic Acid (Vapor) | Yes | Honey from your own stock;Homemade fondant with pollen substitute | none |
| 1503 | 2020 | 9.804245 | Yes | Alcohol wash | Yes | Oxalic Acid (Vapor) | Yes | Honey from your own stock;Homemade fondant with pollen substitute | none |
| 1504 | 2020 | 9.804245 | Yes | Alcohol wash | Yes | Oxalic Acid (Vapor) | Yes | Honey from your own stock;Homemade fondant with pollen substitute | none |
| 1505 | 2020 | 9.804245 | Yes | Alcohol wash | Yes | Oxalic Acid (Vapor) | Yes | Honey from your own stock;Homemade fondant with pollen substitute | none |
| 1506 | 2020 | 9.804245 | Yes | Alcohol wash | Yes | Oxalic Acid (Vapor) | Yes | Honey from your own stock;Homemade fondant with pollen substitute | none |
| 1507 | 2020 | 9.804245 | Yes | Alcohol wash | Yes | Oxalic Acid (Vapor) | Yes | Honey from your own stock;Homemade fondant with pollen substitute | none |
| 1508 | 2020 | 3.811456 | No | NA | Yes | Oxalic Acid (Vapor) | Yes | Fondant or sugar candy;Honey from your own stock | none |
| 1509 | 2020 | 3.811456 | No | NA | Yes | Oxalic Acid (Vapor) | Yes | Fondant or sugar candy;Honey from your own stock | none |
| 1510 | 2020 | 3.314979 | Yes | 48 hr drop (sticky board) | Yes | Formic Acid (Mite Away Quick Strips) | Yes | Fondant or sugar candy;Honey from your own stock | none |
| 1511 | 2020 | 3.314979 | Yes | 48 hr drop (sticky board) | Yes | Formic Acid (Mite Away Quick Strips) | Yes | Fondant or sugar candy;Honey from your own stock | none |
| 1512 | 2020 | 3.314979 | Yes | 48 hr drop (sticky board) | Yes | Formic Acid (Mite Away Quick Strips) | Yes | Fondant or sugar candy;Honey from your own stock | none |
| 1513 | 2020 | 34.33422 | Yes | Sugar roll | Yes | Oxalic Acid (Dribble) | Yes | Sugar syrup;Dry sugar;Pollen substitute;Probiotics | none |
| 1514 | 2020 | 45.98718 | Yes | Sugar roll;Drone brood inspection | Yes | Formic Acid (Mite Away Quick Strips) | Yes | Fondant or sugar candy;Protein patties | none |
| 1515 | 2020 | 45.98718 | Yes | Sugar roll;Drone brood inspection | Yes | Formic Acid (Mite Away Quick Strips) | Yes | Fondant or sugar candy;Protein patties | none |
| 1516 | 2020 | 11.94674 | Yes | Sugar roll | Yes | Hopguard | Yes | Sugar syrup | none |
| 1517 | 2020 | 1.407017 | Yes | NA | Yes | Oxalic Acid (Vapor) | Yes | Fondant or sugar candy | sugar only |
| 1518 | 2020 | 1.407017 | Yes | NA | Yes | Oxalic Acid (Vapor) | Yes | Fondant or sugar candy | sugar only |
| 1519 | 2020 | 1.407017 | Yes | NA | Yes | Oxalic Acid (Vapor) | Yes | Fondant or sugar candy | sugar only |
| 1520 | 2020 | 1.407017 | Yes | NA | Yes | Oxalic Acid (Vapor) | Yes | Fondant or sugar candy | sugar only |
| 1521 | 2020 | 1.407017 | Yes | NA | Yes | Oxalic Acid (Vapor) | Yes | Fondant or sugar candy | sugar only |
| 1522 | 2020 | 1.407017 | Yes | NA | Yes | Oxalic Acid (Vapor) | Yes | Fondant or sugar candy | sugar only |
| 1523 | 2020 | 7.653792 | Yes | 48 hr drop (sticky board) | Yes | Formic Acid (Mite Away Quick Strips) | Yes | Fondant or sugar candy;Dry sugar;Honey from your own stock | none |
| 1524 | 2020 | 7.653792 | Yes | 48 hr drop (sticky board) | Yes | Formic Acid (Mite Away Quick Strips) | Yes | Fondant or sugar candy;Dry sugar;Honey from your own stock | none |
| 1525 | 2020 | 21.70611 | Yes | 48 hr drop (sticky board);Drone brood inspection | Yes | Apivar (Amitraz) | Yes | Sugar syrup | none |
| 1526 | 2020 | 21.70611 | Yes | 48 hr drop (sticky board);Drone brood inspection | Yes | Apivar (Amitraz) | Yes | Sugar syrup | none |
| 1527 | 2020 | 2.119636 | Yes | Sugar roll | Yes | Oxalic Acid (Vapor) | Yes | Fondant or sugar candy;Honey from your own stock;Pollen from your own stock | none |
| 1528 | 2020 | 2.119636 | Yes | Sugar roll | Yes | Oxalic Acid (Vapor) | Yes | Fondant or sugar candy;Honey from your own stock;Pollen from your own stock | none |
| 1529 | 2020 | 2.119636 | Yes | Sugar roll | Yes | Oxalic Acid (Vapor) | Yes | Fondant or sugar candy;Honey from your own stock;Pollen from your own stock | none |
| 1530 | 2020 | 0.680592 | Yes | Sugar roll | Yes | Formic Acid (Mite Away Quick Strips) | Yes | Fondant or sugar candy;Sugar syrup;Honey from your own stock | none |
| 1531 | 2020 | 0.680592 | Yes | Sugar roll | Yes | Formic Acid (Mite Away Quick Strips) | Yes | Fondant or sugar candy;Sugar syrup;Honey from your own stock | none |
| 1532 | 2020 | 0.680592 | Yes | Sugar roll | Yes | Formic Acid (Mite Away Quick Strips) | Yes | Fondant or sugar candy;Sugar syrup;Honey from your own stock | none |
| 1533 | 2020 | 0.680592 | Yes | Sugar roll | Yes | Formic Acid (Mite Away Quick Strips) | Yes | Fondant or sugar candy;Sugar syrup;Honey from your own stock | none |
| 1534 | 2020 | 0.680592 | Yes | Sugar roll | Yes | Formic Acid (Mite Away Quick Strips) | Yes | Fondant or sugar candy;Sugar syrup;Honey from your own stock | none |
| 1535 | 2020 | 0.680592 | Yes | Sugar roll | Yes | Formic Acid (Mite Away Quick Strips) | Yes | Fondant or sugar candy;Sugar syrup;Honey from your own stock | none |
| 1536 | 2020 | 0.680592 | Yes | Sugar roll | Yes | Formic Acid (Mite Away Quick Strips) | Yes | Fondant or sugar candy;Sugar syrup;Honey from your own stock | none |
| 1537 | 2020 | 0.680592 | Yes | Sugar roll | Yes | Formic Acid (Mite Away Quick Strips) | Yes | Fondant or sugar candy;Sugar syrup;Honey from your own stock | none |
| 1538 | 2020 | 2.394984 | Yes | Alcohol wash;Also was shown sugar roll on my bees | Yes | Formic Acid (Mite Away Quick Strips) | Yes | Dry sugar | sugar only |
| 1539 | 2020 | 2.394984 | Yes | Alcohol wash;Also was shown sugar roll on my bees | Yes | Formic Acid (Mite Away Quick Strips) | Yes | Dry sugar | sugar only |
| 1540 | 2020 | 2.394984 | Yes | Alcohol wash;Also was shown sugar roll on my bees | Yes | Formic Acid (Mite Away Quick Strips) | Yes | Dry sugar | sugar only |
| 1541 | 2020 | 0.989914 | Yes | Sugar roll | Yes | Formic Acid (Mite Away Quick Strips) | Yes | Fondant or sugar candy;Pollen substitute;Commercially available supplements | none |
| 1542 | 2020 | 0.989914 | Yes | Sugar roll | Yes | Formic Acid (Mite Away Quick Strips) | Yes | Fondant or sugar candy;Pollen substitute;Commercially available supplements | none |
| 1543 | 2020 | 0.989914 | Yes | Sugar roll | Yes | Formic Acid (Mite Away Quick Strips) | Yes | Fondant or sugar candy;Pollen substitute;Commercially available supplements | none |
| 1544 | 2020 | 5.013378 | Yes | 48 hr drop (sticky board) | Yes | Oxalic Acid (Vapor) | Yes | Fondant or sugar candy;Honey from your own stock | none |
| 1545 | 2020 | 5.013378 | Yes | 48 hr drop (sticky board) | Yes | Oxalic Acid (Vapor) | Yes | Fondant or sugar candy;Honey from your own stock | none |
| 1546 | 2020 | 5.013378 | Yes | 48 hr drop (sticky board) | Yes | Oxalic Acid (Vapor) | Yes | Fondant or sugar candy;Honey from your own stock | none |
| 1547 | 2020 | 5.013378 | Yes | 48 hr drop (sticky board) | Yes | Oxalic Acid (Vapor) | Yes | Fondant or sugar candy;Honey from your own stock | none |
| 1548 | 2020 | 5.013378 | Yes | 48 hr drop (sticky board) | Yes | Oxalic Acid (Vapor) | Yes | Fondant or sugar candy;Honey from your own stock | none |
| 1549 | 2020 | 5.013378 | Yes | 48 hr drop (sticky board) | Yes | Oxalic Acid (Vapor) | Yes | Fondant or sugar candy;Honey from your own stock | none |
| 1550 | 2020 | 5.013378 | Yes | 48 hr drop (sticky board) | Yes | Oxalic Acid (Vapor) | Yes | Fondant or sugar candy;Honey from your own stock | none |
| 1551 | 2020 | 5.013378 | Yes | 48 hr drop (sticky board) | Yes | Oxalic Acid (Vapor) | Yes | Fondant or sugar candy;Honey from your own stock | none |
| 1552 | 2020 | 1.473507 | Yes | Sugar roll | Yes | Formic Acid (Mite Away Quick Strips) | Yes | Fondant or sugar candy | sugar only |
| 1553 | 2020 | 1.473507 | Yes | Sugar roll | Yes | Formic Acid (Mite Away Quick Strips) | Yes | Fondant or sugar candy | sugar only |
| 1554 | 2020 | 0.988075 | Yes | Alcohol wash | Yes | Oxalic Acid (Vapor) | Yes | Dry sugar | sugar only |
| 1555 | 2020 | 0.988075 | Yes | Alcohol wash | Yes | Oxalic Acid (Vapor) | Yes | Dry sugar | sugar only |
| 1556 | 2020 | 0.988075 | Yes | Alcohol wash | Yes | Oxalic Acid (Vapor) | Yes | Dry sugar | sugar only |
| 1557 | 2020 | 0.988075 | Yes | Alcohol wash | Yes | Oxalic Acid (Vapor) | Yes | Dry sugar | sugar only |
| 1558 | 2020 | 0.988075 | Yes | Alcohol wash | Yes | Oxalic Acid (Vapor) | Yes | Dry sugar | sugar only |
| 1559 | 2020 | 0.988075 | Yes | Alcohol wash | Yes | Oxalic Acid (Vapor) | Yes | Dry sugar | sugar only |
| 1560 | 2020 | 0.988075 | Yes | Alcohol wash | Yes | Oxalic Acid (Vapor) | Yes | Dry sugar | sugar only |
| 1561 | 2020 | 0.988075 | Yes | Alcohol wash | Yes | Oxalic Acid (Vapor) | Yes | Dry sugar | sugar only |
| 1562 | 2020 | 0.988075 | Yes | Alcohol wash | Yes | Oxalic Acid (Vapor) | Yes | Dry sugar | sugar only |
| 1563 | 2020 | 0.988075 | Yes | Alcohol wash | Yes | Oxalic Acid (Vapor) | Yes | Dry sugar | sugar only |
| 1564 | 2020 | 0.988075 | Yes | Alcohol wash | Yes | Oxalic Acid (Vapor) | Yes | Dry sugar | sugar only |
| 1565 | 2020 | 0.988075 | Yes | Alcohol wash | Yes | Oxalic Acid (Vapor) | Yes | Dry sugar | sugar only |
| 1566 | 2020 | 7.336563 | Yes | Sugar roll | Yes | Formic Acid (Mite Away Quick Strips) | Yes | Sugar syrup | none |
| 1567 | 2020 | 7.336563 | Yes | Sugar roll | Yes | Formic Acid (Mite Away Quick Strips) | Yes | Sugar syrup | none |
| 1568 | 2020 | 7.336563 | Yes | Sugar roll | Yes | Formic Acid (Mite Away Quick Strips) | Yes | Sugar syrup | none |
| 1569 | 2020 | 7.336563 | Yes | Sugar roll | Yes | Formic Acid (Mite Away Quick Strips) | Yes | Sugar syrup | none |
| 1570 | 2020 | 24.6416 | Yes | Alcohol wash | Yes | Formic Acid (Mite Away Quick Strips) | Yes | Fondant or sugar candy | sugar only |
| 1571 | 2020 | 24.6416 | Yes | Alcohol wash | Yes | Formic Acid (Mite Away Quick Strips) | Yes | Fondant or sugar candy | sugar only |
| 1572 | 2021 | 7.84852 | No | NA | Yes | Oxalic Acid (Vapor) | Yes | Honey from your own stock | none |
| 1573 | 2021 | 1.686579 | Yes | Alcohol wash | Yes | Formic Acid (Mite Away Quick Strips) | Yes | Fondant or sugar candy | sugar only |
| 1574 | 2021 | 1.686579 | Yes | Alcohol wash | Yes | Formic Acid (Mite Away Quick Strips) | Yes | Fondant or sugar candy | sugar only |
| 1575 | 2021 | 1.686579 | Yes | Alcohol wash | Yes | Formic Acid (Mite Away Quick Strips) | Yes | Fondant or sugar candy | sugar only |
| 1576 | 2021 | 8.054671 | Yes | Alcohol wash | Yes | Oxalic Acid (Vapor) | Yes | Fondant or sugar candy, Dry sugar | none |
| 1577 | 2021 | 8.054671 | Yes | Alcohol wash | Yes | Oxalic Acid (Vapor) | Yes | Fondant or sugar candy, Dry sugar | none |
| 1578 | 2021 | 8.054671 | Yes | Alcohol wash | Yes | Oxalic Acid (Vapor) | Yes | Fondant or sugar candy, Dry sugar | none |
| 1579 | 2021 | 8.054671 | Yes | Alcohol wash | Yes | Oxalic Acid (Vapor) | Yes | Fondant or sugar candy, Dry sugar | none |
| 1580 | 2021 | 8.054671 | Yes | Alcohol wash | Yes | Oxalic Acid (Vapor) | Yes | Fondant or sugar candy, Dry sugar | none |
| 1581 | 2021 | 8.054671 | Yes | Alcohol wash | Yes | Oxalic Acid (Vapor) | Yes | Fondant or sugar candy, Dry sugar | none |
| 1582 | 2021 | 8.054671 | Yes | Alcohol wash | Yes | Oxalic Acid (Vapor) | Yes | Fondant or sugar candy, Dry sugar | none |
| 1583 | 2021 | 8.054671 | Yes | Alcohol wash | Yes | Oxalic Acid (Vapor) | Yes | Fondant or sugar candy, Dry sugar | none |
| 1584 | 2021 | 8.054671 | Yes | Alcohol wash | Yes | Oxalic Acid (Vapor) | Yes | Fondant or sugar candy, Dry sugar | none |
| 1585 | 2021 | 8.223008 | Yes | Sugar roll | Yes | Formic Acid (Mite Away Quick Strips) | Yes | Dry sugar, Pollen substitute | none |
| 1586 | 2021 | 8.223008 | Yes | Sugar roll | Yes | Formic Acid (Mite Away Quick Strips) | Yes | Dry sugar, Pollen substitute | none |
| 1587 | 2021 | 8.223008 | Yes | Sugar roll | Yes | Formic Acid (Mite Away Quick Strips) | Yes | Dry sugar, Pollen substitute | none |
| 1588 | 2021 | 8.223008 | Yes | Sugar roll | Yes | Formic Acid (Mite Away Quick Strips) | Yes | Dry sugar, Pollen substitute | none |
| 1589 | 2021 | 0.811353 | Yes | Sugar roll | Yes | Api life Var | Yes | Fondant or sugar candy, Sugar syrup, Pollen substitute | none |
| 1590 | 2021 | 1.733222 | Yes | Sugar roll | Yes | Oxalic Acid (Vapor) | Yes | Fondant or sugar candy | sugar only |
| 1591 | 2021 | 0.454739 | Yes | Sugar roll | Yes | Formic Acid (Mite Away Quick Strips) | Yes | Fondant or sugar candy, Dry sugar, Pollen substitute | none |
| 1592 | 2021 | 0.454739 | Yes | Sugar roll | Yes | Formic Acid (Mite Away Quick Strips) | Yes | Fondant or sugar candy, Dry sugar, Pollen substitute | none |
| 1593 | 2021 | 0.454739 | Yes | Sugar roll | Yes | Formic Acid (Mite Away Quick Strips) | Yes | Fondant or sugar candy, Dry sugar, Pollen substitute | none |
| 1594 | 2021 | 10.58146 | Yes | 48 hr drop (sticky board) | Yes | Formic Acid (Mite Away Quick Strips) | Yes | Sugar syrup, Pollen substitute | none |
| 1595 | 2021 | 8.294543 | Yes | Sugar roll, Alcohol wash | Yes | Oxalic Acid (Dribble) | Yes | Fondant or sugar candy, Honey from your own stock | none |
| 1596 | 2021 | 8.294543 | Yes | Sugar roll, Alcohol wash | Yes | Oxalic Acid (Dribble) | Yes | Fondant or sugar candy, Honey from your own stock | none |
| 1597 | 2021 | 8.294543 | Yes | Sugar roll, Alcohol wash | Yes | Oxalic Acid (Dribble) | Yes | Fondant or sugar candy, Honey from your own stock | none |
| 1598 | 2021 | 16.0595 | Yes | 48 hr drop (sticky board) | Yes | Oxalic Acid (Vapor) | Yes | Fondant or sugar candy | sugar only |
| 1599 | 2021 | 16.0595 | Yes | 48 hr drop (sticky board) | Yes | Oxalic Acid (Vapor) | Yes | Fondant or sugar candy | sugar only |
| 1600 | 2021 | 16.0595 | Yes | 48 hr drop (sticky board) | Yes | Oxalic Acid (Vapor) | Yes | Fondant or sugar candy | sugar only |
| 1601 | 2021 | 16.0595 | Yes | 48 hr drop (sticky board) | Yes | Oxalic Acid (Vapor) | Yes | Fondant or sugar candy | sugar only |
| 1602 | 2021 | 16.0595 | Yes | 48 hr drop (sticky board) | Yes | Oxalic Acid (Vapor) | Yes | Fondant or sugar candy | sugar only |
| 1603 | 2021 | 16.0595 | Yes | 48 hr drop (sticky board) | Yes | Oxalic Acid (Vapor) | Yes | Fondant or sugar candy | sugar only |
| 1604 | 2021 | 16.0595 | Yes | 48 hr drop (sticky board) | Yes | Oxalic Acid (Vapor) | Yes | Fondant or sugar candy | sugar only |
| 1605 | 2021 | 10.54058 | No | NA | Yes | Formic Acid (Mite Away Quick Strips) | Yes | Fondant or sugar candy | sugar only |
| 1606 | 2021 | 10.54058 | No | NA | Yes | Formic Acid (Mite Away Quick Strips) | Yes | Fondant or sugar candy | sugar only |
| 1607 | 2021 | 2.214002 | Yes | Drone brood inspection | Yes | Oxalic Acid (Vapor) | Yes | Fondant or sugar candy, Pollen substitute, Honey from your own stock | none |
| 1608 | 2021 | 2.214002 | Yes | Drone brood inspection | Yes | Oxalic Acid (Vapor) | Yes | Fondant or sugar candy, Pollen substitute, Honey from your own stock | none |
| 1609 | 2021 | 2.214002 | Yes | Drone brood inspection | Yes | Oxalic Acid (Vapor) | Yes | Fondant or sugar candy, Pollen substitute, Honey from your own stock | none |
| 1610 | 2021 | 18.67701 | Yes | Alcohol wash | Yes | Formic Acid (Mite Away Quick Strips) | Yes | Dry sugar | sugar only |
| 1611 | 2021 | 22.58061 | Yes | Sugar roll | Yes | Formic Acid (Mite Away Quick Strips) | Yes | Fondant or sugar candy | sugar only |
| 1612 | 2021 | 22.58061 | Yes | Sugar roll | Yes | Formic Acid (Mite Away Quick Strips) | Yes | Fondant or sugar candy | sugar only |
| 1613 | 2021 | 22.58061 | Yes | Sugar roll | Yes | Formic Acid (Mite Away Quick Strips) | Yes | Fondant or sugar candy | sugar only |
| 1614 | 2021 | 22.58061 | Yes | Sugar roll | Yes | Formic Acid (Mite Away Quick Strips) | Yes | Fondant or sugar candy | sugar only |
| 1615 | 2021 | 22.58061 | Yes | Sugar roll | Yes | Formic Acid (Mite Away Quick Strips) | Yes | Fondant or sugar candy | sugar only |
| 1616 | 2021 | 22.58061 | Yes | Sugar roll | Yes | Formic Acid (Mite Away Quick Strips) | Yes | Fondant or sugar candy | sugar only |
| 1617 | 2021 | 1.191253 | Yes | Alcohol wash | Yes | Formic Acid (Mite Away Quick Strips) | Yes | Fondant or sugar candy | sugar only |
| 1618 | 2021 | 1.191253 | Yes | Alcohol wash | Yes | Formic Acid (Mite Away Quick Strips) | Yes | Fondant or sugar candy | sugar only |
| 1619 | 2021 | 1.191253 | Yes | Alcohol wash | Yes | Formic Acid (Mite Away Quick Strips) | Yes | Fondant or sugar candy | sugar only |
| 1620 | 2021 | 1.191253 | Yes | Alcohol wash | Yes | Formic Acid (Mite Away Quick Strips) | Yes | Fondant or sugar candy | sugar only |
| 1621 | 2021 | 1.191253 | Yes | Alcohol wash | Yes | Formic Acid (Mite Away Quick Strips) | Yes | Fondant or sugar candy | sugar only |
| 1622 | 2021 | 1.191253 | Yes | Alcohol wash | Yes | Formic Acid (Mite Away Quick Strips) | Yes | Fondant or sugar candy | sugar only |
| 1623 | 2021 | 1.191253 | Yes | Alcohol wash | Yes | Formic Acid (Mite Away Quick Strips) | Yes | Fondant or sugar candy | sugar only |
| 1624 | 2021 | 1.191253 | Yes | Alcohol wash | Yes | Formic Acid (Mite Away Quick Strips) | Yes | Fondant or sugar candy | sugar only |
| 1625 | 2021 | 1.191253 | Yes | Alcohol wash | Yes | Formic Acid (Mite Away Quick Strips) | Yes | Fondant or sugar candy | sugar only |
| 1626 | 2021 | 1.191253 | Yes | Alcohol wash | Yes | Formic Acid (Mite Away Quick Strips) | Yes | Fondant or sugar candy | sugar only |
| 1627 | 2021 | 1.191253 | Yes | Alcohol wash | Yes | Formic Acid (Mite Away Quick Strips) | Yes | Fondant or sugar candy | sugar only |
| 1628 | 2021 | 1.191253 | Yes | Alcohol wash | Yes | Formic Acid (Mite Away Quick Strips) | Yes | Fondant or sugar candy | sugar only |
| 1629 | 2021 | 1.191253 | Yes | Alcohol wash | Yes | Formic Acid (Mite Away Quick Strips) | Yes | Fondant or sugar candy | sugar only |
| 1630 | 2021 | 1.191253 | Yes | Alcohol wash | Yes | Formic Acid (Mite Away Quick Strips) | Yes | Fondant or sugar candy | sugar only |
| 1631 | 2021 | 1.191253 | Yes | Alcohol wash | Yes | Formic Acid (Mite Away Quick Strips) | Yes | Fondant or sugar candy | sugar only |
| 1632 | 2021 | 1.191253 | Yes | Alcohol wash | Yes | Formic Acid (Mite Away Quick Strips) | Yes | Fondant or sugar candy | sugar only |
| 1633 | 2021 | 1.191253 | Yes | Alcohol wash | Yes | Formic Acid (Mite Away Quick Strips) | Yes | Fondant or sugar candy | sugar only |
| 1634 | 2021 | 1.191253 | Yes | Alcohol wash | Yes | Formic Acid (Mite Away Quick Strips) | Yes | Fondant or sugar candy | sugar only |
| 1635 | 2021 | 1.191253 | Yes | Alcohol wash | Yes | Formic Acid (Mite Away Quick Strips) | Yes | Fondant or sugar candy | sugar only |
| 1636 | 2021 | 1.191253 | Yes | Alcohol wash | Yes | Formic Acid (Mite Away Quick Strips) | Yes | Fondant or sugar candy | sugar only |
| 1637 | 2021 | 1.191253 | Yes | Alcohol wash | Yes | Formic Acid (Mite Away Quick Strips) | Yes | Fondant or sugar candy | sugar only |
| 1638 | 2021 | 1.191253 | Yes | Alcohol wash | Yes | Formic Acid (Mite Away Quick Strips) | Yes | Fondant or sugar candy | sugar only |
| 1639 | 2021 | 1.191253 | Yes | Alcohol wash | Yes | Formic Acid (Mite Away Quick Strips) | Yes | Fondant or sugar candy | sugar only |
| 1640 | 2021 | 1.191253 | Yes | Alcohol wash | Yes | Formic Acid (Mite Away Quick Strips) | Yes | Fondant or sugar candy | sugar only |
| 1641 | 2021 | 1.191253 | Yes | Alcohol wash | Yes | Formic Acid (Mite Away Quick Strips) | Yes | Fondant or sugar candy | sugar only |
| 1642 | 2021 | 1.191253 | Yes | Alcohol wash | Yes | Formic Acid (Mite Away Quick Strips) | Yes | Fondant or sugar candy | sugar only |
| 1643 | 2021 | 1.191253 | Yes | Alcohol wash | Yes | Formic Acid (Mite Away Quick Strips) | Yes | Fondant or sugar candy | sugar only |
| 1644 | 2021 | 1.191253 | Yes | Alcohol wash | Yes | Formic Acid (Mite Away Quick Strips) | Yes | Fondant or sugar candy | sugar only |
| 1645 | 2021 | 1.191253 | Yes | Alcohol wash | Yes | Formic Acid (Mite Away Quick Strips) | Yes | Fondant or sugar candy | sugar only |
| 1646 | 2021 | 1.191253 | Yes | Alcohol wash | Yes | Formic Acid (Mite Away Quick Strips) | Yes | Fondant or sugar candy | sugar only |
| 1647 | 2021 | 1.191253 | Yes | Alcohol wash | Yes | Formic Acid (Mite Away Quick Strips) | Yes | Fondant or sugar candy | sugar only |
| 1648 | 2021 | 1.191253 | Yes | Alcohol wash | Yes | Formic Acid (Mite Away Quick Strips) | Yes | Fondant or sugar candy | sugar only |
| 1649 | 2021 | 1.191253 | Yes | Alcohol wash | Yes | Formic Acid (Mite Away Quick Strips) | Yes | Fondant or sugar candy | sugar only |
| 1650 | 2021 | 1.191253 | Yes | Alcohol wash | Yes | Formic Acid (Mite Away Quick Strips) | Yes | Fondant or sugar candy | sugar only |
| 1651 | 2021 | 1.191253 | Yes | Alcohol wash | Yes | Formic Acid (Mite Away Quick Strips) | Yes | Fondant or sugar candy | sugar only |
| 1652 | 2021 | 1.191253 | Yes | Alcohol wash | Yes | Formic Acid (Mite Away Quick Strips) | Yes | Fondant or sugar candy | sugar only |
| 1653 | 2021 | 1.191253 | Yes | Alcohol wash | Yes | Formic Acid (Mite Away Quick Strips) | Yes | Fondant or sugar candy | sugar only |
| 1654 | 2021 | 1.191253 | Yes | Alcohol wash | Yes | Formic Acid (Mite Away Quick Strips) | Yes | Fondant or sugar candy | sugar only |
| 1655 | 2021 | 1.191253 | Yes | Alcohol wash | Yes | Formic Acid (Mite Away Quick Strips) | Yes | Fondant or sugar candy | sugar only |
| 1656 | 2021 | 1.191253 | Yes | Alcohol wash | Yes | Formic Acid (Mite Away Quick Strips) | Yes | Fondant or sugar candy | sugar only |
| 1657 | 2021 | 3.415175 | Yes | NA | Yes | Apistan | Yes | Fondant or sugar candy, Pollen substitute | none |
| 1658 | 2021 | 3.415175 | Yes | NA | Yes | Apistan | Yes | Fondant or sugar candy, Pollen substitute | none |
| 1659 | 2021 | 3.415175 | Yes | NA | Yes | Apistan | Yes | Fondant or sugar candy, Pollen substitute | none |
| 1660 | 2021 | 3.415175 | Yes | NA | Yes | Apistan | Yes | Fondant or sugar candy, Pollen substitute | none |
| 1661 | 2021 | 3.415175 | Yes | NA | Yes | Apistan | Yes | Fondant or sugar candy, Pollen substitute | none |
| 1662 | 2021 | 3.415175 | Yes | NA | Yes | Apistan | Yes | Fondant or sugar candy, Pollen substitute | none |
| 1663 | 2021 | 3.415175 | Yes | NA | Yes | Apistan | Yes | Fondant or sugar candy, Pollen substitute | none |
| 1664 | 2021 | 3.415175 | Yes | NA | Yes | Apistan | Yes | Fondant or sugar candy, Pollen substitute | none |
| 1665 | 2021 | 2.733647 | Yes | Sugar roll | Yes | Oxalic Acid (Vapor) | Yes | Fondant or sugar candy, Dry sugar, Pollen substitute | none |
| 1666 | 2021 | 2.733647 | Yes | Sugar roll | Yes | Oxalic Acid (Vapor) | Yes | Fondant or sugar candy, Dry sugar, Pollen substitute | none |
| 1667 | 2021 | 2.733647 | Yes | Sugar roll | Yes | Oxalic Acid (Vapor) | Yes | Fondant or sugar candy, Dry sugar, Pollen substitute | none |
| 1668 | 2021 | 2.733647 | Yes | Sugar roll | Yes | Oxalic Acid (Vapor) | Yes | Fondant or sugar candy, Dry sugar, Pollen substitute | none |
| 1669 | 2021 | 2.733647 | Yes | Sugar roll | Yes | Oxalic Acid (Vapor) | Yes | Fondant or sugar candy, Dry sugar, Pollen substitute | none |
| 1670 | 2021 | 16.14957 | Yes | Sugar roll | Yes | Formic Acid (Mite Away Quick Strips) | Yes | Fondant or sugar candy | sugar only |
| 1671 | 2021 | 16.14957 | Yes | Sugar roll | Yes | Formic Acid (Mite Away Quick Strips) | Yes | Fondant or sugar candy | sugar only |
| 1672 | 2021 | 13.80022 | Yes | Alcohol wash | Yes | Apivar (Amitraz) | Yes | Fondant or sugar candy, Pollen substitute | none |
| 1673 | 2021 | 13.80022 | Yes | Alcohol wash | Yes | Apivar (Amitraz) | Yes | Fondant or sugar candy, Pollen substitute | none |
| 1674 | 2021 | 13.80022 | Yes | Alcohol wash | Yes | Apivar (Amitraz) | Yes | Fondant or sugar candy, Pollen substitute | none |
| 1675 | 2021 | 7.26817 | Yes | Drone brood inspection | Yes | Oxalic Acid (Vapor) | Yes | Honey from your own stock, Commercially available supplements | none |
| 1676 | 2021 | 7.26817 | Yes | Drone brood inspection | Yes | Oxalic Acid (Vapor) | Yes | Honey from your own stock, Commercially available supplements | none |
| 1677 | 2021 | 7.26817 | Yes | Drone brood inspection | Yes | Oxalic Acid (Vapor) | Yes | Honey from your own stock, Commercially available supplements | none |
| 1678 | 2021 | 7.26817 | Yes | Drone brood inspection | Yes | Oxalic Acid (Vapor) | Yes | Honey from your own stock, Commercially available supplements | none |
| 1679 | 2021 | 7.26817 | Yes | Drone brood inspection | Yes | Oxalic Acid (Vapor) | Yes | Honey from your own stock, Commercially available supplements | none |
| 1680 | 2021 | 7.26817 | Yes | Drone brood inspection | Yes | Oxalic Acid (Vapor) | Yes | Honey from your own stock, Commercially available supplements | none |
| 1681 | 2021 | 7.26817 | Yes | Drone brood inspection | Yes | Oxalic Acid (Vapor) | Yes | Honey from your own stock, Commercially available supplements | none |
| 1682 | 2021 | 7.26817 | Yes | Drone brood inspection | Yes | Oxalic Acid (Vapor) | Yes | Honey from your own stock, Commercially available supplements | none |
| 1683 | 2021 | 7.26817 | Yes | Drone brood inspection | Yes | Oxalic Acid (Vapor) | Yes | Honey from your own stock, Commercially available supplements | none |
| 1684 | 2021 | 7.26817 | Yes | Drone brood inspection | Yes | Oxalic Acid (Vapor) | Yes | Honey from your own stock, Commercially available supplements | none |
| 1685 | 2021 | 7.26817 | Yes | Drone brood inspection | Yes | Oxalic Acid (Vapor) | Yes | Honey from your own stock, Commercially available supplements | none |
| 1686 | 2021 | 7.26817 | Yes | Drone brood inspection | Yes | Oxalic Acid (Vapor) | Yes | Honey from your own stock, Commercially available supplements | none |
| 1687 | 2021 | 7.26817 | Yes | Drone brood inspection | Yes | Oxalic Acid (Vapor) | Yes | Honey from your own stock, Commercially available supplements | none |
| 1688 | 2021 | 7.26817 | Yes | Drone brood inspection | Yes | Oxalic Acid (Vapor) | Yes | Honey from your own stock, Commercially available supplements | none |
| 1689 | 2021 | 7.26817 | Yes | Drone brood inspection | Yes | Oxalic Acid (Vapor) | Yes | Honey from your own stock, Commercially available supplements | none |
| 1690 | 2021 | 7.26817 | Yes | Drone brood inspection | Yes | Oxalic Acid (Vapor) | Yes | Honey from your own stock, Commercially available supplements | none |
| 1691 | 2021 | 7.26817 | Yes | Drone brood inspection | Yes | Oxalic Acid (Vapor) | Yes | Honey from your own stock, Commercially available supplements | none |
| 1692 | 2021 | 7.26817 | Yes | Drone brood inspection | Yes | Oxalic Acid (Vapor) | Yes | Honey from your own stock, Commercially available supplements | none |
| 1693 | 2021 | 7.26817 | Yes | Drone brood inspection | Yes | Oxalic Acid (Vapor) | Yes | Honey from your own stock, Commercially available supplements | none |
| 1694 | 2021 | 7.26817 | Yes | Drone brood inspection | Yes | Oxalic Acid (Vapor) | Yes | Honey from your own stock, Commercially available supplements | none |
| 1695 | 2021 | 1.499818 | Yes | 48 hr drop (sticky board) | Yes | Oxalic Acid (Vapor) | Yes | Fondant or sugar candy | sugar only |
| 1696 | 2021 | 13.57898 | Yes | Sugar roll | Yes | Apivar (Amitraz) | Yes | Fondant or sugar candy, Pollen substitute, Commercially available supplements | none |
| 1697 | 2021 | 0.397097 | Yes | Alcohol wash | Yes | Formic Acid (Mite Away Quick Strips) | Yes | Dry sugar, Pollen substitute | none |
| 1698 | 2021 | 0.397097 | Yes | Alcohol wash | Yes | Formic Acid (Mite Away Quick Strips) | Yes | Dry sugar, Pollen substitute | none |
| 1699 | 2021 | 0.397097 | Yes | Alcohol wash | Yes | Formic Acid (Mite Away Quick Strips) | Yes | Dry sugar, Pollen substitute | none |
| 1700 | 2021 | 0.397097 | Yes | Alcohol wash | Yes | Formic Acid (Mite Away Quick Strips) | Yes | Dry sugar, Pollen substitute | none |
| 1701 | 2021 | 0.397097 | Yes | Alcohol wash | Yes | Formic Acid (Mite Away Quick Strips) | Yes | Dry sugar, Pollen substitute | none |
| 1702 | 2021 | 0.397097 | Yes | Alcohol wash | Yes | Formic Acid (Mite Away Quick Strips) | Yes | Dry sugar, Pollen substitute | none |
| 1703 | 2021 | 0.397097 | Yes | Alcohol wash | Yes | Formic Acid (Mite Away Quick Strips) | Yes | Dry sugar, Pollen substitute | none |
| 1704 | 2021 | 0.397097 | Yes | Alcohol wash | Yes | Formic Acid (Mite Away Quick Strips) | Yes | Dry sugar, Pollen substitute | none |
| 1705 | 2021 | 0.397097 | Yes | Alcohol wash | Yes | Formic Acid (Mite Away Quick Strips) | Yes | Dry sugar, Pollen substitute | none |
| 1706 | 2021 | 0.397097 | Yes | Alcohol wash | Yes | Formic Acid (Mite Away Quick Strips) | Yes | Dry sugar, Pollen substitute | none |
| 1707 | 2021 | 0.397097 | Yes | Alcohol wash | Yes | Formic Acid (Mite Away Quick Strips) | Yes | Dry sugar, Pollen substitute | none |
| 1708 | 2021 | 0.397097 | Yes | Alcohol wash | Yes | Formic Acid (Mite Away Quick Strips) | Yes | Dry sugar, Pollen substitute | none |
| 1709 | 2021 | 0.397097 | Yes | Alcohol wash | Yes | Formic Acid (Mite Away Quick Strips) | Yes | Dry sugar, Pollen substitute | none |
| 1710 | 2021 | 0.397097 | Yes | Alcohol wash | Yes | Formic Acid (Mite Away Quick Strips) | Yes | Dry sugar, Pollen substitute | none |
| 1711 | 2021 | 0.397097 | Yes | Alcohol wash | Yes | Formic Acid (Mite Away Quick Strips) | Yes | Dry sugar, Pollen substitute | none |
| 1712 | 2021 | 0.397097 | Yes | Alcohol wash | Yes | Formic Acid (Mite Away Quick Strips) | Yes | Dry sugar, Pollen substitute | none |
| 1713 | 2021 | 1.303747 | No | NA | Yes | Apivar (Amitraz) | Yes | Fondant or sugar candy, Pollen substitute, Honey from your own stock | none |
| 1714 | 2021 | 1.303747 | No | NA | Yes | Apivar (Amitraz) | Yes | Fondant or sugar candy, Pollen substitute, Honey from your own stock | none |
| 1715 | 2021 | 1.303747 | No | NA | Yes | Apivar (Amitraz) | Yes | Fondant or sugar candy, Pollen substitute, Honey from your own stock | none |
| 1716 | 2021 | 1.389696 | Yes | Alcohol wash | Yes | Oxalic Acid (Dribble) | Yes | Fondant or sugar candy | sugar only |
| 1717 | 2021 | 1.389696 | Yes | Alcohol wash | Yes | Oxalic Acid (Dribble) | Yes | Fondant or sugar candy | sugar only |
| 1718 | 2021 | 1.389696 | Yes | Alcohol wash | Yes | Oxalic Acid (Dribble) | Yes | Fondant or sugar candy | sugar only |
| 1719 | 2021 | 1.389696 | Yes | Alcohol wash | Yes | Oxalic Acid (Dribble) | Yes | Fondant or sugar candy | sugar only |
| 1720 | 2021 | 1.389696 | Yes | Alcohol wash | Yes | Oxalic Acid (Dribble) | Yes | Fondant or sugar candy | sugar only |
| 1721 | 2021 | 8.04936 | No | NA | Yes | Formic Acid (Mite Away Quick Strips) | Yes | Dry sugar | sugar only |
| 1722 | 2021 | 8.04936 | No | NA | Yes | Formic Acid (Mite Away Quick Strips) | Yes | Dry sugar | sugar only |
| 1723 | 2021 | 2.928518 | Yes | Sugar roll | Yes | Oxalic Acid (Vapor) | Yes | Dry sugar, Pollen substitute | none |
| 1724 | 2021 | 2.928518 | Yes | Sugar roll | Yes | Oxalic Acid (Vapor) | Yes | Dry sugar, Pollen substitute | none |
| 1725 | 2021 | 16.32379 | Yes | Sugar roll | Yes | Formic Acid (Mite Away Quick Strips) | Yes | Fondant or sugar candy, Pollen substitute | none |
| 1726 | 2021 | 10.85041 | Yes | Alcohol wash | Yes | Formic Acid (Mite Away Quick Strips) | Yes | Commercially available supplements | none |
| 1727 | 2021 | 10.85041 | Yes | Alcohol wash | Yes | Formic Acid (Mite Away Quick Strips) | Yes | Commercially available supplements | none |
| 1728 | 2021 | 10.85041 | Yes | Alcohol wash | Yes | Formic Acid (Mite Away Quick Strips) | Yes | Commercially available supplements | none |
| 1729 | 2021 | 10.85041 | Yes | Alcohol wash | Yes | Formic Acid (Mite Away Quick Strips) | Yes | Commercially available supplements | none |
| 1730 | 2021 | 16.96768 | Yes | Alcohol wash | Yes | Formic Acid (Mite Away Quick Strips) | Yes | Fondant or sugar candy | sugar only |
| 1731 | 2021 | 16.96768 | Yes | Alcohol wash | Yes | Formic Acid (Mite Away Quick Strips) | Yes | Fondant or sugar candy | sugar only |
| 1732 | 2021 | 16.96768 | Yes | Alcohol wash | Yes | Formic Acid (Mite Away Quick Strips) | Yes | Fondant or sugar candy | sugar only |
| 1733 | 2021 | 16.96768 | Yes | Alcohol wash | Yes | Formic Acid (Mite Away Quick Strips) | Yes | Fondant or sugar candy | sugar only |
| 1734 | 2021 | 2.832229 | Yes | Alcohol wash | Yes | Formic Acid (Mite Away Quick Strips) | Yes | Dry sugar | sugar only |
| 1735 | 2021 | 2.832229 | Yes | Alcohol wash | Yes | Formic Acid (Mite Away Quick Strips) | Yes | Dry sugar | sugar only |
| 1736 | 2021 | 2.832229 | Yes | Alcohol wash | Yes | Formic Acid (Mite Away Quick Strips) | Yes | Dry sugar | sugar only |
| 1737 | 2021 | 6.187106 | Yes | Sugar roll | Yes | Oxalic Acid (Dribble) | Yes | Dry sugar | sugar only |
| 1738 | 2021 | 5.090035 | Yes | Alcohol wash | Yes | Hopguard | Yes | Fondant or sugar candy | sugar only |
| 1739 | 2021 | 5.090035 | Yes | Alcohol wash | Yes | Hopguard | Yes | Fondant or sugar candy | sugar only |
| 1740 | 2021 | 5.090035 | Yes | Alcohol wash | Yes | Hopguard | Yes | Fondant or sugar candy | sugar only |
| 1741 | 2021 | 5.090035 | Yes | Alcohol wash | Yes | Hopguard | Yes | Fondant or sugar candy | sugar only |
| 1742 | 2021 | 5.090035 | Yes | Alcohol wash | Yes | Hopguard | Yes | Fondant or sugar candy | sugar only |
| 1743 | 2021 | 5.090035 | Yes | Alcohol wash | Yes | Hopguard | Yes | Fondant or sugar candy | sugar only |
| 1744 | 2021 | 5.090035 | Yes | Alcohol wash | Yes | Hopguard | Yes | Fondant or sugar candy | sugar only |
| 1745 | 2021 | 1.599429 | Yes | Alcohol wash | Yes | Formic Acid (Mite Away Quick Strips) | Yes | Fondant or sugar candy | sugar only |
| 1746 | 2021 | 1.599429 | Yes | Alcohol wash | Yes | Formic Acid (Mite Away Quick Strips) | Yes | Fondant or sugar candy | sugar only |
| 1747 | 2021 | 1.599429 | Yes | Alcohol wash | Yes | Formic Acid (Mite Away Quick Strips) | Yes | Fondant or sugar candy | sugar only |
| 1748 | 2021 | 0.917341 | Yes | Alcohol wash | Yes | Oxalic Acid (Vapor) | Yes | Pollen substitute | pollen only |
| 1749 | 2021 | 0.917341 | Yes | Alcohol wash | Yes | Oxalic Acid (Vapor) | Yes | Pollen substitute | pollen only |
| 1750 | 2021 | 0.917341 | Yes | Alcohol wash | Yes | Oxalic Acid (Vapor) | Yes | Pollen substitute | pollen only |
| 1751 | 2021 | 0.917341 | Yes | Alcohol wash | Yes | Oxalic Acid (Vapor) | Yes | Pollen substitute | pollen only |
| 1752 | 2021 | 2.096637 | Yes | 48 hr drop (sticky board) | Yes | Oxalic Acid (Vapor) | Yes | Fondant or sugar candy, Pollen substitute | none |
| 1753 | 2021 | 2.096637 | Yes | 48 hr drop (sticky board) | Yes | Oxalic Acid (Vapor) | Yes | Fondant or sugar candy, Pollen substitute | none |
| 1754 | 2021 | 2.096637 | Yes | 48 hr drop (sticky board) | Yes | Oxalic Acid (Vapor) | Yes | Fondant or sugar candy, Pollen substitute | none |
| 1755 | 2021 | 2.096637 | Yes | 48 hr drop (sticky board) | Yes | Oxalic Acid (Vapor) | Yes | Fondant or sugar candy, Pollen substitute | none |
| 1756 | 2021 | 2.096637 | Yes | 48 hr drop (sticky board) | Yes | Oxalic Acid (Vapor) | Yes | Fondant or sugar candy, Pollen substitute | none |
| 1757 | 2021 | 2.096637 | Yes | 48 hr drop (sticky board) | Yes | Oxalic Acid (Vapor) | Yes | Fondant or sugar candy, Pollen substitute | none |
| 1758 | 2021 | 0.899815 | Yes | 48 hr drop (sticky board) | Yes | Apivar (Amitraz) | Yes | Fondant or sugar candy | sugar only |
| 1759 | 2021 | 0.899815 | Yes | 48 hr drop (sticky board) | Yes | Apivar (Amitraz) | Yes | Fondant or sugar candy | sugar only |
| 1760 | 2021 | 48.72584 | Yes | Sugar roll | Yes | Formic Acid (Mite Away Quick Strips) | Yes | Fondant or sugar candy | sugar only |
| 1761 | 2021 | 48.72584 | Yes | Sugar roll | Yes | Formic Acid (Mite Away Quick Strips) | Yes | Fondant or sugar candy | sugar only |
| 1762 | 2021 | 48.72584 | Yes | Sugar roll | Yes | Formic Acid (Mite Away Quick Strips) | Yes | Fondant or sugar candy | sugar only |
| 1763 | 2021 | 20.19943 | Yes | 48 hr drop (sticky board) | Yes | Formic Acid (Mite Away Quick Strips) | Yes | Fondant or sugar candy | sugar only |
| 1764 | 2021 | 20.19943 | Yes | 48 hr drop (sticky board) | Yes | Formic Acid (Mite Away Quick Strips) | Yes | Fondant or sugar candy | sugar only |
| 1765 | 2021 | 20.19943 | Yes | 48 hr drop (sticky board) | Yes | Formic Acid (Mite Away Quick Strips) | Yes | Fondant or sugar candy | sugar only |
| 1766 | 2021 | 9.837651 | Yes | Sugar roll | Yes | Formic Acid (Mite Away Quick Strips) | Yes | Dry sugar, Honey from your own stock | none |
| 1767 | 2021 | 9.837651 | Yes | Sugar roll | Yes | Formic Acid (Mite Away Quick Strips) | Yes | Dry sugar, Honey from your own stock | none |
| 1768 | 2021 | 15.79886 | Yes | Sugar roll | Yes | Formic Acid (Mite Away Quick Strips) | Yes | Fondant or sugar candy | sugar only |
| 1769 | 2021 | 11.13922 | Yes | Drone brood inspection | Yes | Oxalic Acid (Vapor) | Yes | Fondant or sugar candy | sugar only |
| 1770 | 2021 | 11.13922 | Yes | Drone brood inspection | Yes | Oxalic Acid (Vapor) | Yes | Fondant or sugar candy | sugar only |
| 1771 | 2021 | 11.13922 | Yes | Drone brood inspection | Yes | Oxalic Acid (Vapor) | Yes | Fondant or sugar candy | sugar only |
| 1772 | 2021 | 11.13922 | Yes | Drone brood inspection | Yes | Oxalic Acid (Vapor) | Yes | Fondant or sugar candy | sugar only |
| 1773 | 2021 | 11.13922 | Yes | Drone brood inspection | Yes | Oxalic Acid (Vapor) | Yes | Fondant or sugar candy | sugar only |
| 1774 | 2021 | 11.13922 | Yes | Drone brood inspection | Yes | Oxalic Acid (Vapor) | Yes | Fondant or sugar candy | sugar only |
| 1775 | 2021 | 11.13922 | Yes | Drone brood inspection | Yes | Oxalic Acid (Vapor) | Yes | Fondant or sugar candy | sugar only |
| 1776 | 2021 | 11.13922 | Yes | Drone brood inspection | Yes | Oxalic Acid (Vapor) | Yes | Fondant or sugar candy | sugar only |
| 1777 | 2021 | 5.728103 | Yes | 48 hr drop (sticky board) | Yes | Formic Acid (Mite Away Quick Strips) | Yes | Fondant or sugar candy | sugar only |
| 1778 | 2021 | 5.728103 | Yes | 48 hr drop (sticky board) | Yes | Formic Acid (Mite Away Quick Strips) | Yes | Fondant or sugar candy | sugar only |
| 1779 | 2021 | 5.728103 | Yes | 48 hr drop (sticky board) | Yes | Formic Acid (Mite Away Quick Strips) | Yes | Fondant or sugar candy | sugar only |
| 1780 | 2021 | 5.728103 | Yes | 48 hr drop (sticky board) | Yes | Formic Acid (Mite Away Quick Strips) | Yes | Fondant or sugar candy | sugar only |
| 1781 | 2021 | 5.728103 | Yes | 48 hr drop (sticky board) | Yes | Formic Acid (Mite Away Quick Strips) | Yes | Fondant or sugar candy | sugar only |
| 1782 | 2021 | 5.728103 | Yes | 48 hr drop (sticky board) | Yes | Formic Acid (Mite Away Quick Strips) | Yes | Fondant or sugar candy | sugar only |
| 1783 | 2021 | 1.247116 | Yes | Sugar roll | Yes | Oxalic Acid (Vapor) | Yes | Fondant or sugar candy | sugar only |
| 1784 | 2021 | 1.247116 | Yes | Sugar roll | Yes | Oxalic Acid (Vapor) | Yes | Fondant or sugar candy | sugar only |
| 1785 | 2021 | 5.530174 | No | NA | Yes | Api life Var | Yes | Dry sugar | sugar only |
| 1786 | 2021 | 5.530174 | No | NA | Yes | Api life Var | Yes | Dry sugar | sugar only |
| 1787 | 2021 | 5.530174 | No | NA | Yes | Api life Var | Yes | Dry sugar | sugar only |
| 1788 | 2021 | 5.530174 | No | NA | Yes | Api life Var | Yes | Dry sugar | sugar only |
| 1789 | 2021 | 5.530174 | No | NA | Yes | Api life Var | Yes | Dry sugar | sugar only |
| 1790 | 2021 | 5.530174 | No | NA | Yes | Api life Var | Yes | Dry sugar | sugar only |
| 1791 | 2021 | 1.501978 | Yes | Drone brood inspection | Yes | Hopguard | Yes | Commercially available supplements | none |
| 1792 | 2021 | 1.501978 | Yes | Drone brood inspection | Yes | Hopguard | Yes | Commercially available supplements | none |
| 1793 | 2021 | 1.501978 | Yes | Drone brood inspection | Yes | Hopguard | Yes | Commercially available supplements | none |
| 1794 | 2021 | 18.88673 | Yes | Alcohol wash | Yes | Formic Acid (Mite Away Quick Strips) | Yes | Other | none |
| 1795 | 2021 | 18.88673 | Yes | Alcohol wash | Yes | Formic Acid (Mite Away Quick Strips) | Yes | Other | none |
| 1796 | 2021 | 6.302087 | Yes | Sugar roll | Yes | Formic Acid (Mite Away Quick Strips) | Yes | Sugar syrup, Dry sugar | none |
| 1797 | 2021 | 6.302087 | Yes | Sugar roll | Yes | Formic Acid (Mite Away Quick Strips) | Yes | Sugar syrup, Dry sugar | none |
| 1798 | 2021 | 6.302087 | Yes | Sugar roll | Yes | Formic Acid (Mite Away Quick Strips) | Yes | Sugar syrup, Dry sugar | none |
| 1799 | 2021 | 5.491681 | Yes | Alcohol wash | Yes | Formic Acid (Mite Away Quick Strips) | Yes | Fondant or sugar candy, Dry sugar, Pollen substitute | none |
| 1800 | 2021 | 5.491681 | Yes | Alcohol wash | Yes | Formic Acid (Mite Away Quick Strips) | Yes | Fondant or sugar candy, Dry sugar, Pollen substitute | none |
| 1801 | 2021 | 5.491681 | Yes | Alcohol wash | Yes | Formic Acid (Mite Away Quick Strips) | Yes | Fondant or sugar candy, Dry sugar, Pollen substitute | none |
| 1802 | 2021 | 5.491681 | Yes | Alcohol wash | Yes | Formic Acid (Mite Away Quick Strips) | Yes | Fondant or sugar candy, Dry sugar, Pollen substitute | none |
| 1803 | 2021 | 0.52663 | Yes | Alcohol wash | Yes | Formic Acid (Mite Away Quick Strips) | Yes | Fondant or sugar candy | sugar only |
| 1804 | 2021 | 0.52663 | Yes | Alcohol wash | Yes | Formic Acid (Mite Away Quick Strips) | Yes | Fondant or sugar candy | sugar only |
| 1805 | 2021 | 10.35496 | Yes | Alcohol wash | Yes | Formic Acid (Mite Away Quick Strips) | Yes | Pollen substitute | pollen only |
| 1806 | 2021 | 10.35496 | Yes | Alcohol wash | Yes | Formic Acid (Mite Away Quick Strips) | Yes | Pollen substitute | pollen only |
| 1807 | 2021 | 10.35496 | Yes | Alcohol wash | Yes | Formic Acid (Mite Away Quick Strips) | Yes | Pollen substitute | pollen only |
| 1808 | 2021 | 10.35496 | Yes | Alcohol wash | Yes | Formic Acid (Mite Away Quick Strips) | Yes | Pollen substitute | pollen only |
| 1809 | 2021 | 2.423268 | Yes | Drone brood inspection | Yes | Oxalic Acid (Vapor) | Yes | Fondant or sugar candy, Pollen substitute | none |
| 1810 | 2021 | 2.423268 | Yes | Drone brood inspection | Yes | Oxalic Acid (Vapor) | Yes | Fondant or sugar candy, Pollen substitute | none |
| 1811 | 2021 | 2.423268 | Yes | Drone brood inspection | Yes | Oxalic Acid (Vapor) | Yes | Fondant or sugar candy, Pollen substitute | none |
| 1812 | 2021 | 2.423268 | Yes | Drone brood inspection | Yes | Oxalic Acid (Vapor) | Yes | Fondant or sugar candy, Pollen substitute | none |
| 1813 | 2021 | 2.423268 | Yes | Drone brood inspection | Yes | Oxalic Acid (Vapor) | Yes | Fondant or sugar candy, Pollen substitute | none |
| 1814 | 2021 | 2.423268 | Yes | Drone brood inspection | Yes | Oxalic Acid (Vapor) | Yes | Fondant or sugar candy, Pollen substitute | none |
| 1815 | 2021 | 2.423268 | Yes | Drone brood inspection | Yes | Oxalic Acid (Vapor) | Yes | Fondant or sugar candy, Pollen substitute | none |
| 1816 | 2021 | 2.423268 | Yes | Drone brood inspection | Yes | Oxalic Acid (Vapor) | Yes | Fondant or sugar candy, Pollen substitute | none |
| 1817 | 2021 | 30.42022 | Yes | Other | Yes | Apivar (Amitraz) | Yes | Fondant or sugar candy, Commercially available supplements | none |
| 1818 | 2021 | 30.42022 | Yes | Other | Yes | Apivar (Amitraz) | Yes | Fondant or sugar candy, Commercially available supplements | none |
| 1819 | 2021 | 30.42022 | Yes | Other | Yes | Apivar (Amitraz) | Yes | Fondant or sugar candy, Commercially available supplements | none |
| 1820 | 2021 | 30.42022 | Yes | Other | Yes | Apivar (Amitraz) | Yes | Fondant or sugar candy, Commercially available supplements | none |
| 1821 | 2021 | 30.42022 | Yes | Other | Yes | Apivar (Amitraz) | Yes | Fondant or sugar candy, Commercially available supplements | none |
| 1822 | 2021 | 13.55301 | Yes | Alcohol wash | Yes | Formic Acid (Mite Away Quick Strips) | Yes | Fondant or sugar candy | sugar only |
| 1823 | 2021 | 19.62828 | Yes | Alcohol wash | Yes | Formic Acid (Mite Away Quick Strips) | Yes | Fondant or sugar candy, Sugar syrup | none |
| 1824 | 2021 | 18.60895 | Yes | Sugar roll | Yes | Formic Acid (Mite Away Quick Strips) | Yes | Fondant or sugar candy | sugar only |
| 1825 | 2021 | 18.60895 | Yes | Sugar roll | Yes | Formic Acid (Mite Away Quick Strips) | Yes | Fondant or sugar candy | sugar only |
| 1826 | 2021 | 5.041248 | Yes | Sugar roll | Yes | Formic Acid (Mite Away Quick Strips) | Yes | Dry sugar, Pollen substitute, Other | none |
| 1827 | 2021 | 20.38761 | No | NA | Yes | Formic Acid (Mite Away Quick Strips) | Yes | Honey from your own stock | none |
| 1828 | 2021 | 20.38761 | No | NA | Yes | Formic Acid (Mite Away Quick Strips) | Yes | Honey from your own stock | none |
| 1829 | 2021 | 20.38761 | No | NA | Yes | Formic Acid (Mite Away Quick Strips) | Yes | Honey from your own stock | none |
| 1830 | 2021 | 20.38761 | No | NA | Yes | Formic Acid (Mite Away Quick Strips) | Yes | Honey from your own stock | none |
| 1831 | 2021 | 2.673899 | Yes | Alcohol wash | Yes | Formic Acid (Mite Away Quick Strips) | Yes | Dry sugar | sugar only |
| 1832 | 2021 | 2.673899 | Yes | Alcohol wash | Yes | Formic Acid (Mite Away Quick Strips) | Yes | Dry sugar | sugar only |
| 1833 | 2021 | 2.673899 | Yes | Alcohol wash | Yes | Formic Acid (Mite Away Quick Strips) | Yes | Dry sugar | sugar only |
| 1834 | 2021 | 2.673899 | Yes | Alcohol wash | Yes | Formic Acid (Mite Away Quick Strips) | Yes | Dry sugar | sugar only |
| 1835 | 2021 | 2.146609 | Yes | Drone brood inspection, Other | Yes | Oxalic Acid (Vapor) | Yes | Dry sugar, Pollen substitute | none |
| 1836 | 2021 | 2.146609 | Yes | Drone brood inspection, Other | Yes | Oxalic Acid (Vapor) | Yes | Dry sugar, Pollen substitute | none |
| 1837 | 2021 | 2.146609 | Yes | Drone brood inspection, Other | Yes | Oxalic Acid (Vapor) | Yes | Dry sugar, Pollen substitute | none |
| 1838 | 2021 | 2.146609 | Yes | Drone brood inspection, Other | Yes | Oxalic Acid (Vapor) | Yes | Dry sugar, Pollen substitute | none |
| 1839 | 2021 | 2.146609 | Yes | Drone brood inspection, Other | Yes | Oxalic Acid (Vapor) | Yes | Dry sugar, Pollen substitute | none |
| 1840 | 2021 | 2.146609 | Yes | Drone brood inspection, Other | Yes | Oxalic Acid (Vapor) | Yes | Dry sugar, Pollen substitute | none |
| 1841 | 2021 | 2.146609 | Yes | Drone brood inspection, Other | Yes | Oxalic Acid (Vapor) | Yes | Dry sugar, Pollen substitute | none |
| 1842 | 2021 | 0.18404 | Yes | Sugar roll, Alcohol wash | Yes | Formic Acid (Mite Away Quick Strips) | Yes | Fondant or sugar candy, Dry sugar | none |
| 1843 | 2021 | 0.18404 | Yes | Sugar roll, Alcohol wash | Yes | Formic Acid (Mite Away Quick Strips) | Yes | Fondant or sugar candy, Dry sugar | none |
| 1844 | 2021 | 0.18404 | Yes | Sugar roll, Alcohol wash | Yes | Formic Acid (Mite Away Quick Strips) | Yes | Fondant or sugar candy, Dry sugar | none |
| 1845 | 2021 | 9.987988 | Yes | 48 hr drop (sticky board), Sugar roll, Alcohol wash, Drone brood inspection | Yes | Formic Acid (Mite Away Quick Strips) | Yes | Fondant or sugar candy, Honey from your own stock | none |
| 1846 | 2021 | 9.987988 | Yes | 48 hr drop (sticky board), Sugar roll, Alcohol wash, Drone brood inspection | Yes | Formic Acid (Mite Away Quick Strips) | Yes | Fondant or sugar candy, Honey from your own stock | none |
| 1847 | 2021 | 9.987988 | Yes | 48 hr drop (sticky board), Sugar roll, Alcohol wash, Drone brood inspection | Yes | Formic Acid (Mite Away Quick Strips) | Yes | Fondant or sugar candy, Honey from your own stock | none |
| 1848 | 2021 | 9.987988 | Yes | 48 hr drop (sticky board), Sugar roll, Alcohol wash, Drone brood inspection | Yes | Formic Acid (Mite Away Quick Strips) | Yes | Fondant or sugar candy, Honey from your own stock | none |
| 1849 | 2021 | 9.987988 | Yes | 48 hr drop (sticky board), Sugar roll, Alcohol wash, Drone brood inspection | Yes | Formic Acid (Mite Away Quick Strips) | Yes | Fondant or sugar candy, Honey from your own stock | none |
| 1850 | 2021 | 9.987988 | Yes | 48 hr drop (sticky board), Sugar roll, Alcohol wash, Drone brood inspection | Yes | Formic Acid (Mite Away Quick Strips) | Yes | Fondant or sugar candy, Honey from your own stock | none |
| 1851 | 2021 | 9.987988 | Yes | 48 hr drop (sticky board), Sugar roll, Alcohol wash, Drone brood inspection | Yes | Formic Acid (Mite Away Quick Strips) | Yes | Fondant or sugar candy, Honey from your own stock | none |
| 1852 | 2021 | 9.987988 | Yes | 48 hr drop (sticky board), Sugar roll, Alcohol wash, Drone brood inspection | Yes | Formic Acid (Mite Away Quick Strips) | Yes | Fondant or sugar candy, Honey from your own stock | none |
| 1853 | 2021 | 9.987988 | Yes | 48 hr drop (sticky board), Sugar roll, Alcohol wash, Drone brood inspection | Yes | Formic Acid (Mite Away Quick Strips) | Yes | Fondant or sugar candy, Honey from your own stock | none |
| 1854 | 2021 | 9.987988 | Yes | 48 hr drop (sticky board), Sugar roll, Alcohol wash, Drone brood inspection | Yes | Formic Acid (Mite Away Quick Strips) | Yes | Fondant or sugar candy, Honey from your own stock | none |
| 1855 | 2021 | 9.987988 | Yes | 48 hr drop (sticky board), Sugar roll, Alcohol wash, Drone brood inspection | Yes | Formic Acid (Mite Away Quick Strips) | Yes | Fondant or sugar candy, Honey from your own stock | none |
| 1856 | 2021 | 9.987988 | Yes | 48 hr drop (sticky board), Sugar roll, Alcohol wash, Drone brood inspection | Yes | Formic Acid (Mite Away Quick Strips) | Yes | Fondant or sugar candy, Honey from your own stock | none |
| 1857 | 2021 | 0.936378 | Yes | Alcohol wash, Drone brood inspection | Yes | Formic Acid (Mite Away Quick Strips) | Yes | Fondant or sugar candy | sugar only |
| 1858 | 2021 | 0.936378 | Yes | Alcohol wash, Drone brood inspection | Yes | Formic Acid (Mite Away Quick Strips) | Yes | Fondant or sugar candy | sugar only |
| 1859 | 2021 | 0.936378 | Yes | Alcohol wash, Drone brood inspection | Yes | Formic Acid (Mite Away Quick Strips) | Yes | Fondant or sugar candy | sugar only |
| 1860 | 2021 | 0.936378 | Yes | Alcohol wash, Drone brood inspection | Yes | Formic Acid (Mite Away Quick Strips) | Yes | Fondant or sugar candy | sugar only |
| 1861 | 2021 | 8.858594 | Yes | Alcohol wash | Yes | Formic Acid (Mite Away Quick Strips) | Yes | Dry sugar | sugar only |
| 1862 | 2021 | 8.858594 | Yes | Alcohol wash | Yes | Formic Acid (Mite Away Quick Strips) | Yes | Dry sugar | sugar only |
| 1863 | 2021 | 8.858594 | Yes | Alcohol wash | Yes | Formic Acid (Mite Away Quick Strips) | Yes | Dry sugar | sugar only |
| 1864 | 2021 | 10.46258 | Yes | Alcohol wash | Yes | Oxalic Acid (Vapor) | Yes | Fondant or sugar candy | sugar only |
| 1865 | 2021 | 10.46258 | Yes | Alcohol wash | Yes | Oxalic Acid (Vapor) | Yes | Fondant or sugar candy | sugar only |
| 1866 | 2021 | 10.46258 | Yes | Alcohol wash | Yes | Oxalic Acid (Vapor) | Yes | Fondant or sugar candy | sugar only |
| 1867 | 2021 | 16.20246 | Yes | Other | Yes | Oxalic Acid (Vapor) | Yes | Commercially available supplements | none |
| 1868 | 2021 | 3.527681 | Yes | Alcohol wash | Yes | Oxalic Acid (Vapor) | Yes | Fondant or sugar candy, Dry sugar, Commercially available supplements | none |
| 1869 | 2021 | 3.527681 | Yes | Alcohol wash | Yes | Oxalic Acid (Vapor) | Yes | Fondant or sugar candy, Dry sugar, Commercially available supplements | none |
| 1870 | 2021 | 3.527681 | Yes | Alcohol wash | Yes | Oxalic Acid (Vapor) | Yes | Fondant or sugar candy, Dry sugar, Commercially available supplements | none |
| 1871 | 2021 | 3.527681 | Yes | Alcohol wash | Yes | Oxalic Acid (Vapor) | Yes | Fondant or sugar candy, Dry sugar, Commercially available supplements | none |
| 1872 | 2021 | 3.527681 | Yes | Alcohol wash | Yes | Oxalic Acid (Vapor) | Yes | Fondant or sugar candy, Dry sugar, Commercially available supplements | none |
| 1873 | 2021 | 3.527681 | Yes | Alcohol wash | Yes | Oxalic Acid (Vapor) | Yes | Fondant or sugar candy, Dry sugar, Commercially available supplements | none |
| 1874 | 2021 | 3.527681 | Yes | Alcohol wash | Yes | Oxalic Acid (Vapor) | Yes | Fondant or sugar candy, Dry sugar, Commercially available supplements | none |
| 1875 | 2021 | 1.237874 | Yes | Sugar roll | Yes | Formic Acid (Mite Away Quick Strips) | Yes | Commercially available supplements | none |
| 1876 | 2021 | 5.205471 | Yes | Other | Yes | Formic Acid (Mite Away Quick Strips) | No | Other | none |
| 1877 | 2021 | 3.332135 | Yes | 48 hr drop (sticky board), Drone brood inspection | Yes | Oxalic Acid (Dribble) | Yes | Fondant or sugar candy | sugar only |
| 1878 | 2021 | 3.272752 | Yes | 48 hr drop (sticky board) | Yes | Formic Acid (Mite Away Quick Strips) | Yes | Fondant or sugar candy, Honey from your own stock | none |
| 1879 | 2021 | 3.272752 | Yes | 48 hr drop (sticky board) | Yes | Formic Acid (Mite Away Quick Strips) | Yes | Fondant or sugar candy, Honey from your own stock | none |
| 1880 | 2021 | 3.272752 | Yes | 48 hr drop (sticky board) | Yes | Formic Acid (Mite Away Quick Strips) | Yes | Fondant or sugar candy, Honey from your own stock | none |
| 1881 | 2021 | 21.9707 | Yes | 48 hr drop (sticky board) | Yes | Formic Acid (Mite Away Quick Strips) | Yes | Fondant or sugar candy, Dry sugar | none |
| 1882 | 2021 | 21.9707 | Yes | 48 hr drop (sticky board) | Yes | Formic Acid (Mite Away Quick Strips) | Yes | Fondant or sugar candy, Dry sugar | none |
| 1883 | 2021 | 21.9707 | Yes | 48 hr drop (sticky board) | Yes | Formic Acid (Mite Away Quick Strips) | Yes | Fondant or sugar candy, Dry sugar | none |
| 1884 | 2021 | 17.37468 | Yes | 48 hr drop (sticky board) | Yes | Apistan | Yes | Sugar syrup, Honey from your own stock | none |
| 1885 | 2021 | 17.37468 | Yes | 48 hr drop (sticky board) | Yes | Apistan | Yes | Sugar syrup, Honey from your own stock | none |
| 1886 | 2021 | 10.08317 | No | NA | Yes | Hopguard | Yes | Fondant or sugar candy | sugar only |
| 1887 | 2021 | 0.655299 | Yes | Alcohol wash | Yes | Oxalic Acid (Vapor) | Yes | Dry sugar, Honey from your own stock | none |
| 1888 | 2021 | 0.655299 | Yes | Alcohol wash | Yes | Oxalic Acid (Vapor) | Yes | Dry sugar, Honey from your own stock | none |
| 1889 | 2021 | 9.810871 | Yes | Other | Yes | Oxalic Acid (Vapor) | Yes | Fondant or sugar candy, Pollen substitute | none |
| 1890 | 2021 | 9.810871 | Yes | Other | Yes | Oxalic Acid (Vapor) | Yes | Fondant or sugar candy, Pollen substitute | none |
| 1891 | 2021 | 9.810871 | Yes | Other | Yes | Oxalic Acid (Vapor) | Yes | Fondant or sugar candy, Pollen substitute | none |
| 1892 | 2021 | 9.810871 | Yes | Other | Yes | Oxalic Acid (Vapor) | Yes | Fondant or sugar candy, Pollen substitute | none |
| 1893 | 2021 | 9.810871 | Yes | Other | Yes | Oxalic Acid (Vapor) | Yes | Fondant or sugar candy, Pollen substitute | none |
| 1894 | 2021 | 9.810871 | Yes | Other | Yes | Oxalic Acid (Vapor) | Yes | Fondant or sugar candy, Pollen substitute | none |
| 1895 | 2021 | 10.08317 | No | NA | Yes | Hopguard | Yes | Fondant or sugar candy | sugar only |
| 1896 | 2021 | 13.37408 | Yes | 48 hr drop (sticky board), Drone brood inspection | Yes | Oxalic Acid (Vapor) | Yes | Fondant or sugar candy | sugar only |
| 1897 | 2021 | 13.37408 | Yes | 48 hr drop (sticky board), Drone brood inspection | Yes | Oxalic Acid (Vapor) | Yes | Fondant or sugar candy | sugar only |
| 1898 | 2021 | 1.187874 | Yes | Sugar roll | Yes | Formic Acid (Mite Away Quick Strips) | Yes | Fondant or sugar candy | sugar only |
| 1899 | 2021 | 5.125858 | Yes | Drone brood inspection | Yes | Hopguard | Yes | Sugar syrup, Pollen substitute | none |
| 1900 | 2021 | 4.945792 | Yes | 48 hr drop (sticky board) | Yes | Formic Acid (Mite Away Quick Strips) | Yes | Dry sugar, Pollen substitute | none |
| 1901 | 2021 | 12.76694 | Yes | Alcohol wash | Yes | Formic Acid (Mite Away Quick Strips) | Yes | Fondant or sugar candy, Pollen substitute | none |
| 1902 | 2021 | 12.76694 | Yes | Alcohol wash | Yes | Formic Acid (Mite Away Quick Strips) | Yes | Fondant or sugar candy, Pollen substitute | none |
| 1903 | 2021 | 12.76694 | Yes | Alcohol wash | Yes | Formic Acid (Mite Away Quick Strips) | Yes | Fondant or sugar candy, Pollen substitute | none |
| 1904 | 2021 | 12.76694 | Yes | Alcohol wash | Yes | Formic Acid (Mite Away Quick Strips) | Yes | Fondant or sugar candy, Pollen substitute | none |
| 1905 | 2021 | 12.76694 | Yes | Alcohol wash | Yes | Formic Acid (Mite Away Quick Strips) | Yes | Fondant or sugar candy, Pollen substitute | none |
| 1906 | 2021 | 12.76694 | Yes | Alcohol wash | Yes | Formic Acid (Mite Away Quick Strips) | Yes | Fondant or sugar candy, Pollen substitute | none |
| 1907 | 2021 | 12.76694 | Yes | Alcohol wash | Yes | Formic Acid (Mite Away Quick Strips) | Yes | Fondant or sugar candy, Pollen substitute | none |
| 1908 | 2021 | 5.69195 | No | NA | Yes | Oxalic Acid (Dribble) | Yes | Sugar syrup, Dry sugar | none |
| 1909 | 2021 | 5.69195 | No | NA | Yes | Oxalic Acid (Dribble) | Yes | Sugar syrup, Dry sugar | none |
| 1910 | 2021 | 5.69195 | No | NA | Yes | Oxalic Acid (Dribble) | Yes | Sugar syrup, Dry sugar | none |
| 1911 | 2021 | 14.17384 | Yes | Sugar roll | Yes | Oxalic Acid (Dribble) | Yes | Fondant or sugar candy, Sugar syrup | none |
| 1912 | 2021 | 14.17384 | Yes | Sugar roll | Yes | Oxalic Acid (Dribble) | Yes | Fondant or sugar candy, Sugar syrup | none |
| 1913 | 2021 | 14.17384 | Yes | Sugar roll | Yes | Oxalic Acid (Dribble) | Yes | Fondant or sugar candy, Sugar syrup | none |
| 1914 | 2021 | 14.05617 | Yes | Sugar roll | Yes | Oxalic Acid (Dribble) | Yes | Dry sugar | sugar only |
| 1915 | 2021 | 14.05617 | Yes | Sugar roll | Yes | Oxalic Acid (Dribble) | Yes | Dry sugar | sugar only |
| 1916 | 2021 | 14.05617 | Yes | Sugar roll | Yes | Oxalic Acid (Dribble) | Yes | Dry sugar | sugar only |
| 1917 | 2021 | 14.05617 | Yes | Sugar roll | Yes | Oxalic Acid (Dribble) | Yes | Dry sugar | sugar only |
| 1918 | 2021 | 18.7242 | Yes | 48 hr drop (sticky board) | Yes | Formic Acid (Mite Away Quick Strips) | Yes | Fondant or sugar candy | sugar only |
| 1919 | 2021 | 18.7242 | Yes | 48 hr drop (sticky board) | Yes | Formic Acid (Mite Away Quick Strips) | Yes | Fondant or sugar candy | sugar only |
| 1920 | 2021 | 18.7242 | Yes | 48 hr drop (sticky board) | Yes | Formic Acid (Mite Away Quick Strips) | Yes | Fondant or sugar candy | sugar only |
| 1921 | 2021 | 10.67071 | Yes | Sugar roll | Yes | Formic Acid (Mite Away Quick Strips) | Yes | Fondant or sugar candy | sugar only |
| 1922 | 2021 | 27.16974 | No | NA | Yes | Formic Acid (Mite Away Quick Strips) | Yes | Fondant or sugar candy | sugar only |
| 1923 | 2021 | 27.16974 | No | NA | Yes | Formic Acid (Mite Away Quick Strips) | Yes | Fondant or sugar candy | sugar only |
| 1924 | 2021 | 27.16974 | No | NA | Yes | Formic Acid (Mite Away Quick Strips) | Yes | Fondant or sugar candy | sugar only |
| 1925 | 2021 | 27.16974 | No | NA | Yes | Formic Acid (Mite Away Quick Strips) | Yes | Fondant or sugar candy | sugar only |
| 1926 | 2021 | 3.769714 | Yes | 48 hr drop (sticky board), Drone brood inspection | Yes | Formic Acid (Mite Away Quick Strips) | Yes | Fondant or sugar candy, Pollen substitute, Honey from your own stock | none |
| 1927 | 2021 | 3.769714 | Yes | 48 hr drop (sticky board), Drone brood inspection | Yes | Formic Acid (Mite Away Quick Strips) | Yes | Fondant or sugar candy, Pollen substitute, Honey from your own stock | none |
| 1928 | 2021 | 3.769714 | Yes | 48 hr drop (sticky board), Drone brood inspection | Yes | Formic Acid (Mite Away Quick Strips) | Yes | Fondant or sugar candy, Pollen substitute, Honey from your own stock | none |
| 1929 | 2021 | 3.769714 | Yes | 48 hr drop (sticky board), Drone brood inspection | Yes | Formic Acid (Mite Away Quick Strips) | Yes | Fondant or sugar candy, Pollen substitute, Honey from your own stock | none |
| 1930 | 2021 | 3.769714 | Yes | 48 hr drop (sticky board), Drone brood inspection | Yes | Formic Acid (Mite Away Quick Strips) | Yes | Fondant or sugar candy, Pollen substitute, Honey from your own stock | none |
| 1931 | 2021 | 3.769714 | Yes | 48 hr drop (sticky board), Drone brood inspection | Yes | Formic Acid (Mite Away Quick Strips) | Yes | Fondant or sugar candy, Pollen substitute, Honey from your own stock | none |
| 1932 | 2021 | 2.004242 | Yes | 48 hr drop (sticky board) | Yes | Oxalic Acid (Vapor) | Yes | Fondant or sugar candy, Pollen from your own stock | none |
| 1933 | 2021 | 2.004242 | Yes | 48 hr drop (sticky board) | Yes | Oxalic Acid (Vapor) | Yes | Fondant or sugar candy, Pollen from your own stock | none |
| 1934 | 2021 | 2.004242 | Yes | 48 hr drop (sticky board) | Yes | Oxalic Acid (Vapor) | Yes | Fondant or sugar candy, Pollen from your own stock | none |
| 1935 | 2021 | 8.340979 | Yes | Alcohol wash | Yes | Formic Acid (Mite Away Quick Strips) | Yes | Fondant or sugar candy | sugar only |
| 1936 | 2021 | 34.31776 | No | NA | Yes | Formic Acid (Mite Away Quick Strips) | Yes | Honey from your own stock | none |
| 1937 | 2021 | 6.397644 | Yes | Sugar roll, Alcohol wash | Yes | Formic Acid (Mite Away Quick Strips) | Yes | Fondant or sugar candy, Dry sugar, Honey from your own stock | none |
| 1938 | 2021 | 6.397644 | Yes | Sugar roll, Alcohol wash | Yes | Formic Acid (Mite Away Quick Strips) | Yes | Fondant or sugar candy, Dry sugar, Honey from your own stock | none |
| 1939 | 2021 | 1.119337 | Yes | Sugar roll | Yes | Hopguard | Yes | Sugar syrup, Honey from your own stock | none |
| 1940 | 2021 | 1.119337 | Yes | Sugar roll | Yes | Hopguard | Yes | Sugar syrup, Honey from your own stock | none |
| 1941 | 2021 | 1.119337 | Yes | Sugar roll | Yes | Hopguard | Yes | Sugar syrup, Honey from your own stock | none |
| 1942 | 2021 | 1.119337 | Yes | Sugar roll | Yes | Hopguard | Yes | Sugar syrup, Honey from your own stock | none |
| 1943 | 2021 | 1.118436 | No | NA | Yes | Apivar (Amitraz) | Yes | Fondant or sugar candy | sugar only |
| 1944 | 2021 | 1.118436 | No | NA | Yes | Apivar (Amitraz) | Yes | Fondant or sugar candy | sugar only |
| 1945 | 2021 | 1.118436 | No | NA | Yes | Apivar (Amitraz) | Yes | Fondant or sugar candy | sugar only |
| 1946 | 2021 | 5.498045 | Yes | 48 hr drop (sticky board) | Yes | Oxalic Acid (Vapor) | Yes | Fondant or sugar candy | sugar only |
| 1947 | 2021 | 5.498045 | Yes | 48 hr drop (sticky board) | Yes | Oxalic Acid (Vapor) | Yes | Fondant or sugar candy | sugar only |
| 1948 | 2021 | 5.498045 | Yes | 48 hr drop (sticky board) | Yes | Oxalic Acid (Vapor) | Yes | Fondant or sugar candy | sugar only |
| 1949 | 2021 | 28.07233 | No | Other | Yes | Formic Acid (Mite Away Quick Strips) | Yes | Fondant or sugar candy, Commercially available supplements | none |
| 1950 | 2021 | 28.07233 | No | Other | Yes | Formic Acid (Mite Away Quick Strips) | Yes | Fondant or sugar candy, Commercially available supplements | none |
| 1951 | 2021 | 28.07233 | No | Other | Yes | Formic Acid (Mite Away Quick Strips) | Yes | Fondant or sugar candy, Commercially available supplements | none |
| 1952 | 2021 | 28.07233 | No | Other | Yes | Formic Acid (Mite Away Quick Strips) | Yes | Fondant or sugar candy, Commercially available supplements | none |
| 1953 | 2021 | 12.41755 | No | NA | Yes | Formic Acid (Mite Away Quick Strips) | Yes | Fondant or sugar candy, Honey from your own stock | none |
| 1954 | 2021 | 12.41755 | No | NA | Yes | Formic Acid (Mite Away Quick Strips) | Yes | Fondant or sugar candy, Honey from your own stock | none |
| 1955 | 2021 | 13.9802 | Yes | Alcohol wash | Yes | Formic Acid (Mite Away Quick Strips) | Yes | Dry sugar | sugar only |
| 1956 | 2021 | 13.9802 | Yes | Alcohol wash | Yes | Formic Acid (Mite Away Quick Strips) | Yes | Dry sugar | sugar only |
| 1957 | 2021 | 13.9802 | Yes | Alcohol wash | Yes | Formic Acid (Mite Away Quick Strips) | Yes | Dry sugar | sugar only |
| 1958 | 2021 | 13.9802 | Yes | Alcohol wash | Yes | Formic Acid (Mite Away Quick Strips) | Yes | Dry sugar | sugar only |
| 1959 | 2021 | 13.9802 | Yes | Alcohol wash | Yes | Formic Acid (Mite Away Quick Strips) | Yes | Dry sugar | sugar only |
| 1960 | 2021 | 18.5469 | Yes | Other | Yes | Oxalic Acid (Vapor) | Yes | Dry sugar, Commercially available supplements | none |
| 1961 | 2021 | 18.5469 | Yes | Other | Yes | Oxalic Acid (Vapor) | Yes | Dry sugar, Commercially available supplements | none |
| 1962 | 2021 | 12.66299 | Yes | 48 hr drop (sticky board), Sugar roll, Drone brood inspection | Yes | Formic Acid (Mite Away Quick Strips) | Yes | Dry sugar | sugar only |
| 1963 | 2021 | 12.66299 | Yes | 48 hr drop (sticky board), Sugar roll, Drone brood inspection | Yes | Formic Acid (Mite Away Quick Strips) | Yes | Dry sugar | sugar only |
| 1964 | 2021 | 0.968524 | No | NA | Yes | Oxalic Acid (Vapor) | Yes | Sugar syrup, Honey from your own stock, Probiotics | none |
| 1965 | 2021 | 0.968524 | No | NA | Yes | Oxalic Acid (Vapor) | Yes | Sugar syrup, Honey from your own stock, Probiotics | none |
| 1966 | 2021 | 0.968524 | No | NA | Yes | Oxalic Acid (Vapor) | Yes | Sugar syrup, Honey from your own stock, Probiotics | none |
| 1967 | 2021 | 0.968524 | No | NA | Yes | Oxalic Acid (Vapor) | Yes | Sugar syrup, Honey from your own stock, Probiotics | none |
| 1968 | 2021 | 0.968524 | No | NA | Yes | Oxalic Acid (Vapor) | Yes | Sugar syrup, Honey from your own stock, Probiotics | none |
| 1969 | 2021 | 0.968524 | No | NA | Yes | Oxalic Acid (Vapor) | Yes | Sugar syrup, Honey from your own stock, Probiotics | none |
| 1970 | 2021 | 0.968524 | No | NA | Yes | Oxalic Acid (Vapor) | Yes | Sugar syrup, Honey from your own stock, Probiotics | none |
| 1971 | 2021 | 0.968524 | No | NA | Yes | Oxalic Acid (Vapor) | Yes | Sugar syrup, Honey from your own stock, Probiotics | none |
| 1972 | 2021 | 0.968524 | No | NA | Yes | Oxalic Acid (Vapor) | Yes | Sugar syrup, Honey from your own stock, Probiotics | none |
| 1973 | 2021 | 0.968524 | No | NA | Yes | Oxalic Acid (Vapor) | Yes | Sugar syrup, Honey from your own stock, Probiotics | none |
| 1974 | 2021 | 0.587202 | No | NA | Yes | Oxalic Acid (Vapor) | Yes | Honey from your own stock | none |
| 1975 | 2021 | 0.587202 | No | NA | Yes | Oxalic Acid (Vapor) | Yes | Honey from your own stock | none |
| 1976 | 2021 | 0.587202 | No | NA | Yes | Oxalic Acid (Vapor) | Yes | Honey from your own stock | none |
| 1977 | 2021 | 0.587202 | No | NA | Yes | Oxalic Acid (Vapor) | Yes | Honey from your own stock | none |
| 1978 | 2021 | 0.587202 | No | NA | Yes | Oxalic Acid (Vapor) | Yes | Honey from your own stock | none |
| 1979 | 2021 | 0.587202 | No | NA | Yes | Oxalic Acid (Vapor) | Yes | Honey from your own stock | none |
| 1980 | 2021 | 0.587202 | No | NA | Yes | Oxalic Acid (Vapor) | Yes | Honey from your own stock | none |
| 1981 | 2021 | 0.587202 | No | NA | Yes | Oxalic Acid (Vapor) | Yes | Honey from your own stock | none |
| 1982 | 2021 | 0.587202 | No | NA | Yes | Oxalic Acid (Vapor) | Yes | Honey from your own stock | none |
| 1983 | 2021 | 0.587202 | No | NA | Yes | Oxalic Acid (Vapor) | Yes | Honey from your own stock | none |
| 1984 | 2021 | 0.587202 | No | NA | Yes | Oxalic Acid (Vapor) | Yes | Honey from your own stock | none |
| 1985 | 2021 | 0.587202 | No | NA | Yes | Oxalic Acid (Vapor) | Yes | Honey from your own stock | none |
| 1986 | 2021 | 0.587202 | No | NA | Yes | Oxalic Acid (Vapor) | Yes | Honey from your own stock | none |
| 1987 | 2021 | 0.587202 | No | NA | Yes | Oxalic Acid (Vapor) | Yes | Honey from your own stock | none |
| 1988 | 2021 | 0.587202 | No | NA | Yes | Oxalic Acid (Vapor) | Yes | Honey from your own stock | none |
| 1989 | 2021 | 0.587202 | No | NA | Yes | Oxalic Acid (Vapor) | Yes | Honey from your own stock | none |
| 1990 | 2021 | 0.587202 | No | NA | Yes | Oxalic Acid (Vapor) | Yes | Honey from your own stock | none |
| 1991 | 2021 | 0.587202 | No | NA | Yes | Oxalic Acid (Vapor) | Yes | Honey from your own stock | none |
| 1992 | 2021 | 0.587202 | No | NA | Yes | Oxalic Acid (Vapor) | Yes | Honey from your own stock | none |
| 1993 | 2021 | 0.587202 | No | NA | Yes | Oxalic Acid (Vapor) | Yes | Honey from your own stock | none |
| 1994 | 2021 | 0.587202 | No | NA | Yes | Oxalic Acid (Vapor) | Yes | Honey from your own stock | none |
| 1995 | 2021 | 0.587202 | No | NA | Yes | Oxalic Acid (Vapor) | Yes | Honey from your own stock | none |
| 1996 | 2021 | 0.587202 | No | NA | Yes | Oxalic Acid (Vapor) | Yes | Honey from your own stock | none |
| 1997 | 2021 | 20.76275 | No | NA | Yes | Oxalic Acid (Vapor) | Yes | Dry sugar | sugar only |
| 1998 | 2021 | 20.76275 | No | NA | Yes | Oxalic Acid (Vapor) | Yes | Dry sugar | sugar only |
| 1999 | 2021 | 20.76275 | No | NA | Yes | Oxalic Acid (Vapor) | Yes | Dry sugar | sugar only |
| 2000 | 2021 | 4.06778 | Yes | Other | Yes | Formic Acid (Mite Away Quick Strips) | Yes | Sugar syrup, Pollen substitute, Honey from your own stock, Commercially available supplements | none |
| 2001 | 2021 | 4.06778 | Yes | Other | Yes | Formic Acid (Mite Away Quick Strips) | Yes | Sugar syrup, Pollen substitute, Honey from your own stock, Commercially available supplements | none |
| 2002 | 2021 | 4.06778 | Yes | Other | Yes | Formic Acid (Mite Away Quick Strips) | Yes | Sugar syrup, Pollen substitute, Honey from your own stock, Commercially available supplements | none |
| 2003 | 2021 | 4.06778 | Yes | Other | Yes | Formic Acid (Mite Away Quick Strips) | Yes | Sugar syrup, Pollen substitute, Honey from your own stock, Commercially available supplements | none |
| 2004 | 2021 | 4.06778 | Yes | Other | Yes | Formic Acid (Mite Away Quick Strips) | Yes | Sugar syrup, Pollen substitute, Honey from your own stock, Commercially available supplements | none |
| 2005 | 2021 | 44.9972 | Yes | Sugar roll | Yes | Oxalic Acid (Dribble) | Yes | Fondant or sugar candy | sugar only |
| 2006 | 2021 | 44.9972 | Yes | Sugar roll | Yes | Oxalic Acid (Dribble) | Yes | Fondant or sugar candy | sugar only |
| 2007 | 2021 | 22.2137 | Yes | Other | Yes | Formic Acid (Mite Away Quick Strips) | Yes | Sugar syrup | none |
| 2008 | 2021 | 22.2137 | Yes | Other | Yes | Formic Acid (Mite Away Quick Strips) | Yes | Sugar syrup | none |
| 2009 | 2021 | 22.2137 | Yes | Other | Yes | Formic Acid (Mite Away Quick Strips) | Yes | Sugar syrup | none |
| 2010 | 2021 | 22.2137 | Yes | Other | Yes | Formic Acid (Mite Away Quick Strips) | Yes | Sugar syrup | none |
| 2011 | 2021 | 22.2137 | Yes | Other | Yes | Formic Acid (Mite Away Quick Strips) | Yes | Sugar syrup | none |
| 2012 | 2021 | 22.2137 | Yes | Other | Yes | Formic Acid (Mite Away Quick Strips) | Yes | Sugar syrup | none |
| 2013 | 2021 | 22.2137 | Yes | Other | Yes | Formic Acid (Mite Away Quick Strips) | Yes | Sugar syrup | none |
| 2014 | 2021 | 22.2137 | Yes | Other | Yes | Formic Acid (Mite Away Quick Strips) | Yes | Sugar syrup | none |
| 2015 | 2021 | 22.2137 | Yes | Other | Yes | Formic Acid (Mite Away Quick Strips) | Yes | Sugar syrup | none |
| 2016 | 2021 | 5.586938 | Yes | Sugar roll | Yes | Oxalic Acid (Vapor) | Yes | Fondant or sugar candy, Other | none |
| 2017 | 2021 | 5.586938 | Yes | Sugar roll | Yes | Oxalic Acid (Vapor) | Yes | Fondant or sugar candy, Other | none |
| 2018 | 2021 | 5.586938 | Yes | Sugar roll | Yes | Oxalic Acid (Vapor) | Yes | Fondant or sugar candy, Other | none |
| 2019 | 2021 | 5.586938 | Yes | Sugar roll | Yes | Oxalic Acid (Vapor) | Yes | Fondant or sugar candy, Other | none |
| 2020 | 2021 | 5.586938 | Yes | Sugar roll | Yes | Oxalic Acid (Vapor) | Yes | Fondant or sugar candy, Other | none |
| 2021 | 2021 | 9.139325 | Yes | Alcohol wash | Yes | Oxalic Acid (Vapor) | Yes | Fondant or sugar candy, Dry sugar, Pollen substitute, Commercially available supplements | none |
| 2022 | 2021 | 9.139325 | Yes | Alcohol wash | Yes | Oxalic Acid (Vapor) | Yes | Fondant or sugar candy, Dry sugar, Pollen substitute, Commercially available supplements | none |
| 2023 | 2021 | 1.049819 | Yes | Alcohol wash | Yes | Oxalic Acid (Vapor) | Yes | Fondant or sugar candy, Dry sugar | none |
| 2024 | 2021 | 1.049819 | Yes | Alcohol wash | Yes | Oxalic Acid (Vapor) | Yes | Fondant or sugar candy, Dry sugar | none |
| 2025 | 2021 | 1.049819 | Yes | Alcohol wash | Yes | Oxalic Acid (Vapor) | Yes | Fondant or sugar candy, Dry sugar | none |
| 2026 | 2021 | 1.049819 | Yes | Alcohol wash | Yes | Oxalic Acid (Vapor) | Yes | Fondant or sugar candy, Dry sugar | none |
| 2027 | 2021 | 1.049819 | Yes | Alcohol wash | Yes | Oxalic Acid (Vapor) | Yes | Fondant or sugar candy, Dry sugar | none |
| 2028 | 2021 | 1.049819 | Yes | Alcohol wash | Yes | Oxalic Acid (Vapor) | Yes | Fondant or sugar candy, Dry sugar | none |
| 2029 | 2021 | 1.049819 | Yes | Alcohol wash | Yes | Oxalic Acid (Vapor) | Yes | Fondant or sugar candy, Dry sugar | none |
| 2030 | 2021 | 1.049819 | Yes | Alcohol wash | Yes | Oxalic Acid (Vapor) | Yes | Fondant or sugar candy, Dry sugar | none |
| 2031 | 2021 | 1.049819 | Yes | Alcohol wash | Yes | Oxalic Acid (Vapor) | Yes | Fondant or sugar candy, Dry sugar | none |
| 2032 | 2021 | 1.049819 | Yes | Alcohol wash | Yes | Oxalic Acid (Vapor) | Yes | Fondant or sugar candy, Dry sugar | none |
| 2033 | 2021 | 1.049819 | Yes | Alcohol wash | Yes | Oxalic Acid (Vapor) | Yes | Fondant or sugar candy, Dry sugar | none |
| 2034 | 2021 | 1.049819 | Yes | Alcohol wash | Yes | Oxalic Acid (Vapor) | Yes | Fondant or sugar candy, Dry sugar | none |
| 2035 | 2021 | 6.765008 | Yes | Other | Yes | Formic Acid (Mite Away Quick Strips) | Yes | Sugar syrup | none |
| 2036 | 2021 | 6.765008 | Yes | Other | Yes | Formic Acid (Mite Away Quick Strips) | Yes | Sugar syrup | none |
| 2037 | 2021 | 2.584815 | Yes | Alcohol wash, Drone brood inspection | Yes | Formic Acid (Mite Away Quick Strips) | Yes | Fondant or sugar candy, Honey from your own stock | none |
| 2038 | 2021 | 2.584815 | Yes | Alcohol wash, Drone brood inspection | Yes | Formic Acid (Mite Away Quick Strips) | Yes | Fondant or sugar candy, Honey from your own stock | none |
| 2039 | 2021 | 12.34639 | Yes | Sugar roll | Yes | Formic Acid (Mite Away Quick Strips) | Yes | Pollen substitute, Commercially available supplements | none |
| 2040 | 2021 | 12.34639 | Yes | Sugar roll | Yes | Formic Acid (Mite Away Quick Strips) | Yes | Pollen substitute, Commercially available supplements | none |
| 2041 | 2021 | 12.34639 | Yes | Sugar roll | Yes | Formic Acid (Mite Away Quick Strips) | Yes | Pollen substitute, Commercially available supplements | none |
| 2042 | 2021 | 12.34639 | Yes | Sugar roll | Yes | Formic Acid (Mite Away Quick Strips) | Yes | Pollen substitute, Commercially available supplements | none |
| 2043 | 2021 | 12.34639 | Yes | Sugar roll | Yes | Formic Acid (Mite Away Quick Strips) | Yes | Pollen substitute, Commercially available supplements | none |
| 2044 | 2021 | 12.34639 | Yes | Sugar roll | Yes | Formic Acid (Mite Away Quick Strips) | Yes | Pollen substitute, Commercially available supplements | none |
| 2045 | 2021 | 12.34639 | Yes | Sugar roll | Yes | Formic Acid (Mite Away Quick Strips) | Yes | Pollen substitute, Commercially available supplements | none |
| 2046 | 2021 | 12.34639 | Yes | Sugar roll | Yes | Formic Acid (Mite Away Quick Strips) | Yes | Pollen substitute, Commercially available supplements | none |
| 2047 | 2021 | 12.34639 | Yes | Sugar roll | Yes | Formic Acid (Mite Away Quick Strips) | Yes | Pollen substitute, Commercially available supplements | none |
| 2048 | 2021 | 12.34639 | Yes | Sugar roll | Yes | Formic Acid (Mite Away Quick Strips) | Yes | Pollen substitute, Commercially available supplements | none |
| 2049 | 2021 | 2.851477 | Yes | Other | Yes | Formic Acid (Mite Away Quick Strips) | Yes | Fondant or sugar candy | sugar only |
| 2050 | 2021 | 2.851477 | Yes | Other | Yes | Formic Acid (Mite Away Quick Strips) | Yes | Fondant or sugar candy | sugar only |
| 2051 | 2021 | 2.851477 | Yes | Other | Yes | Formic Acid (Mite Away Quick Strips) | Yes | Fondant or sugar candy | sugar only |
| 2052 | 2021 | 2.851477 | Yes | Other | Yes | Formic Acid (Mite Away Quick Strips) | Yes | Fondant or sugar candy | sugar only |
| 2053 | 2021 | 2.851477 | Yes | Other | Yes | Formic Acid (Mite Away Quick Strips) | Yes | Fondant or sugar candy | sugar only |
| 2054 | 2021 | 2.851477 | Yes | Other | Yes | Formic Acid (Mite Away Quick Strips) | Yes | Fondant or sugar candy | sugar only |
| 2055 | 2021 | 2.851477 | Yes | Other | Yes | Formic Acid (Mite Away Quick Strips) | Yes | Fondant or sugar candy | sugar only |
| 2056 | 2021 | 34.33544 | Yes | Sugar roll | Yes | Formic Acid (Mite Away Quick Strips) | Yes | Fondant or sugar candy, Dry sugar | none |
| 2057 | 2021 | 34.33544 | Yes | Sugar roll | Yes | Formic Acid (Mite Away Quick Strips) | Yes | Fondant or sugar candy, Dry sugar | none |
| 2058 | 2021 | 2.594102 | Yes | Sugar roll | Yes | Formic Acid (Mite Away Quick Strips) | Yes | Fondant or sugar candy, Honey from your own stock | none |
| 2059 | 2021 | 2.594102 | Yes | Sugar roll | Yes | Formic Acid (Mite Away Quick Strips) | Yes | Fondant or sugar candy, Honey from your own stock | none |
| 2060 | 2021 | 7.641497 | No | NA | Yes | Formic Acid (Mite Away Quick Strips) | Yes | Fondant or sugar candy, Honey from your own stock, Pollen from your own stock | none |
| 2061 | 2021 | 7.641497 | No | NA | Yes | Formic Acid (Mite Away Quick Strips) | Yes | Fondant or sugar candy, Honey from your own stock, Pollen from your own stock | none |
| 2062 | 2021 | 3.320934 | Yes | Alcohol wash | Yes | Formic Acid (Mite Away Quick Strips) | Yes | Fondant or sugar candy | sugar only |
| 2063 | 2021 | 3.320934 | Yes | Alcohol wash | Yes | Formic Acid (Mite Away Quick Strips) | Yes | Fondant or sugar candy | sugar only |
| 2064 | 2021 | 3.320934 | Yes | Alcohol wash | Yes | Formic Acid (Mite Away Quick Strips) | Yes | Fondant or sugar candy | sugar only |
| 2065 | 2021 | 1.750746 | No | NA | Yes | Formic Acid (Mite Away Quick Strips) | Yes | Commercially available supplements | none |
| 2066 | 2021 | 2.324817 | Yes | Sugar roll | Yes | Oxalic Acid (Vapor) | Yes | Fondant or sugar candy, Honey from your own stock, Pollen from your own stock | none |
| 2067 | 2021 | 2.324817 | Yes | Sugar roll | Yes | Oxalic Acid (Vapor) | Yes | Fondant or sugar candy, Honey from your own stock, Pollen from your own stock | none |
| 2068 | 2021 | 3.661819 | Yes | Sugar roll | Yes | Formic Acid (Mite Away Quick Strips) | Yes | Fondant or sugar candy, Pollen substitute, Honey from your own stock | none |
| 2069 | 2021 | 3.661819 | Yes | Sugar roll | Yes | Formic Acid (Mite Away Quick Strips) | Yes | Fondant or sugar candy, Pollen substitute, Honey from your own stock | none |
| 2070 | 2021 | 3.661819 | Yes | Sugar roll | Yes | Formic Acid (Mite Away Quick Strips) | Yes | Fondant or sugar candy, Pollen substitute, Honey from your own stock | none |
| 2071 | 2021 | 3.661819 | Yes | Sugar roll | Yes | Formic Acid (Mite Away Quick Strips) | Yes | Fondant or sugar candy, Pollen substitute, Honey from your own stock | none |
| 2072 | 2021 | 3.661819 | Yes | Sugar roll | Yes | Formic Acid (Mite Away Quick Strips) | Yes | Fondant or sugar candy, Pollen substitute, Honey from your own stock | none |
| 2073 | 2021 | 3.661819 | Yes | Sugar roll | Yes | Formic Acid (Mite Away Quick Strips) | Yes | Fondant or sugar candy, Pollen substitute, Honey from your own stock | none |
| 2074 | 2021 | 6.539953 | Yes | Sugar roll | Yes | Formic Acid (Mite Away Quick Strips) | Yes | Sugar syrup | none |
| 2075 | 2021 | 6.539953 | Yes | Sugar roll | Yes | Formic Acid (Mite Away Quick Strips) | Yes | Sugar syrup | none |
| 2076 | 2021 | 26.32312 | Yes | Other | Yes | Formic Acid (Mite Away Quick Strips) | Yes | Dry sugar | sugar only |
| 2077 | 2021 | 26.32312 | Yes | Other | Yes | Formic Acid (Mite Away Quick Strips) | Yes | Dry sugar | sugar only |
| 2078 | 2021 | 26.32312 | Yes | Other | Yes | Formic Acid (Mite Away Quick Strips) | Yes | Dry sugar | sugar only |
| 2079 | 2021 | 0.355692 | Yes | Alcohol wash | Yes | Apivar (Amitraz) | Yes | Fondant or sugar candy, Sugar syrup | none |
| 2080 | 2021 | 0.355692 | Yes | Alcohol wash | Yes | Apivar (Amitraz) | Yes | Fondant or sugar candy, Sugar syrup | none |
| 2081 | 2021 | 0.355692 | Yes | Alcohol wash | Yes | Apivar (Amitraz) | Yes | Fondant or sugar candy, Sugar syrup | none |
| 2082 | 2021 | 0.355692 | Yes | Alcohol wash | Yes | Apivar (Amitraz) | Yes | Fondant or sugar candy, Sugar syrup | none |
| 2083 | 2021 | 1.575448 | Yes | Drone brood inspection | Yes | Oxalic Acid (Dribble) | Yes | Fondant or sugar candy | sugar only |
| 2084 | 2021 | 1.575448 | Yes | Drone brood inspection | Yes | Oxalic Acid (Dribble) | Yes | Fondant or sugar candy | sugar only |
| 2085 | 2021 | 10.45588 | Yes | Alcohol wash | Yes | Oxalic Acid (Vapor) | Yes | Sugar syrup, Dry sugar | none |
| 2086 | 2021 | 10.45588 | Yes | Alcohol wash | Yes | Oxalic Acid (Vapor) | Yes | Sugar syrup, Dry sugar | none |
| 2087 | 2021 | 10.45588 | Yes | Alcohol wash | Yes | Oxalic Acid (Vapor) | Yes | Sugar syrup, Dry sugar | none |
| 2088 | 2021 | 10.45588 | Yes | Alcohol wash | Yes | Oxalic Acid (Vapor) | Yes | Sugar syrup, Dry sugar | none |
| 2089 | 2021 | 8.665201 | Yes | NA | Yes | Oxalic Acid (Vapor) | Yes | Fondant or sugar candy, Honey from your own stock | none |
| 2090 | 2021 | 8.665201 | Yes | NA | Yes | Oxalic Acid (Vapor) | Yes | Fondant or sugar candy, Honey from your own stock | none |
| 2091 | 2021 | 1.5712 | Yes | Sugar roll | Yes | Formic Acid (Mite Away Quick Strips) | Yes | Sugar syrup, Honey from your own stock, Commercially available supplements | none |
| 2092 | 2021 | 1.5712 | Yes | Sugar roll | Yes | Formic Acid (Mite Away Quick Strips) | Yes | Sugar syrup, Honey from your own stock, Commercially available supplements | none |
| 2093 | 2021 | 1.5712 | Yes | Sugar roll | Yes | Formic Acid (Mite Away Quick Strips) | Yes | Sugar syrup, Honey from your own stock, Commercially available supplements | none |
| 2094 | 2021 | 1.5712 | Yes | Sugar roll | Yes | Formic Acid (Mite Away Quick Strips) | Yes | Sugar syrup, Honey from your own stock, Commercially available supplements | none |
| 2095 | 2021 | 0.635095 | Yes | 48 hr drop (sticky board), Alcohol wash | Yes | Formic Acid (Mite Away Quick Strips) | Yes | Fondant or sugar candy | sugar only |
| 2096 | 2021 | 0.635095 | Yes | 48 hr drop (sticky board), Alcohol wash | Yes | Formic Acid (Mite Away Quick Strips) | Yes | Fondant or sugar candy | sugar only |
| 2097 | 2021 | 0.635095 | Yes | 48 hr drop (sticky board), Alcohol wash | Yes | Formic Acid (Mite Away Quick Strips) | Yes | Fondant or sugar candy | sugar only |
| 2098 | 2021 | 2.347588 | No | NA | Yes | Formic Acid (Mite Away Quick Strips) | Yes | Fondant or sugar candy, Pollen substitute | none |
| 2099 | 2021 | 2.347588 | No | NA | Yes | Formic Acid (Mite Away Quick Strips) | Yes | Fondant or sugar candy, Pollen substitute | none |
| 2100 | 2021 | 2.347588 | No | NA | Yes | Formic Acid (Mite Away Quick Strips) | Yes | Fondant or sugar candy, Pollen substitute | none |
| 2101 | 2021 | 2.347588 | No | NA | Yes | Formic Acid (Mite Away Quick Strips) | Yes | Fondant or sugar candy, Pollen substitute | none |
| 2102 | 2021 | 2.347588 | No | NA | Yes | Formic Acid (Mite Away Quick Strips) | Yes | Fondant or sugar candy, Pollen substitute | none |
| 2103 | 2021 | 2.347588 | No | NA | Yes | Formic Acid (Mite Away Quick Strips) | Yes | Fondant or sugar candy, Pollen substitute | none |
| 2104 | 2022 | 0.566045 | No | NA | Yes | Formic Acid (Mite Away Quick Strips or Formic Pro) | Yes | Dry sugar, Pollen substitute | none |
| 2105 | 2022 | 0.566045 | No | NA | Yes | Formic Acid (Mite Away Quick Strips or Formic Pro) | Yes | Dry sugar, Pollen substitute | none |
| 2106 | 2022 | 0.566045 | No | NA | Yes | Formic Acid (Mite Away Quick Strips or Formic Pro) | Yes | Dry sugar, Pollen substitute | none |
| 2107 | 2022 | 0.566045 | No | NA | Yes | Formic Acid (Mite Away Quick Strips or Formic Pro) | Yes | Dry sugar, Pollen substitute | none |
| 2108 | 2022 | 1.117303 | Yes | Alcohol wash | Yes | Oxalic Acid (Vapor) | No | NA | none |
| 2109 | 2022 | 1.117303 | Yes | Alcohol wash | Yes | Oxalic Acid (Vapor) | No | NA | none |
| 2110 | 2022 | 1.117303 | Yes | Alcohol wash | Yes | Oxalic Acid (Vapor) | No | NA | none |
| 2111 | 2022 | 1.117303 | Yes | Alcohol wash | Yes | Oxalic Acid (Vapor) | No | NA | none |
| 2112 | 2022 | 1.117303 | Yes | Alcohol wash | Yes | Oxalic Acid (Vapor) | No | NA | none |
| 2113 | 2022 | 1.117303 | Yes | Alcohol wash | Yes | Oxalic Acid (Vapor) | No | NA | none |
| 2114 | 2022 | 1.117303 | Yes | Alcohol wash | Yes | Oxalic Acid (Vapor) | No | NA | none |
| 2115 | 2022 | 1.117303 | Yes | Alcohol wash | Yes | Oxalic Acid (Vapor) | No | NA | none |
| 2116 | 2022 | 1.117303 | Yes | Alcohol wash | Yes | Oxalic Acid (Vapor) | No | NA | none |
| 2117 | 2022 | 1.117303 | Yes | Alcohol wash | Yes | Oxalic Acid (Vapor) | No | NA | none |
| 2118 | 2022 | 1.117303 | Yes | Alcohol wash | Yes | Oxalic Acid (Vapor) | No | NA | none |
| 2119 | 2022 | 6.262603 | Yes | Other | Yes | Formic Acid (Mite Away Quick Strips or Formic Pro) | Yes | Fondant or sugar candy, Dry sugar | none |
| 2120 | 2022 | 6.262603 | Yes | Other | Yes | Formic Acid (Mite Away Quick Strips or Formic Pro) | Yes | Fondant or sugar candy, Dry sugar | none |
| 2121 | 2022 | 6.262603 | Yes | Other | Yes | Formic Acid (Mite Away Quick Strips or Formic Pro) | Yes | Fondant or sugar candy, Dry sugar | none |
| 2122 | 2022 | 6.262603 | Yes | Other | Yes | Formic Acid (Mite Away Quick Strips or Formic Pro) | Yes | Fondant or sugar candy, Dry sugar | none |
| 2123 | 2022 | 6.262603 | Yes | Other | Yes | Formic Acid (Mite Away Quick Strips or Formic Pro) | Yes | Fondant or sugar candy, Dry sugar | none |
| 2124 | 2022 | 7.561179 | Yes | 48 hr drop (sticky board) | Yes | Formic Acid (Mite Away Quick Strips or Formic Pro) | Yes | Other | none |
| 2125 | 2022 | 7.561179 | Yes | 48 hr drop (sticky board) | Yes | Formic Acid (Mite Away Quick Strips or Formic Pro) | Yes | Other | none |
| 2126 | 2022 | 7.561179 | Yes | 48 hr drop (sticky board) | Yes | Formic Acid (Mite Away Quick Strips or Formic Pro) | Yes | Other | none |
| 2127 | 2022 | 7.561179 | Yes | 48 hr drop (sticky board) | Yes | Formic Acid (Mite Away Quick Strips or Formic Pro) | Yes | Other | none |
| 2128 | 2022 | 7.561179 | Yes | 48 hr drop (sticky board) | Yes | Formic Acid (Mite Away Quick Strips or Formic Pro) | Yes | Other | none |
| 2129 | 2022 | 7.561179 | Yes | 48 hr drop (sticky board) | Yes | Formic Acid (Mite Away Quick Strips or Formic Pro) | Yes | Other | none |
| 2130 | 2022 | 7.561179 | Yes | 48 hr drop (sticky board) | Yes | Formic Acid (Mite Away Quick Strips or Formic Pro) | Yes | Other | none |
| 2131 | 2022 | 7.561179 | Yes | 48 hr drop (sticky board) | Yes | Formic Acid (Mite Away Quick Strips or Formic Pro) | Yes | Other | none |
| 2132 | 2022 | 5.169285 | Yes | Sugar roll, Alcohol wash | Yes | Formic Acid (Mite Away Quick Strips or Formic Pro) | Yes | Fondant or sugar candy, Honey from your own stock, Pollen from your own stock | none |
| 2133 | 2022 | 5.169285 | Yes | Sugar roll, Alcohol wash | Yes | Formic Acid (Mite Away Quick Strips or Formic Pro) | Yes | Fondant or sugar candy, Honey from your own stock, Pollen from your own stock | none |
| 2134 | 2022 | 5.169285 | Yes | Sugar roll, Alcohol wash | Yes | Formic Acid (Mite Away Quick Strips or Formic Pro) | Yes | Fondant or sugar candy, Honey from your own stock, Pollen from your own stock | none |
| 2135 | 2022 | 1.038678 | Yes | Alcohol wash | Yes | Formic Acid (Mite Away Quick Strips or Formic Pro) | Yes | Fondant or sugar candy, Dry sugar | none |
| 2136 | 2022 | 1.038678 | Yes | Alcohol wash | Yes | Formic Acid (Mite Away Quick Strips or Formic Pro) | Yes | Fondant or sugar candy, Dry sugar | none |
| 2137 | 2022 | 1.038678 | Yes | Alcohol wash | Yes | Formic Acid (Mite Away Quick Strips or Formic Pro) | Yes | Fondant or sugar candy, Dry sugar | none |
| 2138 | 2022 | 1.038678 | Yes | Alcohol wash | Yes | Formic Acid (Mite Away Quick Strips or Formic Pro) | Yes | Fondant or sugar candy, Dry sugar | none |
| 2139 | 2022 | 1.038678 | Yes | Alcohol wash | Yes | Formic Acid (Mite Away Quick Strips or Formic Pro) | Yes | Fondant or sugar candy, Dry sugar | none |
| 2140 | 2022 | 1.038678 | Yes | Alcohol wash | Yes | Formic Acid (Mite Away Quick Strips or Formic Pro) | Yes | Fondant or sugar candy, Dry sugar | none |
| 2141 | 2022 | 2.30783 | Yes | Sugar roll | Yes | Formic Acid (Mite Away Quick Strips or Formic Pro) | Yes | Honey from your own stock, Pollen from your own stock | none |
| 2142 | 2022 | 2.30783 | Yes | Sugar roll | Yes | Formic Acid (Mite Away Quick Strips or Formic Pro) | Yes | Honey from your own stock, Pollen from your own stock | none |
| 2143 | 2022 | 2.30783 | Yes | Sugar roll | Yes | Formic Acid (Mite Away Quick Strips or Formic Pro) | Yes | Honey from your own stock, Pollen from your own stock | none |
| 2144 | 2022 | 14.77226 | Yes | Alcohol wash | Yes | Formic Acid (Mite Away Quick Strips or Formic Pro) | Yes | Fondant or sugar candy, Sugar syrup | none |
| 2145 | 2022 | 14.77226 | Yes | Alcohol wash | Yes | Formic Acid (Mite Away Quick Strips or Formic Pro) | Yes | Fondant or sugar candy, Sugar syrup | none |
| 2146 | 2022 | 14.77226 | Yes | Alcohol wash | Yes | Formic Acid (Mite Away Quick Strips or Formic Pro) | Yes | Fondant or sugar candy, Sugar syrup | none |
| 2147 | 2022 | 14.77226 | Yes | Alcohol wash | Yes | Formic Acid (Mite Away Quick Strips or Formic Pro) | Yes | Fondant or sugar candy, Sugar syrup | none |
| 2148 | 2022 | 14.77226 | Yes | Alcohol wash | Yes | Formic Acid (Mite Away Quick Strips or Formic Pro) | Yes | Fondant or sugar candy, Sugar syrup | none |
| 2149 | 2022 | 14.77226 | Yes | Alcohol wash | Yes | Formic Acid (Mite Away Quick Strips or Formic Pro) | Yes | Fondant or sugar candy, Sugar syrup | none |
| 2150 | 2022 | 9.59422 | Yes | 48 hr drop (sticky board) | Yes | Formic Acid (Mite Away Quick Strips or Formic Pro) | Yes | Fondant or sugar candy | sugar only |
| 2151 | 2022 | 9.59422 | Yes | 48 hr drop (sticky board) | Yes | Formic Acid (Mite Away Quick Strips or Formic Pro) | Yes | Fondant or sugar candy | sugar only |
| 2152 | 2022 | 9.59422 | Yes | 48 hr drop (sticky board) | Yes | Formic Acid (Mite Away Quick Strips or Formic Pro) | Yes | Fondant or sugar candy | sugar only |
| 2153 | 2022 | 22.45908 | Yes | Alcohol wash | Yes | Oxalic Acid (Dribble) | Yes | Dry sugar, Pollen substitute | none |
| 2154 | 2022 | 22.45908 | Yes | Alcohol wash | Yes | Oxalic Acid (Dribble) | Yes | Dry sugar, Pollen substitute | none |
| 2155 | 2022 | 2.066258 | Yes | 48 hr drop (sticky board), Drone brood inspection | Yes | Formic Acid (Mite Away Quick Strips or Formic Pro) | Yes | Fondant or sugar candy | sugar only |
| 2156 | 2022 | 2.066258 | Yes | 48 hr drop (sticky board), Drone brood inspection | Yes | Formic Acid (Mite Away Quick Strips or Formic Pro) | Yes | Fondant or sugar candy | sugar only |
| 2157 | 2022 | 2.066258 | Yes | 48 hr drop (sticky board), Drone brood inspection | Yes | Formic Acid (Mite Away Quick Strips or Formic Pro) | Yes | Fondant or sugar candy | sugar only |
| 2158 | 2022 | 2.066258 | Yes | 48 hr drop (sticky board), Drone brood inspection | Yes | Formic Acid (Mite Away Quick Strips or Formic Pro) | Yes | Fondant or sugar candy | sugar only |
| 2159 | 2022 | 11.94639 | Yes | Drone brood inspection | Yes | Oxalic Acid (Vapor) | Yes | Fondant or sugar candy, Dry sugar, Pollen substitute, Honey from your own stock | none |
| 2160 | 2022 | 11.94639 | Yes | Drone brood inspection | Yes | Oxalic Acid (Vapor) | Yes | Fondant or sugar candy, Dry sugar, Pollen substitute, Honey from your own stock | none |
| 2161 | 2022 | 11.94639 | Yes | Drone brood inspection | Yes | Oxalic Acid (Vapor) | Yes | Fondant or sugar candy, Dry sugar, Pollen substitute, Honey from your own stock | none |
| 2162 | 2022 | 11.94639 | Yes | Drone brood inspection | Yes | Oxalic Acid (Vapor) | Yes | Fondant or sugar candy, Dry sugar, Pollen substitute, Honey from your own stock | none |
| 2163 | 2022 | 11.94639 | Yes | Drone brood inspection | Yes | Oxalic Acid (Vapor) | Yes | Fondant or sugar candy, Dry sugar, Pollen substitute, Honey from your own stock | none |
| 2164 | 2022 | 16.41964 | Yes | Alcohol wash | Yes | Formic Acid (Mite Away Quick Strips or Formic Pro) | Yes | Fondant or sugar candy | sugar only |
| 2165 | 2022 | 16.41964 | Yes | Alcohol wash | Yes | Formic Acid (Mite Away Quick Strips or Formic Pro) | Yes | Fondant or sugar candy | sugar only |
| 2166 | 2022 | 16.41964 | Yes | Alcohol wash | Yes | Formic Acid (Mite Away Quick Strips or Formic Pro) | Yes | Fondant or sugar candy | sugar only |
| 2167 | 2022 | 21.7249 | No | NA | Yes | Oxalic Acid (Vapor) | Yes | Sugar syrup, Pollen substitute | none |
| 2168 | 2022 | 21.7249 | No | NA | Yes | Oxalic Acid (Vapor) | Yes | Sugar syrup, Pollen substitute | none |
| 2169 | 2022 | 21.7249 | No | NA | Yes | Oxalic Acid (Vapor) | Yes | Sugar syrup, Pollen substitute | none |
| 2170 | 2022 | 21.7249 | No | NA | Yes | Oxalic Acid (Vapor) | Yes | Sugar syrup, Pollen substitute | none |
| 2171 | 2022 | 4.658693 | Yes | 48 hr drop (sticky board), Alcohol wash | Yes | Formic Acid (Mite Away Quick Strips or Formic Pro) | Yes | Fondant or sugar candy, Honey from your own stock | none |
| 2172 | 2022 | 4.658693 | Yes | 48 hr drop (sticky board), Alcohol wash | Yes | Formic Acid (Mite Away Quick Strips or Formic Pro) | Yes | Fondant or sugar candy, Honey from your own stock | none |
| 2173 | 2022 | 4.658693 | Yes | 48 hr drop (sticky board), Alcohol wash | Yes | Formic Acid (Mite Away Quick Strips or Formic Pro) | Yes | Fondant or sugar candy, Honey from your own stock | none |
| 2174 | 2022 | 4.658693 | Yes | 48 hr drop (sticky board), Alcohol wash | Yes | Formic Acid (Mite Away Quick Strips or Formic Pro) | Yes | Fondant or sugar candy, Honey from your own stock | none |
| 2175 | 2022 | 4.658693 | Yes | 48 hr drop (sticky board), Alcohol wash | Yes | Formic Acid (Mite Away Quick Strips or Formic Pro) | Yes | Fondant or sugar candy, Honey from your own stock | none |
| 2176 | 2022 | 4.658693 | Yes | 48 hr drop (sticky board), Alcohol wash | Yes | Formic Acid (Mite Away Quick Strips or Formic Pro) | Yes | Fondant or sugar candy, Honey from your own stock | none |
| 2177 | 2022 | 4.658693 | Yes | 48 hr drop (sticky board), Alcohol wash | Yes | Formic Acid (Mite Away Quick Strips or Formic Pro) | Yes | Fondant or sugar candy, Honey from your own stock | none |
| 2178 | 2022 | 4.658693 | Yes | 48 hr drop (sticky board), Alcohol wash | Yes | Formic Acid (Mite Away Quick Strips or Formic Pro) | Yes | Fondant or sugar candy, Honey from your own stock | none |
| 2179 | 2022 | 4.658693 | Yes | 48 hr drop (sticky board), Alcohol wash | Yes | Formic Acid (Mite Away Quick Strips or Formic Pro) | Yes | Fondant or sugar candy, Honey from your own stock | none |
| 2180 | 2022 | 4.658693 | Yes | 48 hr drop (sticky board), Alcohol wash | Yes | Formic Acid (Mite Away Quick Strips or Formic Pro) | Yes | Fondant or sugar candy, Honey from your own stock | none |
| 2181 | 2022 | 4.658693 | Yes | 48 hr drop (sticky board), Alcohol wash | Yes | Formic Acid (Mite Away Quick Strips or Formic Pro) | Yes | Fondant or sugar candy, Honey from your own stock | none |
| 2182 | 2022 | 6.845743 | Yes | Alcohol wash | Yes | Oxalic Acid (Vapor) | Yes | Dry sugar | sugar only |
| 2183 | 2022 | 6.845743 | Yes | Alcohol wash | Yes | Oxalic Acid (Vapor) | Yes | Dry sugar | sugar only |
| 2184 | 2022 | 7.257495 | No | NA | Yes | Formic Acid (Mite Away Quick Strips or Formic Pro) | Yes | Fondant or sugar candy | sugar only |
| 2185 | 2022 | 7.257495 | No | NA | Yes | Formic Acid (Mite Away Quick Strips or Formic Pro) | Yes | Fondant or sugar candy | sugar only |
| 2186 | 2022 | 0.417825 | Yes | Sugar roll | Yes | Formic Acid (Mite Away Quick Strips or Formic Pro) | Yes | Fondant or sugar candy, Pollen substitute, Commercially available supplements | none |
| 2187 | 2022 | 0.417825 | Yes | Sugar roll | Yes | Formic Acid (Mite Away Quick Strips or Formic Pro) | Yes | Fondant or sugar candy, Pollen substitute, Commercially available supplements | none |
| 2188 | 2022 | 4.676882 | Yes | Alcohol wash | No | none | Yes | Pollen substitute, Honey from your own stock, Commercially available supplements | none |
| 2189 | 2022 | 4.676882 | Yes | Alcohol wash | No | none | Yes | Pollen substitute, Honey from your own stock, Commercially available supplements | none |
| 2190 | 2022 | 4.676882 | Yes | Alcohol wash | No | none | Yes | Pollen substitute, Honey from your own stock, Commercially available supplements | none |
| 2191 | 2022 | 4.676882 | Yes | Alcohol wash | No | none | Yes | Pollen substitute, Honey from your own stock, Commercially available supplements | none |
| 2192 | 2022 | 4.676882 | Yes | Alcohol wash | No | none | Yes | Pollen substitute, Honey from your own stock, Commercially available supplements | none |
| 2193 | 2022 | 6.971424 | No | NA | Yes | Oxalic Acid (Vapor) | Yes | Fondant or sugar candy, Dry sugar | none |
| 2194 | 2022 | 6.971424 | No | NA | Yes | Oxalic Acid (Vapor) | Yes | Fondant or sugar candy, Dry sugar | none |
| 2195 | 2022 | 10.37933 | Yes | Alcohol wash | Yes | Formic Acid (Mite Away Quick Strips or Formic Pro) | Yes | Fondant or sugar candy, Pollen substitute | none |
| 2196 | 2022 | 10.37933 | Yes | Alcohol wash | Yes | Formic Acid (Mite Away Quick Strips or Formic Pro) | Yes | Fondant or sugar candy, Pollen substitute | none |
| 2197 | 2022 | 5.85445 | Yes | Drone brood inspection | No | none | No | NA | none |
| 2198 | 2022 | 5.85445 | Yes | Drone brood inspection | No | none | No | NA | none |
| 2199 | 2022 | 5.85445 | Yes | Drone brood inspection | No | none | No | NA | none |
| 2200 | 2022 | 5.85445 | Yes | Drone brood inspection | No | none | No | NA | none |
| 2201 | 2022 | 12.00166 | Yes | Sugar roll | Yes | Oxalic Acid (Vapor) | Yes | Dry sugar | sugar only |
| 2202 | 2022 | 12.00166 | Yes | Sugar roll | Yes | Oxalic Acid (Vapor) | Yes | Dry sugar | sugar only |
| 2203 | 2022 | 13.87531 | Yes | 48 hr drop (sticky board) | No | none | Yes | Sugar syrup | none |
| 2204 | 2022 | 8.063537 | Yes | Other | No | none | Yes | Dry sugar | sugar only |
| 2205 | 2022 | 8.063537 | Yes | Other | No | none | Yes | Dry sugar | sugar only |
| 2206 | 2022 | 8.063537 | Yes | Other | No | none | Yes | Dry sugar | sugar only |
| 2207 | 2022 | 8.063537 | Yes | Other | No | none | Yes | Dry sugar | sugar only |
| 2208 | 2022 | 8.063537 | Yes | Other | No | none | Yes | Dry sugar | sugar only |
| 2209 | 2022 | 4.590377 | Yes | Alcohol wash | Yes | Formic Acid (Mite Away Quick Strips or Formic Pro) | Yes | Fondant or sugar candy | sugar only |
| 2210 | 2022 | 4.590377 | Yes | Alcohol wash | Yes | Formic Acid (Mite Away Quick Strips or Formic Pro) | Yes | Fondant or sugar candy | sugar only |
| 2211 | 2022 | 4.590377 | Yes | Alcohol wash | Yes | Formic Acid (Mite Away Quick Strips or Formic Pro) | Yes | Fondant or sugar candy | sugar only |
| 2212 | 2022 | 4.590377 | Yes | Alcohol wash | Yes | Formic Acid (Mite Away Quick Strips or Formic Pro) | Yes | Fondant or sugar candy | sugar only |
| 2213 | 2022 | 4.590377 | Yes | Alcohol wash | Yes | Formic Acid (Mite Away Quick Strips or Formic Pro) | Yes | Fondant or sugar candy | sugar only |
| 2214 | 2022 | 4.590377 | Yes | Alcohol wash | Yes | Formic Acid (Mite Away Quick Strips or Formic Pro) | Yes | Fondant or sugar candy | sugar only |
| 2215 | 2022 | 4.590377 | Yes | Alcohol wash | Yes | Formic Acid (Mite Away Quick Strips or Formic Pro) | Yes | Fondant or sugar candy | sugar only |
| 2216 | 2022 | 13.94939 | Yes | Drone brood inspection | No | none | No | NA | none |
| 2217 | 2022 | 11.69228 | Yes | Sugar roll | No | none | Yes | Dry sugar | sugar only |
| 2218 | 2022 | 11.69228 | Yes | Sugar roll | No | none | Yes | Dry sugar | sugar only |
| 2219 | 2022 | 11.69228 | Yes | Sugar roll | No | none | Yes | Dry sugar | sugar only |
| 2220 | 2022 | 9.409423 | Yes | Drone brood inspection | Yes | Formic Acid (Mite Away Quick Strips or Formic Pro) | Yes | Fondant or sugar candy | sugar only |
| 2221 | 2022 | 9.409423 | Yes | Drone brood inspection | Yes | Formic Acid (Mite Away Quick Strips or Formic Pro) | Yes | Fondant or sugar candy | sugar only |
| 2222 | 2022 | 14.51506 | Yes | 48 hr drop (sticky board) | Yes | Formic Acid (Mite Away Quick Strips or Formic Pro) | Yes | Fondant or sugar candy, Pollen substitute | none |
| 2223 | 2022 | 14.51506 | Yes | 48 hr drop (sticky board) | Yes | Formic Acid (Mite Away Quick Strips or Formic Pro) | Yes | Fondant or sugar candy, Pollen substitute | none |
| 2224 | 2022 | 14.51506 | Yes | 48 hr drop (sticky board) | Yes | Formic Acid (Mite Away Quick Strips or Formic Pro) | Yes | Fondant or sugar candy, Pollen substitute | none |
| 2225 | 2022 | 5.87922 | Yes | Sugar roll | Yes | Oxalic Acid (Vapor) | Yes | Fondant or sugar candy | sugar only |
| 2226 | 2022 | 5.87922 | Yes | Sugar roll | Yes | Oxalic Acid (Vapor) | Yes | Fondant or sugar candy | sugar only |
| 2227 | 2022 | 5.87922 | Yes | Sugar roll | Yes | Oxalic Acid (Vapor) | Yes | Fondant or sugar candy | sugar only |
| 2228 | 2022 | 5.87922 | Yes | Sugar roll | Yes | Oxalic Acid (Vapor) | Yes | Fondant or sugar candy | sugar only |
| 2229 | 2022 | 5.87922 | Yes | Sugar roll | Yes | Oxalic Acid (Vapor) | Yes | Fondant or sugar candy | sugar only |
| 2230 | 2022 | 5.87922 | Yes | Sugar roll | Yes | Oxalic Acid (Vapor) | Yes | Fondant or sugar candy | sugar only |
| 2231 | 2022 | 5.87922 | Yes | Sugar roll | Yes | Oxalic Acid (Vapor) | Yes | Fondant or sugar candy | sugar only |
| 2232 | 2022 | 0.92903 | Yes | Alcohol wash | Yes | Formic Acid (Mite Away Quick Strips or Formic Pro) | Yes | Fondant or sugar candy | sugar only |
| 2233 | 2022 | 0.92903 | Yes | Alcohol wash | Yes | Formic Acid (Mite Away Quick Strips or Formic Pro) | Yes | Fondant or sugar candy | sugar only |
| 2234 | 2022 | 0.92903 | Yes | Alcohol wash | Yes | Formic Acid (Mite Away Quick Strips or Formic Pro) | Yes | Fondant or sugar candy | sugar only |
| 2235 | 2022 | 0.92903 | Yes | Alcohol wash | Yes | Formic Acid (Mite Away Quick Strips or Formic Pro) | Yes | Fondant or sugar candy | sugar only |
| 2236 | 2022 | 0.92903 | Yes | Alcohol wash | Yes | Formic Acid (Mite Away Quick Strips or Formic Pro) | Yes | Fondant or sugar candy | sugar only |
| 2237 | 2022 | 0.92903 | Yes | Alcohol wash | Yes | Formic Acid (Mite Away Quick Strips or Formic Pro) | Yes | Fondant or sugar candy | sugar only |
| 2238 | 2022 | 0.92903 | Yes | Alcohol wash | Yes | Formic Acid (Mite Away Quick Strips or Formic Pro) | Yes | Fondant or sugar candy | sugar only |
| 2239 | 2022 | 0.92903 | Yes | Alcohol wash | Yes | Formic Acid (Mite Away Quick Strips or Formic Pro) | Yes | Fondant or sugar candy | sugar only |
| 2240 | 2022 | 0.92903 | Yes | Alcohol wash | Yes | Formic Acid (Mite Away Quick Strips or Formic Pro) | Yes | Fondant or sugar candy | sugar only |
| 2241 | 2022 | 0.92903 | Yes | Alcohol wash | Yes | Formic Acid (Mite Away Quick Strips or Formic Pro) | Yes | Fondant or sugar candy | sugar only |
| 2242 | 2022 | 0.92903 | Yes | Alcohol wash | Yes | Formic Acid (Mite Away Quick Strips or Formic Pro) | Yes | Fondant or sugar candy | sugar only |
| 2243 | 2022 | 0.92903 | Yes | Alcohol wash | Yes | Formic Acid (Mite Away Quick Strips or Formic Pro) | Yes | Fondant or sugar candy | sugar only |
| 2244 | 2022 | 3.949436 | Yes | Alcohol wash | Yes | Formic Acid (Mite Away Quick Strips or Formic Pro) | Yes | Fondant or sugar candy | sugar only |
| 2245 | 2022 | 3.949436 | Yes | Alcohol wash | Yes | Formic Acid (Mite Away Quick Strips or Formic Pro) | Yes | Fondant or sugar candy | sugar only |
| 2246 | 2022 | 3.949436 | Yes | Alcohol wash | Yes | Formic Acid (Mite Away Quick Strips or Formic Pro) | Yes | Fondant or sugar candy | sugar only |
| 2247 | 2022 | 6.62782 | Yes | 48 hr drop (sticky board) | Yes | Hopguard | No | NA | none |
| 2248 | 2022 | 1.367479 | No | NA | Yes | Apivar (Amitraz) | Yes | Fondant or sugar candy | sugar only |
| 2249 | 2022 | 14.60115 | Yes | Alcohol wash, Drone brood inspection | Yes | Apistan | Yes | Fondant or sugar candy, Pollen substitute | none |
| 2250 | 2022 | 14.60115 | Yes | Alcohol wash, Drone brood inspection | Yes | Apistan | Yes | Fondant or sugar candy, Pollen substitute | none |
| 2251 | 2022 | 14.60115 | Yes | Alcohol wash, Drone brood inspection | Yes | Apistan | Yes | Fondant or sugar candy, Pollen substitute | none |
| 2252 | 2022 | 14.60115 | Yes | Alcohol wash, Drone brood inspection | Yes | Apistan | Yes | Fondant or sugar candy, Pollen substitute | none |
| 2253 | 2022 | 14.60115 | Yes | Alcohol wash, Drone brood inspection | Yes | Apistan | Yes | Fondant or sugar candy, Pollen substitute | none |
| 2254 | 2022 | 2.364437 | Yes | Sugar roll | Yes | Formic Acid (Mite Away Quick Strips or Formic Pro) | Yes | Commercially available supplements | none |
| 2255 | 2022 | 2.364437 | Yes | Sugar roll | Yes | Formic Acid (Mite Away Quick Strips or Formic Pro) | Yes | Commercially available supplements | none |
| 2256 | 2022 | 2.364437 | Yes | Sugar roll | Yes | Formic Acid (Mite Away Quick Strips or Formic Pro) | Yes | Commercially available supplements | none |
| 2257 | 2022 | 2.071962 | No | NA | Yes | none | Yes | Fondant or sugar candy | sugar only |
| 2258 | 2022 | 2.071962 | No | NA | Yes | none | Yes | Fondant or sugar candy | sugar only |
| 2259 | 2022 | 40.12222 | Yes | Alcohol wash | Yes | Formic Acid (Mite Away Quick Strips or Formic Pro) | Yes | Dry sugar | sugar only |
| 2260 | 2022 | 40.12222 | Yes | Alcohol wash | Yes | Formic Acid (Mite Away Quick Strips or Formic Pro) | Yes | Dry sugar | sugar only |
| 2261 | 2022 | 40.12222 | Yes | Alcohol wash | Yes | Formic Acid (Mite Away Quick Strips or Formic Pro) | Yes | Dry sugar | sugar only |
| 2262 | 2022 | 1.381084 | No | NA | No | none | Yes | Dry sugar | sugar only |
| 2263 | 2022 | 3.729643 | Yes | Drone brood inspection | No | none | Yes | Sugar syrup, Pollen substitute | none |
| 2264 | 2022 | 0.552252 | Yes | Sugar roll | Yes | Formic Acid (Mite Away Quick Strips or Formic Pro) | Yes | Fondant or sugar candy | sugar only |
| 2265 | 2022 | 0.552252 | Yes | Sugar roll | Yes | Formic Acid (Mite Away Quick Strips or Formic Pro) | Yes | Fondant or sugar candy | sugar only |
| 2266 | 2022 | 0.552252 | Yes | Sugar roll | Yes | Formic Acid (Mite Away Quick Strips or Formic Pro) | Yes | Fondant or sugar candy | sugar only |
| 2267 | 2022 | 0.552252 | Yes | Sugar roll | Yes | Formic Acid (Mite Away Quick Strips or Formic Pro) | Yes | Fondant or sugar candy | sugar only |
| 2268 | 2022 | 0.552252 | Yes | Sugar roll | Yes | Formic Acid (Mite Away Quick Strips or Formic Pro) | Yes | Fondant or sugar candy | sugar only |
| 2269 | 2022 | 0.552252 | Yes | Sugar roll | Yes | Formic Acid (Mite Away Quick Strips or Formic Pro) | Yes | Fondant or sugar candy | sugar only |
| 2270 | 2022 | 14.08392 | Yes | 48 hr drop (sticky board) | Yes | Formic Acid (Mite Away Quick Strips or Formic Pro) | Yes | Dry sugar, Pollen substitute | none |
| 2271 | 2022 | 14.08392 | Yes | 48 hr drop (sticky board) | Yes | Formic Acid (Mite Away Quick Strips or Formic Pro) | Yes | Dry sugar, Pollen substitute | none |
| 2272 | 2022 | 37.57264 | No | NA | No | none | No | NA | none |
| 2273 | 2022 | 37.57264 | No | NA | No | none | No | NA | none |
| 2274 | 2022 | 37.57264 | No | NA | No | none | No | NA | none |
| 2275 | 2022 | 37.57264 | No | NA | No | none | No | NA | none |
| 2276 | 2022 | 37.57264 | No | NA | No | none | No | NA | none |
| 2277 | 2022 | 37.57264 | No | NA | No | none | No | NA | none |
| 2278 | 2022 | 37.57264 | No | NA | No | none | No | NA | none |
| 2279 | 2022 | 26.5327 | Yes | Sugar roll | Yes | Oxalic Acid (Vapor) | Yes | Dry sugar | sugar only |
| 2280 | 2022 | 2.649522 | No | NA | Yes | Formic Acid (Mite Away Quick Strips or Formic Pro) | No | NA | none |
| 2281 | 2022 | 2.649522 | No | NA | Yes | Formic Acid (Mite Away Quick Strips or Formic Pro) | No | NA | none |
| 2282 | 2022 | 1.573843 | Yes | Drone brood inspection | No | none | Yes | Fondant or sugar candy | sugar only |
| 2283 | 2022 | 1.573843 | Yes | Drone brood inspection | No | none | Yes | Fondant or sugar candy | sugar only |
| 2284 | 2022 | 1.573843 | Yes | Drone brood inspection | No | none | Yes | Fondant or sugar candy | sugar only |
| 2285 | 2022 | 0.755232 | No | NA | Yes | Apivar (Amitraz) | Yes | Fondant or sugar candy, Honey from your own stock | none |
| 2286 | 2022 | 16.11405 | No | NA | No | none | No | NA | none |
| 2287 | 2022 | 20.825 | No | NA | No | none | No | NA | none |
| 2288 | 2022 | 20.825 | No | NA | No | none | No | NA | none |
| 2289 | 2022 | 0.678509 | No | NA | No | none | Yes | Honey from your own stock, Pollen from your own stock | none |
| 2290 | 2022 | 0.678509 | No | NA | No | none | Yes | Honey from your own stock, Pollen from your own stock | none |
| 2291 | 2022 | 0.678509 | No | NA | No | none | Yes | Honey from your own stock, Pollen from your own stock | none |
| 2292 | 2022 | 4.394436 | Yes | Sugar roll | Yes | Formic Acid (Mite Away Quick Strips or Formic Pro) | No | NA | none |
| 2293 | 2022 | 1.886781 | No | NA | Yes | Formic Acid (Mite Away Quick Strips or Formic Pro) | Yes | Fondant or sugar candy | sugar only |
| 2294 | 2022 | 1.886781 | No | NA | Yes | Formic Acid (Mite Away Quick Strips or Formic Pro) | Yes | Fondant or sugar candy | sugar only |
| 2295 | 2022 | 25.38211 | Yes | 48 hr drop (sticky board) | Yes | Apistan | Yes | Fondant or sugar candy | sugar only |
| 2296 | 2022 | 25.38211 | Yes | 48 hr drop (sticky board) | Yes | Apistan | Yes | Fondant or sugar candy | sugar only |
| 2297 | 2022 | 25.38211 | Yes | 48 hr drop (sticky board) | Yes | Apistan | Yes | Fondant or sugar candy | sugar only |
| 2298 | 2022 | 25.38211 | Yes | 48 hr drop (sticky board) | Yes | Apistan | Yes | Fondant or sugar candy | sugar only |
| 2299 | 2022 | 5.761745 | Yes | Sugar roll | Yes | Formic Acid (Mite Away Quick Strips or Formic Pro) | Yes | Dry sugar | sugar only |
| 2300 | 2022 | 5.761745 | Yes | Sugar roll | Yes | Formic Acid (Mite Away Quick Strips or Formic Pro) | Yes | Dry sugar | sugar only |
| 2301 | 2022 | 5.761745 | Yes | Sugar roll | Yes | Formic Acid (Mite Away Quick Strips or Formic Pro) | Yes | Dry sugar | sugar only |
| 2302 | 2022 | 5.761745 | Yes | Sugar roll | Yes | Formic Acid (Mite Away Quick Strips or Formic Pro) | Yes | Dry sugar | sugar only |
| 2303 | 2022 | 5.761745 | Yes | Sugar roll | Yes | Formic Acid (Mite Away Quick Strips or Formic Pro) | Yes | Dry sugar | sugar only |
| 2304 | 2022 | 1.590759 | Yes | 48 hr drop (sticky board), Sugar roll | Yes | Api life Var | Yes | Fondant or sugar candy | sugar only |
| 2305 | 2022 | 1.590759 | Yes | 48 hr drop (sticky board), Sugar roll | Yes | Api life Var | Yes | Fondant or sugar candy | sugar only |
| 2306 | 2022 | 1.590759 | Yes | 48 hr drop (sticky board), Sugar roll | Yes | Api life Var | Yes | Fondant or sugar candy | sugar only |
| 2307 | 2022 | 1.364367 | No | NA | Yes | Apivar (Amitraz) | No | NA | none |
| 2308 | 2022 | 1.364367 | No | NA | Yes | Apivar (Amitraz) | No | NA | none |
| 2309 | 2022 | 4.238627 | Yes | Alcohol wash | Yes | Formic Acid (Mite Away Quick Strips or Formic Pro) | Yes | Dry sugar | sugar only |
| 2310 | 2022 | 4.238627 | Yes | Alcohol wash | Yes | Formic Acid (Mite Away Quick Strips or Formic Pro) | Yes | Dry sugar | sugar only |
| 2311 | 2022 | 4.238627 | Yes | Alcohol wash | Yes | Formic Acid (Mite Away Quick Strips or Formic Pro) | Yes | Dry sugar | sugar only |
| 2312 | 2022 | 11.1666 | Yes | Alcohol wash | Yes | Formic Acid (Mite Away Quick Strips or Formic Pro) | Yes | Pollen substitute | pollen only |
| 2313 | 2022 | 4.515524 | Yes | Alcohol wash | No | none | Yes | Dry sugar | sugar only |
| 2314 | 2022 | 4.515524 | Yes | Alcohol wash | No | none | Yes | Dry sugar | sugar only |
| 2315 | 2022 | 4.515524 | Yes | Alcohol wash | No | none | Yes | Dry sugar | sugar only |
| 2316 | 2022 | 4.515524 | Yes | Alcohol wash | No | none | Yes | Dry sugar | sugar only |
| 2317 | 2022 | 0.622149 | Yes | Sugar roll, Drone brood inspection | Yes | Oxalic Acid (Vapor) | Yes | Fondant or sugar candy | sugar only |
| 2318 | 2022 | 0.622149 | Yes | Sugar roll, Drone brood inspection | Yes | Oxalic Acid (Vapor) | Yes | Fondant or sugar candy | sugar only |
| 2319 | 2022 | 0.622149 | Yes | Sugar roll, Drone brood inspection | Yes | Oxalic Acid (Vapor) | Yes | Fondant or sugar candy | sugar only |
| 2320 | 2022 | 0.622149 | Yes | Sugar roll, Drone brood inspection | Yes | Oxalic Acid (Vapor) | Yes | Fondant or sugar candy | sugar only |
| 2321 | 2022 | 0.87332 | Yes | Alcohol wash | Yes | Formic Acid (Mite Away Quick Strips or Formic Pro) | Yes | Fondant or sugar candy, Pollen substitute | none |
| 2322 | 2022 | 0.87332 | Yes | Alcohol wash | Yes | Formic Acid (Mite Away Quick Strips or Formic Pro) | Yes | Fondant or sugar candy, Pollen substitute | none |
| 2323 | 2022 | 0.87332 | Yes | Alcohol wash | Yes | Formic Acid (Mite Away Quick Strips or Formic Pro) | Yes | Fondant or sugar candy, Pollen substitute | none |
| 2324 | 2022 | 0.87332 | Yes | Alcohol wash | Yes | Formic Acid (Mite Away Quick Strips or Formic Pro) | Yes | Fondant or sugar candy, Pollen substitute | none |
| 2325 | 2022 | 0.87332 | Yes | Alcohol wash | Yes | Formic Acid (Mite Away Quick Strips or Formic Pro) | Yes | Fondant or sugar candy, Pollen substitute | none |
| 2326 | 2022 | 0.87332 | Yes | Alcohol wash | Yes | Formic Acid (Mite Away Quick Strips or Formic Pro) | Yes | Fondant or sugar candy, Pollen substitute | none |
| 2327 | 2022 | 0.87332 | Yes | Alcohol wash | Yes | Formic Acid (Mite Away Quick Strips or Formic Pro) | Yes | Fondant or sugar candy, Pollen substitute | none |
| 2328 | 2022 | 0.87332 | Yes | Alcohol wash | Yes | Formic Acid (Mite Away Quick Strips or Formic Pro) | Yes | Fondant or sugar candy, Pollen substitute | none |
| 2329 | 2022 | 0.87332 | Yes | Alcohol wash | Yes | Formic Acid (Mite Away Quick Strips or Formic Pro) | Yes | Fondant or sugar candy, Pollen substitute | none |
| 2330 | 2022 | 0.87332 | Yes | Alcohol wash | Yes | Formic Acid (Mite Away Quick Strips or Formic Pro) | Yes | Fondant or sugar candy, Pollen substitute | none |
| 2331 | 2022 | 0.87332 | Yes | Alcohol wash | Yes | Formic Acid (Mite Away Quick Strips or Formic Pro) | Yes | Fondant or sugar candy, Pollen substitute | none |
| 2332 | 2022 | 0.87332 | Yes | Alcohol wash | Yes | Formic Acid (Mite Away Quick Strips or Formic Pro) | Yes | Fondant or sugar candy, Pollen substitute | none |
| 2333 | 2022 | 0.87332 | Yes | Alcohol wash | Yes | Formic Acid (Mite Away Quick Strips or Formic Pro) | Yes | Fondant or sugar candy, Pollen substitute | none |
| 2334 | 2022 | 0.87332 | Yes | Alcohol wash | Yes | Formic Acid (Mite Away Quick Strips or Formic Pro) | Yes | Fondant or sugar candy, Pollen substitute | none |
| 2335 | 2022 | 0.87332 | Yes | Alcohol wash | Yes | Formic Acid (Mite Away Quick Strips or Formic Pro) | Yes | Fondant or sugar candy, Pollen substitute | none |
| 2336 | 2022 | 0.87332 | Yes | Alcohol wash | Yes | Formic Acid (Mite Away Quick Strips or Formic Pro) | Yes | Fondant or sugar candy, Pollen substitute | none |
| 2337 | 2022 | 0.87332 | Yes | Alcohol wash | Yes | Formic Acid (Mite Away Quick Strips or Formic Pro) | Yes | Fondant or sugar candy, Pollen substitute | none |
| 2338 | 2022 | 0.87332 | Yes | Alcohol wash | Yes | Formic Acid (Mite Away Quick Strips or Formic Pro) | Yes | Fondant or sugar candy, Pollen substitute | none |
| 2339 | 2022 | 0.87332 | Yes | Alcohol wash | Yes | Formic Acid (Mite Away Quick Strips or Formic Pro) | Yes | Fondant or sugar candy, Pollen substitute | none |
| 2340 | 2022 | 0.87332 | Yes | Alcohol wash | Yes | Formic Acid (Mite Away Quick Strips or Formic Pro) | Yes | Fondant or sugar candy, Pollen substitute | none |
| 2341 | 2022 | 0.87332 | Yes | Alcohol wash | Yes | Formic Acid (Mite Away Quick Strips or Formic Pro) | Yes | Fondant or sugar candy, Pollen substitute | none |
| 2342 | 2022 | 0.87332 | Yes | Alcohol wash | Yes | Formic Acid (Mite Away Quick Strips or Formic Pro) | Yes | Fondant or sugar candy, Pollen substitute | none |
| 2343 | 2022 | 0.87332 | Yes | Alcohol wash | Yes | Formic Acid (Mite Away Quick Strips or Formic Pro) | Yes | Fondant or sugar candy, Pollen substitute | none |
| 2344 | 2022 | 0.87332 | Yes | Alcohol wash | Yes | Formic Acid (Mite Away Quick Strips or Formic Pro) | Yes | Fondant or sugar candy, Pollen substitute | none |
| 2345 | 2022 | 0.87332 | Yes | Alcohol wash | Yes | Formic Acid (Mite Away Quick Strips or Formic Pro) | Yes | Fondant or sugar candy, Pollen substitute | none |
| 2346 | 2022 | 0.87332 | Yes | Alcohol wash | Yes | Formic Acid (Mite Away Quick Strips or Formic Pro) | Yes | Fondant or sugar candy, Pollen substitute | none |
| 2347 | 2022 | 0.87332 | Yes | Alcohol wash | Yes | Formic Acid (Mite Away Quick Strips or Formic Pro) | Yes | Fondant or sugar candy, Pollen substitute | none |
| 2348 | 2022 | 0.87332 | Yes | Alcohol wash | Yes | Formic Acid (Mite Away Quick Strips or Formic Pro) | Yes | Fondant or sugar candy, Pollen substitute | none |
| 2349 | 2022 | 0.87332 | Yes | Alcohol wash | Yes | Formic Acid (Mite Away Quick Strips or Formic Pro) | Yes | Fondant or sugar candy, Pollen substitute | none |
| 2350 | 2022 | 0.87332 | Yes | Alcohol wash | Yes | Formic Acid (Mite Away Quick Strips or Formic Pro) | Yes | Fondant or sugar candy, Pollen substitute | none |
| 2351 | 2022 | 0.87332 | Yes | Alcohol wash | Yes | Formic Acid (Mite Away Quick Strips or Formic Pro) | Yes | Fondant or sugar candy, Pollen substitute | none |
| 2352 | 2022 | 0.87332 | Yes | Alcohol wash | Yes | Formic Acid (Mite Away Quick Strips or Formic Pro) | Yes | Fondant or sugar candy, Pollen substitute | none |
| 2353 | 2022 | 0.87332 | Yes | Alcohol wash | Yes | Formic Acid (Mite Away Quick Strips or Formic Pro) | Yes | Fondant or sugar candy, Pollen substitute | none |
| 2354 | 2022 | 0.87332 | Yes | Alcohol wash | Yes | Formic Acid (Mite Away Quick Strips or Formic Pro) | Yes | Fondant or sugar candy, Pollen substitute | none |
| 2355 | 2022 | 0.87332 | Yes | Alcohol wash | Yes | Formic Acid (Mite Away Quick Strips or Formic Pro) | Yes | Fondant or sugar candy, Pollen substitute | none |
| 2356 | 2022 | 0.87332 | Yes | Alcohol wash | Yes | Formic Acid (Mite Away Quick Strips or Formic Pro) | Yes | Fondant or sugar candy, Pollen substitute | none |
| 2357 | 2022 | 0.87332 | Yes | Alcohol wash | Yes | Formic Acid (Mite Away Quick Strips or Formic Pro) | Yes | Fondant or sugar candy, Pollen substitute | none |
| 2358 | 2022 | 0.87332 | Yes | Alcohol wash | Yes | Formic Acid (Mite Away Quick Strips or Formic Pro) | Yes | Fondant or sugar candy, Pollen substitute | none |
| 2359 | 2022 | 0.87332 | Yes | Alcohol wash | Yes | Formic Acid (Mite Away Quick Strips or Formic Pro) | Yes | Fondant or sugar candy, Pollen substitute | none |
| 2360 | 2022 | 0.87332 | Yes | Alcohol wash | Yes | Formic Acid (Mite Away Quick Strips or Formic Pro) | Yes | Fondant or sugar candy, Pollen substitute | none |
| 2361 | 2022 | 0.87332 | Yes | Alcohol wash | Yes | Formic Acid (Mite Away Quick Strips or Formic Pro) | Yes | Fondant or sugar candy, Pollen substitute | none |
| 2362 | 2022 | 0.87332 | Yes | Alcohol wash | Yes | Formic Acid (Mite Away Quick Strips or Formic Pro) | Yes | Fondant or sugar candy, Pollen substitute | none |
| 2363 | 2022 | 0.87332 | Yes | Alcohol wash | Yes | Formic Acid (Mite Away Quick Strips or Formic Pro) | Yes | Fondant or sugar candy, Pollen substitute | none |
| 2364 | 2022 | 0.87332 | Yes | Alcohol wash | Yes | Formic Acid (Mite Away Quick Strips or Formic Pro) | Yes | Fondant or sugar candy, Pollen substitute | none |
| 2365 | 2022 | 0.87332 | Yes | Alcohol wash | Yes | Formic Acid (Mite Away Quick Strips or Formic Pro) | Yes | Fondant or sugar candy, Pollen substitute | none |
| 2366 | 2022 | 0.87332 | Yes | Alcohol wash | Yes | Formic Acid (Mite Away Quick Strips or Formic Pro) | Yes | Fondant or sugar candy, Pollen substitute | none |
| 2367 | 2022 | 0.87332 | Yes | Alcohol wash | Yes | Formic Acid (Mite Away Quick Strips or Formic Pro) | Yes | Fondant or sugar candy, Pollen substitute | none |
| 2368 | 2022 | 0.87332 | Yes | Alcohol wash | Yes | Formic Acid (Mite Away Quick Strips or Formic Pro) | Yes | Fondant or sugar candy, Pollen substitute | none |
| 2369 | 2022 | 0.87332 | Yes | Alcohol wash | Yes | Formic Acid (Mite Away Quick Strips or Formic Pro) | Yes | Fondant or sugar candy, Pollen substitute | none |
| 2370 | 2022 | 0.87332 | Yes | Alcohol wash | Yes | Formic Acid (Mite Away Quick Strips or Formic Pro) | Yes | Fondant or sugar candy, Pollen substitute | none |
| 2371 | 2022 | 0.87332 | Yes | Alcohol wash | Yes | Formic Acid (Mite Away Quick Strips or Formic Pro) | Yes | Fondant or sugar candy, Pollen substitute | none |
| 2372 | 2022 | 0.87332 | Yes | Alcohol wash | Yes | Formic Acid (Mite Away Quick Strips or Formic Pro) | Yes | Fondant or sugar candy, Pollen substitute | none |
| 2373 | 2022 | 0.87332 | Yes | Alcohol wash | Yes | Formic Acid (Mite Away Quick Strips or Formic Pro) | Yes | Fondant or sugar candy, Pollen substitute | none |
| 2374 | 2022 | 0.87332 | Yes | Alcohol wash | Yes | Formic Acid (Mite Away Quick Strips or Formic Pro) | Yes | Fondant or sugar candy, Pollen substitute | none |
| 2375 | 2022 | 0.87332 | Yes | Alcohol wash | Yes | Formic Acid (Mite Away Quick Strips or Formic Pro) | Yes | Fondant or sugar candy, Pollen substitute | none |
| 2376 | 2022 | 0.87332 | Yes | Alcohol wash | Yes | Formic Acid (Mite Away Quick Strips or Formic Pro) | Yes | Fondant or sugar candy, Pollen substitute | none |
| 2377 | 2022 | 0.87332 | Yes | Alcohol wash | Yes | Formic Acid (Mite Away Quick Strips or Formic Pro) | Yes | Fondant or sugar candy, Pollen substitute | none |
| 2378 | 2022 | 0.87332 | Yes | Alcohol wash | Yes | Formic Acid (Mite Away Quick Strips or Formic Pro) | Yes | Fondant or sugar candy, Pollen substitute | none |
| 2379 | 2022 | 0.87332 | Yes | Alcohol wash | Yes | Formic Acid (Mite Away Quick Strips or Formic Pro) | Yes | Fondant or sugar candy, Pollen substitute | none |
| 2380 | 2022 | 0.87332 | Yes | Alcohol wash | Yes | Formic Acid (Mite Away Quick Strips or Formic Pro) | Yes | Fondant or sugar candy, Pollen substitute | none |
| 2381 | 2022 | 26.8095 | No | NA | Yes | Formic Acid (Mite Away Quick Strips or Formic Pro) | Yes | Fondant or sugar candy, Commercially available supplements | none |
| 2382 | 2022 | 26.8095 | No | NA | Yes | Formic Acid (Mite Away Quick Strips or Formic Pro) | Yes | Fondant or sugar candy, Commercially available supplements | none |
| 2383 | 2022 | 26.8095 | No | NA | Yes | Formic Acid (Mite Away Quick Strips or Formic Pro) | Yes | Fondant or sugar candy, Commercially available supplements | none |
| 2384 | 2022 | 6.723522 | Yes | Alcohol wash | Yes | Formic Acid (Mite Away Quick Strips or Formic Pro) | Yes | Fondant or sugar candy, Honey from your own stock | none |
| 2385 | 2022 | 6.723522 | Yes | Alcohol wash | Yes | Formic Acid (Mite Away Quick Strips or Formic Pro) | Yes | Fondant or sugar candy, Honey from your own stock | none |
| 2386 | 2022 | 46.36648 | No | NA | Yes | Other | Yes | Sugar syrup | none |
| 2387 | 2022 | 46.36648 | No | NA | Yes | Other | Yes | Sugar syrup | none |
| 2388 | 2022 | 46.36648 | No | NA | Yes | Other | Yes | Sugar syrup | none |
| 2389 | 2022 | 46.36648 | No | NA | Yes | Other | Yes | Sugar syrup | none |
| 2390 | 2022 | 46.36648 | No | NA | Yes | Other | Yes | Sugar syrup | none |
| 2391 | 2022 | 46.36648 | No | NA | Yes | Other | Yes | Sugar syrup | none |
| 2392 | 2022 | 46.36648 | No | NA | Yes | Other | Yes | Sugar syrup | none |
| 2393 | 2022 | 46.36648 | No | NA | Yes | Other | Yes | Sugar syrup | none |
| 2394 | 2022 | 46.36648 | No | NA | Yes | Other | Yes | Sugar syrup | none |
| 2395 | 2022 | 46.36648 | No | NA | Yes | Other | Yes | Sugar syrup | none |
| 2396 | 2022 | 46.36648 | No | NA | Yes | Other | Yes | Sugar syrup | none |
| 2397 | 2022 | 46.36648 | No | NA | Yes | Other | Yes | Sugar syrup | none |
| 2398 | 2022 | 46.36648 | No | NA | Yes | Other | Yes | Sugar syrup | none |
| 2399 | 2022 | 46.36648 | No | NA | Yes | Other | Yes | Sugar syrup | none |
| 2400 | 2022 | 46.36648 | No | NA | Yes | Other | Yes | Sugar syrup | none |
| 2401 | 2022 | 46.36648 | No | NA | Yes | Other | Yes | Sugar syrup | none |
| 2402 | 2022 | 5.136822 | Yes | 48 hr drop (sticky board) | Yes | Formic Acid (Mite Away Quick Strips or Formic Pro) | Yes | Fondant or sugar candy, Pollen substitute | none |
| 2403 | 2022 | 5.136822 | Yes | 48 hr drop (sticky board) | Yes | Formic Acid (Mite Away Quick Strips or Formic Pro) | Yes | Fondant or sugar candy, Pollen substitute | none |
| 2404 | 2022 | 5.136822 | Yes | 48 hr drop (sticky board) | Yes | Formic Acid (Mite Away Quick Strips or Formic Pro) | Yes | Fondant or sugar candy, Pollen substitute | none |
| 2405 | 2022 | 67.97092 | Yes | Alcohol wash | Yes | Formic Acid (Mite Away Quick Strips or Formic Pro) | Yes | Dry sugar | sugar only |
| 2406 | 2022 | 67.97092 | Yes | Alcohol wash | Yes | Formic Acid (Mite Away Quick Strips or Formic Pro) | Yes | Dry sugar | sugar only |
| 2407 | 2022 | 67.97092 | Yes | Alcohol wash | Yes | Formic Acid (Mite Away Quick Strips or Formic Pro) | Yes | Dry sugar | sugar only |
| 2408 | 2022 | 67.97092 | Yes | Alcohol wash | Yes | Formic Acid (Mite Away Quick Strips or Formic Pro) | Yes | Dry sugar | sugar only |
| 2409 | 2022 | 8.978805 | Yes | 48 hr drop (sticky board) | Yes | Apistan | Yes | Fondant or sugar candy, Pollen substitute | none |
| 2410 | 2022 | 8.978805 | Yes | 48 hr drop (sticky board) | Yes | Apistan | Yes | Fondant or sugar candy, Pollen substitute | none |
| 2411 | 2022 | 5.614225 | Yes | 48 hr drop (sticky board) | Yes | Formic Acid (Mite Away Quick Strips or Formic Pro) | No | NA | none |
| 2412 | 2022 | 13.87531 | Yes | 48 hr drop (sticky board), Drone brood inspection | Yes | Hopguard | No | NA | none |
| 2413 | 2022 | 13.87531 | Yes | 48 hr drop (sticky board), Drone brood inspection | Yes | Hopguard | No | NA | none |
| 2414 | 2022 | 13.87531 | Yes | 48 hr drop (sticky board), Drone brood inspection | Yes | Hopguard | No | NA | none |
| 2415 | 2022 | 13.87531 | Yes | 48 hr drop (sticky board), Drone brood inspection | Yes | Hopguard | No | NA | none |
| 2416 | 2022 | 13.87531 | Yes | 48 hr drop (sticky board), Drone brood inspection | Yes | Hopguard | No | NA | none |
| 2417 | 2022 | 13.87531 | Yes | 48 hr drop (sticky board), Drone brood inspection | Yes | Hopguard | No | NA | none |
| 2418 | 2022 | 13.87531 | Yes | 48 hr drop (sticky board), Drone brood inspection | Yes | Hopguard | No | NA | none |
| 2419 | 2022 | 13.87531 | Yes | 48 hr drop (sticky board), Drone brood inspection | Yes | Hopguard | No | NA | none |
| 2420 | 2022 | 13.87531 | Yes | 48 hr drop (sticky board), Drone brood inspection | Yes | Hopguard | No | NA | none |
| 2421 | 2022 | 13.87531 | Yes | 48 hr drop (sticky board), Drone brood inspection | Yes | Hopguard | No | NA | none |
| 2422 | 2022 | 13.87531 | Yes | 48 hr drop (sticky board), Drone brood inspection | Yes | Hopguard | No | NA | none |
| 2423 | 2022 | 13.87531 | Yes | 48 hr drop (sticky board), Drone brood inspection | Yes | Hopguard | No | NA | none |
| 2424 | 2022 | 13.87531 | Yes | 48 hr drop (sticky board), Drone brood inspection | Yes | Hopguard | No | NA | none |
| 2425 | 2022 | 13.87531 | Yes | 48 hr drop (sticky board), Drone brood inspection | Yes | Hopguard | No | NA | none |
| 2426 | 2022 | 27.97152 | Yes | Alcohol wash | Yes | Oxalic Acid (Dribble) | No | NA | none |
| 2427 | 2022 | 48.38647 | No | NA | Yes | Other | No | NA | none |
| 2428 | 2022 | 48.38647 | No | NA | Yes | Other | No | NA | none |
| 2429 | 2022 | 48.38647 | No | NA | Yes | Other | No | NA | none |
| 2430 | 2022 | 48.38647 | No | NA | Yes | Other | No | NA | none |
| 2431 | 2022 | 6.158959 | Yes | Sugar roll | Yes | Api life Var | Yes | Dry sugar | sugar only |
| 2432 | 2022 | 0.843282 | Yes | Sugar roll | Yes | Oxalic Acid (Dribble) | Yes | Honey from your own stock | none |
| 2433 | 2022 | 4.802203 | No | NA | No | none | Yes | Other | none |
| 2434 | 2022 | 4.802203 | No | NA | No | none | Yes | Other | none |
| 2435 | 2022 | 12.73371 | Yes | Drone brood inspection | No | none | Yes | Honey from your own stock | none |
| 2436 | 2022 | 12.73371 | Yes | Drone brood inspection | No | none | Yes | Honey from your own stock | none |
| 2437 | 2022 | 8.519914 | No | NA | Yes | Oxalic Acid (Vapor) | No | NA | none |
| 2438 | 2022 | 8.519914 | No | NA | Yes | Oxalic Acid (Vapor) | No | NA | none |
| 2439 | 2022 | 8.519914 | No | NA | Yes | Oxalic Acid (Vapor) | No | NA | none |
| 2440 | 2022 | 2.349556 | Yes | Drone brood inspection | Yes | Apivar (Amitraz) | Yes | Dry sugar, Honey from your own stock | none |
| 2441 | 2022 | 2.349556 | Yes | Drone brood inspection | Yes | Apivar (Amitraz) | Yes | Dry sugar, Honey from your own stock | none |
| 2442 | 2022 | 32.77322 | No | NA | No | none | Yes | Fondant or sugar candy | sugar only |
| 2443 | 2022 | 2.582486 | Yes | 48 hr drop (sticky board), Sugar roll | Yes | Formic Acid (Mite Away Quick Strips or Formic Pro) | Yes | Dry sugar | sugar only |
| 2444 | 2022 | 2.582486 | Yes | 48 hr drop (sticky board), Sugar roll | Yes | Formic Acid (Mite Away Quick Strips or Formic Pro) | Yes | Dry sugar | sugar only |
| 2445 | 2022 | 17.66475 | No | NA | Yes | Oxalic Acid (Vapor) | Yes | Fondant or sugar candy, Pollen substitute | none |
| 2446 | 2022 | 2.104875 | No | NA | No | none | No | NA | none |
| 2447 | 2022 | 2.104875 | No | NA | No | none | No | NA | none |
| 2448 | 2022 | 2.104875 | No | NA | No | none | No | NA | none |
| 2449 | 2022 | 2.104875 | No | NA | No | none | No | NA | none |
| 2450 | 2022 | 1.942503 | Yes | Sugar roll | Yes | Apivar (Amitraz) | Yes | Fondant or sugar candy, Dry sugar | none |
| 2451 | 2022 | 1.942503 | Yes | Sugar roll | Yes | Apivar (Amitraz) | Yes | Fondant or sugar candy, Dry sugar | none |
| 2452 | 2022 | 1.942503 | Yes | Sugar roll | Yes | Apivar (Amitraz) | Yes | Fondant or sugar candy, Dry sugar | none |
| 2453 | 2022 | 1.942503 | Yes | Sugar roll | Yes | Apivar (Amitraz) | Yes | Fondant or sugar candy, Dry sugar | none |
| 2454 | 2022 | 3.38816 | Yes | 48 hr drop (sticky board) | No | none | Yes | Sugar syrup | none |
| 2455 | 2022 | 2.523737 | Yes | Alcohol wash | Yes | Apivar (Amitraz) | No | NA | none |
| 2456 | 2022 | 2.523737 | Yes | Alcohol wash | Yes | Apivar (Amitraz) | No | NA | none |
| 2457 | 2022 | 2.523737 | Yes | Alcohol wash | Yes | Apivar (Amitraz) | No | NA | none |
| 2458 | 2022 | 14.72785 | Yes | Alcohol wash | Yes | Oxalic Acid (Vapor) | Yes | Fondant or sugar candy, Dry sugar | none |
| 2459 | 2022 | 14.72785 | Yes | Alcohol wash | Yes | Oxalic Acid (Vapor) | Yes | Fondant or sugar candy, Dry sugar | none |
| 2460 | 2022 | 14.72785 | Yes | Alcohol wash | Yes | Oxalic Acid (Vapor) | Yes | Fondant or sugar candy, Dry sugar | none |
| 2461 | 2022 | 14.72785 | Yes | Alcohol wash | Yes | Oxalic Acid (Vapor) | Yes | Fondant or sugar candy, Dry sugar | none |
| 2462 | 2022 | 14.72785 | Yes | Alcohol wash | Yes | Oxalic Acid (Vapor) | Yes | Fondant or sugar candy, Dry sugar | none |
| 2463 | 2022 | 9.74853 | Yes | 48 hr drop (sticky board), Drone brood inspection | Yes | Api life Var | Yes | Fondant or sugar candy | sugar only |
| 2464 | 2022 | 9.74853 | Yes | 48 hr drop (sticky board), Drone brood inspection | Yes | Api life Var | Yes | Fondant or sugar candy | sugar only |
| 2465 | 2022 | 9.74853 | Yes | 48 hr drop (sticky board), Drone brood inspection | Yes | Api life Var | Yes | Fondant or sugar candy | sugar only |
| 2466 | 2022 | 9.74853 | Yes | 48 hr drop (sticky board), Drone brood inspection | Yes | Api life Var | Yes | Fondant or sugar candy | sugar only |
| 2467 | 2022 | 12.64823 | Yes | Sugar roll | Yes | Oxalic Acid (Vapor) | Yes | Fondant or sugar candy | sugar only |
| 2468 | 2022 | 12.64823 | Yes | Sugar roll | Yes | Oxalic Acid (Vapor) | Yes | Fondant or sugar candy | sugar only |
| 2469 | 2022 | 3.158607 | Yes | Sugar roll | No | none | No | NA | none |
| 2470 | 2022 | 3.158607 | Yes | Sugar roll | No | none | No | NA | none |
| 2471 | 2022 | 16.87403 | Yes | Alcohol wash | Yes | Oxalic Acid (Vapor) | Yes | Sugar syrup | none |
| 2472 | 2022 | 16.87403 | Yes | Alcohol wash | Yes | Oxalic Acid (Vapor) | Yes | Sugar syrup | none |
| 2473 | 2022 | 16.87403 | Yes | Alcohol wash | Yes | Oxalic Acid (Vapor) | Yes | Sugar syrup | none |
| 2474 | 2022 | 16.87403 | Yes | Alcohol wash | Yes | Oxalic Acid (Vapor) | Yes | Sugar syrup | none |
| 2475 | 2022 | 16.87403 | Yes | Alcohol wash | Yes | Oxalic Acid (Vapor) | Yes | Sugar syrup | none |
| 2476 | 2022 | 27.3311 | Yes | 48 hr drop (sticky board), Drone brood inspection | No | none | No | NA | none |
| 2477 | 2022 | 27.3311 | Yes | 48 hr drop (sticky board), Drone brood inspection | No | none | No | NA | none |
| 2478 | 2022 | 27.3311 | Yes | 48 hr drop (sticky board), Drone brood inspection | No | none | No | NA | none |
| 2479 | 2022 | 27.3311 | Yes | 48 hr drop (sticky board), Drone brood inspection | No | none | No | NA | none |
| 2480 | 2022 | 27.3311 | Yes | 48 hr drop (sticky board), Drone brood inspection | No | none | No | NA | none |
| 2481 | 2022 | 27.3311 | Yes | 48 hr drop (sticky board), Drone brood inspection | No | none | No | NA | none |
| 2482 | 2022 | 27.3311 | Yes | 48 hr drop (sticky board), Drone brood inspection | No | none | No | NA | none |
| 2483 | 2022 | 27.3311 | Yes | 48 hr drop (sticky board), Drone brood inspection | No | none | No | NA | none |
| 2484 | 2022 | 3.650761 | Yes | 48 hr drop (sticky board), Sugar roll | Yes | Formic Acid (Mite Away Quick Strips or Formic Pro) | Yes | Pollen substitute, Honey from your own stock | none |
| 2485 | 2022 | 3.650761 | Yes | 48 hr drop (sticky board), Sugar roll | Yes | Formic Acid (Mite Away Quick Strips or Formic Pro) | Yes | Pollen substitute, Honey from your own stock | none |
| 2486 | 2022 | 3.650761 | Yes | 48 hr drop (sticky board), Sugar roll | Yes | Formic Acid (Mite Away Quick Strips or Formic Pro) | Yes | Pollen substitute, Honey from your own stock | none |
| 2487 | 2022 | 7.903955 | Yes | Sugar roll | Yes | Oxalic Acid (Vapor) | Yes | Fondant or sugar candy | sugar only |
| 2488 | 2022 | 7.903955 | Yes | Sugar roll | Yes | Oxalic Acid (Vapor) | Yes | Fondant or sugar candy | sugar only |
| 2489 | 2022 | 7.903955 | Yes | Sugar roll | Yes | Oxalic Acid (Vapor) | Yes | Fondant or sugar candy | sugar only |
| 2490 | 2022 | 7.903955 | Yes | Sugar roll | Yes | Oxalic Acid (Vapor) | Yes | Fondant or sugar candy | sugar only |
| 2491 | 2022 | 4.783526 | No | NA | Yes | Oxalic Acid (Vapor) | Yes | Fondant or sugar candy | sugar only |
| 2492 | 2022 | 4.783526 | No | NA | Yes | Oxalic Acid (Vapor) | Yes | Fondant or sugar candy | sugar only |
| 2493 | 2022 | 4.783526 | No | NA | Yes | Oxalic Acid (Vapor) | Yes | Fondant or sugar candy | sugar only |
| 2494 | 2022 | 4.783526 | No | NA | Yes | Oxalic Acid (Vapor) | Yes | Fondant or sugar candy | sugar only |
| 2495 | 2022 | 4.783526 | No | NA | Yes | Oxalic Acid (Vapor) | Yes | Fondant or sugar candy | sugar only |
| 2496 | 2022 | 11.79289 | No | NA | Yes | Formic Acid (Mite Away Quick Strips or Formic Pro) | No | NA | none |
| 2497 | 2022 | 11.79289 | No | NA | Yes | Formic Acid (Mite Away Quick Strips or Formic Pro) | No | NA | none |
| 2498 | 2022 | 11.79289 | No | NA | Yes | Formic Acid (Mite Away Quick Strips or Formic Pro) | No | NA | none |
| 2499 | 2022 | 11.79289 | No | NA | Yes | Formic Acid (Mite Away Quick Strips or Formic Pro) | No | NA | none |
| 2500 | 2022 | 11.79289 | No | NA | Yes | Formic Acid (Mite Away Quick Strips or Formic Pro) | No | NA | none |
|  |  |  |  |  |  |  |  |  |  |
| 2501 | 2022 | 11.79289 | No | NA | Yes | Formic Acid (Mite Away Quick Strips or Formic Pro) | No | NA | none |
| 2502 | 2022 | 1.252488 | No | 48 hr drop (sticky board) | Yes | Formic Acid (Mite Away Quick Strips or Formic Pro) | Yes | Honey from your own stock | none |
| 2503 | 2022 | 1.252488 | No | 48 hr drop (sticky board) | Yes | Formic Acid (Mite Away Quick Strips or Formic Pro) | Yes | Honey from your own stock | none |
| 2504 | 2022 | 25.86209 | Yes | Other | Yes | Formic Acid (Mite Away Quick Strips or Formic Pro) | Yes | Commercially available supplements | none |
| 2505 | 2022 | 25.86209 | Yes | Other | Yes | Formic Acid (Mite Away Quick Strips or Formic Pro) | Yes | Commercially available supplements | none |
| 2506 | 2022 | 8.741833 | Yes | Drone brood inspection | Yes | Formic Acid (Mite Away Quick Strips or Formic Pro) | Yes | Dry sugar | sugar only |
| 2507 | 2022 | 8.741833 | Yes | Drone brood inspection | Yes | Formic Acid (Mite Away Quick Strips or Formic Pro) | Yes | Dry sugar | sugar only |
| 2508 | 2022 | 8.741833 | Yes | Drone brood inspection | Yes | Formic Acid (Mite Away Quick Strips or Formic Pro) | Yes | Dry sugar | sugar only |
| 2509 | 2022 | 6.130395 | Yes | 48 hr drop (sticky board), Drone brood inspection | Yes | Oxalic Acid (Vapor) | Yes | Fondant or sugar candy | sugar only |
| 2510 | 2022 | 6.130395 | Yes | 48 hr drop (sticky board), Drone brood inspection | Yes | Oxalic Acid (Vapor) | Yes | Fondant or sugar candy | sugar only |
| 2511 | 2022 | 6.130395 | Yes | 48 hr drop (sticky board), Drone brood inspection | Yes | Oxalic Acid (Vapor) | Yes | Fondant or sugar candy | sugar only |
| 2512 | 2022 | 6.130395 | Yes | 48 hr drop (sticky board), Drone brood inspection | Yes | Oxalic Acid (Vapor) | Yes | Fondant or sugar candy | sugar only |
| 2513 | 2022 | 5.707164 | Yes | Other | Yes | Oxalic Acid (Vapor) | Yes | Fondant or sugar candy | sugar only |
| 2514 | 2022 | 5.707164 | Yes | Other | Yes | Oxalic Acid (Vapor) | Yes | Fondant or sugar candy | sugar only |
| 2515 | 2022 | 1.761004 | Yes | Sugar roll, Drone brood inspection | Yes | Oxalic Acid (Vapor) | Yes | Fondant or sugar candy | sugar only |
| 2516 | 2022 | 11.23274 | Yes | Alcohol wash | Yes | Formic Acid (Mite Away Quick Strips or Formic Pro) | Yes | Probiotics | none |
| 2517 | 2022 | 14.70713 | Yes | Sugar roll | Yes | Api life Var | Yes | Fondant or sugar candy | sugar only |
| 2518 | 2022 | 14.70713 | Yes | Sugar roll | Yes | Api life Var | Yes | Fondant or sugar candy | sugar only |
| 2519 | 2022 | 14.70713 | Yes | Sugar roll | Yes | Api life Var | Yes | Fondant or sugar candy | sugar only |
| 2520 | 2022 | 14.70713 | Yes | Sugar roll | Yes | Api life Var | Yes | Fondant or sugar candy | sugar only |
| 2521 | 2022 | 14.70713 | Yes | Sugar roll | Yes | Api life Var | Yes | Fondant or sugar candy | sugar only |
| 2522 | 2022 | 14.70713 | Yes | Sugar roll | Yes | Api life Var | Yes | Fondant or sugar candy | sugar only |
| 2523 | 2022 | 27.45161 | No | NA | No | none | Yes | Fondant or sugar candy, Sugar syrup | none |
| 2524 | 2022 | 39.20395 | No | NA | Yes | Formic Acid (Mite Away Quick Strips or Formic Pro) | No | NA | none |
| 2525 | 2022 | 39.20395 | No | NA | Yes | Formic Acid (Mite Away Quick Strips or Formic Pro) | No | NA | none |
| 2526 | 2022 | 7.600376 | Yes | Sugar roll, Alcohol wash, Drone brood inspection | Yes | Formic Acid (Mite Away Quick Strips or Formic Pro) | No | NA | none |
| 2527 | 2022 | 7.600376 | Yes | Sugar roll, Alcohol wash, Drone brood inspection | Yes | Formic Acid (Mite Away Quick Strips or Formic Pro) | No | NA | none |
| 2528 | 2022 | 0.301202 | No | NA | No | none | No | NA | none |
| 2529 | 2022 | 0.301202 | No | NA | No | none | No | NA | none |
| 2530 | 2022 | 2.903958 | Yes | Alcohol wash, Drone brood inspection | No | none | No | NA | none |
| 2531 | 2022 | 2.903958 | Yes | Alcohol wash, Drone brood inspection | No | none | No | NA | none |
| 2532 | 2022 | 2.903958 | Yes | Alcohol wash, Drone brood inspection | No | none | No | NA | none |
| 2533 | 2022 | 2.903958 | Yes | Alcohol wash, Drone brood inspection | No | none | No | NA | none |
| 2534 | 2022 | 29.4811 | Yes | Drone brood inspection | No | none | Yes | Fondant or sugar candy | sugar only |
| 2535 | 2022 | 29.4811 | Yes | Drone brood inspection | No | none | Yes | Fondant or sugar candy | sugar only |
| 2536 | 2022 | 4.954668 | Yes | 48 hr drop (sticky board) | No | none | Yes | Fondant or sugar candy | sugar only |
| 2537 | 2022 | 0.826634 | Yes | Sugar roll | Yes | Oxalic Acid (Vapor) | No | NA | none |
| 2538 | 2022 | 0.826634 | Yes | Sugar roll | Yes | Oxalic Acid (Vapor) | No | NA | none |
| 2539 | 2022 | 31.26808 | Yes | Alcohol wash | Yes | Formic Acid (Mite Away Quick Strips or Formic Pro) | Yes | Fondant or sugar candy | sugar only |
| 2540 | 2022 | 31.26808 | Yes | Alcohol wash | Yes | Formic Acid (Mite Away Quick Strips or Formic Pro) | Yes | Fondant or sugar candy | sugar only |
| 2541 | 2022 | 31.26808 | Yes | Alcohol wash | Yes | Formic Acid (Mite Away Quick Strips or Formic Pro) | Yes | Fondant or sugar candy | sugar only |
| 2542 | 2022 | 0.18948 | No | NA | Yes | Oxalic Acid (Vapor) | Yes | Fondant or sugar candy | sugar only |
| 2543 | 2022 | 0.18948 | No | NA | Yes | Oxalic Acid (Vapor) | Yes | Fondant or sugar candy | sugar only |
| 2544 | 2022 | 0.18948 | No | NA | Yes | Oxalic Acid (Vapor) | Yes | Fondant or sugar candy | sugar only |
| 2545 | 2022 | 0.18948 | No | NA | Yes | Oxalic Acid (Vapor) | Yes | Fondant or sugar candy | sugar only |
| 2546 | 2022 | 0.18948 | No | NA | Yes | Oxalic Acid (Vapor) | Yes | Fondant or sugar candy | sugar only |
| 2547 | 2022 | 0.18948 | No | NA | Yes | Oxalic Acid (Vapor) | Yes | Fondant or sugar candy | sugar only |
| 2548 | 2022 | 0.18948 | No | NA | Yes | Oxalic Acid (Vapor) | Yes | Fondant or sugar candy | sugar only |
| 2549 | 2022 | 0.18948 | No | NA | Yes | Oxalic Acid (Vapor) | Yes | Fondant or sugar candy | sugar only |
| 2550 | 2022 | 0.18948 | No | NA | Yes | Oxalic Acid (Vapor) | Yes | Fondant or sugar candy | sugar only |
| 2551 | 2022 | 0.18948 | No | NA | Yes | Oxalic Acid (Vapor) | Yes | Fondant or sugar candy | sugar only |
| 2552 | 2022 | 0.18948 | No | NA | Yes | Oxalic Acid (Vapor) | Yes | Fondant or sugar candy | sugar only |
| 2553 | 2022 | 0.18948 | No | NA | Yes | Oxalic Acid (Vapor) | Yes | Fondant or sugar candy | sugar only |
| 2554 | 2022 | 0.18948 | No | NA | Yes | Oxalic Acid (Vapor) | Yes | Fondant or sugar candy | sugar only |
| 2555 | 2022 | 0.18948 | No | NA | Yes | Oxalic Acid (Vapor) | Yes | Fondant or sugar candy | sugar only |
| 2556 | 2022 | 0.18948 | No | NA | Yes | Oxalic Acid (Vapor) | Yes | Fondant or sugar candy | sugar only |
| 2557 | 2022 | 0.18948 | No | NA | Yes | Oxalic Acid (Vapor) | Yes | Fondant or sugar candy | sugar only |
| 2558 | 2022 | 0.18948 | No | NA | Yes | Oxalic Acid (Vapor) | Yes | Fondant or sugar candy | sugar only |
| 2559 | 2022 | 0.18948 | No | NA | Yes | Oxalic Acid (Vapor) | Yes | Fondant or sugar candy | sugar only |
| 2560 | 2022 | 0.18948 | No | NA | Yes | Oxalic Acid (Vapor) | Yes | Fondant or sugar candy | sugar only |
| 2561 | 2022 | 0.18948 | No | NA | Yes | Oxalic Acid (Vapor) | Yes | Fondant or sugar candy | sugar only |
| 2562 | 2022 | 0.18948 | No | NA | Yes | Oxalic Acid (Vapor) | Yes | Fondant or sugar candy | sugar only |
| 2563 | 2022 | 0.18948 | No | NA | Yes | Oxalic Acid (Vapor) | Yes | Fondant or sugar candy | sugar only |
| 2564 | 2022 | 0.18948 | No | NA | Yes | Oxalic Acid (Vapor) | Yes | Fondant or sugar candy | sugar only |
| 2565 | 2022 | 18.10874 | Yes | Alcohol wash | Yes | Formic Acid (Mite Away Quick Strips or Formic Pro) | No | NA | none |
| 2566 | 2022 | 18.10874 | Yes | Alcohol wash | Yes | Formic Acid (Mite Away Quick Strips or Formic Pro) | No | NA | none |
| 2567 | 2022 | 18.10874 | Yes | Alcohol wash | Yes | Formic Acid (Mite Away Quick Strips or Formic Pro) | No | NA | none |
| 2568 | 2022 | 0.263715 | No | NA | No | none | Yes | Dry sugar, Honey from your own stock | none |
| 2569 | 2022 | 0.263715 | No | NA | No | none | Yes | Dry sugar, Honey from your own stock | none |
| 2570 | 2022 | 5.559985 | No | NA | Yes | Formic Acid (Mite Away Quick Strips or Formic Pro) | Yes | Dry sugar | sugar only |
| 2571 | 2022 | 0.580336 | No | NA | Yes | Formic Acid (Mite Away Quick Strips or Formic Pro) | Yes | Fondant or sugar candy | sugar only |
| 2572 | 2022 | 1.376313 | No | NA | No | none | No | NA | none |
| 2573 | 2022 | 6.186008 | Yes | Alcohol wash | Yes | Formic Acid (Mite Away Quick Strips or Formic Pro) | Yes | Fondant or sugar candy | sugar only |
| 2574 | 2022 | 6.186008 | Yes | Alcohol wash | Yes | Formic Acid (Mite Away Quick Strips or Formic Pro) | Yes | Fondant or sugar candy | sugar only |
| 2575 | 2022 | 6.186008 | Yes | Alcohol wash | Yes | Formic Acid (Mite Away Quick Strips or Formic Pro) | Yes | Fondant or sugar candy | sugar only |
| 2576 | 2022 | 6.186008 | Yes | Alcohol wash | Yes | Formic Acid (Mite Away Quick Strips or Formic Pro) | Yes | Fondant or sugar candy | sugar only |
| 2577 | 2022 | 6.186008 | Yes | Alcohol wash | Yes | Formic Acid (Mite Away Quick Strips or Formic Pro) | Yes | Fondant or sugar candy | sugar only |
| 2578 | 2022 | 6.186008 | Yes | Alcohol wash | Yes | Formic Acid (Mite Away Quick Strips or Formic Pro) | Yes | Fondant or sugar candy | sugar only |
| 2579 | 2022 | 6.186008 | Yes | Alcohol wash | Yes | Formic Acid (Mite Away Quick Strips or Formic Pro) | Yes | Fondant or sugar candy | sugar only |
| 2580 | 2022 | 6.186008 | Yes | Alcohol wash | Yes | Formic Acid (Mite Away Quick Strips or Formic Pro) | Yes | Fondant or sugar candy | sugar only |
| 2581 | 2022 | 6.186008 | Yes | Alcohol wash | Yes | Formic Acid (Mite Away Quick Strips or Formic Pro) | Yes | Fondant or sugar candy | sugar only |
| 2582 | 2022 | 6.186008 | Yes | Alcohol wash | Yes | Formic Acid (Mite Away Quick Strips or Formic Pro) | Yes | Fondant or sugar candy | sugar only |
| 2583 | 2022 | 6.186008 | Yes | Alcohol wash | Yes | Formic Acid (Mite Away Quick Strips or Formic Pro) | Yes | Fondant or sugar candy | sugar only |
| 2584 | 2022 | 6.186008 | Yes | Alcohol wash | Yes | Formic Acid (Mite Away Quick Strips or Formic Pro) | Yes | Fondant or sugar candy | sugar only |
| 2585 | 2022 | 6.186008 | Yes | Alcohol wash | Yes | Formic Acid (Mite Away Quick Strips or Formic Pro) | Yes | Fondant or sugar candy | sugar only |
| 2586 | 2022 | 6.186008 | Yes | Alcohol wash | Yes | Formic Acid (Mite Away Quick Strips or Formic Pro) | Yes | Fondant or sugar candy | sugar only |
| 2587 | 2022 | 6.186008 | Yes | Alcohol wash | Yes | Formic Acid (Mite Away Quick Strips or Formic Pro) | Yes | Fondant or sugar candy | sugar only |
| 2588 | 2022 | 6.186008 | Yes | Alcohol wash | Yes | Formic Acid (Mite Away Quick Strips or Formic Pro) | Yes | Fondant or sugar candy | sugar only |
| 2589 | 2022 | 14.03052 | Yes | Sugar roll | Yes | Formic Acid (Mite Away Quick Strips or Formic Pro) | Yes | Fondant or sugar candy | sugar only |
| 2590 | 2022 | 14.03052 | Yes | Sugar roll | Yes | Formic Acid (Mite Away Quick Strips or Formic Pro) | Yes | Fondant or sugar candy | sugar only |
| 2591 | 2022 | 14.03052 | Yes | Sugar roll | Yes | Formic Acid (Mite Away Quick Strips or Formic Pro) | Yes | Fondant or sugar candy | sugar only |
| 2592 | 2022 | 0.184129 | Yes | Alcohol wash, Other | Yes | Formic Acid (Mite Away Quick Strips or Formic Pro) | Yes | Fondant or sugar candy | sugar only |
| 2593 | 2022 | 0.184129 | Yes | Alcohol wash, Other | Yes | Formic Acid (Mite Away Quick Strips or Formic Pro) | Yes | Fondant or sugar candy | sugar only |
| 2594 | 2022 | 0.184129 | Yes | Alcohol wash, Other | Yes | Formic Acid (Mite Away Quick Strips or Formic Pro) | Yes | Fondant or sugar candy | sugar only |
| 2595 | 2022 | 0.184129 | Yes | Alcohol wash, Other | Yes | Formic Acid (Mite Away Quick Strips or Formic Pro) | Yes | Fondant or sugar candy | sugar only |
| 2596 | 2022 | 0.184129 | Yes | Alcohol wash, Other | Yes | Formic Acid (Mite Away Quick Strips or Formic Pro) | Yes | Fondant or sugar candy | sugar only |
| 2597 | 2022 | 0.184129 | Yes | Alcohol wash, Other | Yes | Formic Acid (Mite Away Quick Strips or Formic Pro) | Yes | Fondant or sugar candy | sugar only |
| 2598 | 2022 | 0.184129 | Yes | Alcohol wash, Other | Yes | Formic Acid (Mite Away Quick Strips or Formic Pro) | Yes | Fondant or sugar candy | sugar only |
| 2599 | 2022 | 0.184129 | Yes | Alcohol wash, Other | Yes | Formic Acid (Mite Away Quick Strips or Formic Pro) | Yes | Fondant or sugar candy | sugar only |
| 2600 | 2022 | 2.000963 | Yes | Alcohol wash | Yes | Oxalic Acid (Vapor) | Yes | Fondant or sugar candy | sugar only |
| 2601 | 2022 | 2.000963 | Yes | Alcohol wash | Yes | Oxalic Acid (Vapor) | Yes | Fondant or sugar candy | sugar only |
| 2602 | 2022 | 2.000963 | Yes | Alcohol wash | Yes | Oxalic Acid (Vapor) | Yes | Fondant or sugar candy | sugar only |
| 2603 | 2022 | 2.000963 | Yes | Alcohol wash | Yes | Oxalic Acid (Vapor) | Yes | Fondant or sugar candy | sugar only |
| 2604 | 2022 | 2.000963 | Yes | Alcohol wash | Yes | Oxalic Acid (Vapor) | Yes | Fondant or sugar candy | sugar only |
| 2605 | 2022 | 2.000963 | Yes | Alcohol wash | Yes | Oxalic Acid (Vapor) | Yes | Fondant or sugar candy | sugar only |
| 2606 | 2022 | 32.08404 | No | NA | Yes | Other | Yes | Honey from your own stock | none |
| 2607 | 2022 | 32.08404 | No | NA | Yes | Other | Yes | Honey from your own stock | none |
| 2608 | 2022 | 32.08404 | No | NA | Yes | Other | Yes | Honey from your own stock | none |
| 2609 | 2022 | 32.08404 | No | NA | Yes | Other | Yes | Honey from your own stock | none |
| 2610 | 2022 | 32.08404 | No | NA | Yes | Other | Yes | Honey from your own stock | none |
| 2611 | 2022 | 21.32675 | No | NA | No | none | No | NA | none |
| 2612 | 2022 | 9.088616 | Yes | Alcohol wash, Drone brood inspection | Yes | Formic Acid (Mite Away Quick Strips or Formic Pro) | No | NA | none |
| 2613 | 2022 | 9.088616 | Yes | Alcohol wash, Drone brood inspection | Yes | Formic Acid (Mite Away Quick Strips or Formic Pro) | No | NA | none |
| 2614 | 2022 | 9.088616 | Yes | Alcohol wash, Drone brood inspection | Yes | Formic Acid (Mite Away Quick Strips or Formic Pro) | No | NA | none |
| 2615 | 2022 | 19.41322 | No | NA | No | none | No | NA | none |
| 2616 | 2022 | 19.41322 | No | NA | No | none | No | NA | none |
| 2617 | 2022 | 19.41322 | No | NA | No | none | No | NA | none |
| 2618 | 2022 | 19.41322 | No | NA | No | none | No | NA | none |
| 2619 | 2022 | 1.592081 | Yes | 48 hr drop (sticky board) | Yes | Api life Var | Yes | Fondant or sugar candy, Honey from your own stock | none |
| 2620 | 2022 | 1.592081 | Yes | 48 hr drop (sticky board) | Yes | Api life Var | Yes | Fondant or sugar candy, Honey from your own stock | none |
| 2621 | 2022 | 1.592081 | Yes | 48 hr drop (sticky board) | Yes | Api life Var | Yes | Fondant or sugar candy, Honey from your own stock | none |
| 2622 | 2022 | 1.592081 | Yes | 48 hr drop (sticky board) | Yes | Api life Var | Yes | Fondant or sugar candy, Honey from your own stock | none |
| 2623 | 2022 | 1.592081 | Yes | 48 hr drop (sticky board) | Yes | Api life Var | Yes | Fondant or sugar candy, Honey from your own stock | none |
| 2624 | 2022 | 1.592081 | Yes | 48 hr drop (sticky board) | Yes | Api life Var | Yes | Fondant or sugar candy, Honey from your own stock | none |
| 2625 | 2022 | 1.592081 | Yes | 48 hr drop (sticky board) | Yes | Api life Var | Yes | Fondant or sugar candy, Honey from your own stock | none |
| 2626 | 2022 | 1.592081 | Yes | 48 hr drop (sticky board) | Yes | Api life Var | Yes | Fondant or sugar candy, Honey from your own stock | none |
| 2627 | 2022 | 1.592081 | Yes | 48 hr drop (sticky board) | Yes | Api life Var | Yes | Fondant or sugar candy, Honey from your own stock | none |
| 2628 | 2022 | 1.592081 | Yes | 48 hr drop (sticky board) | Yes | Api life Var | Yes | Fondant or sugar candy, Honey from your own stock | none |
| 2629 | 2022 | 1.592081 | Yes | 48 hr drop (sticky board) | Yes | Api life Var | Yes | Fondant or sugar candy, Honey from your own stock | none |
| 2630 | 2022 | 1.592081 | Yes | 48 hr drop (sticky board) | Yes | Api life Var | Yes | Fondant or sugar candy, Honey from your own stock | none |
| 2631 | 2022 | 1.592081 | Yes | 48 hr drop (sticky board) | Yes | Api life Var | Yes | Fondant or sugar candy, Honey from your own stock | none |
| 2632 | 2022 | 1.592081 | Yes | 48 hr drop (sticky board) | Yes | Api life Var | Yes | Fondant or sugar candy, Honey from your own stock | none |
| 2633 | 2022 | 1.592081 | Yes | 48 hr drop (sticky board) | Yes | Api life Var | Yes | Fondant or sugar candy, Honey from your own stock | none |
| 2634 | 2022 | 1.592081 | Yes | 48 hr drop (sticky board) | Yes | Api life Var | Yes | Fondant or sugar candy, Honey from your own stock | none |
| 2635 | 2022 | 1.592081 | Yes | 48 hr drop (sticky board) | Yes | Api life Var | Yes | Fondant or sugar candy, Honey from your own stock | none |
| 2636 | 2022 | 1.592081 | Yes | 48 hr drop (sticky board) | Yes | Api life Var | Yes | Fondant or sugar candy, Honey from your own stock | none |
| 2637 | 2022 | 4.376916 | Yes | Drone brood inspection | Yes | Oxalic Acid (Vapor) | Yes | Pollen substitute, Commercially available supplements | none |
| 2638 | 2022 | 4.376916 | Yes | Drone brood inspection | Yes | Oxalic Acid (Vapor) | Yes | Pollen substitute, Commercially available supplements | none |
| 2639 | 2022 | 4.376916 | Yes | Drone brood inspection | Yes | Oxalic Acid (Vapor) | Yes | Pollen substitute, Commercially available supplements | none |
| 2640 | 2022 | 4.376916 | Yes | Drone brood inspection | Yes | Oxalic Acid (Vapor) | Yes | Pollen substitute, Commercially available supplements | none |
| 2641 | 2022 | 4.376916 | Yes | Drone brood inspection | Yes | Oxalic Acid (Vapor) | Yes | Pollen substitute, Commercially available supplements | none |
| 2642 | 2022 | 6.324415 | Yes | Drone brood inspection | Yes | Formic Acid (Mite Away Quick Strips or Formic Pro) | Yes | Fondant or sugar candy | sugar only |
| 2643 | 2022 | 28.21171 | Yes | Sugar roll | No | none | Yes | Sugar syrup, Pollen substitute, Honey from your own stock | none |
| 2644 | 2022 | 28.21171 | Yes | Sugar roll | No | none | Yes | Sugar syrup, Pollen substitute, Honey from your own stock | none |
| 2645 | 2022 | 8.752937 | Yes | Alcohol wash | Yes | Formic Acid (Mite Away Quick Strips or Formic Pro) | Yes | Fondant or sugar candy, Dry sugar | none |
| 2646 | 2022 | 8.752937 | Yes | Alcohol wash | Yes | Formic Acid (Mite Away Quick Strips or Formic Pro) | Yes | Fondant or sugar candy, Dry sugar | none |
| 2647 | 2022 | 8.752937 | Yes | Alcohol wash | Yes | Formic Acid (Mite Away Quick Strips or Formic Pro) | Yes | Fondant or sugar candy, Dry sugar | none |
| 2648 | 2022 | 8.752937 | Yes | Alcohol wash | Yes | Formic Acid (Mite Away Quick Strips or Formic Pro) | Yes | Fondant or sugar candy, Dry sugar | none |
| 2649 | 2022 | 8.752937 | Yes | Alcohol wash | Yes | Formic Acid (Mite Away Quick Strips or Formic Pro) | Yes | Fondant or sugar candy, Dry sugar | none |
| 2650 | 2022 | 8.752937 | Yes | Alcohol wash | Yes | Formic Acid (Mite Away Quick Strips or Formic Pro) | Yes | Fondant or sugar candy, Dry sugar | none |
| 2651 | 2022 | 8.752937 | Yes | Alcohol wash | Yes | Formic Acid (Mite Away Quick Strips or Formic Pro) | Yes | Fondant or sugar candy, Dry sugar | none |
| 2652 | 2022 | 8.752937 | Yes | Alcohol wash | Yes | Formic Acid (Mite Away Quick Strips or Formic Pro) | Yes | Fondant or sugar candy, Dry sugar | none |
| 2653 | 2022 | 8.752937 | Yes | Alcohol wash | Yes | Formic Acid (Mite Away Quick Strips or Formic Pro) | Yes | Fondant or sugar candy, Dry sugar | none |
| 2654 | 2022 | 8.752937 | Yes | Alcohol wash | Yes | Formic Acid (Mite Away Quick Strips or Formic Pro) | Yes | Fondant or sugar candy, Dry sugar | none |
| 2655 | 2022 | 8.752937 | Yes | Alcohol wash | Yes | Formic Acid (Mite Away Quick Strips or Formic Pro) | Yes | Fondant or sugar candy, Dry sugar | none |
| 2656 | 2022 | 8.752937 | Yes | Alcohol wash | Yes | Formic Acid (Mite Away Quick Strips or Formic Pro) | Yes | Fondant or sugar candy, Dry sugar | none |
| 2657 | 2022 | 8.752937 | Yes | Alcohol wash | Yes | Formic Acid (Mite Away Quick Strips or Formic Pro) | Yes | Fondant or sugar candy, Dry sugar | none |
| 2658 | 2022 | 8.752937 | Yes | Alcohol wash | Yes | Formic Acid (Mite Away Quick Strips or Formic Pro) | Yes | Fondant or sugar candy, Dry sugar | none |
| 2659 | 2022 | 8.752937 | Yes | Alcohol wash | Yes | Formic Acid (Mite Away Quick Strips or Formic Pro) | Yes | Fondant or sugar candy, Dry sugar | none |
| 2660 | 2022 | 8.752937 | Yes | Alcohol wash | Yes | Formic Acid (Mite Away Quick Strips or Formic Pro) | Yes | Fondant or sugar candy, Dry sugar | none |
| 2661 | 2022 | 8.752937 | Yes | Alcohol wash | Yes | Formic Acid (Mite Away Quick Strips or Formic Pro) | Yes | Fondant or sugar candy, Dry sugar | none |
| 2662 | 2022 | 8.752937 | Yes | Alcohol wash | Yes | Formic Acid (Mite Away Quick Strips or Formic Pro) | Yes | Fondant or sugar candy, Dry sugar | none |
| 2663 | 2022 | 8.752937 | Yes | Alcohol wash | Yes | Formic Acid (Mite Away Quick Strips or Formic Pro) | Yes | Fondant or sugar candy, Dry sugar | none |
| 2664 | 2022 | 8.752937 | Yes | Alcohol wash | Yes | Formic Acid (Mite Away Quick Strips or Formic Pro) | Yes | Fondant or sugar candy, Dry sugar | none |
| 2665 | 2022 | 8.752937 | Yes | Alcohol wash | Yes | Formic Acid (Mite Away Quick Strips or Formic Pro) | Yes | Fondant or sugar candy, Dry sugar | none |
| 2666 | 2022 | 8.752937 | Yes | Alcohol wash | Yes | Formic Acid (Mite Away Quick Strips or Formic Pro) | Yes | Fondant or sugar candy, Dry sugar | none |
| 2667 | 2022 | 8.752937 | Yes | Alcohol wash | Yes | Formic Acid (Mite Away Quick Strips or Formic Pro) | Yes | Fondant or sugar candy, Dry sugar | none |
| 2668 | 2022 | 8.752937 | Yes | Alcohol wash | Yes | Formic Acid (Mite Away Quick Strips or Formic Pro) | Yes | Fondant or sugar candy, Dry sugar | none |
| 2669 | 2022 | 5.212239 | Yes | Alcohol wash | Yes | Formic Acid (Mite Away Quick Strips or Formic Pro) | Yes | Fondant or sugar candy, Honey from your own stock, Pollen from your own stock | none |
| 2670 | 2022 | 5.212239 | Yes | Alcohol wash | Yes | Formic Acid (Mite Away Quick Strips or Formic Pro) | Yes | Fondant or sugar candy, Honey from your own stock, Pollen from your own stock | none |
| 2671 | 2022 | 5.212239 | Yes | Alcohol wash | Yes | Formic Acid (Mite Away Quick Strips or Formic Pro) | Yes | Fondant or sugar candy, Honey from your own stock, Pollen from your own stock | none |
| 2672 | 2022 | 5.212239 | Yes | Alcohol wash | Yes | Formic Acid (Mite Away Quick Strips or Formic Pro) | Yes | Fondant or sugar candy, Honey from your own stock, Pollen from your own stock | none |
| 2673 | 2022 | 36.76253 | Yes | Drone brood inspection | No | none | No | NA | none |
| 2674 | 2022 | 36.76253 | Yes | Drone brood inspection | No | none | No | NA | none |
| 2675 | 2022 | 36.76253 | Yes | Drone brood inspection | No | none | No | NA | none |
| 2676 | 2022 | 36.76253 | Yes | Drone brood inspection | No | none | No | NA | none |
| 2677 | 2022 | 36.76253 | Yes | Drone brood inspection | No | none | No | NA | none |
| 2678 | 2022 | 36.76253 | Yes | Drone brood inspection | No | none | No | NA | none |
| 2679 | 2022 | 0.562467 | No | NA | Yes | Apistan | No | NA | none |
| 2680 | 2022 | 0.562467 | No | NA | Yes | Apistan | No | NA | none |
| 2681 | 2022 | 0.562467 | No | NA | Yes | Apistan | No | NA | none |
| 2682 | 2022 | 11.88588 | Yes | Sugar roll | Yes | Formic Acid (Mite Away Quick Strips or Formic Pro) | No | NA | none |
| 2683 | 2022 | 11.88588 | Yes | Sugar roll | Yes | Formic Acid (Mite Away Quick Strips or Formic Pro) | No | NA | none |
| 2684 | 2022 | 8.318106 | Yes | Sugar roll | Yes | Formic Acid (Mite Away Quick Strips or Formic Pro) | Yes | Fondant or sugar candy, Sugar syrup | none |
| 2685 | 2022 | 8.318106 | Yes | Sugar roll | Yes | Formic Acid (Mite Away Quick Strips or Formic Pro) | Yes | Fondant or sugar candy, Sugar syrup | none |
| 2686 | 2022 | 8.318106 | Yes | Sugar roll | Yes | Formic Acid (Mite Away Quick Strips or Formic Pro) | Yes | Fondant or sugar candy, Sugar syrup | none |
| 2687 | 2022 | 8.318106 | Yes | Sugar roll | Yes | Formic Acid (Mite Away Quick Strips or Formic Pro) | Yes | Fondant or sugar candy, Sugar syrup | none |
| 2688 | 2022 | 8.318106 | Yes | Sugar roll | Yes | Formic Acid (Mite Away Quick Strips or Formic Pro) | Yes | Fondant or sugar candy, Sugar syrup | none |
| 2689 | 2022 | 11.93223 | Yes | Alcohol wash | Yes | Oxalic Acid (Vapor) | Yes | Sugar syrup, Honey from your own stock | none |
| 2690 | 2022 | 11.93223 | Yes | Alcohol wash | Yes | Oxalic Acid (Vapor) | Yes | Sugar syrup, Honey from your own stock | none |
| 2691 | 2022 | 11.93223 | Yes | Alcohol wash | Yes | Oxalic Acid (Vapor) | Yes | Sugar syrup, Honey from your own stock | none |
| 2692 | 2022 | 11.93223 | Yes | Alcohol wash | Yes | Oxalic Acid (Vapor) | Yes | Sugar syrup, Honey from your own stock | none |
| 2693 | 2022 | 11.93223 | Yes | Alcohol wash | Yes | Oxalic Acid (Vapor) | Yes | Sugar syrup, Honey from your own stock | none |
| 2694 | 2022 | 11.93223 | Yes | Alcohol wash | Yes | Oxalic Acid (Vapor) | Yes | Sugar syrup, Honey from your own stock | none |
| 2695 | 2022 | 11.93223 | Yes | Alcohol wash | Yes | Oxalic Acid (Vapor) | Yes | Sugar syrup, Honey from your own stock | none |
| 2696 | 2022 | 11.93223 | Yes | Alcohol wash | Yes | Oxalic Acid (Vapor) | Yes | Sugar syrup, Honey from your own stock | none |
| 2697 | 2022 | 11.93223 | Yes | Alcohol wash | Yes | Oxalic Acid (Vapor) | Yes | Sugar syrup, Honey from your own stock | none |
| 2698 | 2022 | 11.93223 | Yes | Alcohol wash | Yes | Oxalic Acid (Vapor) | Yes | Sugar syrup, Honey from your own stock | none |
| 2699 | 2022 | 0.580353 | No | NA | Yes | Formic Acid (Mite Away Quick Strips or Formic Pro) | Yes | Dry sugar, Pollen substitute | none |
| 2700 | 2022 | 0.580353 | No | NA | Yes | Formic Acid (Mite Away Quick Strips or Formic Pro) | Yes | Dry sugar, Pollen substitute | none |
| 2701 | 2022 | 0.580353 | No | NA | Yes | Formic Acid (Mite Away Quick Strips or Formic Pro) | Yes | Dry sugar, Pollen substitute | none |
| 2702 | 2022 | 0.713409 | Yes | Other | No | none | Yes | Fondant or sugar candy, Sugar syrup, Dry sugar | none |
| 2703 | 2022 | 0.713409 | Yes | Other | No | none | Yes | Fondant or sugar candy, Sugar syrup, Dry sugar | none |
| 2704 | 2022 | 0.29682 | No | NA | Yes | Apivar (Amitraz) | No | NA | none |
| 2705 | 2022 | 0.29682 | No | NA | Yes | Apivar (Amitraz) | No | NA | none |
| 2706 | 2022 | 0.29682 | No | NA | Yes | Apivar (Amitraz) | No | NA | none |
| 2707 | 2022 | 0.29682 | No | NA | Yes | Apivar (Amitraz) | No | NA | none |
| 2708 | 2022 | 0.29682 | No | NA | Yes | Apivar (Amitraz) | No | NA | none |
| 2709 | 2022 | 0.29682 | No | NA | Yes | Apivar (Amitraz) | No | NA | none |
| 2710 | 2022 | 0.29682 | No | NA | Yes | Apivar (Amitraz) | No | NA | none |
| 2711 | 2022 | 0.29682 | No | NA | Yes | Apivar (Amitraz) | No | NA | none |
| 2712 | 2022 | 0.29682 | No | NA | Yes | Apivar (Amitraz) | No | NA | none |
| 2713 | 2022 | 0.29682 | No | NA | Yes | Apivar (Amitraz) | No | NA | none |
| 2714 | 2022 | 0.29682 | No | NA | Yes | Apivar (Amitraz) | No | NA | none |
| 2715 | 2022 | 0.29682 | No | NA | Yes | Apivar (Amitraz) | No | NA | none |
| 2716 | 2022 | 0.29682 | No | NA | Yes | Apivar (Amitraz) | No | NA | none |
| 2717 | 2022 | 0.29682 | No | NA | Yes | Apivar (Amitraz) | No | NA | none |
| 2718 | 2022 | 0.29682 | No | NA | Yes | Apivar (Amitraz) | No | NA | none |
| 2719 | 2022 | 0.29682 | No | NA | Yes | Apivar (Amitraz) | No | NA | none |
| 2720 | 2022 | 0.29682 | No | NA | Yes | Apivar (Amitraz) | No | NA | none |
| 2721 | 2022 | 0.29682 | No | NA | Yes | Apivar (Amitraz) | No | NA | none |
| 2722 | 2022 | 0.29682 | No | NA | Yes | Apivar (Amitraz) | No | NA | none |
| 2723 | 2022 | 1.398594 | Yes | Sugar roll | No | none | No | NA | none |
| 2724 | 2022 | 5.956534 | Yes | Sugar roll | Yes | Formic Acid (Mite Away Quick Strips or Formic Pro) | Yes | Fondant or sugar candy | sugar only |
| 2725 | 2022 | 5.956534 | Yes | Sugar roll | Yes | Formic Acid (Mite Away Quick Strips or Formic Pro) | Yes | Fondant or sugar candy | sugar only |
| 2726 | 2022 | 5.956534 | Yes | Sugar roll | Yes | Formic Acid (Mite Away Quick Strips or Formic Pro) | Yes | Fondant or sugar candy | sugar only |
| 2727 | 2022 | 18.61326 | Yes | 48 hr drop (sticky board) | Yes | Formic Acid (Mite Away Quick Strips or Formic Pro) | Yes | Fondant or sugar candy | sugar only |
| 2728 | 2022 | 18.61326 | Yes | 48 hr drop (sticky board) | Yes | Formic Acid (Mite Away Quick Strips or Formic Pro) | Yes | Fondant or sugar candy | sugar only |
| 2729 | 2022 | 6.129009 | Yes | Sugar roll | Yes | Oxalic Acid (Dribble) | Yes | Dry sugar | sugar only |
| 2730 | 2022 | 1.533366 | Yes | Alcohol wash, Drone brood inspection | Yes | Formic Acid (Mite Away Quick Strips or Formic Pro) | Yes | Fondant or sugar candy | sugar only |
| 2731 | 2022 | 1.533366 | Yes | Alcohol wash, Drone brood inspection | Yes | Formic Acid (Mite Away Quick Strips or Formic Pro) | Yes | Fondant or sugar candy | sugar only |
| 2732 | 2022 | 1.533366 | Yes | Alcohol wash, Drone brood inspection | Yes | Formic Acid (Mite Away Quick Strips or Formic Pro) | Yes | Fondant or sugar candy | sugar only |
| 2733 | 2022 | 1.533366 | Yes | Alcohol wash, Drone brood inspection | Yes | Formic Acid (Mite Away Quick Strips or Formic Pro) | Yes | Fondant or sugar candy | sugar only |
| 2734 | 2022 | 42.88483 | Yes | Alcohol wash | No | none | Yes | Sugar syrup | none |
| 2735 | 2022 | 24.02668 | No | NA | Yes | Oxalic Acid (Vapor) | No | NA | none |
| 2736 | 2022 | 24.02668 | No | NA | Yes | Oxalic Acid (Vapor) | No | NA | none |
| 2737 | 2022 | 24.02668 | No | NA | Yes | Oxalic Acid (Vapor) | No | NA | none |
| 2738 | 2022 | 24.02668 | No | NA | Yes | Oxalic Acid (Vapor) | No | NA | none |
| 2739 | 2022 | 1.426768 | Yes | Alcohol wash | No | none | Yes | Fondant or sugar candy | sugar only |
| 2740 | 2022 | 1.426768 | Yes | Alcohol wash | No | none | Yes | Fondant or sugar candy | sugar only |
| 2741 | 2022 | 1.260604 | Yes | Alcohol wash | Yes | Apivar (Amitraz) | No | NA | none |
| 2742 | 2022 | 1.260604 | Yes | Alcohol wash | Yes | Apivar (Amitraz) | No | NA | none |
| 2743 | 2022 | 1.260604 | Yes | Alcohol wash | Yes | Apivar (Amitraz) | No | NA | none |
| 2744 | 2022 | 1.260604 | Yes | Alcohol wash | Yes | Apivar (Amitraz) | No | NA | none |
| 2745 | 2022 | 1.260604 | Yes | Alcohol wash | Yes | Apivar (Amitraz) | No | NA | none |
| 2746 | 2022 | 1.958834 | Yes | Drone brood inspection | Yes | Oxalic Acid (Vapor) | Yes | Fondant or sugar candy, Pollen from your own stock | none |
| 2747 | 2022 | 1.958834 | Yes | Drone brood inspection | Yes | Oxalic Acid (Vapor) | Yes | Fondant or sugar candy, Pollen from your own stock | none |
| 2748 | 2022 | 0.626575 | No | NA | No | none | No | NA | none |
| 2749 | 2022 | 0.626575 | No | NA | No | none | No | NA | none |
| 2750 | 2022 | 5.820803 | No | NA | No | none | Yes | Sugar syrup | none |
| 2751 | 2022 | 5.820803 | No | NA | No | none | Yes | Sugar syrup | none |
| 2752 | 2022 | 5.820803 | No | NA | No | none | Yes | Sugar syrup | none |
| 2753 | 2022 | 5.570883 | No | NA | Yes | Oxalic Acid (Vapor) | Yes | Sugar syrup | none |
| 2754 | 2022 | 5.570883 | No | NA | Yes | Oxalic Acid (Vapor) | Yes | Sugar syrup | none |
| 2755 | 2022 | 5.570883 | No | NA | Yes | Oxalic Acid (Vapor) | Yes | Sugar syrup | none |
| 2756 | 2022 | 30.47172 | Yes | Sugar roll | Yes | Oxalic Acid (Dribble) | Yes | Fondant or sugar candy | sugar only |
| 2757 | 2022 | 30.47172 | Yes | Sugar roll | Yes | Oxalic Acid (Dribble) | Yes | Fondant or sugar candy | sugar only |
| 2758 | 2022 | 0.62874 | Yes | Sugar roll, Drone brood inspection | Yes | Formic Acid (Mite Away Quick Strips or Formic Pro) | Yes | Fondant or sugar candy, Pollen substitute | none |
| 2759 | 2022 | 0.62874 | Yes | Sugar roll, Drone brood inspection | Yes | Formic Acid (Mite Away Quick Strips or Formic Pro) | Yes | Fondant or sugar candy, Pollen substitute | none |
| 2760 | 2022 | 0.62874 | Yes | Sugar roll, Drone brood inspection | Yes | Formic Acid (Mite Away Quick Strips or Formic Pro) | Yes | Fondant or sugar candy, Pollen substitute | none |
| 2761 | 2022 | 0.62874 | Yes | Sugar roll, Drone brood inspection | Yes | Formic Acid (Mite Away Quick Strips or Formic Pro) | Yes | Fondant or sugar candy, Pollen substitute | none |
| 2762 | 2022 | 0.62874 | Yes | Sugar roll, Drone brood inspection | Yes | Formic Acid (Mite Away Quick Strips or Formic Pro) | Yes | Fondant or sugar candy, Pollen substitute | none |
| 2763 | 2022 | 17.86652 | No | NA | No | none | Yes | Sugar syrup | none |
| 2764 | 2022 | 17.86652 | No | NA | No | none | Yes | Sugar syrup | none |
| 2765 | 2022 | 17.86652 | No | NA | No | none | Yes | Sugar syrup | none |
| 2766 | 2022 | 17.86652 | No | NA | No | none | Yes | Sugar syrup | none |
| 2767 | 2022 | 17.86652 | No | NA | No | none | Yes | Sugar syrup | none |
| 2768 | 2022 | 17.86652 | No | NA | No | none | Yes | Sugar syrup | none |
| 2769 | 2022 | 17.86652 | No | NA | No | none | Yes | Sugar syrup | none |
| 2770 | 2022 | 17.86652 | No | NA | No | none | Yes | Sugar syrup | none |
| 2771 | 2022 | 17.86652 | No | NA | No | none | Yes | Sugar syrup | none |
| 2772 | 2022 | 17.86652 | No | NA | No | none | Yes | Sugar syrup | none |
| 2773 | 2022 | 17.86652 | No | NA | No | none | Yes | Sugar syrup | none |
| 2774 | 2022 | 17.86652 | No | NA | No | none | Yes | Sugar syrup | none |
| 2775 | 2022 | 5.033648 | Yes | Sugar roll | Yes | Oxalic Acid (Vapor) | Yes | Sugar syrup | none |
| 2776 | 2022 | 5.033648 | Yes | Sugar roll | Yes | Oxalic Acid (Vapor) | Yes | Sugar syrup | none |
| 2777 | 2022 | 1.498173 | Yes | 48 hr drop (sticky board) | Yes | Formic Acid (Mite Away Quick Strips or Formic Pro) | Yes | Fondant or sugar candy, Honey from your own stock | none |
| 2778 | 2022 | 1.498173 | Yes | 48 hr drop (sticky board) | Yes | Formic Acid (Mite Away Quick Strips or Formic Pro) | Yes | Fondant or sugar candy, Honey from your own stock | none |
| 2779 | 2022 | 1.498173 | Yes | 48 hr drop (sticky board) | Yes | Formic Acid (Mite Away Quick Strips or Formic Pro) | Yes | Fondant or sugar candy, Honey from your own stock | none |
| 2780 | 2022 | 1.498173 | Yes | 48 hr drop (sticky board) | Yes | Formic Acid (Mite Away Quick Strips or Formic Pro) | Yes | Fondant or sugar candy, Honey from your own stock | none |
| 2781 | 2022 | 1.498173 | Yes | 48 hr drop (sticky board) | Yes | Formic Acid (Mite Away Quick Strips or Formic Pro) | Yes | Fondant or sugar candy, Honey from your own stock | none |
| 2782 | 2022 | 9.152787 | Yes | Alcohol wash | Yes | Oxalic Acid (Vapor) | Yes | Fondant or sugar candy, Pollen substitute | none |
| 2783 | 2022 | 9.152787 | Yes | Alcohol wash | Yes | Oxalic Acid (Vapor) | Yes | Fondant or sugar candy, Pollen substitute | none |
| 2784 | 2022 | 9.152787 | Yes | Alcohol wash | Yes | Oxalic Acid (Vapor) | Yes | Fondant or sugar candy, Pollen substitute | none |
| 2785 | 2022 | 9.152787 | Yes | Alcohol wash | Yes | Oxalic Acid (Vapor) | Yes | Fondant or sugar candy, Pollen substitute | none |
| 2786 | 2022 | 9.152787 | Yes | Alcohol wash | Yes | Oxalic Acid (Vapor) | Yes | Fondant or sugar candy, Pollen substitute | none |
| 2787 | 2022 | 23.53965 | Yes | Alcohol wash | Yes | Formic Acid (Mite Away Quick Strips or Formic Pro) | Yes | Dry sugar | sugar only |
| 2788 | 2022 | 23.53965 | Yes | Alcohol wash | Yes | Formic Acid (Mite Away Quick Strips or Formic Pro) | Yes | Dry sugar | sugar only |
| 2789 | 2022 | 23.53965 | Yes | Alcohol wash | Yes | Formic Acid (Mite Away Quick Strips or Formic Pro) | Yes | Dry sugar | sugar only |
| 2790 | 2022 | 23.53965 | Yes | Alcohol wash | Yes | Formic Acid (Mite Away Quick Strips or Formic Pro) | Yes | Dry sugar | sugar only |
| 2791 | 2022 | 10.25646 | Yes | Sugar roll | Yes | Formic Acid (Mite Away Quick Strips or Formic Pro) | Yes | Fondant or sugar candy, Pollen substitute | none |
| 2792 | 2022 | 10.25646 | Yes | Sugar roll | Yes | Formic Acid (Mite Away Quick Strips or Formic Pro) | Yes | Fondant or sugar candy, Pollen substitute | none |
| 2793 | 2022 | 35.41107 | Yes | Other | Yes | Oxalic Acid (Dribble) | No | NA | none |
| 2794 | 2022 | 35.41107 | Yes | Other | Yes | Oxalic Acid (Dribble) | No | NA | none |
| 2795 | 2022 | 19.97152 | No | NA | No | none | Yes | Dry sugar | sugar only |
| 2796 | 2022 | 19.97152 | No | NA | No | none | Yes | Dry sugar | sugar only |
| 2797 | 2022 | 19.97152 | No | NA | No | none | Yes | Dry sugar | sugar only |
| 2798 | 2022 | 19.97152 | No | NA | No | none | Yes | Dry sugar | sugar only |
| 2799 | 2022 | 3.474743 | Yes | Alcohol wash | Yes | Formic Acid (Mite Away Quick Strips or Formic Pro) | Yes | Dry sugar | sugar only |
| 2800 | 2022 | 3.474743 | Yes | Alcohol wash | Yes | Formic Acid (Mite Away Quick Strips or Formic Pro) | Yes | Dry sugar | sugar only |
| 2801 | 2022 | 4.068771 | Yes | 48 hr drop (sticky board) | Yes | Formic Acid (Mite Away Quick Strips or Formic Pro) | Yes | Dry sugar | sugar only |
| 2802 | 2022 | 6.971424 | No | NA | Yes | Oxalic Acid (Vapor) | Yes | Fondant or sugar candy, Dry sugar | none |
| 2803 | 2022 | 6.971424 | No | NA | Yes | Oxalic Acid (Vapor) | Yes | Fondant or sugar candy, Dry sugar | none |
| 2804 | 2022 | 9.67598 | No | NA | Yes | Apivar (Amitraz) | Yes | Commercially available supplements | none |
| 2805 | 2022 | 9.67598 | No | NA | Yes | Apivar (Amitraz) | Yes | Commercially available supplements | none |
| 2806 | 2022 | 2.393757 | Yes | Alcohol wash | Yes | Apivar (Amitraz) | No | NA | none |
| 2807 | 2022 | 2.923231 | Yes | Other | Yes | Oxalic Acid (Vapor) | Yes | Fondant or sugar candy | sugar only |
| 2808 | 2022 | 2.923231 | Yes | Other | Yes | Oxalic Acid (Vapor) | Yes | Fondant or sugar candy | sugar only |
| 2809 | 2022 | 2.923231 | Yes | Other | Yes | Oxalic Acid (Vapor) | Yes | Fondant or sugar candy | sugar only |
| 2810 | 2022 | 2.923231 | Yes | Other | Yes | Oxalic Acid (Vapor) | Yes | Fondant or sugar candy | sugar only |
| 2811 | 2022 | 2.923231 | Yes | Other | Yes | Oxalic Acid (Vapor) | Yes | Fondant or sugar candy | sugar only |
| 2812 | 2022 | 2.923231 | Yes | Other | Yes | Oxalic Acid (Vapor) | Yes | Fondant or sugar candy | sugar only |
| 2813 | 2022 | 2.923231 | Yes | Other | Yes | Oxalic Acid (Vapor) | Yes | Fondant or sugar candy | sugar only |
| 2814 | 2022 | 2.923231 | Yes | Other | Yes | Oxalic Acid (Vapor) | Yes | Fondant or sugar candy | sugar only |
| 2815 | 2022 | 2.923231 | Yes | Other | Yes | Oxalic Acid (Vapor) | Yes | Fondant or sugar candy | sugar only |
| 2816 | 2022 | 2.923231 | Yes | Other | Yes | Oxalic Acid (Vapor) | Yes | Fondant or sugar candy | sugar only |
| 2817 | 2022 | 1.543537 | Yes | Sugar roll | Yes | Formic Acid (Mite Away Quick Strips or Formic Pro) | Yes | Commercially available supplements | none |
| 2818 | 2022 | 1.543537 | Yes | Sugar roll | Yes | Formic Acid (Mite Away Quick Strips or Formic Pro) | Yes | Commercially available supplements | none |
| 2819 | 2022 | 1.543537 | Yes | Sugar roll | Yes | Formic Acid (Mite Away Quick Strips or Formic Pro) | Yes | Commercially available supplements | none |
| 2820 | 2022 | 3.042271 | Yes | Sugar roll, Drone brood inspection | Yes | Apivar (Amitraz) | Yes | Pollen substitute | pollen only |
| 2821 | 2022 | 15.09916 | No | NA | No | none | Yes | Fondant or sugar candy | sugar only |
| 2822 | 2022 | 3.13478 | Yes | Alcohol wash | Yes | Apivar (Amitraz) | No | NA | none |
| 2823 | 2022 | 3.13478 | Yes | Alcohol wash | Yes | Apivar (Amitraz) | No | NA | none |
| 2824 | 2022 | 3.13478 | Yes | Alcohol wash | Yes | Apivar (Amitraz) | No | NA | none |
| 2825 | 2022 | 3.13478 | Yes | Alcohol wash | Yes | Apivar (Amitraz) | No | NA | none |
| 2826 | 2022 | 3.13478 | Yes | Alcohol wash | Yes | Apivar (Amitraz) | No | NA | none |
| 2827 | 2022 | 3.13478 | Yes | Alcohol wash | Yes | Apivar (Amitraz) | No | NA | none |
| 2828 | 2022 | 3.13478 | Yes | Alcohol wash | Yes | Apivar (Amitraz) | No | NA | none |
| 2829 | 2022 | 3.13478 | Yes | Alcohol wash | Yes | Apivar (Amitraz) | No | NA | none |
| 2830 | 2022 | 3.13478 | Yes | Alcohol wash | Yes | Apivar (Amitraz) | No | NA | none |
| 2831 | 2022 | 3.13478 | Yes | Alcohol wash | Yes | Apivar (Amitraz) | No | NA | none |
| 2832 | 2022 | 3.13478 | Yes | Alcohol wash | Yes | Apivar (Amitraz) | No | NA | none |
| 2833 | 2022 | 3.13478 | Yes | Alcohol wash | Yes | Apivar (Amitraz) | No | NA | none |
| 2834 | 2022 | 3.13478 | Yes | Alcohol wash | Yes | Apivar (Amitraz) | No | NA | none |
| 2835 | 2022 | 3.13478 | Yes | Alcohol wash | Yes | Apivar (Amitraz) | No | NA | none |
| 2836 | 2022 | 3.13478 | Yes | Alcohol wash | Yes | Apivar (Amitraz) | No | NA | none |
| 2837 | 2022 | 3.13478 | Yes | Alcohol wash | Yes | Apivar (Amitraz) | No | NA | none |
| 2838 | 2022 | 3.13478 | Yes | Alcohol wash | Yes | Apivar (Amitraz) | No | NA | none |
| 2839 | 2022 | 3.13478 | Yes | Alcohol wash | Yes | Apivar (Amitraz) | No | NA | none |
| 2840 | 2022 | 3.13478 | Yes | Alcohol wash | Yes | Apivar (Amitraz) | No | NA | none |
| 2841 | 2022 | 3.13478 | Yes | Alcohol wash | Yes | Apivar (Amitraz) | No | NA | none |
| 2842 | 2022 | 31.40784 | Yes | Drone brood inspection | Yes | Apivar (Amitraz) | Yes | Commercially available supplements | none |
| 2843 | 2022 | 1.218419 | Yes | Sugar roll | Yes | Formic Acid (Mite Away Quick Strips or Formic Pro) | Yes | Fondant or sugar candy | sugar only |
| 2844 | 2022 | 1.547156 | Yes | Alcohol wash | Yes | Oxalic Acid (Vapor) | No | NA | none |
| 2845 | 2022 | 1.547156 | Yes | Alcohol wash | Yes | Oxalic Acid (Vapor) | No | NA | none |
| 2846 | 2022 | 1.547156 | Yes | Alcohol wash | Yes | Oxalic Acid (Vapor) | No | NA | none |
| 2847 | 2022 | 1.547156 | Yes | Alcohol wash | Yes | Oxalic Acid (Vapor) | No | NA | none |
| 2848 | 2022 | 1.547156 | Yes | Alcohol wash | Yes | Oxalic Acid (Vapor) | No | NA | none |
| 2849 | 2022 | 14.72996 | Yes | Sugar roll | Yes | Formic Acid (Mite Away Quick Strips or Formic Pro) | Yes | Fondant or sugar candy, Commercially available supplements | none |
| 2850 | 2022 | 14.72996 | Yes | Sugar roll | Yes | Formic Acid (Mite Away Quick Strips or Formic Pro) | Yes | Fondant or sugar candy, Commercially available supplements | none |
| 2851 | 2022 | 14.72996 | Yes | Sugar roll | Yes | Formic Acid (Mite Away Quick Strips or Formic Pro) | Yes | Fondant or sugar candy, Commercially available supplements | none |
| 2852 | 2022 | 14.72996 | Yes | Sugar roll | Yes | Formic Acid (Mite Away Quick Strips or Formic Pro) | Yes | Fondant or sugar candy, Commercially available supplements | none |
| 2853 | 2022 | 14.72996 | Yes | Sugar roll | Yes | Formic Acid (Mite Away Quick Strips or Formic Pro) | Yes | Fondant or sugar candy, Commercially available supplements | none |
| 2854 | 2022 | 14.72996 | Yes | Sugar roll | Yes | Formic Acid (Mite Away Quick Strips or Formic Pro) | Yes | Fondant or sugar candy, Commercially available supplements | none |
| 2855 | 2022 | 14.72996 | Yes | Sugar roll | Yes | Formic Acid (Mite Away Quick Strips or Formic Pro) | Yes | Fondant or sugar candy, Commercially available supplements | none |
| 2856 | 2022 | 14.72996 | Yes | Sugar roll | Yes | Formic Acid (Mite Away Quick Strips or Formic Pro) | Yes | Fondant or sugar candy, Commercially available supplements | none |
| 2857 | 2022 | 14.72996 | Yes | Sugar roll | Yes | Formic Acid (Mite Away Quick Strips or Formic Pro) | Yes | Fondant or sugar candy, Commercially available supplements | none |
| 2858 | 2022 | 7.329462 | Yes | Alcohol wash | Yes | Formic Acid (Mite Away Quick Strips or Formic Pro) | Yes | Fondant or sugar candy, Honey from your own stock, Probiotics | none |
| 2859 | 2022 | 7.329462 | Yes | Alcohol wash | Yes | Formic Acid (Mite Away Quick Strips or Formic Pro) | Yes | Fondant or sugar candy, Honey from your own stock, Probiotics | none |
| 2860 | 2022 | 17.01562 | Yes | Sugar roll | Yes | Formic Acid (Mite Away Quick Strips or Formic Pro) | No | NA | none |
| 2861 | 2022 | 17.01562 | Yes | Sugar roll | Yes | Formic Acid (Mite Away Quick Strips or Formic Pro) | No | NA | none |
| 2862 | 2022 | 9.296276 | No | NA | Yes | Other | No | NA | none |
| 2863 | 2022 | 9.296276 | No | NA | Yes | Other | No | NA | none |
| 2864 | 2022 | 14.96311 | Yes | Other | No | none | No | NA | none |
| 2865 | 2022 | 11.64321 | Yes | Alcohol wash | Yes | Apivar (Amitraz) | Yes | Fondant or sugar candy | sugar only |
| 2866 | 2022 | 11.64321 | Yes | Alcohol wash | Yes | Apivar (Amitraz) | Yes | Fondant or sugar candy | sugar only |
| 2867 | 2022 | 22.79707 | No | NA | No | none | Yes | Fondant or sugar candy | sugar only |
| 2868 | 2022 | 10.89061 | Yes | Sugar roll | No | none | Yes | Fondant or sugar candy, Honey from your own stock | none |
| 2869 | 2022 | 12.30567 | Yes | Sugar roll | Yes | Formic Acid (Mite Away Quick Strips or Formic Pro) | No | NA | none |
| 2870 | 2022 | 12.30567 | Yes | Sugar roll | Yes | Formic Acid (Mite Away Quick Strips or Formic Pro) | No | NA | none |
| 2871 | 2022 | 12.30567 | Yes | Sugar roll | Yes | Formic Acid (Mite Away Quick Strips or Formic Pro) | No | NA | none |
| 2872 | 2022 | 12.30567 | Yes | Sugar roll | Yes | Formic Acid (Mite Away Quick Strips or Formic Pro) | No | NA | none |
| 2873 | 2022 | 12.30567 | Yes | Sugar roll | Yes | Formic Acid (Mite Away Quick Strips or Formic Pro) | No | NA | none |
| 2874 | 2022 | 10.03575 | No | NA | Yes | Formic Acid (Mite Away Quick Strips or Formic Pro) | Yes | Fondant or sugar candy, Dry sugar | none |
| 2875 | 2022 | 10.03575 | No | NA | Yes | Formic Acid (Mite Away Quick Strips or Formic Pro) | Yes | Fondant or sugar candy, Dry sugar | none |
| 2876 | 2022 | 10.03575 | No | NA | Yes | Formic Acid (Mite Away Quick Strips or Formic Pro) | Yes | Fondant or sugar candy, Dry sugar | none |
| 2877 | 2022 | 10.03575 | No | NA | Yes | Formic Acid (Mite Away Quick Strips or Formic Pro) | Yes | Fondant or sugar candy, Dry sugar | none |
| 2878 | 2022 | 10.03575 | No | NA | Yes | Formic Acid (Mite Away Quick Strips or Formic Pro) | Yes | Fondant or sugar candy, Dry sugar | none |
| 2879 | 2022 | 10.03575 | No | NA | Yes | Formic Acid (Mite Away Quick Strips or Formic Pro) | Yes | Fondant or sugar candy, Dry sugar | none |
| 2880 | 2022 | 10.03575 | No | NA | Yes | Formic Acid (Mite Away Quick Strips or Formic Pro) | Yes | Fondant or sugar candy, Dry sugar | none |
| 2881 | 2022 | 10.03575 | No | NA | Yes | Formic Acid (Mite Away Quick Strips or Formic Pro) | Yes | Fondant or sugar candy, Dry sugar | none |
| 2882 | 2022 | 10.03575 | No | NA | Yes | Formic Acid (Mite Away Quick Strips or Formic Pro) | Yes | Fondant or sugar candy, Dry sugar | none |
| 2883 | 2022 | 0.993985 | Yes | 48 hr drop (sticky board), Drone brood inspection | Yes | Apivar (Amitraz) | Yes | Fondant or sugar candy, Pollen substitute | none |
| 2884 | 2022 | 0.993985 | Yes | 48 hr drop (sticky board), Drone brood inspection | Yes | Apivar (Amitraz) | Yes | Fondant or sugar candy, Pollen substitute | none |
| 2885 | 2022 | 0.993985 | Yes | 48 hr drop (sticky board), Drone brood inspection | Yes | Apivar (Amitraz) | Yes | Fondant or sugar candy, Pollen substitute | none |
| 2886 | 2022 | 8.93014 | Yes | Alcohol wash | Yes | Apivar (Amitraz) | Yes | Fondant or sugar candy, Sugar syrup, Honey from your own stock | none |
| 2887 | 2022 | 8.93014 | Yes | Alcohol wash | Yes | Apivar (Amitraz) | Yes | Fondant or sugar candy, Sugar syrup, Honey from your own stock | none |
| 2888 | 2022 | 0.810432 | No | NA | No | none | Yes | Fondant or sugar candy | sugar only |
| 2889 | 2022 | 6.60026 | Yes | Alcohol wash | No | none | Yes | Fondant or sugar candy | sugar only |
| 2890 | 2022 | 6.60026 | Yes | Alcohol wash | No | none | Yes | Fondant or sugar candy | sugar only |
| 2891 | 2022 | 6.60026 | Yes | Alcohol wash | No | none | Yes | Fondant or sugar candy | sugar only |
| 2892 | 2022 | 14.3838 | No | NA | No | none | No | NA | none |
| 2893 | 2022 | 14.3838 | No | NA | No | none | No | NA | none |
| 2894 | 2022 | 5.056134 | Yes | Alcohol wash | No | none | Yes | Sugar syrup, Dry sugar | none |
| 2895 | 2022 | 5.056134 | Yes | Alcohol wash | No | none | Yes | Sugar syrup, Dry sugar | none |
| 2896 | 2022 | 5.056134 | Yes | Alcohol wash | No | none | Yes | Sugar syrup, Dry sugar | none |
| 2897 | 2022 | 5.056134 | Yes | Alcohol wash | No | none | Yes | Sugar syrup, Dry sugar | none |
| 2898 | 2022 | 5.056134 | Yes | Alcohol wash | No | none | Yes | Sugar syrup, Dry sugar | none |
| 2899 | 2022 | 5.056134 | Yes | Alcohol wash | No | none | Yes | Sugar syrup, Dry sugar | none |
| 2900 | 2022 | 7.863567 | No | NA | Yes | Formic Acid (Mite Away Quick Strips or Formic Pro) | Yes | Dry sugar | sugar only |
| 2901 | 2022 | 7.863567 | No | NA | Yes | Formic Acid (Mite Away Quick Strips or Formic Pro) | Yes | Dry sugar | sugar only |
| 2902 | 2022 | 6.977097 | Yes | Alcohol wash | Yes | Oxalic Acid (Vapor) | Yes | Fondant or sugar candy | sugar only |
| 2903 | 2022 | 6.977097 | Yes | Alcohol wash | Yes | Oxalic Acid (Vapor) | Yes | Fondant or sugar candy | sugar only |
| 2904 | 2022 | 0.886181 | Yes | Sugar roll | No | none | Yes | Fondant or sugar candy, Commercially available supplements | none |
| 2905 | 2022 | 0.886181 | Yes | Sugar roll | No | none | Yes | Fondant or sugar candy, Commercially available supplements | none |
| 2906 | 2022 | 0.886181 | Yes | Sugar roll | No | none | Yes | Fondant or sugar candy, Commercially available supplements | none |
| 2907 | 2022 | 3.037704 | Yes | Sugar roll | Yes | Oxalic Acid (Dribble) | Yes | Fondant or sugar candy, Pollen substitute, Honey from your own stock | none |
| 2908 | 2022 | 3.037704 | Yes | Sugar roll | Yes | Oxalic Acid (Dribble) | Yes | Fondant or sugar candy, Pollen substitute, Honey from your own stock | none |
| 2909 | 2022 | 8.121927 | Yes | Sugar roll | Yes | Formic Acid (Mite Away Quick Strips or Formic Pro) | Yes | Fondant or sugar candy | sugar only |
| 2910 | 2022 | 8.121927 | Yes | Sugar roll | Yes | Formic Acid (Mite Away Quick Strips or Formic Pro) | Yes | Fondant or sugar candy | sugar only |
| 2911 | 2022 | 7.472901 | Yes | Sugar roll | Yes | Hopguard | Yes | Sugar syrup, Dry sugar | none |
| 2912 | 2022 | 7.472901 | Yes | Sugar roll | Yes | Hopguard | Yes | Sugar syrup, Dry sugar | none |
| 2913 | 2022 | 7.472901 | Yes | Sugar roll | Yes | Hopguard | Yes | Sugar syrup, Dry sugar | none |
| 2914 | 2022 | 7.472901 | Yes | Sugar roll | Yes | Hopguard | Yes | Sugar syrup, Dry sugar | none |
| 2915 | 2022 | 7.472901 | Yes | Sugar roll | Yes | Hopguard | Yes | Sugar syrup, Dry sugar | none |
| 2916 | 2022 | 7.472901 | Yes | Sugar roll | Yes | Hopguard | Yes | Sugar syrup, Dry sugar | none |
| 2917 | 2022 | 14.56617 | No | NA | No | none | Yes | Sugar syrup | none |
| 2918 | 2022 | 14.56617 | No | NA | No | none | Yes | Sugar syrup | none |
| 2919 | 2022 | 2.483499 | No | NA | Yes | Apivar (Amitraz) | Yes | Fondant or sugar candy, Commercially available supplements | none |
| 2920 | 2022 | 2.483499 | No | NA | Yes | Apivar (Amitraz) | Yes | Fondant or sugar candy, Commercially available supplements | none |
| 2921 | 2022 | 2.483499 | No | NA | Yes | Apivar (Amitraz) | Yes | Fondant or sugar candy, Commercially available supplements | none |
| 2922 | 2022 | 2.114381 | Yes | Alcohol wash | Yes | Formic Acid (Mite Away Quick Strips or Formic Pro) | Yes | Fondant or sugar candy, Pollen substitute | none |
| 2923 | 2022 | 2.114381 | Yes | Alcohol wash | Yes | Formic Acid (Mite Away Quick Strips or Formic Pro) | Yes | Fondant or sugar candy, Pollen substitute | none |
| 2924 | 2022 | 2.114381 | Yes | Alcohol wash | Yes | Formic Acid (Mite Away Quick Strips or Formic Pro) | Yes | Fondant or sugar candy, Pollen substitute | none |
| 2925 | 2022 | 2.114381 | Yes | Alcohol wash | Yes | Formic Acid (Mite Away Quick Strips or Formic Pro) | Yes | Fondant or sugar candy, Pollen substitute | none |
| 2926 | 2022 | 2.114381 | Yes | Alcohol wash | Yes | Formic Acid (Mite Away Quick Strips or Formic Pro) | Yes | Fondant or sugar candy, Pollen substitute | none |
| 2927 | 2022 | 2.114381 | Yes | Alcohol wash | Yes | Formic Acid (Mite Away Quick Strips or Formic Pro) | Yes | Fondant or sugar candy, Pollen substitute | none |
| 2928 | 2022 | 2.114381 | Yes | Alcohol wash | Yes | Formic Acid (Mite Away Quick Strips or Formic Pro) | Yes | Fondant or sugar candy, Pollen substitute | none |
| 2929 | 2022 | 2.114381 | Yes | Alcohol wash | Yes | Formic Acid (Mite Away Quick Strips or Formic Pro) | Yes | Fondant or sugar candy, Pollen substitute | none |
| 2930 | 2022 | 2.114381 | Yes | Alcohol wash | Yes | Formic Acid (Mite Away Quick Strips or Formic Pro) | Yes | Fondant or sugar candy, Pollen substitute | none |
| 2931 | 2022 | 2.114381 | Yes | Alcohol wash | Yes | Formic Acid (Mite Away Quick Strips or Formic Pro) | Yes | Fondant or sugar candy, Pollen substitute | none |
| 2932 | 2022 | 2.114381 | Yes | Alcohol wash | Yes | Formic Acid (Mite Away Quick Strips or Formic Pro) | Yes | Fondant or sugar candy, Pollen substitute | none |
| 2933 | 2022 | 2.114381 | Yes | Alcohol wash | Yes | Formic Acid (Mite Away Quick Strips or Formic Pro) | Yes | Fondant or sugar candy, Pollen substitute | none |
| 2934 | 2022 | 2.114381 | Yes | Alcohol wash | Yes | Formic Acid (Mite Away Quick Strips or Formic Pro) | Yes | Fondant or sugar candy, Pollen substitute | none |
| 2935 | 2022 | 2.114381 | Yes | Alcohol wash | Yes | Formic Acid (Mite Away Quick Strips or Formic Pro) | Yes | Fondant or sugar candy, Pollen substitute | none |
| 2936 | 2022 | 2.114381 | Yes | Alcohol wash | Yes | Formic Acid (Mite Away Quick Strips or Formic Pro) | Yes | Fondant or sugar candy, Pollen substitute | none |
| 2937 | 2022 | 2.114381 | Yes | Alcohol wash | Yes | Formic Acid (Mite Away Quick Strips or Formic Pro) | Yes | Fondant or sugar candy, Pollen substitute | none |
| 2938 | 2022 | 2.114381 | Yes | Alcohol wash | Yes | Formic Acid (Mite Away Quick Strips or Formic Pro) | Yes | Fondant or sugar candy, Pollen substitute | none |
| 2939 | 2022 | 2.114381 | Yes | Alcohol wash | Yes | Formic Acid (Mite Away Quick Strips or Formic Pro) | Yes | Fondant or sugar candy, Pollen substitute | none |
| 2940 | 2022 | 2.114381 | Yes | Alcohol wash | Yes | Formic Acid (Mite Away Quick Strips or Formic Pro) | Yes | Fondant or sugar candy, Pollen substitute | none |
| 2941 | 2022 | 2.114381 | Yes | Alcohol wash | Yes | Formic Acid (Mite Away Quick Strips or Formic Pro) | Yes | Fondant or sugar candy, Pollen substitute | none |
| 2942 | 2022 | 2.114381 | Yes | Alcohol wash | Yes | Formic Acid (Mite Away Quick Strips or Formic Pro) | Yes | Fondant or sugar candy, Pollen substitute | none |
| 2943 | 2022 | 2.114381 | Yes | Alcohol wash | Yes | Formic Acid (Mite Away Quick Strips or Formic Pro) | Yes | Fondant or sugar candy, Pollen substitute | none |
| 2944 | 2022 | 0.234092 | No | NA | Yes | Formic Acid (Mite Away Quick Strips or Formic Pro) | Yes | Dry sugar | sugar only |
| 2945 | 2022 | 0.234092 | No | NA | Yes | Formic Acid (Mite Away Quick Strips or Formic Pro) | Yes | Dry sugar | sugar only |
| 2946 | 2022 | 2.483499 | No | NA | Yes | Apivar (Amitraz) | Yes | Fondant or sugar candy, Commercially available supplements | none |
| 2947 | 2022 | 2.483499 | No | NA | Yes | Apivar (Amitraz) | Yes | Fondant or sugar candy, Commercially available supplements | none |
| 2948 | 2022 | 2.483499 | No | NA | Yes | Apivar (Amitraz) | Yes | Fondant or sugar candy, Commercially available supplements | none |
| 2949 | 2022 | 10.91372 | Yes | Alcohol wash | Yes | Oxalic Acid (Dribble) | Yes | Dry sugar | sugar only |
| 2950 | 2022 | 10.91372 | Yes | Alcohol wash | Yes | Oxalic Acid (Dribble) | Yes | Dry sugar | sugar only |
| 2951 | 2022 | 2.632791 | No | NA | Yes | Hopguard | No | NA | none |
| 2952 | 2022 | 16.66484 | Yes | Alcohol wash | Yes | Oxalic Acid (Dribble) | Yes | Fondant or sugar candy, Honey from your own stock | none |
| 2953 | 2022 | 11.91571 | No | NA | No | none | No | NA | none |
| 2954 | 2022 | 17.74132 | Yes | Drone brood inspection | Yes | Formic Acid (Mite Away Quick Strips or Formic Pro) | Yes | Commercially available supplements | none |
| 2955 | 2022 | 17.74132 | Yes | Drone brood inspection | Yes | Formic Acid (Mite Away Quick Strips or Formic Pro) | Yes | Commercially available supplements | none |
| 2956 | 2022 | 17.74132 | Yes | Drone brood inspection | Yes | Formic Acid (Mite Away Quick Strips or Formic Pro) | Yes | Commercially available supplements | none |
| 2957 | 2022 | 17.74132 | Yes | Drone brood inspection | Yes | Formic Acid (Mite Away Quick Strips or Formic Pro) | Yes | Commercially available supplements | none |
| 2958 | 2022 | 17.74132 | Yes | Drone brood inspection | Yes | Formic Acid (Mite Away Quick Strips or Formic Pro) | Yes | Commercially available supplements | none |
| 2959 | 2022 | 2.483499 | No | NA | Yes | Apivar (Amitraz) | Yes | Fondant or sugar candy, Commercially available supplements | none |
| 2960 | 2022 | 2.483499 | No | NA | Yes | Apivar (Amitraz) | Yes | Fondant or sugar candy, Commercially available supplements | none |
| 2961 | 2022 | 2.483499 | No | NA | Yes | Apivar (Amitraz) | Yes | Fondant or sugar candy, Commercially available supplements | none |
[truncated: 591,760 more chars]
